# Supplementary material for: Starchy staples production shortfalls in Ghana: Technical inefficiency effects outweigh technological differences across ecologies
Source: PLoS One. 2023 Apr 24;18(4):e0284780. doi: 10.1371/journal.pone.0284780 (PMC10124864; doi:10.1371/journal.pone.0284780)
Supplement: S1 File — (DOCX) [file pone.0284780.s001.docx]

# Appendix S: supplementary materials

#### **S1 Table.** Ecology Population, Climatic, and Soil Characteristics in Ghana.

| Variable | Sudan Savanah | Guinea Savanna | Transitional Zone | Semi-Deciduous Forest | Rain Forest | Coastal Savanna |
| --- | --- | --- | --- | --- | --- | --- |
| Area (1000 km^2^) | 50.12 | 25.92 | 14.21 | 16.66 | 18.48 | 10.81 |
| Pop. Den. (N/km^2^) | 59.09 | 162.76 | 140.14 | 89.61 | 37.99 | 115.97 |
| Agric Pop. (%) | 55.41 | 38.73 | 55.00 | 45.59 | 77.09 | 31.63 |
| Rural Pop. (%) | 48.50 | 38.39 | 56.68 | 48.42 | 79.46 | 30.48 |
| Soil pH | 4.28-7.40 | 4.13-7.44 | 4.10-7.66 | 4.21-7.34 | 4.40-6.70 | 4.29-8.28 |
| Soil Org. matter (%) | 0.52-6.74 | 0.06-7.63 | 0.00-11.18 | 0.05-11.31 | 0.19-13.83 | 0.00-4.84 |
| Soil N (%) | 0.00-0.11 | 0.00-0.18 | 0.00-0.39 | 0.00-0.39 | 0.00-0.37 | 0.00-0.25 |
| Soil P (mg/kg soil) | 0.00-4.54 | 0.00-6.35 | 0.00-3.41 | 0.00-3.92 | 0.00-0.26 | 0.00-7.51 |
| Soil CEC | 0.00-6.41 | 0.00-7.86 | 0.00-9.55 | 0.00-6.54 | 0.00-1.21 | 0.00-6.96 |
| 10-year avg rain |  |  |  |  |  |  |
| Mean [mm/year] | 943.29 | 1,128.52 | 1,244.52 | 1,310.65 | 1,443.30 | 1,064.89 |
| CV [%] | 17.55 | 10.54 | 9.85 | 9.57 | 13.36 | 10.93 |

All values are calculated as weighted averages (by area) using data retrieved from published reports Agriculture in Ghana: Facts and figures (2016)

#### **S2 Table.** Ecology Level Starchy Staple Production Statistics in Ghana for 2010/2015

| Ecology/Crop | Cultivating Household ^a^ | | | Farm  size (ha) ^a^ | Area  Share (%) ^b^ | Production  Share (%) ^b^ | Yield  ratio ^b^ |
| --- | --- | --- | --- | --- | --- | --- | --- |
|  | Count  (1000) | Share  (%) | Size  (count) |  |  |  |  |
| Cassava |  |  |  |  |  |  |  |
| Semi-Deciduous Forest | 1647.48 (1269.22) | 59.47 (6.27) | 5.06 (0.84) | 0.39 (0.22) | 46.54 (2.26) | 49.06 (3.00) | 1.00 (0.00) |
| Transitional Zone | 558.77 (490.46) | 18.48 (4.09) | 5.56 (0.81) | 0.81 (0.59) | 26.00 (1.36) | 27.42 (2.27) | 1.00 (0.09) |
| Coastal Savanna | 384.44 (396.35) | 10.58 (3.16) | 5.05 (1.03) | 0.83 (0.60) | 13.61 (0.62) | 11.60 (0.67) | 0.81 (0.06) |
| Guinea Savanna | 273.88 (323.54) | 6.17 (4.42) | 7.81 (1.56) | 2.35 (2.14) | 10.77 (3.71) | 10.24 (3.86) | 0.92 (0.25) |
| Rain Forest | 140.94 (105.37) | 5.18 (0.76) | 5.31 (0.96) | 0.25 (0.15) | 2.58 (0.28) | 1.51 (0.22) | 0.56 (0.05) |
| Sudan Savanah | 3.07 (2.59) | 0.14 (0.13) | 4.57 (0.91) | 3.73 (4.10) | 0.58 (0.38) | 0.19 (0.05) | 0.35 (0.10) |
| Yam |  |  |  |  |  |  |  |
| Transitional Zone | 298.94 (252.41) | 34.45 (8.29) | 5.64 (0.86) | 1.06 (0.62) | 50.73 (9.70) | 52.51 (10.28) | 1.46 (0.04) |
| Guinea Savanna | 441.81 (451.37) | 38.86 (9.20) | 7.78 (1.68) | 0.98 (0.96) | 31.30 (11.57) | 33.77 (12.01) | 1.54 (0.24) |
| Semi-Deciduous Forest | 164.93 (113.55) | 21.25 (5.56) | 5.04 (0.29) | 0.39 (0.22) | 12.03 (2.48) | 8.55 (1.81) | 1.00 (0.00) |
| Sudan Savanah | 39.68 (44.83) | 3.00 (1.36) | 6.67 (0.78) | 2.43 (2.30) | 5.27 (0.75) | 4.88 (0.25) | 1.32 (0.18) |
| Coastal Savanna | 27.83 (31.93) | 2.01 (1.14) | 3.86 (1.57) | 0.38 (0.34) | 0.57 (0.15) | 0.26 (0.11) | 0.62 (0.08) |
| Rain Forest | 3.85 (3.12) | 0.44 (0.31) | 5.60 (2.15) | 0.23 (0.19) | 0.10 (0.02) | 0.04 (0.01) | 0.50 (0.11) |
| Cocoyam |  |  |  |  |  |  |  |
| Semi-Deciduous Forest | 507.52 (417.81) | 76.76 (3.65) | 5.10 (0.95) | 0.64 (0.43) | 81.79 (9.17) | 83.96 (1.72) | 1.00 (0.00) |
| Transitional Zone | 96.49 (75.16) | 16.54 (5.19) | 5.50 (1.38) | 0.45 (0.27) | 12.22 (1.78) | 13.99 (1.69) | 1.11 (0.05) |
| Coastal Savanna | 31.59 (35.94) | 3.17 (2.25) | 3.99 (1.60) | 0.73 (0.83) | 1.38 (0.63) | 1.21 (0.75) | 0.82 (0.16) |
| Rain Forest | 31.89 (35.58) | 3.45 (1.81) | 5.53 (1.91) | 0.15 (0.12) | 0.62 (0.31) | 0.55 (0.41) | 0.82 (0.14) |
| Guinea Savanna | 0.33 (0.01) | 0.14 (0.03) | 7.40 (3.58) | 55.35 (116.28) | 6.39 (13.03) | 0.47 (0.05) | 0.69 (0.39) |
| Plantain |  |  |  |  |  |  |  |
| Semi-Deciduous Forest | 1259.52 (992.94) | 78.26 (3.50) | 5.01 (0.71) | 0.36 (0.21) | 80.01 (0.73) | 82.38 (0.82) | 1.00 (0.00) |
| Transitional Zone | 175.63 (144.96) | 10.84 (2.54) | 5.12 (0.90) | 0.59 (0.38) | 16.85 (0.42) | 15.29 (0.67) | 0.88 (0.03) |
| Coastal Savanna | 157.98 (173.84) | 7.02 (3.70) | 4.85 (0.85) | 0.19 (0.15) | 2.40 (0.53) | 1.74 (0.25) | 0.71 (0.08) |
| Rain Forest | 69.62 (62.34) | 3.87 (0.86) | 5.40 (1.43) | 0.08 (0.06) | 0.72 (0.05) | 0.57 (0.05) | 0.77 (0.05) |

**^a^** Calculated by authors from data retrieved from GLSS Waves 5-7

**^b^** Calculated by authors from data retrieved from FAO CountryStat

**S3 Table.** Stochastic Meta-Frontier Results for Cassava Production in Ghana (1987-2017)

|  | **Guinea Savanah** | **Transitional Zone** | **Forest Zone** | **Coastal Savanah** | **National** | **Meta-frontier** |
| --- | --- | --- | --- | --- | --- | --- |
| **Production function** |  |  |  |  |  |  |
| Land(ln[ha]) {lnI1} | -0.22** (0.099) | 0.23*** (0.062) | 0.16*** (0.034) | 0.16** (0.075) | 0.17*** (0.027) | 0.16*** (0.007) |
| Seed (ln[Mt]) {lnI2} | 0.18*** (0.061) | -0.07* (0.037) | 0.04** (0.019) | 0.07** (0.035) | 0.03** (0.015) | 0.04*** (0.004) |
| Hired labor(ln[days]) {lnI3} | 0.13 (0.189) | 0.23* (0.134) | 0.06 (0.073) | 0.15 (0.172) | 0.09 (0.059) | 0.13*** (0.015) |
| Family labor(ln[days]) {lnI4} | 0.17*** (0.044) | 0.10*** (0.032) | 0.10*** (0.015) | 0.08*** (0.030) | 0.10*** (0.012) | 0.10*** (0.003) |
| Pesticide (ln[liter]) {lnI5} | 0.10** (0.049) | 0.12*** (0.031) | 0.08*** (0.013) | 0.16*** (0.033) | 0.08*** (0.011) | 0.09*** (0.003) |
| Trend {I6} | -0.17 (0.198) | -0.17 (0.162) | 0.20*** (0.068) | -0.20* (0.118) | 0.03 (0.053) | 0.01 (0.013) |
| P5∙lnI1∙lnI1 | -0.04 (0.038) | -0.07*** (0.023) | -0.04*** (0.012) | -0.02 (0.026) | -0.05*** (0.009) | -0.04*** (0.003) |
| P5∙lnI2∙lnI2 | 0.05*** (0.010) | 0.00 (0.006) | 0.02*** (0.003) | 0.03*** (0.007) | 0.02*** (0.003) | 0.02*** (0.001) |
| P5∙lnI3∙lnI3 | 0.13 (0.171) | 0.11 (0.121) | -0.06 (0.066) | -0.05 (0.136) | -0.02 (0.050) | -0.01 (0.015) |
| P5∙lnI4∙lnI4 | 0.04*** (0.007) | 0.02*** (0.005) | 0.03*** (0.003) | 0.02*** (0.005) | 0.03*** (0.002) | 0.03*** (0.001) |
| P5∙lnI5∙lnI5 | 0.02*** (0.009) | 0.03*** (0.006) | 0.02*** (0.003) | 0.04*** (0.008) | 0.02*** (0.002) | 0.02*** (0.001) |
| P5∙I6∙I6 | 0.02*** (0.009) | 0.03*** (0.006) | 0.02*** (0.003) | 0.04*** (0.008) | 0.02*** (0.002) | 0.02*** (0.001) |
| lnI1.lnI2 | 0.00 (0.008) | 0.01** (0.005) | 0.00 (0.003) | -0.01** (0.005) | 0.00 (0.002) | 0.00 (0.001) |
| lnI1.lnI3 | 0.09* (0.048) | -0.01 (0.035) | -0.02 (0.018) | -0.04 (0.038) | -0.01 (0.014) | -0.01** (0.004) |
| lnI1.lnI4 | -0.02** (0.007) | -0.01 (0.005) | 0.00 (0.002) | 0.01** (0.004) | 0.00* (0.002) | 0.00*** (0.001) |
| lnI1.lnI5 | -0.01 (0.007) | 0.01* (0.005) | -0.01*** (0.002) | 0.00 (0.005) | 0.00 (0.002) | 0.00*** (0.001) |
| lnI1.I6 | 0.02*** (0.003) | 0.01*** (0.002) | 0.01*** (0.001) | 0.01*** (0.002) | 0.01*** (0.001) | 0.01*** (0.000) |
| lnI2.lnI3 | 0.00 (0.015) | 0.00 (0.011) | -0.01 (0.006) | 0.01 (0.013) | 0.00 (0.005) | 0.00* (0.001) |
| lnI2.lnI4 | 0.00 (0.002) | 0.00** (0.001) | -0.01*** (0.001) | -0.01*** (0.001) | 0.00*** (0.001) | -0.01*** (0.000) |
| lnI2.lnI5 | 0.00 (0.002) | 0.00** (0.002) | 0.00 (0.001) | 0.00 (0.002) | 0.00** (0.001) | 0.00*** (0.000) |
| lnI2.I6 | 0.00 (0.001) | 0.01*** (0.001) | 0.00*** (0.000) | 0.00*** (0.001) | 0.00*** (0.000) | 0.00*** (0.000) |
| lnI3.lnI4 | -0.03** (0.014) | 0.03*** (0.010) | 0.00 (0.005) | 0.00 (0.011) | 0.00 (0.004) | 0.00 (0.001) |
| lnI3.lnI5 | 0.00 (0.015) | -0.01 (0.010) | 0.00 (0.005) | 0.00 (0.013) | 0.00 (0.004) | 0.00 (0.001) |
| lnI3.I6 | -0.01 (0.006) | 0.00 (0.004) | 0.00 (0.002) | 0.00 (0.005) | 0.00 (0.002) | 0.00 (0.000) |
| lnI4.lnI5 | 0.00 (0.002) | 0.00 (0.001) | 0.00 (0.001) | 0.00 (0.001) | 0.00 (0.001) | 0.00*** (0.000) |
| lnI4.I6 | 0.00 (0.001) | 0.00** (0.001) | 0.00 (0.000) | 0.00 (0.001) | 0.00 (0.000) | 0.00*** (0.000) |
| lnI5.I6 | 0.00* (0.001) | 0.00 (0.001) | 0.00 (0.000) | 0.00* (0.001) | 0.00* (0.000) | 0.00*** (0.000) |
| Constant | -1.92 (2.991) | 2.20 (1.939) | -0.94 (0.919) | 0.13 (1.568) | 0.80 (0.694) | 1.36*** (0.184) |
| Source (base=GLSS7) |  |  |  |  |  |  |
| GLSS1 | 2.14 (2.913) | -1.63 (1.961) | 1.11 (0.917) | -0.36 (1.564) | -0.47 (0.697) | -0.75*** (0.186) |
| GLSS2 | 2.19 (2.753) | -2.41 (1.846) | 0.75 (0.876) | 0.21 (1.498) | -0.74 (0.662) | -1.32*** (0.177) |
| GLSS3 | 2.37 (2.348) | -1.91 (1.511) | 0.07 (0.782) | 0.14 (1.301) | -0.98* (0.575) | -1.58*** (0.158) |
| GLSS4 | 3.43** (1.719) | -0.86 (1.084) | -0.44 (0.634) | 0.78 (1.004) | -0.77* (0.451) | -1.24*** (0.129) |
| GLSS5 | 3.43*** (1.136) | 0.08 (0.793) | -0.85* (0.464) | 1.25* (0.743) | -0.58* (0.333) | -0.91*** (0.095) |
| GLSS6 | 2.11*** (0.496) | 0.54 (0.365) | -0.41* (0.215) | -0.22 (0.359) | -0.20 (0.156) | -0.09* (0.052) |
| GSPS1 | 2.12*** (0.759) | 0.57 (0.561) | -0.91*** (0.334) | 1.47*** (0.544) | -0.38 (0.240) | -0.30*** (0.069) |
| GSPS2 | 0.62 (0.382) | 0.69*** (0.244) | 0.10 (0.142) | -0.23 (0.294) | 0.07 (0.105) | 0.16*** (0.031) |
| **Uncertainty function** |  |  |  |  |  |  |
| Mean | -0.99*** (0.155) | -0.65*** (0.077) | -0.52*** (0.039) | -0.60*** (0.055) | -0.55*** (0.035) | -3.12*** (0.050) |
| Variance | 0.61 (0.047) | 0.72 (0.028) | 0.77 (0.015) | 0.74 (0.020) | 0.76 (0.013) | 0.21 (0.005) |

Significance levels: * p<0.10, ** p<0.05, ***p<0.01; Data Sources: Ghana Living Standards Surveys [wave 1-7] and Ghana Socioeconomic Panel Survey [wave 1-2]

**S4 Table.** Stochastic Meta-Frontier Results for Yam Production in Ghana (1987-2017)

|  | **Sudan Savanah** | **Guinea Savanah** | **Transitional Zone** | **Forest Zone** | **National** | **Meta-frontier** |
| --- | --- | --- | --- | --- | --- | --- |
| **Production function** |  |  |  |  |  |  |
| Land(ln[ha]) {lnI1} | 0.30 (0.282) | -0.10 (0.097) | 0.27*** (0.094) | 0.08 (0.127) | 0.18*** (0.057) | 0.06** (0.028) |
| Seed (ln[Mt]) {lnI2} | 0.32** (0.127) | 0.21*** (0.041) | 0.06 (0.051) | 0.20** (0.079) | 0.20*** (0.029) | 0.22*** (0.011) |
| Hired labor(ln[days]) {lnI3} | 1.71*** (0.450) | -0.10 (0.159) | -0.12 (0.221) | 0.41 (0.331) | 0.09 (0.120) | 0.17** (0.073) |
| Family labor(ln[days]) {lnI4} | 0.21** (0.103) | 0.12*** (0.029) | 0.19*** (0.048) | 0.16*** (0.061) | 0.18*** (0.024) | 0.18*** (0.011) |
| Pesticide (ln[liter]) {lnI5} | -0.22* (0.119) | 0.21*** (0.044) | 0.13*** (0.044) | 0.23*** (0.053) | 0.17*** (0.026) | 0.15*** (0.011) |
| Trend {I6} | 0.49 (0.627) | -0.62*** (0.168) | 0.05 (0.383) | 0.99*** (0.182) | -0.08 (0.122) | -0.36*** (0.062) |
| P5∙lnI1∙lnI1 | 0.00 (0.068) | 0.01 (0.025) | -0.10*** (0.030) | -0.09*** (0.032) | -0.04** (0.017) | 0.00 (0.009) |
| P5∙lnI2∙lnI2 | 0.09*** (0.015) | 0.06*** (0.008) | 0.03*** (0.008) | 0.04** (0.016) | 0.05*** (0.005) | 0.05*** (0.002) |
| P5∙lnI3∙lnI3 | 0.12 (0.304) | 0.10 (0.137) | -0.12 (0.159) | -0.10 (0.216) | -0.05 (0.094) | -0.05 (0.038) |
| P5∙lnI4∙lnI4 | 0.05*** (0.017) | 0.03*** (0.005) | 0.04*** (0.007) | 0.05*** (0.008) | 0.04*** (0.004) | 0.05*** (0.002) |
| P5∙lnI5∙lnI5 | 0.02 (0.019) | 0.04*** (0.007) | 0.02** (0.010) | 0.03*** (0.010) | 0.04*** (0.005) | 0.04*** (0.002) |
| P5∙I6∙I6 | 0.02 (0.019) | 0.04*** (0.007) | 0.02** (0.010) | 0.03*** (0.010) | 0.04*** (0.005) | 0.04*** (0.002) |
| lnI1.lnI2 | -0.01 (0.016) | -0.02*** (0.007) | 0.00 (0.007) | 0.00 (0.011) | -0.02*** (0.004) | -0.02*** (0.002) |
| lnI1.lnI3 | 0.13 (0.101) | 0.07 (0.044) | 0.02 (0.047) | 0.01 (0.055) | 0.05* (0.028) | 0.07*** (0.013) |
| lnI1.lnI4 | -0.02 (0.018) | 0.00 (0.006) | 0.00 (0.007) | 0.01 (0.009) | -0.01 (0.004) | -0.01*** (0.002) |
| lnI1.lnI5 | -0.01 (0.016) | -0.02*** (0.007) | -0.01 (0.006) | -0.01 (0.008) | -0.01 (0.004) | -0.01*** (0.002) |
| lnI1.I6 | -0.01 (0.009) | 0.02*** (0.003) | 0.01*** (0.003) | 0.01** (0.003) | 0.01*** (0.002) | 0.01*** (0.001) |
| lnI2.lnI3 | 0.06** (0.027) | 0.00 (0.011) | 0.00 (0.015) | 0.00 (0.027) | 0.00 (0.008) | -0.01** (0.003) |
| lnI2.lnI4 | 0.00 (0.004) | 0.00*** (0.001) | 0.00 (0.002) | -0.01 (0.005) | 0.00* (0.001) | 0.00*** (0.000) |
| lnI2.lnI5 | -0.01 (0.006) | 0.00 (0.002) | 0.00 (0.002) | 0.01* (0.003) | 0.00 (0.001) | 0.00 (0.000) |
| lnI2.I6 | 0.00 (0.004) | 0.00*** (0.001) | 0.01*** (0.001) | 0.00 (0.002) | 0.00*** (0.001) | 0.00*** (0.000) |
| lnI3.lnI4 | -0.01 (0.028) | -0.01 (0.011) | -0.01 (0.016) | -0.01 (0.020) | -0.02** (0.008) | -0.01*** (0.003) |
| lnI3.lnI5 | 0.00 (0.030) | -0.02 (0.013) | 0.01 (0.015) | 0.01 (0.019) | -0.01 (0.009) | -0.01* (0.004) |
| lnI3.I6 | -0.05*** (0.013) | 0.00 (0.005) | 0.00 (0.008) | -0.01 (0.008) | 0.00 (0.004) | -0.01*** (0.003) |
| lnI4.lnI5 | -0.01 (0.004) | 0.00 (0.001) | 0.00 (0.002) | 0.00 (0.003) | 0.00 (0.001) | 0.00*** (0.000) |
| lnI4.I6 | 0.00 (0.002) | 0.00 (0.001) | 0.00 (0.001) | 0.00*** (0.001) | 0.00 (0.001) | 0.00*** (0.000) |
| lnI5.I6 | 0.01*** (0.003) | 0.00 (0.001) | 0.00 (0.001) | 0.00 (0.001) | 0.00 (0.001) | 0.00** (0.000) |
| Constant | -11.98 (9.086) | 4.49* (2.390) | -1.90 (4.634) | -6.20** (2.491) | 0.63 (1.699) | 3.67*** (0.860) |
| Source (base=GLSS7) |  |  |  |  |  |  |
| GLSS1 | 11.33 (9.002) | -3.96* (2.311) | 2.87 (4.613) | 7.42*** (2.515) | 0.38 (1.685) | -2.56*** (0.881) |
| GLSS2 | 9.78 (8.294) | -3.68* (2.172) | 1.41 (4.289) | 6.37*** (2.387) | 0.03 (1.587) | -2.64*** (0.803) |
| GLSS3 | 9.18 (7.044) | -1.65 (1.873) | 2.07 (3.371) | 3.10 (2.120) | 0.36 (1.356) | -1.51** (0.662) |
| GLSS4 | 5.49 (4.128) | 0.89 (1.273) | 2.66 (2.007) | -1.46 (1.644) | 1.01 (0.902) | 0.79** (0.402) |
| GLSS5 | 4.41** (2.203) | 2.44*** (0.828) | 1.13 (1.322) | -2.94** (1.190) | 0.80 (0.621) | 1.18*** (0.256) |
| GLSS6 | 2.22*** (0.736) | 1.80*** (0.356) | 0.91 (0.663) | -2.46*** (0.572) | 0.76*** (0.271) | 1.39*** (0.100) |
| GSPS1 | 2.92** (1.191) | 2.15*** (0.550) | 1.23 (0.945) | -2.29*** (0.831) | 0.75* (0.410) | 1.12*** (0.167) |
| GSPS2 | 1.48*** (0.393) | 1.92*** (0.239) | -0.53 (0.496) | -2.12*** (0.417) | 1.29*** (0.186) | 1.94*** (0.057) |
| **Uncertainty function** |  |  |  |  |  |  |
| Mean | -1.33*** (0.223) | -1.27*** (0.160) | -0.84*** (0.217) | -0.57*** (0.139) | -1.05*** (0.091) | -2.68*** (0.075) |
| Variance | 0.51 (0.057) | 0.53 (0.042) | 0.66 (0.071) | 0.75 (0.053) | 0.59 (0.027) | 0.26 (0.010) |

Significance levels: * p<0.10, ** p<0.05, ***p<0.01; Data Sources: Ghana Living Standards Surveys [wave 1-7] and Ghana Socioeconomic Panel Survey [wave 1-2]

**S5 Table.** Stochastic Meta-Frontier Results for Cocoyam Production in Ghana (1987-2017)

|  | **Transitional Zone** | **Forest Zone** | **National** | **Meta-frontier** |
| --- | --- | --- | --- | --- |
| **Production function** |  |  |  |  |
| Land(ln[ha]) {lnI1} | 0.00 (0.097) | 0.14 (0.092) | 0.11 (0.071) | 0.13*** (0.029) |
| Seed (ln[Mt]) {lnI2} | 0.21** (0.107) | 0.20*** (0.048) | 0.18*** (0.046) | 0.19*** (0.023) |
| Hired labor(ln[days]) {lnI3} | 0.19 (0.322) | 0.02 (0.202) | -0.04 (0.192) | -0.02 (0.071) |
| Family labor(ln[days]) {lnI4} | 0.16** (0.080) | 0.12*** (0.038) | 0.13*** (0.035) | 0.13*** (0.017) |
| Pesticide (ln[liter]) {lnI5} | 0.11 (0.079) | 0.01 (0.042) | 0.02 (0.037) | 0.02 (0.014) |
| Trend {I6} | -0.87 (0.865) | 0.11 (0.223) | -0.09 (0.189) | -0.06 (0.067) |
| P5∙lnI1∙lnI1 | -0.19*** (0.046) | -0.13*** (0.029) | -0.14*** (0.025) | -0.14*** (0.008) |
| P5∙lnI2∙lnI2 | 0.07*** (0.023) | 0.04*** (0.010) | 0.04*** (0.010) | 0.04*** (0.006) |
| P5∙lnI3∙lnI3 | 0.27 (0.258) | -0.05 (0.168) | 0.01 (0.146) | -0.06 (0.040) |
| P5∙lnI4∙lnI4 | 0.04*** (0.011) | 0.02*** (0.006) | 0.03*** (0.005) | 0.02*** (0.001) |
| P5∙lnI5∙lnI5 | 0.03 (0.017) | -0.01 (0.008) | 0.00 (0.007) | 0.00 (0.002) |
| P5∙I6∙I6 | 0.03 (0.017) | -0.01 (0.008) | 0.00 (0.007) | 0.00 (0.002) |
| lnI1.lnI2 | -0.01 (0.009) | -0.01 (0.008) | -0.01 (0.007) | -0.01** (0.004) |
| lnI1.lnI3 | -0.01 (0.067) | 0.03 (0.040) | 0.01 (0.037) | 0.02 (0.010) |
| lnI1.lnI4 | 0.02* (0.010) | 0.01 (0.005) | 0.01 (0.005) | 0.00*** (0.002) |
| lnI1.lnI5 | 0.00 (0.010) | 0.00 (0.006) | 0.00 (0.005) | 0.00 (0.001) |
| lnI1.I6 | 0.00 (0.005) | 0.01** (0.002) | 0.00* (0.002) | 0.00*** (0.000) |
| lnI2.lnI3 | 0.00 (0.029) | 0.00 (0.019) | -0.01 (0.018) | -0.02** (0.009) |
| lnI2.lnI4 | -0.01** (0.005) | 0.00 (0.003) | -0.01** (0.003) | -0.01*** (0.002) |
| lnI2.lnI5 | 0.00 (0.003) | 0.00 (0.002) | 0.00 (0.002) | 0.00 (0.001) |
| lnI2.I6 | 0.01* (0.003) | 0.00 (0.001) | 0.00 (0.001) | 0.00 (0.001) |
| lnI3.lnI4 | 0.04* (0.024) | 0.00 (0.014) | 0.01 (0.012) | 0.02*** (0.004) |
| lnI3.lnI5 | 0.00 (0.027) | 0.00 (0.015) | 0.00 (0.013) | 0.00 (0.004) |
| lnI3.I6 | 0.01 (0.010) | 0.00 (0.005) | 0.00 (0.005) | 0.00*** (0.001) |
| lnI4.lnI5 | 0.00 (0.004) | 0.00 (0.002) | 0.00 (0.002) | 0.00*** (0.001) |
| lnI4.I6 | 0.00*** (0.001) | 0.00 (0.001) | 0.00** (0.001) | 0.00*** (0.000) |
| lnI5.I6 | 0.00 (0.002) | 0.00 (0.001) | 0.00 (0.001) | 0.00*** (0.000) |
| Constant | 18.24* (9.490) | -2.33 (3.801) | 3.53 (3.063) | 4.45*** (1.083) |
| Source (base=GLSS7) |  |  |  |  |
| GLSS1 | -18.15* (9.547) | 3.40 (3.814) | -2.70 (3.053) | -3.46*** (1.094) |
| GLSS2 | -17.32** (8.795) | 2.84 (3.755) | -2.88 (2.990) | -3.79*** (1.085) |
| GLSS3 | -16.01** (6.824) | 3.02 (3.629) | -2.67 (2.887) | -2.92*** (1.065) |
| GLSS4 | -10.38** (4.212) | 2.33 (3.289) | -2.21 (2.576) | -2.40** (0.977) |
| GLSS5 | -4.93* (2.975) | 1.24 (2.540) | -1.10 (2.020) | -1.59** (0.743) |
| GLSS6 | -0.94 (1.651) | 0.24 (1.141) | -0.41 (0.932) | -0.44 (0.340) |
| GSPS1 | -2.93 (2.241) | 0.50 (1.727) | -1.04 (1.400) | -1.52*** (0.545) |
| GSPS2 | -1.06 (1.158) | 0.02 (0.660) | -0.56 (0.557) | -0.51*** (0.196) |
| **Uncertainty function** |  |  |  |  |
| Mean | -0.58*** (0.196) | -0.66*** (0.241) | -0.37*** (0.123) | -2.94*** (0.110) |
| Variance | 0.75 (0.073) | 0.72 (0.087) | 0.83 (0.051) | 0.23 (0.013) |

Significance levels: * p<0.10, ** p<0.05, ***p<0.01; Data Sources: Ghana Living Standards Surveys [wave 1-7] and Ghana Socioeconomic Panel Survey [wave 1-2]

**S6 Table.** Stochastic Meta-Frontier Results for Plantain Production in Ghana (1987-2017)

|  | **Transitional Zone** | **Forest Zone** | **Coastal Savanah** | **National** | **Meta-frontier** |
| --- | --- | --- | --- | --- | --- |
| **Production function** |  |  |  |  |  |
| Land(ln[ha]) {lnI1} | 0.35*** (0.106) | 0.31*** (0.044) | 0.24* (0.138) | 0.32*** (0.038) | 0.33*** (0.009) |
| Seed (ln[Mt]) {lnI2} | 0.04 (0.062) | 0.03 (0.023) | -0.06 (0.057) | 0.03 (0.020) | 0.03*** (0.005) |
| Hired labor(ln[days]) {lnI3} | 0.02 (0.206) | 0.07 (0.100) | -0.17 (0.292) | 0.07 (0.088) | 0.03 (0.025) |
| Family labor(ln[days]) {lnI4} | 0.13*** (0.048) | 0.13*** (0.019) | 0.11* (0.066) | 0.13*** (0.017) | 0.13*** (0.004) |
| Pesticide (ln[liter]) {lnI5} | 0.06 (0.055) | 0.05*** (0.017) | 0.16*** (0.049) | 0.06*** (0.016) | 0.06*** (0.004) |
| Trend {I6} | 0.40* (0.215) | 0.05 (0.079) | -0.13 (0.255) | 0.09 (0.070) | 0.17*** (0.021) |
| P5∙lnI1∙lnI1 | -0.04 (0.033) | -0.01 (0.013) | -0.09** (0.038) | -0.01 (0.012) | -0.01*** (0.002) |
| P5∙lnI2∙lnI2 | 0.02** (0.009) | 0.02*** (0.004) | -0.02 (0.012) | 0.02*** (0.003) | 0.02*** (0.001) |
| P5∙lnI3∙lnI3 | 0.11 (0.166) | -0.11 (0.084) | -0.23 (0.230) | -0.07 (0.072) | -0.04** (0.019) |
| P5∙lnI4∙lnI4 | 0.03*** (0.008) | 0.02*** (0.003) | 0.04*** (0.009) | 0.02*** (0.003) | 0.03*** (0.001) |
| P5∙lnI5∙lnI5 | 0.02** (0.010) | 0.01*** (0.003) | 0.04*** (0.011) | 0.02*** (0.003) | 0.01*** (0.001) |
| P5∙I6∙I6 | 0.02** (0.010) | 0.01*** (0.003) | 0.04*** (0.011) | 0.02*** (0.003) | 0.01*** (0.001) |
| lnI1.lnI2 | 0.01 (0.008) | 0.01* (0.004) | 0.00 (0.010) | 0.01** (0.003) | 0.01*** (0.001) |
| lnI1.lnI3 | 0.11** (0.050) | -0.01 (0.021) | 0.00 (0.057) | 0.01 (0.018) | 0.01*** (0.004) |
| lnI1.lnI4 | 0.00 (0.008) | 0.00 (0.003) | -0.02** (0.009) | 0.00 (0.003) | 0.00*** (0.001) |
| lnI1.lnI5 | 0.00 (0.008) | -0.01*** (0.003) | 0.02** (0.009) | -0.01** (0.003) | -0.01*** (0.001) |
| lnI1.I6 | 0.01** (0.003) | 0.01*** (0.001) | 0.01 (0.004) | 0.01*** (0.001) | 0.01*** (0.000) |
| lnI2.lnI3 | 0.01 (0.017) | -0.01 (0.008) | -0.01 (0.023) | -0.01 (0.007) | -0.01*** (0.002) |
| lnI2.lnI4 | -0.01** (0.002) | 0.00** (0.001) | 0.00 (0.005) | 0.00*** (0.001) | 0.00*** (0.000) |
| lnI2.lnI5 | 0.00* (0.003) | 0.00 (0.001) | 0.00 (0.003) | 0.00* (0.001) | 0.00*** (0.000) |
| lnI2.I6 | 0.00** (0.002) | 0.00*** (0.001) | 0.00* (0.002) | 0.00*** (0.000) | 0.00*** (0.000) |
| lnI3.lnI4 | 0.02 (0.016) | 0.00 (0.007) | 0.02 (0.019) | 0.01 (0.006) | 0.01*** (0.001) |
| lnI3.lnI5 | -0.01 (0.015) | 0.01 (0.007) | -0.02 (0.018) | 0.01 (0.006) | 0.00** (0.002) |
| lnI3.I6 | 0.02** (0.006) | 0.00 (0.003) | 0.01 (0.007) | 0.00 (0.003) | 0.01*** (0.001) |
| lnI4.lnI5 | 0.00 (0.002) | 0.00 (0.001) | 0.00 (0.003) | 0.00 (0.001) | 0.00 (0.000) |
| lnI4.I6 | 0.00 (0.001) | 0.00 (0.000) | 0.00* (0.001) | 0.00 (0.000) | 0.00*** (0.000) |
| lnI5.I6 | 0.00 (0.001) | 0.00** (0.000) | 0.00 (0.001) | 0.00** (0.000) | 0.00*** (0.000) |
| Constant | -1.36 (2.633) | -0.02 (1.119) | 0.85 (3.498) | -0.20 (0.943) | -1.04*** (0.257) |
| Source (base=GLSS7) |  |  |  |  |  |
| GLSS1 | 2.31 (2.633) | 0.44 (1.102) | -0.09 (3.486) | 0.83 (0.935) | 1.98*** (0.265) |
| GLSS2 | 2.05 (2.476) | 0.36 (1.057) | -1.04 (3.263) | 0.65 (0.892) | 2.03*** (0.246) |
| GLSS3 | 0.59 (2.087) | -0.04 (0.944) | -0.16 (2.715) | 0.10 (0.789) | 0.84*** (0.225) |
| GLSS4 | -1.38 (1.514) | -0.05 (0.752) | -0.20 (1.785) | -0.20 (0.615) | 0.10 (0.167) |
| GLSS5 | -1.86* (1.104) | 0.08 (0.556) | 0.30 (1.110) | -0.13 (0.447) | -0.17 (0.112) |
| GLSS6 | -0.94* (0.527) | -0.06 (0.257) | 0.79 (0.512) | -0.11 (0.209) | -0.32*** (0.059) |
| GSPS1 | -1.37* (0.779) | -0.09 (0.401) | 0.68 (0.771) | -0.14 (0.323) | -0.39*** (0.090) |
| GSPS2 | -0.06 (0.362) | 0.01 (0.176) | -0.12 (0.408) | -0.01 (0.144) | 0.07 (0.057) |
| **Uncertainty function** |  |  |  |  |  |
| Mean | -1.15*** (0.148) | -0.62*** (0.112) | -0.90*** (0.145) | -0.69*** (0.083) | -3.76*** (0.078) |
| Variance | 0.56 (0.042) | 0.73 (0.041) | 0.64 (0.046) | 0.71 (0.029) | 0.15 (0.006) |

Significance levels: * p<0.10, ** p<0.05, ***p<0.01; Data Sources: Ghana Living Standards Surveys [wave 1-7] and Ghana Socioeconomic Panel Survey [wave 1-2]

#### **S7 Table**. Determinants of Starchy Staple Technical Inefficiency/ Technology Gap in Ghana (1987-2017)

|  | Female  (dummy) | Age (years) | | Education (years) | | Land owned  (dummy) | Extension  (dummy) | Credit  (dummy) | Mechanization  (dummy) |
| --- | --- | --- | --- | --- | --- | --- | --- | --- | --- |
|  |  | Level | Squared | Level | Squared |  |  |  |  |
| Cassava |  |  |  |  |  |  |  |  |  |
| Guinea Savanah | 0.49* (0.263) | 0.53* (0.315) | -0.78 (0.892) | 0.13 (0.244) | 0.01 (0.028) | -0.58*** (0.206) | 0.26 (0.215) | 0.79*** (0.236) | 0.32 (0.451) |
| Transitional Zone | 0.37*** (0.116) | 0.12 (0.184) | 1.05*** (0.371) | 0.10 (0.129) | 0.01 (0.015) | 0.03 (0.114) | -0.26* (0.135) | 0.41*** (0.151) | - |
| Forest Zone | 0.16** (0.077) | 0.24* (0.125) | 0.39 (0.313) | 0.05 (0.073) | 0.00 (0.008) | -0.12* (0.069) | -0.10 (0.082) | -0.02 (0.106) | 0.37 (0.329) |
| Coastal Savanah | 0.35** (0.159) | 0.08 (0.223) | 0.05 (0.507) | -0.08 (0.196) | -0.01 (0.023) | 0.21 (0.157) | - | 0.41* (0.213) | 0.01 (0.431) |
| National | 0.28*** (0.054) | 0.28*** (0.085) | 0.39* (0.206) | 0.09 (0.055) | 0.01 (0.006) | -0.11** (0.050) | -0.06 (0.060) | 0.19*** (0.069) | 0.12 (0.164) |
| Meta-frontier | 0.11 (0.070) | 0.03 (0.088) | -0.12 (0.205) | 0.01 (0.062) | 0.00 (0.007) | -0.02 (0.058) | -0.14 (0.088) | 0.15** (0.066) | 0.47*** (0.135) |
| Yam |  |  |  |  |  |  |  |  |  |
| Sudan Savanah | -1.96* (1.138) | -0.05 (0.559) | 1.28 (1.284) | 1.48* (0.852) | 0.18* (0.103) | -2.20** (1.118) | -0.38 (1.012) | -0.19 (0.412) | -1.92** (0.899) |
| Guinea Savanah | 0.33* (0.196) | -0.14 (0.161) | 0.22 (0.305) | 0.15 (0.136) | 0.02 (0.016) | -0.16 (0.133) | 0.03 (0.117) | 0.19 (0.130) | -0.79** (0.401) |
| Transitional Zone | 0.36** (0.152) | 0.30 (0.195) | 0.74* (0.440) | 0.54*** (0.152) | 0.06*** (0.018) | 0.07 (0.132) | -0.20 (0.156) | 0.40** (0.173) | -0.46 (0.550) |
| Forest Zone | 0.01 (0.166) | 0.14 (0.254) | -0.09 (0.690) | -0.11 (0.154) | -0.02 (0.018) | 0.02 (0.163) | 0.26 (0.200) | -0.15 (0.178) | -0.96* (0.495) |
| National | 0.41*** (0.077) | 0.38*** (0.098) | 0.24 (0.223) | 0.22*** (0.071) | 0.02** (0.008) | -0.02 (0.069) | -0.15** (0.076) | 0.17** (0.073) | -0.77*** (0.202) |
| Meta-frontier | 0.47*** (0.086) | 0.64*** (0.126) | -0.02 (0.281) | 0.04 (0.077) | -0.01 (0.009) | -0.05 (0.087) | -0.29*** (0.100) | 0.16* (0.088) | -0.81*** (0.215) |
| Cocoyam |  |  |  |  |  |  |  |  |  |
| Transitional Zone | 0.45* (0.246) | -0.69 (0.428) | -0.11 (1.029) | -0.17 (0.221) | -0.02 (0.025) | 0.92*** (0.337) | 0.37 (0.379) | 0.57* (0.327) | -49.53 (0.000) |
| Forest Zone | 0.09 (0.123) | 0.33* (0.198) | 0.36 (0.412) | 0.13 (0.110) | 0.01 (0.013) | -0.17 (0.109) | 0.07 (0.119) | 0.06 (0.133) | -0.39 (0.449) |
| National | 0.27** (0.131) | 0.25 (0.200) | 0.52 (0.422) | 0.09 (0.113) | 0.01 (0.013) | 0.00 (0.118) | 0.06 (0.127) | 0.12 (0.143) | -0.62 (0.660) |
| Meta-frontier | 0.08 (0.141) | -0.14 (0.252) | 0.63 (0.505) | 0.25 (0.169) | 0.03 (0.019) | -0.16 (0.124) | -0.19 (0.141) | 0.02 (0.161) | -0.55 (0.835) |
| Plantain |  |  |  |  |  |  |  |  |  |
| Transitional Zone | -0.02 (0.161) | 0.29 (0.244) | 0.48 (0.576) | 0.05 (0.159) | 0.01 (0.018) | -0.24* (0.137) | -0.36* (0.183) | -0.09 (0.195) | -3.61 (8.208) |
| Forest Zone | 0.00 (0.077) | 0.20* (0.118) | 0.49* (0.294) | 0.04 (0.079) | 0.00 (0.009) | -0.23*** (0.080) | -0.13 (0.089) | -0.02 (0.094) | - |
| Coastal Savanah | 0.04 (0.283) | 0.06 (0.363) | 1.18 (1.079) | 0.37 (0.270) | 0.04 (0.032) | 0.01 (0.249) | -0.21 (0.363) | -0.47 (0.396) | 0.54 (1.051) |
| National | -0.02 (0.066) | 0.20** (0.099) | 0.39 (0.239) | 0.06 (0.065) | 0.01 (0.007) | -0.22*** (0.063) | -0.14* (0.075) | -0.06 (0.078) | -0.39 (0.335) |
| Meta-frontier | -0.09 (0.085) | 0.07 (0.126) | -0.32 (0.280) | 0.02 (0.091) | 0.00 (0.011) | -0.06 (0.081) | -0.37*** (0.140) | -0.20* (0.101) | 1.02*** (0.394) |

Significance levels: * p<0.10, ** p<0.05, ***p<0.01

Data Sources: Ghana Living Standards Surveys [wave 1-7] and Ghana Socioeconomic Panel Survey [wave 1-2

#### **S8 Table.** Ghanaian Starchy Staple Production Technology Level and Technical Efficiency Parameters Across Seasons (1987-2017)

| Season |  | TGR | TE | MTE | GAP |  | TGR | TE | MTE | GAP |
| --- | --- | --- | --- | --- | --- | --- | --- | --- | --- | --- |
|  |  | Cassava | | | |  | Yam | | | |
| 1987/88 |  | 0.717 | 0.455 | 0.324 | 28.30% |  | 0.663 | 0.401 | 0.268 | 33.70% |
| 1988/89 |  | 0.998 | 0.490 | 0.493 | 0.16% |  | 0.678 | 0.572 | 0.364 | 32.16% |
| 1990/91 |  | 1.000 | 0.833 | 0.836 | 0.03% |  | 0.530 | 0.466 | 0.255 | 47.02% |
| 1991/92 |  | 1.000 | 0.850 | 0.852 | 0.03% |  | 0.642 | 0.450 | 0.295 | 35.81% |
| 1997/98 |  | 0.969 | 0.700 | 0.682 | 3.13% |  | 0.381 | 0.812 | 0.216 | 61.89% |
| 1998/99 |  | 0.967 | 0.690 | 0.669 | 3.29% |  | 0.622 | 0.661 | 0.365 | 37.84% |
| 2004/05 |  | 0.981 | 0.744 | 0.728 | 1.94% |  | 0.566 | 0.562 | 0.311 | 43.40% |
| 2005/06 |  | 0.982 | 0.748 | 0.736 | 1.84% |  | 0.588 | 0.568 | 0.324 | 41.19% |
| 2009/10 |  | 0.702 | 0.751 | 0.518 | 29.84% |  | 0.837 | 0.535 | 0.449 | 16.34% |
| 2011/12 |  | 0.738 | 0.645 | 0.457 | 26.24% |  | 0.594 | 0.512 | 0.298 | 40.60% |
| 2012/13 |  | 0.757 | 0.645 | 0.476 | 24.30% |  | 0.580 | 0.489 | 0.267 | 42.01% |
| 2014/15 |  | 0.832 | 0.592 | 0.488 | 16.84% |  | 0.406 | 0.427 | 0.187 | 59.37% |
| 2015/16 |  | 0.876 | 0.443 | 0.389 | 12.38% |  | 1.000 | 0.496 | 0.484 | 0.00% |
| 2016/17 |  | 0.879 | 0.459 | 0.404 | 12.08% |  | 1.000 | 0.550 | 0.526 | 0.00% |
|  |  |  |  |  |  |  |  |  |  |  |
| Season |  | Cocoyam | | | |  | Plantain | | | |
| 1987/88 |  | 0.981 | 0.567 | 0.561 | 1.90% |  | 0.719 | 0.410 | 0.298 | 28.08% |
| 1988/89 |  | - | 0.534 | - | - |  | 0.534 | 0.404 | 0.209 | 46.56% |
| 1990/91 |  | 0.653 | 0.494 | 0.316 | 34.75% |  | 0.859 | 0.704 | 0.604 | 14.07% |
| 1991/92 |  | 0.576 | 0.643 | 0.301 | 42.44% |  | 0.837 | 0.674 | 0.563 | 16.28% |
| 1997/98 |  | 0.784 | 0.590 | 0.467 | 21.63% |  | 0.854 | 0.662 | 0.558 | 14.63% |
| 1998/99 |  | 0.817 | 0.603 | 0.502 | 18.30% |  | 0.839 | 0.675 | 0.564 | 16.05% |
| 2004/05 |  | 0.782 | 0.415 | 0.336 | 21.79% |  | 0.890 | 0.659 | 0.584 | 11.03% |
| 2005/06 |  | 0.794 | 0.410 | 0.318 | 20.57% |  | 0.905 | 0.624 | 0.561 | 9.48% |
| 2009/10 |  | 1.000 | 0.564 | 0.557 | 0.00% |  | 0.995 | 0.724 | 0.721 | 0.50% |
| 2011/12 |  | 0.665 | 0.437 | 0.294 | 33.55% |  | 1.000 | 0.587 | 0.578 | 0.00% |
| 2012/13 |  | 0.676 | 0.419 | 0.286 | 32.38% |  | 1.000 | 0.576 | 0.571 | 0.00% |
| 2014/15 |  | 0.822 | 0.556 | 0.448 | 17.81% |  | 0.767 | 0.544 | 0.413 | 23.29% |
| 2015/16 |  | 0.749 | 0.095 | 0.065 | 25.08% |  | 0.831 | 0.439 | 0.356 | 16.89% |
| 2016/17 |  | 0.828 | 0.271 | 0.221 | 17.23% |  | 0.852 | 0.452 | 0.382 | 14.76% |
|  |  |  |  |  |  |  |  |  |  |  |


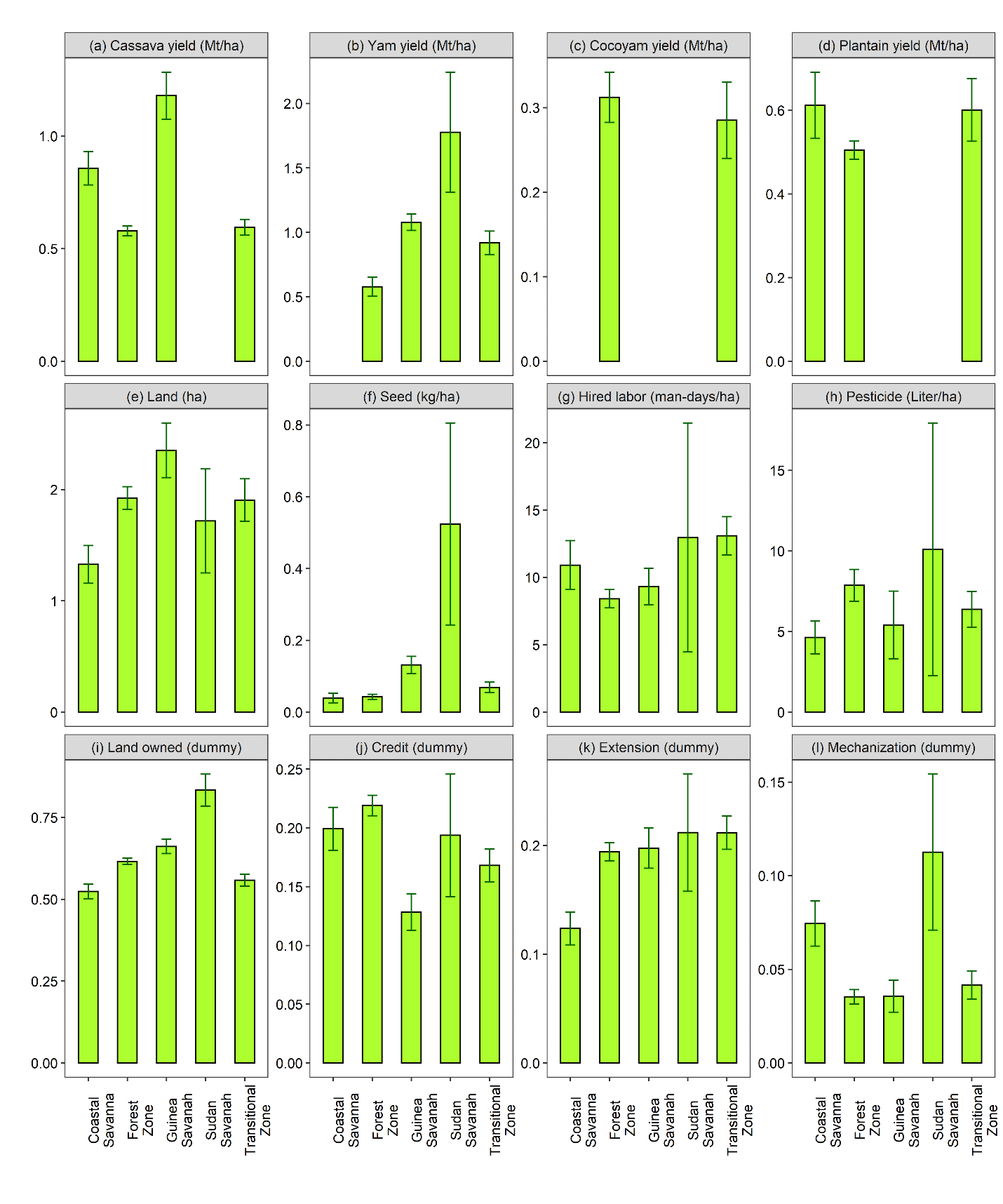


#### **S1a** F**igure.** Ecological variation in Starchy Staple production yield, input, and enabling environment factors in Ghana (1987-2017)


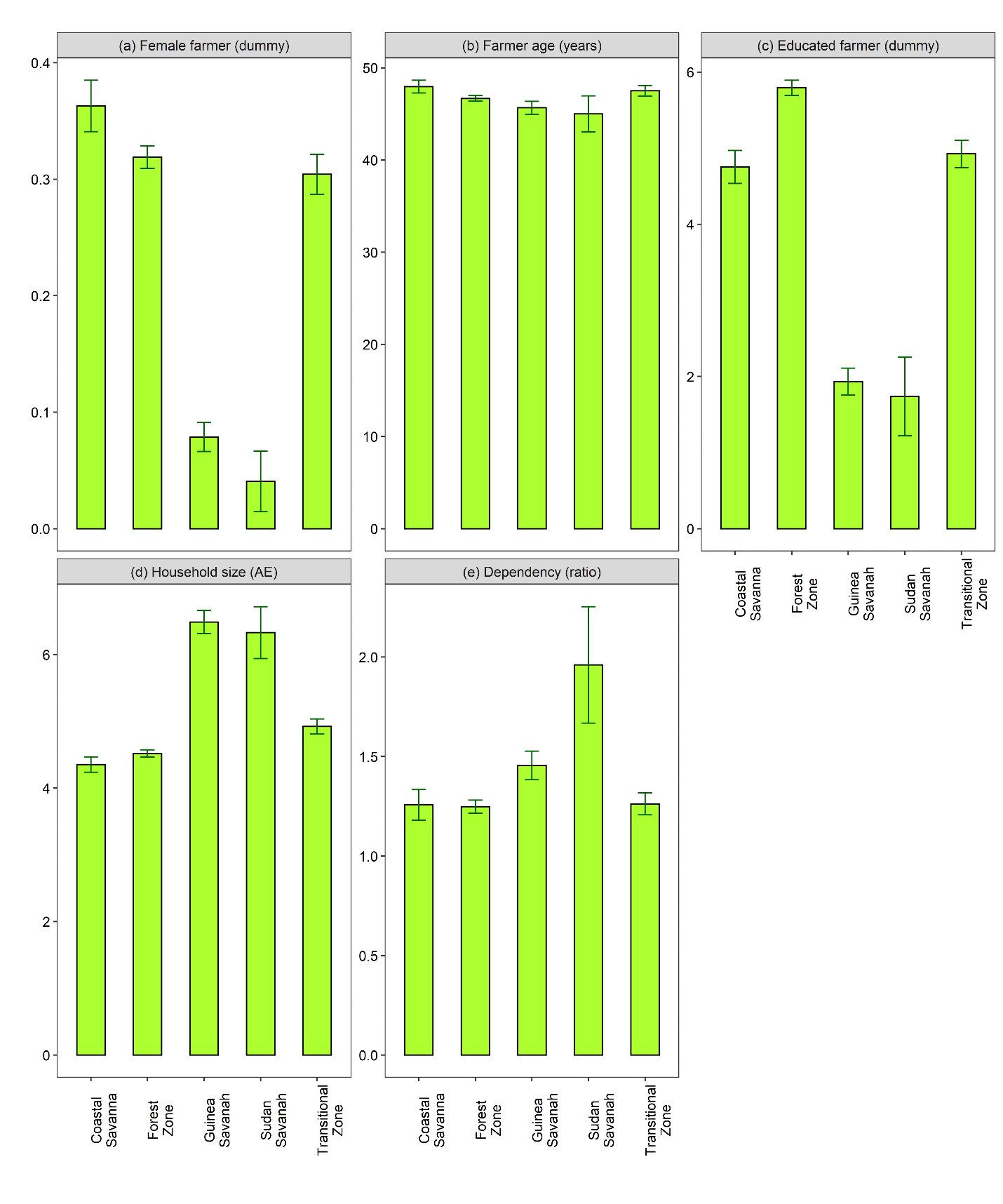


#### **S1b Figure.** Ecological variation in Starchy Staple Farmer and Household Demographics factors in Ghana (1987-2017)

#### **S1 Note. Construction of variables**

Female (dummy): Across all surveys, this variable takes on a value of unity if the actual farmer is female, zero otherwise.

Age (years): Across all surveys, this variable was taken as the recorded age of the farmer whose production information was analyzed.

Education (years): Across all surveys, this variable was taken as the recorded years of education of the farmer whose production information was analyzed.

Land owned (dummy): Across all surveys, this variable takes on a value of unity if the actual farmer owned portions of the land under cultivation.

Land (ha): Across all surveys, this variable is taken as the farmer recalled land under production, converted to hectares.

Yield (kg/ha): Across all surveys, this value was taken as the quantity of harvest divided by the land under cultivation of a given starchy staple.

Family labor (AE): Across all surveys, family labor was calculated as the total adult equivalence (AE) attributable to members aged 15 years and older.

Hired labor (man-days): For GSPS, input usage was reported at the plot level, thus the quantity of hired labor was taken as the farmer recalled quantity of paid labor in man-days, multiplied by the ratio of production value to total production value for a given plot. These were then summed for a given farmer and starchy staple. Information on inputs in all seven GLSSs were collected at the household as expenditures. Consequently, for each farmer, the expenditure for hired labor was calculated as the respective share of that farmer's crop production value in total household crop harvest value; subsequently, these were divided by the annual minimum agricultural daily wage to approximate the man-days of hired labor used.

Pesticide (Liter): Same as hired labor, the only exception is that, where necessary, the annual price for fertilizer (GHC/kg) was used to convert pesticide expenditures to liters.

Household Size (AE): Across all surveys, household size in adult male equivalence (AE) was computed by dividing the total energy requirements of the household by 2,250 kcal.

Dependency (ratio): Given the AE of its members, the dependency ratio of the household was then calculated as the total AE attributable to members aged between 17 and 70 years divided by that for those aged below 18 years or above 69 years.

Credit (dummy): Across all surveys, this variable takes on a value of unity if at least one household member applied for a loan or received inputs on credit, zero if not, and two if no credit information was collected in the survey. A value of two was assigned to data drawn from the GLSS1, GLSS2, GLSS3, and GLSS4 surveys since they were never collected. In this case, the coefficient on category one is taken as the credit effect in any econometric estimation.

The extension (dummy): Across all surveys, this variable takes on a value of unity if the actual farmer is in an enumeration area (as defined by the survey) in which at least one farmer reported having received extension services from MoFA or NGOs. Also, where community information was collected, this variable takes on a value of unity if there was an extension agent or office in that community. This variable takes on a value of zero if the above conditions are not met.

#### **S2** **Note.** Test for Model Specification

***Skewness test:*** The Coelli [1] and Schmidt and Lin [2] skewness tests for OLS residuals are negative for all the models, suggesting that the production variation of starchy staples across Ghana’s ecologies is negatively skewed. The Gutierrez [3] test confirmed the conclusions of the Coelli [1] and Schmidt and Lin [2] tests. The outcome of these tests indicates that the negative skewed error needed for the justification of SFA is strong. Furthermore, the null hypotheses $H_{o}:\boldsymbol{\alpha}=0$ was also rejected, thus, providing further justification for the technical inefficiency functions.

***The similarity of production frontiers across ecologies:*** To statistically check the similarities of the production frontiers for a given starchy staple across the different ecologies, the study applied a generalized likelihood ratio test. The log-likelihood values under the null hypothesis of a uniform technology were all smaller than that of the alternate hypothesis of heterogeneous technology. The differences in the log-likelihood values led to test statistics that soundly rejected the null hypothesis, and thus, support the fact that starchy staple farmers are operating under heterogeneous technologies along ecological lines.

***Production variance:*** The proportion of production variance due to inefficiency ($\gamma$) ranged from 0.20 to 0.99 for the ecology frontiers; for a given crop, this was always higher than that of the Meta-frontier. The $\gamma$ values suggest that a considerable amount of the observed variation in output for the ecology-frontiers [Meta-frontier] could be attributed to inefficient use of inputs [technological gaps]. The range of $\gamma$ for the Meta-frontier also suggests that the observed variation in output, given the ecology frontiers, could not be attributed to idiosyncrasies. Finally, the SFA model chi-squared test statistics indicate that the models are all significant. In Table C1, we report the summary of estimates for the ecology- and meta-frontier production elasticities for the starchy staples and discuss the details in the following subsections along crop-specific lines.

**Appendix S References**

1. Coelli T. Estimators and hypothesis tests for a stochastic frontier function: A Monte Carlo analysis. J Product Anal. 1995;6: 247–268. doi:10.1007/BF01076978

2. Schmidt P, Lin T-F. Simple tests of alternative specifications in stochastic frontier models. J Econom. 1984;24: 349–361. doi:10.1016/0304-4076(84)90058-7

3. Gutierrez RG, Carter S, Drukker DM. On boundary-value likelihood-ratio tests. Stata Tech Bull. 2001;10. Available: https://econpapers.repec.org/article/tsjstbull/y_3a2001_3av_3a10_3ai_3a60_3asg160.htm
