# Supplementary material for: Starchy staples production shortfalls in Ghana: Technical inefficiency effects outweigh technological differences across ecologies
Source: PLoS One. 2023 Apr 24;18(4):e0284780. doi: 10.1371/journal.pone.0284780 (PMC10124864; doi:10.1371/journal.pone.0284780)
Supplement: Attachment — Submitted filename: PONE-D-22-23407_reviewer_Completed.pdf [file pone.0284780.s003.pdf]

PLOS ONE

Starchy staples production shortfalls in Ghana: Technical inefficiency effects outweigh technological differences across ecologies

--Manuscript Draft--

**Manuscript Number:**	PONE-D-22-23407
**Article Type:**	Research Article
**Full Title:**	Starchy staples production shortfalls in Ghana: Technical inefficiency effects outweigh technological differences across ecologies
**Short Title:**	Starchy staples production shortfalls in Ghana
**Corresponding Author:**	Isaac Gershon Kodwo Ansah, Ph.D<br>University for Development Studies<br>Tamale, Northern Region GHANA
**Keywords:**	starchy staples; technological gap ratio; technical inefficiency; Ghana
**Abstract:**	Staple crop production in developing Africa provides both food and income to participating households, hence closing the yield gaps is crucial to improving food security and nutrition. This paper assesses the production shortfalls of major starchy staple crops in Ghana using a meta-frontier efficiency analysis. Results show strong evidence of about 50% production shortfall for cassava, yam, cocoyam, and plantain. The production shortfall is further shown to be more influenced by pure farmer technical inefficiencies (45%) rather than by technology gaps (12%) along ecological lines. Thus, the sector could benefit from improvements in farmer managerial skills and efficient use of existing technologies.
**Order of Authors:**	Isaac Gershon Kodwo Ansah, Ph.D
	Francis Tsiboe, Ph.D
	Mark Appiah-Twumasi, MD
**Additional Information:**	
**Question**	**Response**
**Financial Disclosure**<br><br>Enter a financial disclosure statement that describes the sources of funding for the work included in this submission. Review the submission guidelines for detailed requirements. View published research articles from *PLOS ONE* for specific examples.<br><br>This statement is required for submission and **will appear in the published article** if the submission is accepted. Please make sure it is accurate.	The authors received no specific funding for this work.

Powered by Editorial Manager® and ProduXion Manager® from Aries Systems Corporation

**Unfunded studies**<br>Enter: *The author(s) received no specific funding for this work.*<br><br>**Funded studies**<br>Enter a statement with the following details:<br>• Initials of the authors who received each award<br>• Grant numbers awarded to each author<br>• The full name of each funder<br>• URL of each funder website<br>• Did the sponsors or funders play any role in the study design, data collection and analysis, decision to publish, or preparation of the manuscript?<br>• **NO** - Include this sentence at the end of your statement: *The funders had no role in study design, data collection and analysis, decision to publish, or preparation of the manuscript.*<br>• **YES** - Specify the role(s) played.<br><br>* typeset	
**Competing Interests**<br><br>Use the instructions below to enter a competing interest statement for this submission. On behalf of all authors, disclose any competing interests that could be perceived to bias this work—acknowledging all financial support and any other relevant financial or non-financial competing interests.<br><br>This statement is required for submission and **will appear in the published article** if the submission is accepted. Please make sure it is accurate and that any funding sources listed in your Funding Information later in the submission form are also declared in your Financial Disclosure statement.<br><br>View published research articles from *PLOS ONE* for specific examples.	The authors have declared that no competing interests exist.

Powered by Editorial Manager® and ProduXion Manager® from Aries Systems Corporation

**NO authors have competing interests**<br><br>Enter: *The authors have declared that no competing interests exist.*<br><br>**Authors with competing interests**<br><br>Enter competing interest details beginning with this statement:<br><br>*I have read the journal's policy and the authors of this manuscript have the following competing interests: [insert competing interests here]*<br><br>* typeset	
**Ethics Statement**<br><br>Enter an ethics statement for this submission. This statement is required if the study involved:<br><br>• Human participants<br>• Human specimens or tissue<br>• Vertebrate animals or cephalopods<br>• Vertebrate embryos or tissues<br>• Field research<br><br>Write "N/A" if the submission does not require an ethics statement.<br><br>General guidance is provided below. Consult the submission guidelines for detailed instructions. **Make sure that all information entered here is included in the Methods section of the manuscript.**	N/A

**Format for specific study types**<br><br>**Human Subject Research (involving human participants and/or tissue)**<br>• Give the name of the institutional review board or ethics committee that approved the study<br>• Include the approval number and/or a statement indicating approval of this research<br>• Indicate the form of consent obtained (written/oral) or the reason that consent was not obtained (e.g. the data were analyzed anonymously)<br><br>**Animal Research (involving vertebrate animals, embryos or tissues)**<br>• Provide the name of the Institutional Animal Care and Use Committee (IACUC) or other relevant ethics board that reviewed the study protocol, and indicate whether they approved this research or granted a formal waiver of ethical approval<br>• Include an approval number if one was obtained<br>• If the study involved *non-human primates*, add *additional details* about animal welfare and steps taken to ameliorate suffering<br>• If anesthesia, euthanasia, or any kind of animal sacrifice is part of the study, include briefly which substances and/or methods were applied<br><br>**Field Research**<br><br>Include the following details if this study involves the collection of plant, animal, or other materials from a natural setting:<br>• Field permit number<br>• Name of the institution or relevant body that granted permission	
**Data Availability**<br><br>Authors are required to make all data underlying the findings described fully available, without restriction, and from the time of publication. PLOS allows rare exceptions to address legal and ethical concerns. See the PLOS Data Policy and FAQ for detailed information.	No - some restrictions will apply

Powered by Editorial Manager® and ProduXion Manager® from Aries Systems Corporation

A Data Availability Statement describing where the data can be found is required at submission. Your answers to this question constitute the Data Availability Statement and **will be published in the article**, if accepted.<br><br>**Important:** Stating 'data available on request from the author' is not sufficient. If your data are only available upon request, select 'No' for the first question and explain your exceptional situation in the text box.<br><br>Do the authors confirm that all data underlying the findings described in their manuscript are fully available without restriction?	
**Describe where the data may be found in full sentences. If you are copying our sample text, replace any instances of XXX with the appropriate details.**<br><br>• If the data are **held or will be held in a public repository**, include URLs, accession numbers or DOIs. If this information will only be available after acceptance, indicate this by ticking the box below. For example: *All XXX files are available from the XXX database (accession number(s) XXX, XXX.*).<br>• If the data are all contained **within the manuscript and/or Supporting Information files**, enter the following: *All relevant data are within the manuscript and its Supporting Information files.*<br>• If neither of these applies but you are able to provide **details of access elsewhere**, with or without limitations, please do so. For example:<br><br>*Data cannot be shared publicly because of [XXX]. Data are available from the XXX Institutional Data Access / Ethics Committee (contact via XXX) for researchers who meet the criteria for access to confidential data.*<br><br>*The data underlying the results presented in the study are available from (include the name of the third party*	The data underlying the results presented in the study are available from Ghana Statistical Service and can be accessed freely online via https://www.statsghana.gov.gh/gssdatadownloadspage.php.

and contact information or URL).<br>• This text is appropriate if the data are owned by a third party and authors do not have permission to share the data.<br><br>* typeset	
Additional data availability information:	Tick here if the URLs/accession numbers/DOIs will be available only after acceptance of the manuscript for publication so that we can ensure their inclusion before publication.

Powered by Editorial Manager® and ProduXion Manager® from Aries Systems Corporation

Manuscript

Click here to access/download;Manuscript;v03_Roots_TE_GH_MT_FT - PloS

Starchy staples production shortfalls in Ghana: Technical inefficiency effects outweigh technological differences across ecologies

Isaac Gershon Kodwo Ansah [a*], Francis Tsiboe[b], Mark Appiah-Twumasi[c]

[a] Department of Economics, School of Applied Economics and Management Sciences, University for Development Studies, Tamale, Ghana.

[b] USDA, Economic Research Service, MS9999, Beacon Facility, P. O. Box 419205, Kansas City, MO 64141, USA

[a] Department of Agricultural and Food Economics, Faculty of Agriculture, Food and Consumer Sciences, University for Development Studies, Tamale, Ghana.

* Corresponding author at: Department of Economics, School of Applied Economics and Management Sciences, University for Development Studies, Tamale, Ghana. E-mail address: agershon@uds.edu.gh (Isaac Gershon Kodwo Ansah) (ORCID ID: 0000-0001-5071-6224)

Data Availability: Replication materials are available in GitHub at https://github.com/ftsiboe/Agricultural-Productivity-in-Ghana

Funding : The authors did not receive any funding for this manuscript.

Abstract

Staple crop production in developing Africa provides both food and income to participating households, hence closing the yield gaps is crucial to improving food security and nutrition. This paper assesses the production shortfalls of major starchy staple crops in Ghana using a meta-frontier efficiency analysis. Results show strong evidence of about 50% production shortfall for cassava, yam, cocoyam, and plantain. The production shortfall is further shown to be more influenced by pure farmer technical inefficiencies (45%) rather than by technology gaps (12%) along ecological lines. Thus, the sector could benefit from improvements in farmer managerial skills and efficient use of existing technologies.

Keywords: starchy staples; technological gap ratio; technical inefficiency; Ghana

JEL codes: D24, Q18, Q12.

Starchy staples production shortfalls in Ghana: Technical inefficiency effects outweigh technological differences across ecologies

1. Introduction

In 2018 the Food and Agriculture Organization's document on the State of Food and Nutrition Security in the world reported that the number of undernourished people globally has worsened, rising from 804 million people in 2016 to 821 million, an equivalence of one out of every nine persons in the world (FAO et al., 2018). In explaining the possible causes of these rising food insecurity challenges, the report identifies poor access to food, among other factors, with Africa and Asia being the main hosts of these challenges. Since the primary source of food in these areas, Africa especially, is domestic production, this, coupled with lessons from border closures due to COVID-19, creates the urgent need to examine the pathways through which food is made available and accessible locally to the vulnerable segment of the population.

In many parts of the developing world, smallholder farmers mostly organize the production of staples. Several FAO reports on "The State of Food Security in the World" point to low agricultural productivity as characterizing smallholder production systems. Productivity growth and resource use efficiency are important causal pathways to increased food production and access for the poor in these areas. Besides direct food availability, agro-industrial development rests so much on the availability of raw materials to supply industrial needs. How could the industrial need be met if what is available cannot satisfy direct consumption demand? Therefore, productivity growth in these crops is crucial to foster rapid economic transformation in the developing world.

The production of cassava (*Manihot Esculenta*), cocoyam (*Colocasia Esculenta*), plantain (*Musa Sapientum*) and yam (*Dioscorea Alata*) (henceforth called starchy staples) plays important roles

in the Ghanaian economy. Starchy staples are a major source of livelihood support for farmers, traders, and processors who participate in these crops' value chains, while also providing staple food to many people, especially the less affluent in society. Despite this position, the productivity figures of starchy staples are low. There are non-ignorable productivity gaps between actual and potential yields. In 2018 the national average yield of cassava, cocoyam, plantain, and yam were all over 50% lower than their potential yields (Ministry of Food and Agriculture [MoFA], 2019).[1]

These yield gaps tend to vary widely across regions and ecological zones in Ghana. Such variations indicate that the yield gaps might be location-specific, crop-specific, and farmer-specific. The large yield gaps generate costs to the country in terms of wasted resources amid scarcity as well as creating environmental challenges. These productivity gaps, therefore, border on the efficiency of resource utilization which would require attention. Resource use efficiency, in the layman's understanding, means *doing more with less*. A transition from doing less with more (inefficiency of resource utilization) to doing more with less (efficiency of resource utilization) allows the creation of more economic value with less natural, human, and man-made resources, and eventually enhances household welfare. But how can this be achieved if we have limited knowledge of what drives these production shortfalls, or their nature and distribution thereof?

Several studies relevant to Ghanaian agriculture have investigated the primary drivers of resource use efficiency for rice (A. Abdulai and Huffman, 2000; Anang, Bäckman, and Sipiläinen, 2016; Asravor et al., 2019; Donkor, Matthews, and Ogundeji, 2018; Owusu, Donkor, and Owusu-

[1] Potential yield are the expected yields of crops published by Ghana's Ministry of Food and Agriculture. They are usually obtained through field experiments by agronomists/crop scientists where all the technical conditions needed for optimum performance of crops are provided. In 2019, these were estimated at 45Mt/ha, 38Mt/ha, 52Mt/ha, and 20Mt/ha for Cassava, Plantain, Yam, and Cocoyam respectively (MoFA, 2019).

Sekyere, 2018), maize (S. Abdulai, Nkegbe, and Donkoh, 2013; Owusu, 2016; Wongnaa and Awunyo-Vitor, 2019), legumes (Avea et al., 2016; Awunyo-vitor et al., 2013; Etwire, Martey, and Dogbe, 2013), cocoa (Besseah and Kim, 2014; Danso-Abbeam and Baiyegunhi, 2019; Tsiboe, 2021), and vegetables (Tsiboe, Asravor, and Osei, 2019). However, to the best of knowledge, similar studies for starchy staples are scanty and limited in scope. Particularly, the few studies like Owusu Asante, Villano, and Battese (2014) that considered starchy staples analyzed data for only one season, and they did not consider the possibility of heterogeneous technology adoption within Ghana. Like their peers cultivating vegetables (Tsiboe et al., 2019), rice (Asravor et al., 2019), and maize (Owusu, 2016), starchy staples farmers could be operating under different technologies constrained by the ecology and/or enabling environment. According to the literature, failure to account for these technological differences could lead to falsely attributing production shortfalls due to technology gaps to technical inefficiency (Battese, Rao, and O'Donnell, 2004). This paper extends the analysis by accommodating multiple seasons and heterogeneity in ecological technology adoption.

Particularly, this study applies Huang, Huang, and Liu (2014)'s method of estimating meta-stochastic-frontier to a sample of 15,420 starchy staple farmers drawn from nine cross-sectional population-based surveys periodically fielded throughout Ghana from 1987 to 2017. The aim is to examine whether production shortfalls are primarily driven by resource use efficiency among farmers or due to technological gaps across ecological zones in Ghana, as well as their nature and distribution thereof. The pooled data has the broadest coverage of starchy staple households across time and space than previous studies conducted on Ghana. As such, it presents a unique opportunity for utilizing the meta-stochastic frontier to ascertain the ecological and temporal dynamics of starchy staple resource use efficiency in Ghana. This is important because the

achievement of Sustainable Development Goals 2.1 (ensuring access to food for all) and 2.2 (ending all forms of malnutrition) would, at least partly, require the realization of Sustainable Development Goal 12, which leverages the efficient use of natural resources to create a circular economic growth (World Bank, 2018).

2. Productivity and Efficiency Literature

Globally, the methods for productivity and efficiency analyses have witnessed a clear evolution since the seminal work of Farrell (1957). From this evolution, agricultural productive efficiency has generally been grouped into technical or allocative, with the two combining to form economic efficiency. Technical Efficiency (TE), the focus of this study, is the ability of a (farm) firm to maximize output based on an optimal set of inputs. TE can be measured from an output orientation, where observed output is compared to the potential output given the input set. TE can also be measured from an input orientation where observed input levels are compared to the minimum input required to produce a given output level (Belotti et al., 2013). Both orientations can be implemented using either Stochastic Frontier Analysis (SFA) (Aigner, Lovell, and Schmidt, 1977; Meeusen and van Den Broeck, 1977) or Data Envelopment Analysis (DEA) (Charnes, Cooper, and Rhodes, 1978, 1981).

The two major differences between DEA and SFA are that, unlike the latter, the former does not impose a functional form on the data and assumes that output variability is deterministic as it is solely due to inefficiency. By not accounting for idiosyncratic shocks, the implied production frontier of DEA is susceptible to outliers and measurement error and has no statistical properties for inferences. Nonetheless, there has been significant progress made on dealing with the limitations of DEA regarding statistical properties and testing via bootstrapping. For the SFA, its main limitation has been the imposition of a functional form on the production process. The

distribution for the inefficiency term also sometimes comes with a few challenges. However, the SFA accommodates idiosyncratic shocks and therefore makes the model less susceptible to outliers and measurement errors. With a well-specified functional form, SFA allows the researcher to undertake statistical inferences. In the Ghanaian context, while both SFA and DEA have been applied in empirical studies, SFA dominates mainly due to the unavailability of reliable input price data that is often required by DEA. In this study, reliable price data is also not available, hence we utilize the output oriented SFA approach.

In the literature, SFA is the more popular choice for estimating the efficiency of smallholder agricultural production systems. In Africa, this method has been applied mainly for studies on cereals and legumes. Recently, Morse (2021) and Ojo and Ayanwale (2019) have examined the efficiency of starchy staples production using the SFA. A meta-analysis by Morse (2021) on the technical efficiency of yam production in Nigeria revealed an average technical efficiency between 0.6 and 0.8. The study further recommended enhancing farmers' knowledge on input application, instead of intensifying application, as the major policy initiative for reducing technical inefficiency among farmers in Nigeria. On the other hand, Ojo and Ayanwale (2019) attempted to determine the credit amount needed to bridge the inefficiency gap in plantain production by combining a Cobb-Douglas SFA, the Harrod-Domar growth equation, and Heckman selection model to control selectivity bias in credit access. They reported a mean TE of 0.53 and a financing gap of US$263 per growing season.

Cassava production is one of Africa's most significant crop enterprises, contributing significantly to food security and supply of raw materials for agro-industrial use in Africa and her trading partners. Nigeria is the leading global producer of cassava followed by Thailand and Brazil, but the top 10 cassava producers are exclusively made of countries in the global south (Ebewore and

Isiorhovoja, 2019). Hence, cassava has received the most attention of the four starchy staples in the production efficiency literature. Studies on cassava production tend to prefer other methods to the SFA in estimating its efficiency. Ogundari and Brümmer (2011) justify their choice of a primal output distance function on the high level of intercropping in cassava fields in Nigeria. Rahman and Awerije (2015) opted for a two-stage DEA in their study because of its ability to estimate efficiency at the production and processing stages while Fu, Qu, and Pan (2018) opted for an output-oriented DEA. These studies commonly admit that cassava yields have stagnated lately due to the discontinuity of government policy by succeeding administrations and the saturated adoption of technologies by farmers.

In Ghana, most of the literature focused on applications of the SFA to cross-sectional samples of cereals, legumes, and cocoa farmers. The samples for these studies have largely been limited to districts or at best a region and for one production season. Notably, Abdulai and Huffman (2000) examined how the Structural Adjustment Programme influenced the economic efficiency of rice production in northern Ghana based on a cross-section of 120 northern rice farmers for the 1992/1993 growing season. Using the Translog SFA and ordinary least squares (OLS) regression, the authors reported profit losses of 27% due to inefficiency in domestic rice production. Owusu Asante et al. (2014) examined how the adoption of improved yam production technology affected farmers' TE in Ghana's Transitional zone. Using 375 farmers from the Ashanti and Brong-Ahafo regions, the authors used a Translog SFA to model the efficiency of yam mini-sett technology adoption. The results showed that adopters in the Ashanti region were more efficient than those in Brong Ahafo. Anang et al. (2016) used Cobb-Douglas SFA to model rice production technical inefficiency in the Upper East and Northern regions of Ghana. Based on a sample of 300 rice farmers that cultivated in the 2013/2014 season, TE was estimated at 62%.

Extensions of the SFA accommodated the possibility that Decision Making Units (DMUs) do not operate with identical technologies. Differences in technology could be due to resource availability, government regulation, input prices, and the environment. From the literature, failure to account for technological differences in each DMU's circumstance could lead to falsely attributing technological gap-related production shortfalls to inefficient input use (Battese et al., 2004). Consequently, since Hayami (1969) introduced the Meta-Stochastic-frontier (MSF) approach and further developments (Hayami and Ruttan, 1970, 1971), it has been used to capture technological differences among DMUs.

The literature on MSF has grown by incorporating the possibility of technological heterogeneity in the analysis. However, except for a few studies (Tsiboe, 2020; Tsiboe, Aseete, and Djokoto, 2021; Tsiboe et al., 2019), the Ghanaian literature is limited to districts/regions for one production season. For instance, Owusu et al. (2018) assessed the gender technology gap among smallholder rice farmers by estimating a Cobb-Douglas MSF function to generate TE, Meta-Technical Efficiency (MTE), and Technology Gap Ratio (TGR) for male and female farmers. The TE estimates showed that male farmers were marginally more efficient because of higher seeding and fertilizer application rates, while the MTE and TGR showed that female-managed farms were closer to the MSF than that of their male counterparts. However, by incorporating data from multiple seasons, a recent study also using MSF showed that from 1987-2017, the overall crop production gender gap in Ghana has reduced (Adaku, Tsiboe, and Clottey, 2022); thus, highlighting the merits of incorporating data from multiple seasons.

Owusu (2016) investigated the impact of improved maize variety adoption on TE and productivity from three agroecological zones using a Translog SFA combined with a matching estimator to correct for selectivity bias. The results showed that adopters in the Semi-Deciduous Forest Zone

were between 25% to 36% more efficient than non-adopters, while in the Guinea Savannah Zone adopters were between 15% to 26% more efficient than non-adopters. In the Transitional Zone, adoption was found to diminish farmers' TE by 6-8%, which led to productivity losses of 12-19%. The author attributed differences in TE and productivity effects to differences in levels of adoption of complementary inputs among farmers in the various agroecological zones. A similar study also reported TGRs and MTE differences for rice farms in the Transitional and Guinea Savannah Zones of Ghana (Asravor et al., 2019).

One of the few studies (Tsiboe et al., 2019), that looked at production throughout Ghana over growing seasons between 1987-2017 and along ecological lines showed that TEs for okra, pepper, and tomato were 0.54, 0.74, and 0.58 respectively. Like this study, Tsiboe et al. (2019) used harmonized data from seven cross-sectional population-based surveys fielded throughout Ghana between 1987-2017 and were able to show that TE has generally increased for okra and pepper but remained constant for tomato. Furthermore, Tsiboe et al. (2019) showed that ecological gaps are very small for pepper, modest for tomato, and high for okra.

In the last two decades, research on productivity and efficiency analyses in Ghana is highly skewed towards cereal crops. In principle, the starchy staples have received little attention, against the backdrop of the critical roles these crops play, and the significant yield gaps observed in practice. Except for a few studies (Adaku et al., 2022; Tsiboe, 2021; Tsiboe et al., 2021, 2019), all efficiency studies in Ghana used data based on a single season, and data size is limited to a few villages or at best a region. This data limitation and lack of locational heterogeneity masks important lessons that could emerge from analysing a more comprehensive and heterogeneous sample. For example, knowledge on whether technology gaps across ecologies or resource use inefficiencies among farmers contribute most to production shortfalls and how these evolve is not yet clear. Such

incomplete information might limit scientific progress, and serve as a barrier to societal development, in the sense that policymakers may be inadequately or wrongly informed. Focusing on starchy staples, this study addresses most of these limitations.

3. Research area and data

3.1 Study Area

Ghana covers a total land area of 24 million hectares of which 20.7% is considered arable, 40.7% under forests, 11.8% under permanent crops, and 26.8% for other uses. As shown in Figure 1, Ghana's ecology is classified as Rain Forest (RFEZ), Semi-Deciduous Forest (SFEZ), Coastal Savanna (CSEZ), Transitional Zone (TEZ), Guinea Savanna (GSEZ), and Sudan Savanna (SSEZ). The aridity of these ecologies increases from south to north. Farming systems across these ecologies are highly heterogeneous. However, the highest yielding ecologies across all the starchy staples are SFEZ and TEZ, and the low-yielding ones are SSEZ and RFEZ. Given their well-balanced annual rainfall and modest temperatures, SFEZ and TEZ have the optimal conditions to grow starchy staples. Thus, it is not surprising that they also contain most of the cultivated lands allocated to starchy staples as shown in Tables S1 and S2 (all tables, figures, and notes with a leading S indicates supplemental materials in the online appendix)

3.2 Household Level Data Sources

The study uses data drawn from two sources: (1) the two waves of the Ghana Socioeconomic Panel Survey (GSPS) fielded in 2009/10 and 2014/15 (Institute of Statistical Social and Economic Research (ISSER) and Economic Growth Center (EGC), 2016); and (2) all seven Ghana Living Standards Surveys (GLSS) fielded between 1987-2017 and publicly available at the Ghana Statistical Service (GSS) National Data Archive (NADA). The surveys were based on nationally representative samples, focusing on the household as the key socio-economic unit, and provides

insights into Ghana's living conditions. The GSPS was collected by the Economic Growth Center (EGC) at Yale University and the Institute of Statistical, Social, and Economic Research (ISSER), and the GLSS by GSS. In terms of the raw data collection, both surveys followed a two-stage stratified sampling design, where enumeration areas and households were selected in the first and second stages, respectively. The harmonization details of these datasets to construct farmer-level data are published in Tsiboe (2020). It is worth noting that while the GSPS constitutes a panel, households are resampled for each GLSS round, hence not a panel. Consequently, since a large proportion of the data is from the GLSS, the harmonized data by Tsiboe (2020) can be viewed as a cross-sectional sample of the population of Ghanaian starchy staple farmers at roughly five-year intervals.

The final sample used in this study was limited to starchy staple farmers drawn from the various surveys, with yield (kg/ha) above the 5th and below the 90th percentile by survey, crop, and region. The final sample, therefore, consisted of 15,402 farmers from 15,130 households, out of which 11,584, 3,468, 1,834, and 6,223 farmers cultivate cassava, yam, cocoyam, and plantain, respectively. All of Ghana's ecologies are well represented in the final sample, and the dataset captures 14 growing seasons. Nonetheless, due to sparse data, the RFEZ and SFEZ are combined into a Forest Ecology (FEZ). Note S1 documents the definition of all the variables used in this study.

3.3 Descriptive statistics

Table 1 provides the descriptive statistics of the household level characteristics, showing their temporal, ecological, and crop variation. Except for land and yield, average values were estimated across all ecologies and by crop. In assessing the differences in these variables across ecology and starchy staples, we used linear regression for continuous variables and probit model for dummies.

A trend variable, and a fixed effect for ecology and crop, as well as their interactions, were included in the estimations. Plots of differences in the household level variables across ecologies are further elaborated in Figure S1 (*a and b*).

From Table 1, an average household contains five members in adult equivalence (AE) units, consistent with national estimations of 4.4 and 5.1 in the 2010 and 2000 censuses respectively (Ghana Statistical Service [GSS], 2012). The dependency ratio, with an average rate of 1.30, measures the extent of pressure on an earning individual in the household. Averagely, the starchy staple farmer in Ghana is a 47-year-old male with five years of formal education and a 61% chance of owning his main farm plot. Regarding starchy staples, we found no significant variation between males and females (1=female), education, and land ownership; but in terms of ecological zones, we found significant variation in these variables. Age, on the other hand, showed no significant ecological or crop variation. In terms of their temporal dynamics from 1987 to 2017, while the probability of observing a female starchy staple farmer significantly declined by 0.17% annually, that of age and years of formal education increased by 0.47% and 1.58% annually, respectively.

In terms of production scale, the value of mean land area confirms the widely held notion that agriculture in Ghana is operated on a smallholder basis. Generally, yam (1.57 ha) has received the largest plot allocation for the period 1987-2017 while cassava (1.17 ha) received the lowest. Starchy staple farm sizes are statistically different across ecologies and have declined over the period (1987-2017). Given these scales of production, yields were estimated at 0.66, 0.96, 0.31, and 0.53 Mt/ha for cassava, yam, cocoyam, and plantain, respectively. Except for cocoyam, these yields also vary along ecological lines. Nonetheless, except for yam, they all show an annual upward trend from 1987 to 2017. Figure S1 (*a and b*) provides further details on how the household level variables differ across ecologies and crops.

In terms of input utilization, planting material usage rate was estimated at 0.06 Mt/ha, with the SSEZ having the highest and CSEZ having the lowest. On average, three household members provided labor for starchy staple production, and this has significantly increased by about 0.45% annually since 1987. Hired labor is used at a rate of about 11 man-days/ha, which is equivalent to an annual increase of about 1.24% since 1987. The highest usage of hired labor is for farms in the SSEZ, followed by TEZ, CSEZ, GSEZ, and FEZ, in that order. Farmers in the sample used about 8 liters of pesticides per hectare. Analyzing pesticide use across ecological zones and time shows consistently low usage in the CSEZ relative to the SSEZ and FEZ for all crops.

Access to credit and extension (measured as dummies) depict factors that reflect the enabling environment for production success. Access to credit and extension services are reported in the literature to have diverse effects on technical inefficiency. The rate of access to credit or extension is estimated at 19%; however, credit access is higher in FEZ and CSEZ than in the GSEZ and SSEZ. Furthermore, Table 1 shows that the rate of access has significantly declined annually by 0.19% for credit but increased for extension by 0.75% from 1987 to 2017.

4. Methodology

4. Theoretical framework

The prototypical output oriented SFA approach assumes that given their input set, DMUs utilizing a homogeneous technology will put them at various points along the Stochastic Frontier (SF). However, due to technical inefficiency and/or idiosyncratic shocks (i.e., downside production risk), one may observe some DMUs below the SF. Furthermore, some DMUs may also be observed above the SF solely due to upside production risk. The SFA is formulated as:

$$y_i = f(x_i)e^{\varepsilon_i}, \qquad \varepsilon_i = v_i - u_i \tag{1}$$

where, y_i is the output level when x_i inputs are used with the technology $f(x_i)$. The deviations from the SF due to technical inefficiency and production risk are represented by u_i and v_i, respectively. Consequently, the assumptions made on the distribution of the deviations (v_i and u_i) underpin the estimation of Equation (1). Generally, v_i is assumed to follow a normal distribution with zero mean and variance $\sigma_{v_i}^2$ [$v_i \sim N(0, \sigma_v^2)$]. However, due to its negative skewness, u_i is assumed to follow either half-normal, exponential, truncated, or gamma distributions (Belotti et al., 2013). It is important to mention that the distribution of u_i affects the estimates of TE (Van Nguyen et al., 2021).

In the context of SFA, the technical inefficiency component (u_i) has been shown to be influenced by exogenous variables other than inputs, and its variance has been redefined as $\sigma_{u_i}^2 = \exp(\boldsymbol{w}_i\boldsymbol{\alpha})$; where $\boldsymbol{w}_i$ and $\boldsymbol{\alpha}$ are the vectors of explanatory variables and parameters (Caudill, Ford, and Gropper, 1995), respectively. Under these modifications, rejecting $H_o\!:\boldsymbol{\alpha} = 0$ provides justification that exogenous factors explain inefficiency (Aigner et al., 1977). The TE of the *i*th DMU is calculated as:

$$TE_i = E[\exp(-u_i)\,|\hat{\varepsilon}_i] \qquad (2)$$

Assuming that DMUs can be grouped into technological groups, Equation (1) can be estimated as a Meta-Stochastic-frontier (MSF) following Huang et al. (2014)'s two-step-method. Equation (1) is initially estimated separately for each sub-group (*j*), and then the predicted output levels from the sub-group frontiers are used as the observation for MSF (pooled stochastic frontiers) in the second step. The conventional one-sided error term (u_i^M) for the MSF serves as the estimate for any technology gaps among the different sub-groups. The MSF which envelops all sub-group-specific stochastic frontiers [$f^j(x_i)$] is specified in Equation (3) as

$$f^j(x_i) = f^M(x_i)e^{-u_i^M}, \qquad u_i^M \sim N^+\big(0, \exp(\boldsymbol{w}_i\boldsymbol{\alpha})\big) \tag{3}$$

where u_i^M is strictly positive, implying that $f^j(x_i) \leq f^M(x_i)$. Consequently, the ratio of group j's stochastic frontier to the MSF is the TGR, represented as

$$TGR_i = \frac{f^j(x_i)}{f^M(x_i)} = e^{-u_i^M} \leq 1 \tag{4}$$

The TGR depends on the accessibility and adoption level of the available MSF which in turn depends on DMU-specific circumstances. The MTE represents each DMU's technical efficiency with respect to the meta frontier production technology. MTE can be decomposed into the TE (which is the efficiency measured with respect to the zone-specific frontier) and the TGR (which is the difference between the best available technology and the technology set adopted). Accordingly, each DMU's MTE is given by equation (5) as follows:

$$MTE_i = f^j(x_i)[f^M(x_i)e^{v_i}]^{-1} = TGR_i \times TE_i \tag{5}$$

4.1 Empirical model specification

Previous studies have shown that Ghanaian crop production at the micro-level is best captured by the Cobb-Douglas (Anang et al., 2016; Owusu et al., 2018), or Translog (Asravor et al., 2019; Tsiboe, 2021) production function. However, due to its relative flexibility, this study implements the MSF assuming that $f_t^j(\cdot)$ is Translog. Nonetheless, since Cobb-Douglas is nested within the Translog, the former was tested after estimation and was soundly rejected at p<0.01 for all models (see Table 2). Thus, the Translog specification is appropriate for the data, and is represented as

$$\ln y_{ijt} = \beta_{0r} + \textstyle\sum_k \beta_{kj} \ln x_{kijt} + \frac{1}{2}\sum_s \sum_k \beta_{skj} log \ln x_{kijt} \ln x_{sijt} + v_{ijt} - u_{ijt}$$

$$u_{ijt} \sim N^+\big[0, \exp\big(\boldsymbol{w}_{ijt}\boldsymbol{\alpha}\big)\big], \qquad v_{ijt} \sim N(0, \sigma_v^2) \tag{6}$$

where, y_{jit} is total production (Mt) for the ith farmer in ecology j at time t. Each x_{kjit} represents the kth input (i.e., land, planting material, family and hired labor, and pesticides) used by the ith farmer and a trend variable. As Van Nguyen et al. (2021) report, outcomes from the SFA, especially with efficiency estimates, are severely affected by the distribution assumptions. Therefore, we considered all possible distributions but eventually had to settle on a half-normal distribution (i.e., $u_{ijt} \sim N^{+}\left[0, \exp\left(\boldsymbol{w}_{ijt}\boldsymbol{\alpha}\right)\right]$), since the truncated normal and exponential distributions presented issues of convergence. Following Tsiboe et al. (2019), $\boldsymbol{w}_{ijt}$ contained farmer characteristics (age, education, and gender), institutional factors (land ownership, credit, and extension), a trend and constant term.

The parameters of the ecology- and meta-frontiers were estimated via maximum likelihood, using the "*frontier*" command in Stata 15. Furthermore, estimation variables were standardized by their sample means by survey and crop. Given the parameters, input elasticities were estimated as the first derivative of the frontiers with respect to that input, evaluated at their means. The production returns to scale (RTS) were then estimated as the sum of the input elasticities. The standard errors for the elasticities were estimated via the delta method. Ecology-specific technical TE, TGR, and MTE were estimated using equations (2), (4), and (5), respectively. Estimates were summarized by ecology and season.

5. Results and Discussions

The core objective of this paper is to understand whether production shortfalls existing among the starchy staples in Ghana are driven by technological gap, inefficiency, or both. Thus, the discussion in this section seeks to accomplish that purpose. In the interest of space, we only present the diagnostic test of the empirical model, estimates of elasticities, and estimates of TE, TGR, and

MTE in the main text in Tables 2, 3, and 4, respectively. The paper presents the estimates of the production functions (Tables S3-S6) and drivers of technical inefficiency (Table S7) in the online appendix. Discussions on the drivers of technical inefficiency and technology gaps are also presented in Note S3 in the online appendix. Trends of TE, TGR, and MTE are also presented in Figure 2 and Table S8.

The diagnostics on the negative skewed error needed for the justification of SFA shown in Table 2 confirmed the appropriateness of the empirical model. Further, test results on the similarity of production frontiers across ecologies support the fact that starchy staple farmers are operating under heterogeneous technologies in the various ecologies. This justifies further inquiry into whether technology gaps across ecologies or resource use inefficiencies among farmers contribute most to production shortfalls. The major learning points include the wide range of the estimated γ values which suggest that considerable amounts of the observed crop output variation for the ecology-frontiers [meta-frontier] could be attributed to inefficient use of inputs [technological gaps]. The range of γ for the meta-frontier also suggests that the observed variation in output, given the ecology-frontiers, could not be attributed to idiosyncrasies. Also, the SFA model chi-squared test statistics indicate that the models are all significant. Specific discussion of relevant diagnostics is presented in Note S2.

5.1 Elasticities and Meta-Frontier Results of Ghanaian Starchy Staples Production

Based on production elasticities for cassava in Table 3, land is the most operationally significant input contributing the highest to output across all ecological zones. The contribution of family labor, hired labor, planting material, and pesticide use follow in that order across all ecological zones. The returns to scale (RTS) estimates for all ecologies and the meta-frontier confirm decreasing returns to scale (DRS).

Like cassava, land contributes the highest returns to yam production in all ecological zones. Yam is a very dominant cash crop enterprise. Due to the laborious nature of the enterprise, farmers often employ hired labor as much as they rely on family labor. The labor activities in yam production are often specialized (e.g., making mounds) and this limits issues of moral hazard from hired labor. Hence, it is not too surprising that family labor (for SSEZ and FEZ), as well as hired labor (GSEZ and TEZ), have the second-highest significant elasticities. Besides land and labor, the contributions of pesticides and planting materials to yam output are either not significant or negative. Estimated returns to scale follow the pattern observed for cassava; increasing all yam production inputs proportionately would yield a less than proportionate increase in yam output. The SSEZ reported the highest productivity estimate, implying that yam farmers in the SSEZ are closest to the meta-frontier technology. On the contrary, yam farmers in the FEZ are the farthest from the meta-frontier.

For cocoyam, land makes the highest contribution to output in the FEZ but is marginally outstripped by family labor in the TEZ. Along with land, hired labor makes consistently positive contributions to cocoyam output in both ecological zones; planting materials significantly reduce output while pesticides make no significant contributions. Cocoyam returns to scale follows that of cassava and yam. Furthermore, cocoyam farmers in the FEZ operate closer to the meta-frontier than their TEZ counterparts.

Again, land has the highest output elasticity across all ecological zones for plantain and is followed by family labor and hired labor. Planting materials show significant effects in FEZ (negative) and CSEZ (positive) while pesticide is only significant in the CSEZ (negative). Like the other starchy staples, plantain production exhibits DRS in all ecological zones and nationally. Plantain farmers in the TEZ are closer to the meta-frontier, while those in the CSEZ are the farthest.

Next, we analyze the trends and distributions of technological gaps and technical efficiencies based on crops in the various ecologies and highlight the implications of these findings. The discussions here are principally based on results in Table 4 and Figure 2. Corresponding values to Figure 2 are reported in Table S8. These results show the temporal and spatial dynamics of starchy staple-specific technology level and technical efficiency.

5.2 Cassava Production

In the data, four main ecological zones were significantly involved in cassava production, which are the GSEZ, TEZ, FEZ, and CSEZ. The TGR ranges from zero to one: values closer to one indicate the use of technology like the meta-frontier technology. In other words, the technology gap is the difference between the best technology available in the production of a particular crop and the technology set adopted by a farmer. The choice of the adopted technology is determined by environmental and socio-economic factors. The mean TGR for cassava, estimated at 0.91, indicates that the technological gap averaged over all ecological zones is 9%. Figure 2, panel a1 shows that the TGR for cassava increased from 0.76 in 1987 to 1.00 in 1998 and dropped to an average of 0.85 between 2004 and 2012. Afterward, it increased until reaching a peak of 1.00 in 2014 before then settling at 0.90 in 2017. Given the high mean TGR across the sample, it is not surprising that the ecology specific TGRs are all above 80% with the highest in GSEZ (0.94), reflecting a superior technology for that ecology. The TGR for GSEZ is followed by that of TEZ (0.93), FEZ (0.90), and then CSEZ (0.88). The TGR for cassava is relatively high when compared to 0.75 for tomato (Tsiboe et al., 2019) and 0.74 for rice (Asravor et al., 2019).

Ecology-specific TE for cassava is estimated at a mean of 0.63 across the entire sample. Like the TGR, the TE for cassava increased by about 72% from 0.46 in 1987 to a peak of 0.79 in 1991 and has been declining at about 4.47% annually to 0.45 in 2017 (Figure 2, panel b1). Again, the mean

TE for cassava is the highest in GSEZ (0.64) and followed by FEZ/CSEZ (0.64) and then TEZ (0.58). An important caveat about these TE scores is that they do not rank the farms based on the cassava meta frontier, so we discuss the MTE results next.

After accounting for ecology-specific production technologies, the mean MTE across the entire sample is 0.57. Specifically, the most technically efficient cassava farmers relative to the metafrontier are those in the GSEZ with an MTE of 0.61. Table 4 shows that the GSEZ farmers are followed by their peers in FEZ (0.57), CSEZ (0.56), and then TEZ (0.55). Figure 2, panel c1 shows that the MTE increased from 0.34 in 1987 to 0.80 in 1991 due to improvements in technology adoption. However, from 1991 to 2017, MTE declined at the same rate as pure farmer technical efficiency to 0.41. Generally, the interest of policies is to introduce new technologies while less attention is given to mechanisms that can increase the efficiency of production. For instance, improved crop varieties often require complementary inputs (enabling environments such as credit to purchase fertilizer, among others) to produce optimal yields. However, such conditions often are not the focus of research (P. P. Acheampong, Owusu, and Nurah, 2018) or government policies after introducing improved seeds to farmers. Overall, the high TGR and high resource wastage (farmers in the most efficient ecological zone can reduce input use by 36% and achieve the same output) show that cassava MTE could be enhanced by improving TE.

5.3 Yam Production

Yam is significantly cultivated in four main ecological zones, which are the SSEZ, GSEZ, TEZ, and FEZ. Mean TGR for yam, estimated at 0.93, is higher than that of cassava. Thus, on average the ecological technology gap for yam is only about 7%. Figure 2, panel a2 shows that the TGR for yam has remained relatively constant at an average of 0.93 over the study period. At the ecology

level, the highest TGR is estimated for SSEZ (0.98). Table 4 shows that SSEZ is followed by GSEZ (0.98), TEZ (0.93), and then FEZ (0.84).

Ecology-specific TE for yam is estimated at a mean of 0.55 and has remained relatively constant over the study period (Figure 2, panel b2). Yam TE follows similar patterns as the TGR, the only difference being that FEZ (0.51) is higher than TEZ (0.49) and the highest found in SSEZ (0.64) as reported in Table 4. The TE values for yam estimated in this study are relatively low when compared to values of 0.85 and 0.89 estimated by Owusu Asante et al. (2014) for Ashanti and Brong Ahafo regions (all approximately located in the TEZ), respectively. Several things distinguish this study from Owusu Asante et al. (2014) that could account for the differences in TE. First, our study contains production data from multiple regions/ecologies throughout Ghana and multiple production seasons, while Owusu Asante et al. (2014) used data from only 375 yam farmers from only one ecology and for only one season. Thus, the values estimated by Owusu Asante et al. (2014) do not holistically capture the spatiotemporal dynamics of yam TE as this study does. Secondly, Owusu Asante et al. (2014) utilized data that is over-represented by those farmers (58%) using yam mini-sett technology which the authors show induces positive and significant effects on the TE of some of its adopters. By the nature of its data, this study is not able to pinpoint the specific technologies used by each farmer. However, farmers in this study sample use several technologies which may include yam mini-sett technology.[2]

Accounting for ecology-specific production technologies shows that the mean yam MTE is 0.51. Like the TGR and TE, the MTE for yam is also constant across the study period but improves from

[2] TE scores are relative measures that track efficiency relative to the most efficient observations within a particular sample. Thus, as a caveat any difference between this study's estimates and previous works could simply be reflecting differences in heterogeneity across samples.

Southern to Northern Ghana. Specifically, the most technically efficient yam farmers relative to the industrial production frontier are those in the SSEZ with an MTE of 0.66. Table 4 shows that the SSEZ farmers are followed by their peers in GSEZ (0.52), TEZ (0.46), and then FEZ (0.41). Like cassava, the overall results for yam also show that yam MTE could be enhanced by improving TE.

These findings of inefficient production outcomes for yam are worrying for several reasons. Ghana is the world's second-largest producer of yam (behind Nigeria) but the leading exporter to the global market (P. Acheampong et al., 2019; Ansah and Tetteh, 2016). Given that about 94% of yam produced in Ghana is destined for the international market, eliminating the causes of production shortfalls has the potential to improve the livelihoods of actors in the yam value chain. At the local levels, yam holds keystone positions in the cultural expression of several tribes in Ghana (Odotei, 2002; Ogundeji and Fasehun, 2021) and remains an essential food security crop. For instance, yam consumption per capita stood at 125kg in 2015 (P. Acheampong et al., 2019); however, about 40% of yam harvested in the GSEZ is lost through poor postharvest handling (Ansah and Tetteh, 2016). This, along with resource use inefficiencies, threatens the capacity of yam to contribute to Ghana attaining the targets of SDG 2.

5.4 Cocoyam Production

Cocoyam production dominates only in two agroecological zones (TEZ and FEZ). This is hardly surprising since these areas are also famous for their high cocoa production. Traditionally, cocoyam was grown as a complement to provide shade for young cocoa seedlings (P. Acheampong et al., 2014). Even though cocoyam production has since expanded beyond this basic use, its proliferation in the country has been largely restricted to specific areas in southern Ghana. Coupled with the fact that the cocoyam was originally introduced in the forest belt, the dominance of

cocoyam production in the TEZ and FEZ has been mainly attributed to the wide vegetation cover that the forest belt provides as well as the well-distributed rainfall amounts (Aidoo et al., 2019). This assertion is given credence by the fact that the FEZ (Semi-Deciduous and Rain Forests) and TEZ recorded the highest average rainfall estimates of all ecological zones in this study (Table S1).

Mean TGR for cocoyam is lower than the estimates for cassava and yam. The estimated cocoyam TGR of 0.82 indicates that the ecological technology gap is about 18%. Figure 2, panel a3 shows that the TGR for cocoyam has remained at a higher value of 0.80 over the study period. This reflects the same dynamics in the yam TGR but at a lower magnitude. At the ecology level, the TGR for cocoyam is higher in the FEZ (0.84) than in the TEZ (0.80).

As indicated, TGR is dependent on the accessibility and adoption level of frontier-enhancing technologies. For cocoyam production in Ghana, P. P. Acheampong, Owusu, and Nurah (2018) examined the rates of adopting improved technologies, the determinants of adoption, and the benefits of adopting cocoyam technologies to farmers. The results of their analysis partly resonate with ours because they drew their sample from similar ecological zones. Among others, P. P. Acheampong, Owusu, and Nurah (2018) found that only about 22% of farmers adopted improved planting material varieties although more than 60% were aware of their availability. Similarly, low rates of adoption were realized for agronomic practices such as the application of manure, inorganic fertilizer, cover cropping, minimum tillage, and row planting. However, there were high adoption levels for techniques such as appropriate planting distance, timely weed control, and weedicide application. With regards to the determinants of adoption, they found that experienced farmers and farmers whose farm plots were far removed from their homestead had lower probabilities of adopting all technologies. The opposite was observed for farmers that belonged to

farmers' associations primarily because they were more aware of the benefits of new technologies through higher social integration. The study reported significant gender and ecological variations although unlike in this study, they found higher adoption among farmers in the TEZ than those in the FEZ.

Ecology-specific TE for yam is estimated at a mean of 0.52 across the entire sample. Unlike the TGR, the cocoyam TE has been declining at a rate of about 5.2% from 1987 to 2017 (Figure 2, panel b3). Contrary to the TGR, cocoyam TE is higher in TEZ (0.57) than in the FEZ (0.48) as depicted in Table 4.

The dynamics in TGR and TE culminate in an estimated mean MTE of 0.41 for cocoyam. This follows the temporal trend exhibited by TE but improves from Southern to Northern Ghana. Unlike cassava and yam, the overall results for cocoyam show that production could be enhanced by improving both TGR and TE. The estimated RTS parameter for cocoyam (0.45) is the lowest for all the starchy staples considered in this study. Just like for cassava and yam, the implications are that extensification of production (for example, through area expansion) is not a feasible entry point to change the status quo.

5.5 Plantain Production

Like yam and cocoyam, plantain has traditionally been integrated as a complementary crop to cocoa. Its broad leaves and succulent stems provide ecosystems services to young cocoa seedlings. Hence, our finding that plantain cultivation takes place in three ecological zones, namely, the TEZ, FEZ, and CSEZ is to be expected as these zones describe the "cocoa belt" of Ghana. Apart from providing shade for cocoa seedlings, smallholder farm households in Ghana secure their food by integrating plantain into tree crop plantations to offset the income gap between plantation establishment and commercial yields.

Plantain is integral to the food security and poverty alleviation efforts of Ghana. Its utilization in the diets of the producing areas is varied and has been increasing in recent times (MoFA, 2019). Results from Table 3 show that land has the highest output elasticity across all ecological zones and is followed by family labor, hired labor, pesticide, and then planting materials. Like the other starchy staples, plantain production exhibits DRS in all ecological zones and nationally. The estimated RTS of 0.63 implies that a 1% increase in all inputs will yield about 0.63% increase in plantain output. Practically, this means the cost-saving or profit returns of increasing all the factors of plantain production have been attained, given the present technology.

Mean TGR for plantain (0.91) is lower than that of cassava and yam but higher than cocoyam. This indicates a plantain technology gap of about 9%. Figure 2, panel a4 shows that the TGR for plantain has increased approximately 3% annually from 0.73 in 1987 to 0.86 in 2017. At the ecology level, plantain TGR decreases from TEZ (0.94), FEZ (0.87) to CSEZ (0.76). Thus, plantain farmers in the TEZ are closest to the meta-frontier, while those in the CSEZ can increase their productivity by about 24% if they adopt the best available technology.

Ecology-specific TE for plantain is estimated at a mean of 0.54 across the entire sample. Like TGR, TE for plantain has been increasing at a rate of about 3% from 1987 to 2017 (Figure 2, panel b4). The ecological zone ranking (high to low) of plantain TE is from CSEZ (0.68), FEZ (0.58), to TEZ (0.50). In terms of MTE, the mean for plantain is 0.50 and it follows the temporal trend exhibited by TE and TGR. Specifically, the MTE ranking (high to low) for plantain is from FEZ (0.52) and CSEZ (0.51) to TEZ (0.47). Like cocoyam, plantain production could be enhanced by improving both TGR and TE. The low MTE estimates for plantain production in this study are indicative of what has been reported in the literature. For instance, MoFA (2019) indicated that

actual average plantain yields represent only 32% of what is achievable; even the best performing district attains less than 50% of the estimated potential yield.

6. Conclusions and Recommendations

Improvements in crop productivity by closing production gaps in rural economies are generally touted as an important pathway to achieving three main developmental goals, namely food security, reduced poverty, and sustainable resource utilization. Yet, many farmers unwittingly waste significant productive resources through production and technological inefficiencies which engender poverty and food insecurity. Changing the status quo and fostering rapid economic transformation would require sustained improvements in agricultural productivity. In this study, we take advantage of a large Ghanaian dataset that spans over three decades and examine the spatiotemporal trends of production gaps of one of the most important staple crop categories, the starchy staples. Unlike similar studies thus far, and in addition to the unique dataset used, the method adopted accounts for technological heterogeneity across the main ecological zones in Ghana where these starchy staple crops are cultivated. Using the stochastic meta-frontier approach, technical efficiency, meta-technical efficiency, and technology gap ratios are measured across crops and ecological zones. The main aim of doing this analysis is to provide a broad understanding of what the starchy staples industry looks like, the major growth points, and the limitations that need critical policy attention.

The key findings suggest that there are significant shortfalls in starchy staples production among agroecological zones, which could be more attributed to technical efficiencies and less to technology gaps. Stunningly, the results show that the current set of production technologies used by starchy staple farmers in Ghana across ecologies are quite similar as there exists an ecological technology gap of less than 13% across all four crops. On the other hand, there exist significant

production shortfalls that could be more attributed to technical inefficiencies. Respectively, about 37, 45, 48, and 46% of cassava, yam, cocoyam, and plantain production is lost due solely to technical inefficiencies and these have been relatively constant or at worst increased from 1987 to 2017. These dynamics suggest that over time, limited attention might have been given to interventions that inure to improve the efficiency of resource use among starchy staple farmers. With the large production shortfalls attributable largely to technical inefficiency, one implication of our results is that the starchy staples crop sector could benefit from improvements in conditions that enhance farmer technical efficiencies, such as providing starchy staple farmers more access to extension services. Research in Ghana's cocoa sector shows that increasing farmer access to technology improving inputs such as fertilizer is only one part of the solution to improve production; training and other human capital investments pertaining to proper input usage are also needed (Opoko et al., 2009; Tsiboe et al., 2016).

Further, the results also show that for any given starchy staple crop some ecological zones perform better than others, thereby indicating heterogeneity in both technology and production management practices. Thus, the sector could benefit from the documentation of lessons from the frontier enhancing technologies and practices in high-performing ecological zones, which can then be improved further and disseminated to less performing agro-ecologies, guided of course by contextual factors.

References

Abdulai, A., and W. Huffman. 'Structural Adjustment and Economic Efficiency of Rice Farmers in Northern Ghana'. *Economic Development and Cultural Change* 48,3(April 2000):503–520.

Abdulai, S., P. K. Nkegbe, and S. a Donkoh. 'Technical efficiency of maize production in Northern Ghana'. *African Journal of Agricultural Reseearch* 8,43(2013):5251–5259.

Acheampong, P., J. Osei-Adu, N. E. Amengor, and R. Sagoe. 'Cocoyam value chain and

benchmark study in Ghana.' 2014.

Acheampong, P., E. Owusu Danquah, H. Dissanayake, P. Hayford, and C. Weebadde. 'A Socioeconomic Study of Transition Zone Yam Farmers Addressing Constraints and Exploring Opportunities for Integrating Pigeonpea into Yam Cropping Systems'. *Sustainability* 11,3(January 2019):717.

Acheampong, P. P., V. Owusu, and G. Nurah. 'How does Farmer Preference matter in Crop variety Adoption? The case of Improved Cassava varieties' Adoption in Ghana'. *Open Agriculture* 3,1(November 2018):466–477.

Adaku, A. A., F. Tsiboe, and J. Clottey. 'Taking Stock of Gender Gaps in Crop Production Technology Adoption and Technical Efficiency in Ghana'. 2022.

Aidoo, R., J. K. A. I, F. D. Wireko-Manu, and A. Wangel. 'Root and Tubers in Ghana Overview and selected Research Papers'. 2019.

Aigner, D., C. A. K. Lovell, and P. Schmidt. 'Formulation and estimation of stochastic frontier production function models'. *Journal of Econometrics* 6,1(July 1977):21–37.

Anang, B. T., S. Bäckman, and T. Sipiläinen. 'Technical efficiency and its determinants in smallholder rice production in northern Ghana'. *The Journal of Developing Areas* 50,2(2016):311–328.

Ansah, I. G. K., and B. K. D. Tetteh. 'Determinants of Yam Postharvest Management in the Zabzugu District of Northern Ghana'. *Advances in Agriculture* 2016(2016):1–9.

Asravor, J., A. Wiredu, K. Siddig, and E. Onumah. 'Evaluating the Environmental-Technology Gaps of Rice Farms in Distinct Agro-Ecological Zones of Ghana'. *Sustainability* 11,7(April 2019):2072.

Avea, A., J. Zhu, X. Tian, T. Baležentis, T. Li, M. Rickaille, and W. Funsani. 'Do NGOs and Development Agencies Contribute to Sustainability of Smallholder Soybean Farmers in Northern Ghana—A Stochastic Production Frontier Approach'. *Sustainability* 8,5(May 2016):465.

Awunyo-vitor, D., J. a Bakang, S. K. Gyan, and S. Cofie. 'Estimation of Farm Level Technical Efficiency of Small-Scale Cowpea Production in Ghana'. *American-Eurasian Journal of Agricultural and Environmental Science* 13,8(2013):1080–1087.

Battese, G. E., D. S. P. Rao, and C. J. O'Donnell. 'A Metafrontier Production Function for Estimation of Technical Efficiencies and Technology Gaps for Firms Operating Under Different Technologies'. *Journal of Productivity Analysis* 21,1(January 2004):91–103.

Belotti, F., S. Daidone, G. Ilardi, and V. Atella. 'Stochastic frontier analysis using Stata'. *The Stata Journal* 13,4(2013):719–758.

Besseah, F. H. F. A., and S. Kim. 'Technical Efficiency of Cocoa Farmers in Ghana'. *Journal of Rural Development/Nongchon-Gyeongje* 37,2(2014):159–182.

Caudill, S. B., J. M. Ford, and D. M. Gropper. 'Frontier Estimation and Firm-Specific Inefficiency Measures in the Presence of Heteroscedasticity'. *Journal of Business & Economic Statistics*

13,1(January 1995):105.

Charnes, A., W. W. Cooper, and E. Rhodes. 'Measuring the efficiency of decision making units'. *European Journal of Operational Research* 2,6(November 1978):429–444.

Charnes, A., W. W. Cooper, and E. Rhodes. 'Evaluating Program and Managerial Efficiency: An Application of Data Envelopment Analysis to Program Follow Through'. *Management Science* 27,6(June 1981):668–697.

Coelli, T. 'Estimators and hypothesis tests for a stochastic frontier function: A Monte Carlo analysis'. *Journal of Productivity Analysis* 6,3(September 1995):247–268.

Danso-Abbeam, G., and L. J. S. Baiyegunhi. 'Technical efficiency and technology gap in Ghana's cocoa industry: accounting for farm heterogeneity'. *Applied Economics* (July 2019):1–13.

Donkor, E., N. Matthews, and A. A. Ogundeji. 'Efficiency of Rice Farming in Ghana: Policy Implications for Rice Sector Development'. *African Development Review* 30,2(June 2018):149–161.

Ebewore, S. O., and R. A. Isiorhovoja. 'Knowledge Status and Disease Control Practices of Cassava Farmers in Delta State, Nigeria: Implications for Extension Delivery'. *Open Agriculture* 4,1(April 2019):173–186.

Etwire, P. M., E. Martey, and W. Dogbe. 'Technical Efficiency of Soybean Farms and Its Determinants in Saboba and Chereponi Districts of Northern Ghana: A Stochastic Frontier Approach'. *Sustainable Agriculture Research* 2,4(September 2013):106.

FAO, IFAD, UNICEF, WFP, and WHO. *The State of Food Security and Nutrition in the World 2018. Building climate resilience for food security and nutrition. Rome, FAO*. 2018.

Farrell, M. J. 'The Measurement of Productive Efficiency'. *Journal of the Royal Statistical Society. Series A (General)* 120,3(1957):253.

Fu, H., Y. Qu, and Y. Pan. 'Efficiency of Cassava Production in China: Empirical Analysis of Field Surveys from Six Provinces'. *Applied Sciences* 8,8(August 2018):1356.

Ghana Statistical Service [GSS]. *2010 Population and Housing Census Summary Results of Final Report*. Accra,Ghana. 2012. www. statsghana.gov.gh (Accessed

Gutierrez, R. G., S. Carter, and D. M. Drukker. 'On boundary-value likelihood-ratio tests'. *Stata Technical Bulletin* 10,60(2001).

Hayami, Y. 'Sources of Agricultural Productivity Gap among Selected Countries'. *American Journal of Agricultural Economics* 51,3(August 1969):564.

Hayami, Y., and V. W. Ruttan. 'Agricultural Productivity Differences among Countries'. *The American Economic Review*. American Economic Association.

Hayami, Y., and V. W. Ruttan. 'Agricultural development: an international perspective.' *Agricultural Development: An International Perspective.* (1971).

Huang, C. J., T.-H. Huang, and N.-H. Liu. 'A new approach to estimating the metafrontier production function based on a stochastic frontier framework'. *Journal of Productivity*

Analysis (2014).

Institute of Statistical Social and Economic Research (ISSER), and Economic Growth Center (EGC). 'Ghana - Socioeconomic Panel Survey: 2009-2010'. 2016. http://microdata.worldbank.org/index.php/catalog/2534 (Accessed 9 November 2018).

Meeusen, W., and J. van Den Broeck. 'Efficiency Estimation from Cobb-Douglas Production Functions with Composed Error'. *International Economic Review* 18,2(June 1977):435.

Ministry of Food and Agriculture [MOFA]. 'Agriculture in Ghana: Facts and figures (2016)'. *Document Provided by Ministry of Agriculture, Accra, Ghana*. Accra,Ghana.

Morse, S. 'A meta-analysis of the technical efficiency of yam production in Nigeria'. *Journal of Crop Improvement* 35,1(January 2021):69–95.

Odotei, I. 'Festivals in Ghana: Continuity, transformation and politicisation of tradition.' *Transactions of the Historical Society of Ghana* 6(2002).

Ogundari, K., and B. Brümmer. 'Estimating Technical Efficiency, Input substitution and complementary effects using Output Distance Function: A study of Cassava production in Nigeria'. *Agricultural Economics Review* 12,2(2011):62–79.

Ogundeji, P. A., and M. A. Fasehun. 'Insights from Festivals and Carnivals'. In *The Palgrave Handbook of African Oral Traditions and Folklore* (pp. 105–118). Cham: Springer International Publishing. 2021.

Ojo, M. P., and A. B. Ayanwale. 'Value chain financing and plantain production in Nigeria: an ex-ante approach'. *Financial Innovation* 5,1(December 2019):18.

Opoko, E., R. Dzene, S. Caria, F. Teal, and A. Zeitlin. 'Impacts of Group based Microfinance in Agriculture: Evidence from Ghana's Cocoa Abrabopa Association'. 2009. http://www.csae.ox.ac.uk/conferences/2009-edia/papers/473-CariaTealZeitlin.pdf (Accessed 16 October 2015).

Owusu Asante, B., R. A. Villano, and G. E. Battese. *The effect of the adoption of yam minisett technology on the technical efficiency of yam farmers in the forest-savanna transition zone of Ghana*. *African Journal of Agricultural and Resource Economics*. 2014.

Owusu, V. 'Technical Efficiency Of Technology Adoption By Maize Farmers In Three Agro-Ecological Zones Of Ghana'. *Review of Agricultural and Applied Economics* 19,02(November 2016):39–50.

Owusu, V., E. Donkor, and E. Owusu-Sekyere. 'Accounting for the Gender Technology Gap Amongst Smallholder Rice Farmers in Northern Ghana'. *Journal of Agricultural Economics* 69,2(June 2018):439–457.

Rahman, S., and B. O. Awerije. 'Technical and scale efficiency of cassava production system in Delta State, Nigeria: an application of Two-Stage DEA approach'. *Journal of Agriculture and Rural Development in the Tropics and Subtropics* 116,1(2015):59–69.

Rhebergen, T., T. Fairhurst, S. Zingore, M. Fisher, T. Oberthür, and A. Whitbread. 'Climate, soil and land-use based land suitability evaluation for oil palm production in Ghana'. *European*

Journal of Agronomy 81(November 2016):1–14.

Schmidt, P., and T.-F. Lin. 'Simple tests of alternative specifications in stochastic frontier models'. *Journal of Econometrics* 24,3(March 1984):349–361.

Tsiboe, F. 'Nationally Representative Farm/Household Level Dataset on Crop Production in Ghana from 1987-2017'. 2020. https://doi.org/10.13140/RG.2.2.15922.02249/1

Tsiboe, F. 'Chronic sources of low cocoa production in Ghana: new insights from meta-analysis of old survey data'. *Agricultural and Resource Economics Review* (March 2021):1–26.

Tsiboe, F., P. Aseete, and J. G. Djokoto. 'Spatiotemporal Comparison of Dry Beans and Groundnut Production Technology and Inefficiency in Ghana'. *Review of Agricultural and Applied Economics* 24,1(2021):76–87.

Tsiboe, F., J. Asravor, and E. Osei. 'Vegetable Production Technical Efficiency and Technology Gaps in Ghana'. *African Journal of Agricultural and Resource Economics* 14,4(2019).

Tsiboe, F., B. L. Dixon, L. L. Nalley, J. S. Popp, and J. Luckstead. 'Estimating the impact of farmer field schools in sub-Saharan Africa: the case of cocoa'. *Agricultural Economics* 47,3(May 2016):329–339.

Van Nguyen, Q., S. Pascoe, L. Coglan, and S. Nghiem. 'The sensitivity of efficiency scores to input and other choices in stochastic frontier analysis: an empirical investigation'. *Journal of Productivity Analysis* 55,1(February 2021):31–40.

Wongnaa, C. A., and D. Awunyo-Vitor. 'Scale efficiency of maize farmers in four agro ecological zones of Ghana: A parametric approach'. *Journal of the Saudi Society of Agricultural Sciences* 18,3(July 2019):275–287.

World Bank. 'World Development Indicators'. 2018. https://data.worldbank.org/products/wdi (Accessed 24 September 2018).

Tables

Table 1. Summary Statistics of Starchy Staple Producing Farmers in Ghana (1987-2017**)**

Variable	Mean (SD)	Trend (%)
Household [a]		
Size (AE)	4.83 † (2.886)	0.10 [0.055]
Dependency (ratio)	1.29 (1.561)	0.16 [0.113]
Farmer [b]		
Female (dummy)	0.29 † (0.454)	-0.17* † [0.031]
Age (years)	46.83 (14.870)	0.47* [0.029]
Education (years)	5.01 † (4.819)	1.58* † ‡ [0.103]
Land owned (dummy)	0.61 † (0.489)	-0.03 † [0.033]
Land (ha) [b]		
Cassava	1.17 † (2.754)	-54.13 † [59.529]
Yam	1.57 † (3.655)	-13.88* † [2.141]
Cocoyam	1.56 (3.884)	2.84 [495.041]
Plantain	1.19 † (2.613)	-7.19 † [102.818]
Yield (Mt/ha) [b]		
Cassava	0.66 † (1.080)	2.78* † [0.220]
Yam	0.96 (1.540)	-38.47 † [3146.763]
Cocoyam	0.31 † (0.547)	0.15 [0.487]
Plantain	0.53 † (0.838)	1.57* † [0.296]
Input use [b]		
Planting material (Mt/ha)	0.06 † ‡ (0.428)	-5.68 † ‡ [20.430]
Family labor (AE)	2.74 † (1.543)	0.45* [0.054]
Hired labor (man-days/ha)	11.29 ‡ (43.202)	1.24* † ‡ [0.350]
Pesticide (Liter/ha)	7.78 † (49.023)	-71.16 † ‡ [7439.006]
Enabling environment [a]		
Credit (dummy)	0.19 † ‡ (0.390)	-0.19* ‡ [0.028]
Mechanization (dummy)	0.04 † ‡ (0.201)	-0.21* ‡ [0.015]
Extension (dummy)	0.19 (0.392)	0.75* † ‡ [0.029]

* Indicates significance at p<0.05
† and *‡* indicate statistically significant (p<0.05) differences in the variables across ecology and crop, respectively. The differences were determined via a linear regression for continuous variables and a probit model for dummies. A trend variable, and a fixed effect for ecology and crop, as well as their interactions, were included in the estimation.
[a] Household sample size; Cassava [11,458], Yam [3,439], Cocoyam [1,830], Plantain [6,163], Pooled [15,130]
[b] Farmer sample size; Cassava [11,584], Yam [3,468], Cocoyam [1,834], Plantain [6,223], Pooled [15,402]
Data Sources: Ghana Living Standards Surveys [wave 1-7] and Ghana Socioeconomic Panel Survey [wave 1-2]

Table 2. Hypothesis Tests for Ecology- and Meta- Frontier Models for Starchy Staple Production in Ghana (1987-2017)

	Sample size	Log-likelihood	Cobb-Douglas test	Coelli, (1995) [ab] skewness test	Gutierrez (2001) [a] LR test	Inefficiency variance $[\sigma_u]$	Total variance $[\sigma^2 = \sigma_u^2 + \sigma_v^2]$	Gamma $[\gamma = \sigma_u^2/\sigma^2]$	Inefficiency function test	Model significance
Cassava										
Guinea Savanah	801	-920	228.46***	-6.02*	11.27***	0.86 (0.092)	1.10 (0.119)	0.68*** (0.076)	36.04***	712.76***
Transitional Zone	2,064	-2,735	308.53***	-3.52*	13.95***	0.94 (0.088)	1.46 (0.118)	0.61*** (0.066)	87.91***	1196.39***
Forest Zone	7,022	-9,127	554.11***	-5.13*	19.07***	0.77 (0.061)	1.20 (0.065)	0.50*** (0.053)	109.90***	3947.44***
Coastal Savanah	1,697	-2,155	211.06***	-2.84*	6.13**	0.80 (0.114)	1.21 (0.126)	0.53*** (0.097)	62.11***	1228.60***
National	11,584	-15,345	1012.73***	-9.07*	66.27***	0.89 (0.038)	1.37 (0.048)	0.58*** (0.030)	196.00***	6321.83***
Meta-frontier	11,584	-260	16013.93***	4554.87	#N/A	0.00 (0.027)	0.05 (0.001)	0.00 (0.001)	14868.21***	88548.64***
Yam										
Sudan Savanah	222	-233	163.91***	-0.23	0.52	0.87 (0.401)	1.12 (0.480)	0.68** (0.338)	276.27***	501.21***
Guinea Savanah	1,472	-1,732	401.08***	-9.80*	34.52***	1.04 (0.064)	1.34 (0.103)	0.81*** (0.041)	25.93*	2381.50***
Transitional Zone	943	-1,289	199.23***	-4.89*	32.22***	1.35 (0.091)	2.16 (0.195)	0.84*** (0.042)	2038.88***	1327.19***
Forest Zone	831	-1,212	169.69***	-3.31*	23.13***	1.41 (0.115)	2.51 (0.251)	0.79*** (0.054)	47.90***	769.40***
National	3,468	-4,936	515.81***	-12.13*	226.72***	1.53 (0.040)	2.67 (0.104)	0.88*** (0.014)	215.92***	4671.21***
Meta-frontier	3,468	-2,600	2217.78***	-197.52*	741.95***	0.72 (0.011)	0.53 (0.015)	0.97*** (0.003)	3533.33***	16702.58***
Cocoyam										
Transitional Zone	422	-597	99.26***	-1.84	9.07***	1.38 (0.171)	2.40 (0.359)	0.79*** (0.084)	263.53***	322.12***
Forest Zone	1,412	-2,044	74.15***	-3.16*	25.76***	1.36 (0.104)	2.37 (0.211)	0.78*** (0.053)	43.77***	500.01***
National	1,834	-2,720	111.21***	-2.55*	20.72***	1.27 (0.105)	2.30 (0.194)	0.71*** (0.059)	56.41***	614.85***
Meta-frontier	1,834	-371	1557.34***	651.41	#N/A	0.00 (0.160)	0.08 (0.003)	0.00 (0.007)	164.16***	9002.26***
Plantain										
Transitional Zone	739	-983	129.61***	-5.70*	39.31***	1.38 (0.085)	2.21 (0.194)	0.86*** (0.035)	61.58***	764.77***
Forest Zone	4,980	-6,565	355.08***	-6.30*	34.56***	0.96 (0.058)	1.45 (0.079)	0.64*** (0.043)	46.19***	3485.90***
Coastal Savanah	504	-576	110.41***	0.28	#N/A	0.03 (0.687)	0.61 (0.046)	0.00 (0.068)	1127.47***	392.89***
National	6,223	-8,274	448.17***	-9.13*	76.05***	1.06 (0.044)	1.61 (0.068)	0.70*** (0.030)	85.93***	4374.08***
Meta-frontier	6,223	1,235	9327.65***	-1887.87*	1003.92***	0.40 (0.007)	0.19 (0.005)	0.82*** (0.009)	1447.93***	110390.91***

Significance levels: * p<0.10, ** p<0.05, ***p<0.01
[a] Null hypothesis of no one-sided error (i.e. no inefficiency) was tested
[b] Values less than the critical value of -1.96 confirm the rejection of the null hypothesis.
Data Sources: Ghana Living Standards Surveys [wave 1-7] and Ghana Socioeconomic Panel Survey [wave 1-2]

Table 3. Elasticities for Ecology- and Meta- Frontier Models for Starchy Staple Production in Ghana (1987-2017)

	Land elasticity	Planting material elasticity	Family labor elasticity	Hired Labor elasticity	Pesticide elasticity	Returns to scale [a]	Technical change
Cassava							
Guinea Savanah	0.31*** (0.030)	-0.13*** (0.025)	0.16*** (0.063)	0.05*** (0.008)	0.00 (0.014)	0.39*** (0.069)	0.14 (0.090)
Transitional Zone	0.36*** (0.019)	0.02 (0.018)	0.10** (0.042)	0.04*** (0.009)	0.01 (0.007)	0.53*** (0.047)	-0.07 (0.057)
Forest Zone	0.40*** (0.010)	-0.04*** (0.010)	0.14*** (0.023)	0.04*** (0.003)	-0.01* (0.004)	0.53*** (0.026)	-0.01 (0.031)
Coastal Savanah	0.41*** (0.021)	-0.05*** (0.019)	0.17*** (0.047)	0.04*** (0.007)	-0.09*** (0.018)	0.47*** (0.060)	0.01 (0.052)
National	0.39*** (0.008)	-0.03*** (0.008)	0.15*** (0.018)	0.04*** (0.003)	-0.01** (0.004)	0.55*** (0.020)	-0.04* (0.023)
Meta-frontier	0.39*** (0.002)	-0.04*** (0.002)	0.16*** (0.005)	0.04*** (0.001)	-0.01*** (0.001)	0.54*** (0.006)	-0.06*** (0.007)
Yam							
Sudan Savanah	0.44*** (0.068)	-0.08** (0.032)	0.18* (0.103)	0.01 (0.015)	-0.03 (0.020)	0.51*** (0.133)	0.30 (0.197)
Guinea Savanah	0.59*** (0.025)	-0.08*** (0.019)	0.02 (0.047)	0.02*** (0.005)	-0.02 (0.013)	0.51*** (0.055)	0.03 (0.066)
Transitional Zone	0.55*** (0.035)	0.00 (0.020)	-0.06 (0.061)	0.10*** (0.016)	0.02 (0.011)	0.61*** (0.071)	0.07 (0.108)
Forest Zone	0.32*** (0.030)	-0.08* (0.043)	0.20*** (0.076)	0.09*** (0.014)	-0.01 (0.014)	0.53*** (0.091)	0.11 (0.084)
National	0.55*** (0.018)	-0.06*** (0.013)	0.12*** (0.035)	0.05*** (0.005)	-0.02*** (0.007)	0.64*** (0.040)	0.00 (0.049)
Meta-frontier	0.52*** (0.010)	-0.06*** (0.007)	0.12*** (0.019)	0.06*** (0.003)	-0.01*** (0.004)	0.63*** (0.021)	0.04 (0.028)
Cocoyam							
Transitional Zone	0.19*** (0.040)	-0.18** (0.083)	0.21** (0.104)	0.08*** (0.022)	-0.03 (0.034)	0.27*** (0.134)	-0.65* (0.361)
Forest Zone	0.42*** (0.041)	-0.10*** (0.037)	0.07 (0.059)	0.04*** (0.010)	0.02 (0.016)	0.45*** (0.076)	0.13 (0.136)
National	0.36*** (0.027)	-0.10*** (0.035)	0.09 (0.052)	0.05*** (0.009)	0.01 (0.014)	0.42*** (0.066)	-0.08 (0.107)
Meta-frontier	0.38*** (0.005)	-0.10*** (0.015)	0.11*** (0.015)	0.05*** (0.003)	0.01*** (0.004)	0.45*** (0.023)	-0.10*** (0.039)
Plantain							
Transitional Zone	0.44*** (0.031)	-0.04 (0.032)	0.11* (0.066)	0.06*** (0.011)	0.02 (0.015)	0.59*** (0.073)	-0.01 (0.081)
Forest Zone	0.47*** (0.015)	-0.03** (0.013)	0.14*** (0.028)	0.04*** (0.004)	0.00 (0.005)	0.63*** (0.031)	-0.01 (0.039)
Coastal Savanah	0.37*** (0.032)	0.06* (0.033)	0.12* (0.073)	0.07*** (0.015)	-0.05** (0.023)	0.57*** (0.098)	-0.02 (0.106)
National	0.47*** (0.012)	-0.02* (0.011)	0.14*** (0.025)	0.04*** (0.004)	0.00 (0.005)	0.63*** (0.028)	-0.01 (0.031)

Meta-frontier	0.47*** (0.002)	-0.03*** (0.002)	0.14*** (0.007)	0.04*** (0.001)	0.01*** (0.001)	0.63*** (0.007)	0.02 (0.017)

Significance levels: * p<0.10, ** p<0.05, ***p<0.01

[a] Null hypothesis of constant returns to scale was tested

Data Sources: Ghana Living Standards Surveys [wave 1-7] and Ghana Socioeconomic Panel Survey [wave 1-2]

Table 4. Ghanaian Starchy Staple Production Technology Level and Technical Efficiency Across Ecologies (1987-2017)

	Technology gap ratio (TGR)	Technical efficiency (TE)	Meta-technical efficiency (MTE)	Technology gap
Cassava				
Guinea Savanah	0.94*** (0.002)	0.64*** (0.006)	0.61*** (0.006)	11.60%
Transitional Zone	0.92*** (0.001)	0.58*** (0.004)	0.55*** (0.003)	10.31%
Forest Zone	0.90*** (0.000)	0.64*** (0.001)	0.57*** (0.001)	7.50%
Coastal Savanah	0.88*** (0.003)	0.64*** (0.004)	0.56*** (0.003)	6.08%
Mean	0.91	0.63	0.57	8.87%
Yam				
Sudan Savanah	0.98*** (0.005)	0.67*** (0.018)	0.66*** (0.018)	1.86%
Guinea Savanah	0.98*** (0.002)	0.54*** (0.005)	0.53*** (0.005)	2.43%
Transitional Zone	0.93*** (0.004)	0.49*** (0.007)	0.46*** (0.007)	7.16%
Forest Zone	0.84*** (0.006)	0.51*** (0.008)	0.41*** (0.008)	15.94%
Mean	0.932	0.554	0.515	
Cocoyam				
Transitional Zone	0.80*** (0.008)	0.57*** (0.011)	0.42*** (0.008)	19.93%
Forest Zone	0.84*** (0.002)	0.48*** (0.005)	0.41*** (0.004)	15.60%
Mean	0.82	0.52	0.41	17.77%
Plantain				
Transitional Zone	0.94*** (0.002)	0.50*** (0.008)	0.47*** (0.007)	23.76%
Forest Zone	0.87*** (0.000)	0.58*** (0.002)	0.52*** (0.002)	12.57%
Coastal Savanah	0.76*** (0.005)	0.68*** (0.009)	0.51*** (0.008)	6.07%
Mean	0.907	0.542	0.495	9.32%
Overall mean	0.877	0.555	0.482	12.28%

Significance levels: * p<0.10, ** p<0.05, ***p<0.01

[a] Null hypothesis of constant returns to scale was tested

Data Sources: Ghana Living Standards Surveys [wave 1-7] and Ghana Socioeconomic Panel Survey [wave 1-2]

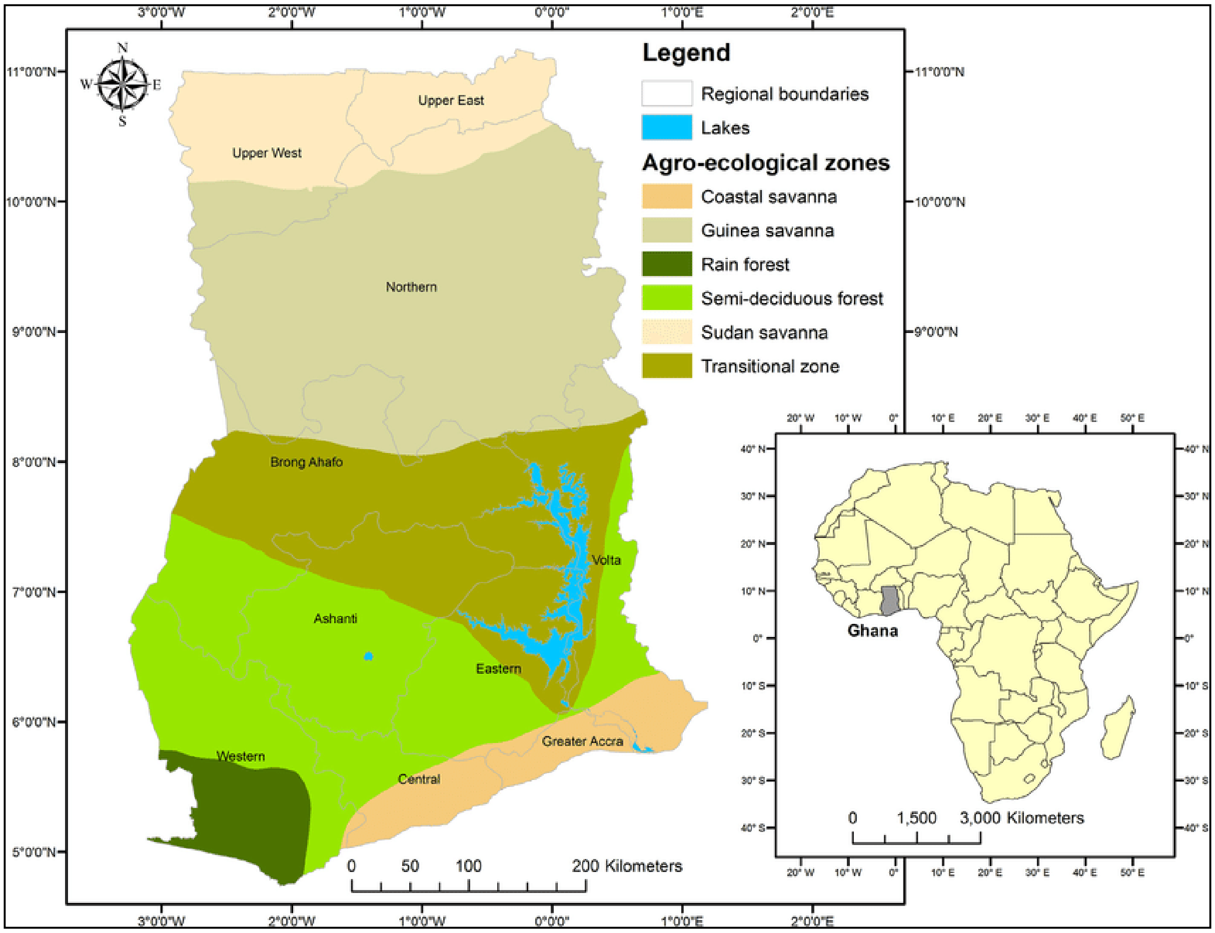

Figure 1. Ecological Zones of Ghana

Source: Retrieved from Rhebergen et al. (2016)

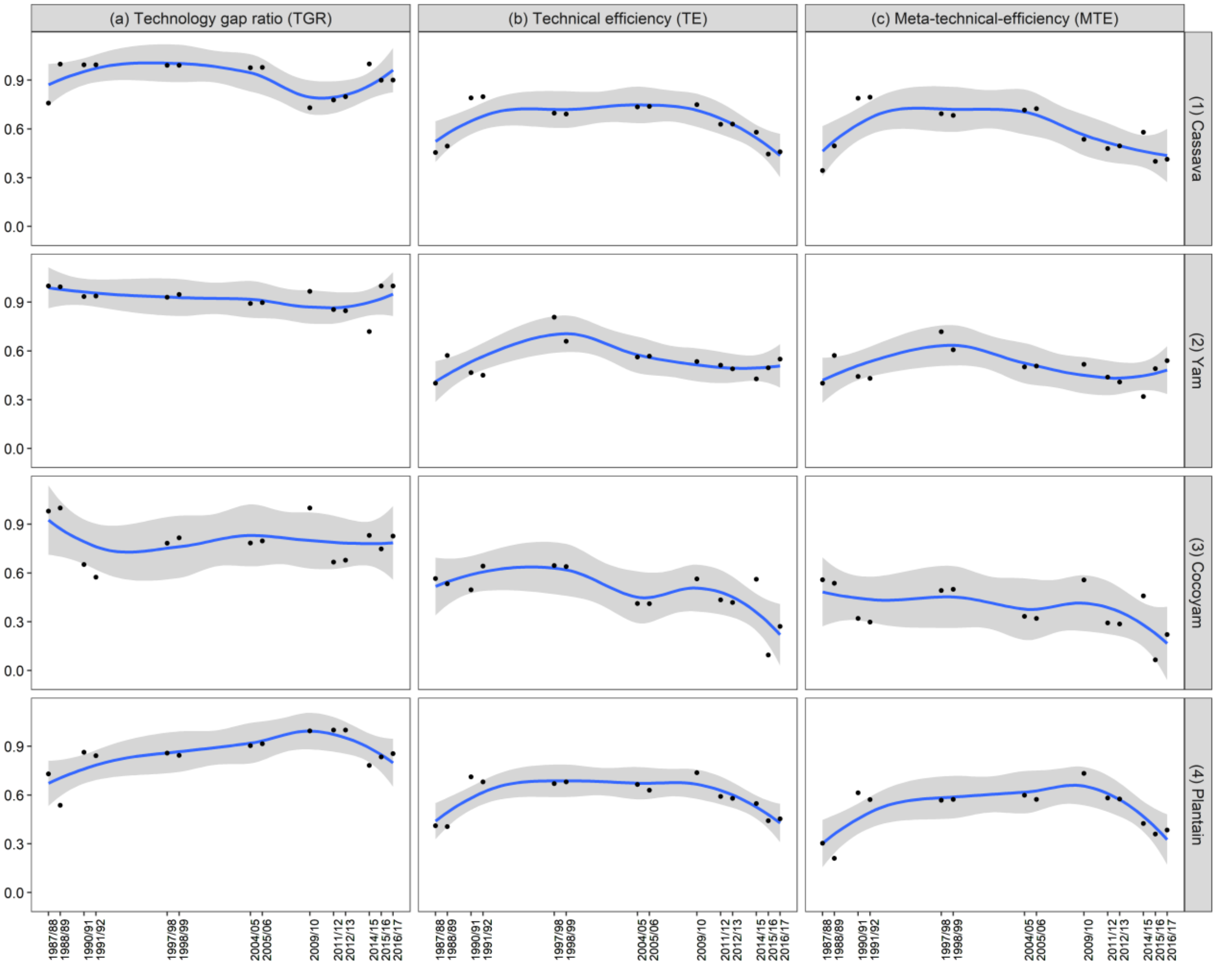

Figure 2. Ghanaian Starchy Staple Production Technology Level and Technical Efficiency Parameters Across Seasons (1987-2017)

Notes: Farmer level scores were first estimated via a Meta−stochastic−frontier (MSF) analysis applied separately to 10 population−based surveys that represent 30 years of farmer level data collection in Ghana the surveys used included Ghana Living Standards Surveys [wave 1−7] and Ghana Socioeconomic Panel Survey [wave 1−2]. The farmer-level elasticities were subsequently averaged across seasons via a regression framework to account for controls. Each point on a sub−panel represents the mean of the estimates. Given the seasonal means, the fitted line was done locally using neighborhood points, weighted by distance. The size of the neighborhood was set to 75% of the points with a tri−cubic weighting. The gray region is the 95% confidence interval of the fitted line.

Appendix S: supplementary materials

Table S1. Ecology Population, Climatic, and Soil Characteristics in Ghana.

Variable	Sudan Savanah	Guinea Savanna	Transitional Zone	Semi-Deciduous Forest	Rain Forest	Coastal Savanna
Area (1000 km^2)	50.12	25.92	14.21	16.66	18.48	10.81
Pop. Den. (N/km^2)	59.09	162.76	140.14	89.61	37.99	115.97
Agric Pop. (%)	55.41	38.73	55.00	45.59	77.09	31.63
Rural Pop. (%)	48.50	38.39	56.68	48.42	79.46	30.48
Soil pH	4.28-7.40	4.13-7.44	4.10-7.66	4.21-7.34	4.40-6.70	4.29-8.28
Soil Org. matter (%)	0.52-6.74	0.06-7.63	0.00-11.18	0.05-11.31	0.19-13.83	0.00-4.84
Soil N (%)	0.00-0.11	0.00-0.18	0.00-0.39	0.00-0.39	0.00-0.37	0.00-0.25
Soil P (mg/kg soil)	0.00-4.54	0.00-6.35	0.00-3.41	0.00-3.92	0.00-0.26	0.00-7.51
Soil CEC	0.00-6.41	0.00-7.86	0.00-9.55	0.00-6.54	0.00-1.21	0.00-6.96
10-year avg rain						
Mean [mm/year]	943.29	1,128.52	1,244.52	1,310.65	1,443.30	1,064.89
CV [%]	17.55	10.54	9.85	9.57	13.36	10.93

All values are calculated as weighted averages (by area) using data retried from published reports Agriculture in Ghana: Facts and figures (2016)

Table S2. Ecology Level **Starchy Staple** Production Statistics in Ghana for 2010/2015

Ecology/Crop	Cultivating Household [a]			Farm size (ha) [a]	Area share (%) [b]	Production share (%) [b]	Yield ratio [b]
	Count (1000)	Share (%)	Size (count)				
Cassava							
Semi-Deciduous Forest	1647.48 (1269.22)	59.47 (6.27)	5.06 (0.84)	0.39 (0.22)	46.54 (2.26)	49.06 (3.00)	1.00 (0.00)
Transitional Zone	558.77 (490.46)	18.48 (4.09)	5.56 (0.81)	0.81 (0.59)	26.00 (1.36)	27.42 (2.27)	1.00 (0.09)
Coastal Savanna	384.44 (396.35)	10.58 (3.16)	5.05 (1.03)	0.83 (0.60)	13.61 (0.62)	11.60 (0.67)	0.81 (0.06)
Guinea Savanna	273.88 (323.54)	6.17 (4.42)	7.81 (1.56)	2.35 (2.14)	10.77 (3.71)	10.24 (3.86)	0.92 (0.25)
Rain Forest	140.94 (105.37)	5.18 (0.76)	5.31 (0.96)	0.25 (0.15)	2.58 (0.28)	1.51 (0.22)	0.56 (0.05)
Sudan Savanah	3.07 (2.59)	0.14 (0.13)	4.57 (0.91)	3.73 (4.10)	0.58 (0.38)	0.19 (0.05)	0.35 (0.10)
Yam							
Transitional Zone	298.94 (252.41)	34.45 (8.29)	5.64 (0.86)	1.06 (0.62)	50.73 (9.70)	52.51 (10.28)	1.46 (0.04)
Guinea Savanna	441.81 (451.37)	38.86 (9.20)	7.78 (1.68)	0.98 (0.96)	31.30 (11.57)	33.77 (12.01)	1.54 (0.24)
Semi-Deciduous Forest	164.93 (113.55)	21.25 (5.56)	5.04 (0.29)	0.39 (0.22)	12.03 (2.48)	8.55 (1.81)	1.00 (0.00)
Sudan Savanah	39.68 (44.83)	3.00 (1.36)	6.67 (0.78)	2.43 (2.30)	5.27 (0.75)	4.88 (0.25)	1.32 (0.18)
Coastal Savanna	27.83 (31.93)	2.01 (1.14)	3.86 (1.57)	0.38 (0.34)	0.57 (0.15)	0.26 (0.11)	0.62 (0.08)
Rain Forest	3.85 (3.12)	0.44 (0.31)	5.60 (2.15)	0.23 (0.19)	0.10 (0.02)	0.04 (0.01)	0.50 (0.11)
Cocoyam							
Semi-Deciduous Forest	507.52 (417.81)	76.76 (3.65)	5.10 (0.95)	0.64 (0.43)	81.79 (9.17)	83.96 (1.72)	1.00 (0.00)
Transitional Zone	96.49 (75.16)	16.54 (5.19)	5.50 (1.38)	0.45 (0.27)	12.22 (1.78)	13.99 (1.69)	1.11 (0.05)
Coastal Savanna	31.59 (35.94)	3.17 (2.25)	3.99 (1.60)	0.73 (0.83)	1.38 (0.63)	1.21 (0.75)	0.82 (0.16)
Rain Forest	31.89 (35.58)	3.45 (1.81)	5.53 (1.91)	0.15 (0.12)	0.62 (0.31)	0.55 (0.41)	0.82 (0.14)
Guinea Savanna	0.33 (0.01)	0.14 (0.03)	7.40 (3.58)	55.35 (116.28)	6.39 (13.03)	0.47 (0.05)	0.69 (0.39)
Plantain							
Semi-Deciduous Forest	1259.52 (992.94)	78.26 (3.50)	5.01 (0.71)	0.36 (0.21)	80.01 (0.73)	82.38 (0.82)	1.00 (0.00)
Transitional Zone	175.63 (144.96)	10.84 (2.54)	5.12 (0.90)	0.59 (0.38)	16.85 (0.42)	15.29 (0.67)	0.88 (0.03)
Coastal Savanna	157.98 (173.84)	7.02 (3.70)	4.85 (0.85)	0.19 (0.15)	2.40 (0.53)	1.74 (0.25)	0.71 (0.08)
Rain Forest	69.62 (62.34)	3.87 (0.86)	5.40 (1.43)	0.08 (0.06)	0.72 (0.05)	0.57 (0.05)	0.77 (0.05)

[a] Calculated by authors from data retrieved from GLSS Waves 5-7
[b] Calculated by authors from data retrieved from FAO CountryStat

Table S3. Stochastic Meta-Frontier Results for Cassava Production in Ghana (1987-2017)

	Guinea Savanah	Transitional Zone	Forest Zone	Coastal Savanah	National	Meta-frontier
**Production function**						
Land(ln[ha]) {lnI1}	-0.22** (0.099)	0.23*** (0.062)	0.16*** (0.034)	0.17** (0.076)	0.17*** (0.027)	0.15*** (0.007)
Seed (ln[Mt]) {lnI2}	0.19*** (0.061)	-0.07* (0.037)	0.04** (0.019)	0.07* (0.035)	0.03** (0.015)	0.04*** (0.004)
Hired labor(ln[days]) {lnI3}	0.13 (0.189)	0.23* (0.134)	0.06 (0.073)	0.14 (0.174)	0.09 (0.058)	0.13*** (0.016)
Family labor(ln[days]) {lnI4}	0.17*** (0.044)	0.10*** (0.032)	0.10*** (0.015)	0.08*** (0.031)	0.10*** (0.012)	0.10*** (0.003)
Pesticide (ln[liter]) {lnI5}	0.10* (0.049)	0.12*** (0.031)	0.08*** (0.013)	0.16*** (0.033)	0.08*** (0.011)	0.08*** (0.003)
Trend {I6}	-0.17 (0.199)	-0.17 (0.162)	0.20*** (0.068)	-0.19 (0.117)	0.03 (0.053)	0.02 (0.015)
P5·lnI1·lnI1	-0.04 (0.038)	-0.07*** (0.023)	-0.04*** (0.012)	-0.02 (0.026)	-0.05*** (0.009)	-0.04*** (0.003)
P5·lnI2·lnI2	0.05*** (0.010)	0.01 (0.006)	0.02*** (0.003)	0.03*** (0.007)	0.02*** (0.003)	0.02*** (0.001)
P5·lnI3·lnI3	0.13 (0.171)	0.12 (0.121)	-0.06 (0.066)	-0.04 (0.140)	-0.02 (0.050)	-0.01 (0.016)
P5·lnI4·lnI4	0.04*** (0.007)	0.02*** (0.005)	0.03*** (0.003)	0.02*** (0.005)	0.03*** (0.002)	0.03*** (0.001)
P5·lnI5·lnI5	0.02** (0.009)	0.03*** (0.006)	0.02*** (0.003)	0.04*** (0.008)	0.02*** (0.002)	0.02*** (0.001)
P5·I6·I6	0.02** (0.009)	0.03*** (0.006)	0.02*** (0.003)	0.04*** (0.008)	0.02*** (0.002)	0.02*** (0.001)
lnI1.lnI2	0.00 (0.008)	0.01** (0.005)	0.00 (0.003)	-0.01** (0.005)	0.00 (0.002)	0.00 (0.001)
lnI1.lnI3	0.09* (0.048)	-0.01 (0.035)	-0.02 (0.018)	-0.04 (0.038)	-0.01 (0.014)	-0.01** (0.004)
lnI1.lnI4	-0.02** (0.007)	-0.01 (0.005)	0.00 (0.002)	0.01** (0.005)	0.00* (0.002)	0.00*** (0.001)
lnI1.lnI5	-0.01 (0.007)	0.01* (0.005)	-0.01*** (0.002)	0.00 (0.005)	0.00 (0.002)	0.00** (0.001)
lnI1.I6	0.02*** (0.003)	0.01*** (0.002)	0.01*** (0.001)	0.01*** (0.002)	0.01*** (0.001)	0.01*** (0.000)
lnI2.lnI3	0.00 (0.015)	0.00 (0.011)	-0.01 (0.006)	0.01 (0.013)	0.00 (0.005)	0.00** (0.001)
lnI2.lnI4	0.00 (0.002)	0.00** (0.001)	-0.01*** (0.001)	-0.01*** (0.001)	0.00*** (0.001)	0.00*** (0.000)
lnI2.lnI5	0.00 (0.002)	0.00** (0.002)	0.00 (0.001)	0.00 (0.002)	0.00** (0.001)	0.00*** (0.000)
lnI2.I6	0.00 (0.001)	0.01*** (0.001)	0.00*** (0.000)	0.00*** (0.001)	0.00*** (0.000)	0.00*** (0.000)
lnI3.lnI4	-0.04** (0.014)	0.03*** (0.010)	0.00 (0.005)	0.00 (0.011)	0.00 (0.004)	0.00 (0.001)
lnI3.lnI5	0.00 (0.015)	-0.01 (0.010)	0.00 (0.005)	0.00 (0.013)	0.00 (0.004)	0.00 (0.001)
lnI3.I6	-0.01 (0.006)	0.00 (0.004)	0.00 (0.002)	0.00 (0.005)	0.00 (0.002)	0.00 (0.001)
lnI4.lnI5	0.00 (0.002)	0.00 (0.001)	0.00 (0.001)	0.00 (0.001)	0.00 (0.001)	0.00*** (0.000)
lnI4.I6	0.00 (0.001)	0.00** (0.001)	0.00 (0.000)	0.00 (0.001)	0.00 (0.000)	0.00*** (0.000)
lnI5.I6	0.00* (0.001)	0.00 (0.001)	0.00 (0.000)	0.00* (0.001)	0.00* (0.000)	0.00*** (0.000)
Constant	-1.77 (3.003)	2.21 (1.938)	-0.94 (0.919)	0.12 (1.567)	0.80 (0.694)	1.27*** (0.205)
Source (base=GLSS7)						
GLSS1	2.00 (2.924)	-1.64 (1.961)	1.12 (0.917)	-0.33 (1.561)	-0.48 (0.697)	-0.73*** (0.205)
GLSS2	2.06 (2.762)	-2.42 (1.846)	0.76 (0.876)	0.24 (1.497)	-0.75 (0.662)	-1.25*** (0.197)
GLSS3	2.24 (2.356)	-1.92 (1.511)	0.07 (0.782)	0.40 (1.310)	-0.99* (0.575)	-1.46*** (0.174)
GLSS4	3.32* (1.723)	-0.86 (1.084)	-0.43 (0.633)	0.77 (1.010)	-0.78* (0.451)	-1.24*** (0.141)
GLSS5	3.36*** (1.137)	0.07 (0.795)	-0.85* (0.464)	1.28* (0.745)	-0.59* (0.333)	-0.89*** (0.110)
GLSS6	2.08*** (0.496)	0.54 (0.364)	-0.40* (0.215)	-0.17 (0.393)	-0.20 (0.155)	-0.12* (0.070)
GSPS1	2.07*** (0.760)	0.57 (0.560)	-0.91*** (0.334)	1.43*** (0.545)	-0.39 (0.240)	-0.33*** (0.081)
GSPS2	0.60 (0.382)	0.69*** (0.244)	0.10 (0.142)	-0.14 (0.272)	0.07 (0.106)	0.00 (0.055)
**Uncertainty function**						
Mean	-0.99*** (0.155)	-0.64*** (0.078)	-0.52*** (0.039)	-0.66*** (0.098)	-0.55*** (0.035)	-2.99*** (0.045)
Variance	0.61 (0.047)	0.72 (0.028)	0.77 (0.015)	0.72 (0.035)	0.76 (0.013)	0.22 (0.005)

Significance levels: * p<0.10, ** p<0.05, ***p<0.01; Data Sources: Ghana Living Standards Surveys [wave 1-7] and Ghana Socioeconomic Panel Survey [wave 1-2]

Table S4. Stochastic Meta-Frontier Results for Yam Production in Ghana (1987-2017)

	**Sudan Savanah**	**Guinea Savanah**	**Transitional Zone**	**Forest Zone**	**National**	**Meta-frontier**
**Production function**						
Land(ln[ha]) {lnI1}	0.30 (0.284)	-0.10 (0.097)	0.27*** (0.094)	0.08 (0.128)	0.18*** (0.057)	0.10*** (0.028)
Seed (ln[Mt]) {lnI2}	0.31** (0.125)	0.21*** (0.040)	0.06 (0.051)	0.20** (0.080)	0.20*** (0.029)	0.22*** (0.014)
Hired labor(ln[days]) {lnI3}	1.71*** (0.453)	-0.10 (0.158)	-0.12 (0.221)	0.41 (0.331)	0.09 (0.120)	0.17** (0.068)
Family labor(ln[days]) {lnI4}	0.22** (0.102)	0.12*** (0.029)	0.19*** (0.048)	0.16*** (0.061)	0.18*** (0.024)	0.21*** (0.013)
Pesticide (ln[liter]) {lnI5}	-0.23* (0.121)	0.21*** (0.044)	0.13*** (0.044)	0.23*** (0.053)	0.17*** (0.026)	0.14*** (0.013)
Trend {I6}	0.48 (0.636)	-0.62*** (0.168)	0.05 (0.383)	0.99*** (0.182)	-0.08 (0.122)	0.15* (0.087)
P5·lnI1·lnI1	0.00 (0.067)	0.01 (0.025)	-0.10*** (0.030)	-0.09*** (0.032)	-0.04** (0.017)	-0.03*** (0.010)
P5·lnI2·lnI2	0.09*** (0.015)	0.06*** (0.008)	0.03*** (0.008)	0.04** (0.016)	0.05*** (0.005)	0.05*** (0.003)
P5·lnI3·lnI3	0.13 (0.306)	0.10 (0.136)	-0.12 (0.159)	-0.10 (0.216)	-0.05 (0.094)	-0.17*** (0.052)
P5·lnI4·lnI4	0.05*** (0.017)	0.03*** (0.005)	0.04*** (0.007)	0.05*** (0.008)	0.04*** (0.004)	0.05*** (0.002)
P5·lnI5·lnI5	0.02 (0.020)	0.04*** (0.007)	0.02** (0.010)	0.03*** (0.010)	0.04*** (0.005)	0.03*** (0.003)
P5·I6·I6	0.02 (0.020)	0.04*** (0.007)	0.02** (0.010)	0.03*** (0.010)	0.04*** (0.005)	0.03*** (0.003)
lnI1.lnI2	-0.01 (0.016)	-0.02*** (0.007)	0.00 (0.007)	0.00 (0.011)	-0.02*** (0.004)	-0.01*** (0.003)
lnI1.lnI3	0.13 (0.103)	0.07 (0.044)	0.02 (0.047)	0.01 (0.055)	0.05* (0.028)	0.06*** (0.016)
lnI1.lnI4	-0.02 (0.018)	0.00 (0.006)	0.00 (0.007)	0.01 (0.009)	-0.01 (0.004)	-0.01*** (0.002)
lnI1.lnI5	-0.01 (0.016)	-0.02*** (0.007)	-0.01 (0.006)	-0.01 (0.008)	-0.01 (0.004)	-0.01*** (0.002)
lnI1.I6	-0.01 (0.009)	0.02*** (0.003)	0.01*** (0.003)	0.01** (0.003)	0.01*** (0.002)	0.01*** (0.001)
lnI2.lnI3	0.06** (0.027)	0.00 (0.011)	0.01 (0.015)	0.00 (0.027)	0.00 (0.008)	-0.01* (0.005)
lnI2.lnI4	0.00 (0.004)	0.00*** (0.001)	0.00 (0.002)	-0.01 (0.005)	0.00* (0.001)	0.00*** (0.001)
lnI2.lnI5	-0.01 (0.006)	0.00 (0.002)	0.00 (0.002)	0.01* (0.003)	0.00 (0.001)	0.00** (0.001)
lnI2.I6	0.00 (0.004)	0.00*** (0.001)	0.01*** (0.001)	0.00 (0.002)	0.00*** (0.001)	0.00*** (0.000)
lnI3.lnI4	-0.01 (0.029)	-0.01 (0.011)	-0.01 (0.016)	-0.01 (0.020)	-0.02** (0.008)	-0.02*** (0.005)
lnI3.lnI5	0.00 (0.030)	-0.02 (0.013)	0.01 (0.015)	0.01 (0.019)	-0.01 (0.009)	0.00 (0.005)
lnI3.I6	-0.05*** (0.013)	0.00 (0.005)	0.00 (0.008)	-0.01 (0.008)	0.00 (0.004)	-0.01*** (0.002)
lnI4.lnI5	-0.01 (0.004)	0.00 (0.001)	0.00 (0.002)	0.00 (0.003)	0.00 (0.001)	0.00*** (0.001)
lnI4.I6	0.00 (0.002)	0.00 (0.001)	0.00 (0.001)	0.00*** (0.001)	0.00 (0.001)	0.00 (0.000)
lnI5.I6	0.01*** (0.003)	0.00 (0.001)	0.00* (0.001)	0.00 (0.001)	0.00 (0.001)	0.00* (0.000)
Constant	-11.81 (9.158)	4.49* (2.389)	-1.93 (4.629)	-6.22** (2.493)	0.63 (1.699)	-1.96* (1.158)
Source (base=GLSS7)						
GLSS1	11.12 (9.085)	-3.95* (2.310)	2.91 (4.607)	7.44*** (2.518)	0.38 (1.684)	2.92** (1.152)
GLSS2	9.58 (8.361)	-3.68* (2.171)	1.44 (4.284)	6.39*** (2.390)	0.03 (1.586)	2.32** (1.074)
GLSS3	9.02 (7.094)	-1.65 (1.872)	2.10 (3.367)	3.12 (2.122)	0.36 (1.356)	1.96** (0.859)
GLSS4	5.40 (4.137)	0.88 (1.273)	2.68 (2.006)	-1.44 (1.643)	1.01 (0.902)	1.22** (0.506)
GLSS5	4.37** (2.199)	2.44*** (0.827)	1.14 (1.322)	-2.94** (1.192)	0.81 (0.620)	0.80*** (0.290)
GLSS6	2.22*** (0.734)	1.80*** (0.356)	0.91 (0.663)	-2.46*** (0.578)	0.77*** (0.271)	0.67*** (0.125)
GSPS1	2.91** (1.185)	2.15*** (0.550)	1.23 (0.945)	-2.29*** (0.833)	0.75* (0.410)	0.70*** (0.178)
GSPS2	1.48*** (0.403)	1.92*** (0.239)	-0.53 (0.494)	-2.12*** (0.417)	1.29*** (0.186)	0.98*** (0.120)
**Uncertainty function**						
Mean	-1.32*** (0.230)	-1.28*** (0.159)	-0.85*** (0.215)	-0.56*** (0.143)	-1.06*** (0.091)	-1.44*** (0.045)
Variance	0.52 (0.059)	0.53 (0.042)	0.66 (0.071)	0.75 (0.054)	0.59 (0.027)	0.49 (0.011)

Significance levels: * p<0.10, ** p<0.05, ***p<0.01; Data Sources: Ghana Living Standards Surveys [wave 1-7] and Ghana Socioeconomic Panel Survey [wave 1-2]

Table S5. Stochastic Meta-Frontier Results for Cocoyam Production in Ghana (1987-2017)

	Transitional Zone	Forest Zone	National	Meta-frontier
**Production function**				
Land(ln[ha]) {lnI1}	0.01 (0.098)	0.15 (0.091)	0.11 (0.071)	0.13*** (0.029)
Seed (ln[Mt]) {lnI2}	0.21** (0.107)	0.20*** (0.048)	0.18*** (0.046)	0.19*** (0.023)
Hired labor(ln[days]) {lnI3}	0.19 (0.322)	0.04 (0.201)	-0.04 (0.192)	-0.01 (0.071)
Family labor(ln[days]) {lnI4}	0.16** (0.080)	0.12*** (0.038)	0.12*** (0.035)	0.12*** (0.017)
Pesticide (ln[liter]) {lnI5}	0.11 (0.079)	0.01 (0.042)	0.02 (0.037)	0.02 (0.014)
Trend {I6}	-0.88 (0.866)	0.11 (0.223)	-0.09 (0.189)	-0.06 (0.067)
P5·lnI1·lnI1	-0.19*** (0.046)	-0.12*** (0.029)	-0.14*** (0.025)	-0.14*** (0.008)
P5·lnI2·lnI2	0.07*** (0.023)	0.04*** (0.010)	0.04*** (0.010)	0.04*** (0.006)
P5·lnI3·lnI3	0.27 (0.258)	-0.05 (0.169)	0.01 (0.146)	-0.06 (0.040)
P5·lnI4·lnI4	0.04*** (0.011)	0.02*** (0.006)	0.03*** (0.005)	0.02*** (0.001)
P5·lnI5·lnI5	0.03 (0.017)	-0.01 (0.008)	0.00 (0.007)	0.00 (0.002)
P5·I6·I6	0.03 (0.017)	-0.01 (0.008)	0.00 (0.007)	0.00 (0.002)
lnI1.lnI2	-0.01 (0.009)	-0.01 (0.008)	-0.01 (0.007)	-0.01** (0.003)
lnI1.lnI3	-0.01 (0.067)	0.03 (0.040)	0.02 (0.037)	0.02* (0.010)
lnI1.lnI4	0.02* (0.010)	0.01 (0.005)	0.00 (0.005)	0.00*** (0.002)
lnI1.lnI5	0.00 (0.010)	0.00 (0.006)	0.00 (0.005)	0.00 (0.001)
lnI1.I6	0.00 (0.005)	0.01** (0.002)	0.00* (0.002)	0.00*** (0.000)
lnI2.lnI3	0.00 (0.029)	0.00 (0.019)	-0.01 (0.018)	-0.02** (0.009)
lnI2.lnI4	-0.01** (0.005)	0.00 (0.003)	-0.01** (0.003)	-0.01*** (0.002)
lnI2.lnI5	0.00 (0.003)	0.00 (0.002)	0.00 (0.002)	0.00 (0.001)
lnI2.I6	0.01* (0.003)	0.00 (0.001)	0.00 (0.001)	0.00 (0.001)
lnI3.lnI4	0.04* (0.024)	0.00 (0.014)	0.01 (0.012)	0.02*** (0.004)
lnI3.lnI5	0.00 (0.027)	0.00 (0.015)	0.00 (0.013)	0.00 (0.004)
lnI3.I6	0.01 (0.010)	0.00 (0.005)	0.00 (0.005)	0.00*** (0.001)
lnI4.lnI5	0.00 (0.004)	0.00 (0.002)	0.00 (0.002)	0.00*** (0.001)
lnI4.I6	0.00*** (0.001)	0.00 (0.001)	0.00** (0.001)	0.00*** (0.000)
lnI5.I6	0.00 (0.002)	0.00 (0.001)	0.00 (0.001)	0.00*** (0.000)
Constant	18.34* (9.502)	-2.37 (3.802)	3.50 (3.067)	4.35*** (1.080)
Source (base=GLSS7)				
GLSS1	-18.25* (9.560)	3.44 (3.814)	-2.67 (3.057)	-3.36*** (1.090)
GLSS2	-17.42** (8.807)	2.89 (3.754)	-2.85 (2.993)	-3.69*** (1.080)
GLSS3	-16.08** (6.831)	3.06 (3.627)	-2.64 (2.891)	-2.84*** (1.060)
GLSS4	-10.40** (4.217)	2.35 (3.285)	-2.20 (2.580)	-2.34** (0.972)
GLSS5	-4.93* (2.979)	1.25 (2.537)	-1.09 (2.022)	-1.56** (0.738)
GLSS6	-0.93 (1.655)	0.24 (1.139)	-0.41 (0.933)	-0.44 (0.337)
GSPS1	-2.92 (2.245)	0.51 (1.725)	-1.02 (1.402)	-1.50*** (0.541)
GSPS2	-1.04 (1.160)	0.01 (0.659)	-0.56 (0.557)	-0.52*** (0.194)
**Uncertainty function**				

Mean	-0.58*** (0.197)	-0.67*** (0.239)	-0.38*** (0.122)	-2.94*** (0.111)
Variance	0.75 (0.074)	0.71 (0.085)	0.83 (0.051)	0.23 (0.013)

Significance levels: * p<0.10, ** p<0.05, ***p<0.01; Data Sources: Ghana Living Standards Surveys [wave 1-7] and Ghana Socioeconomic Panel Survey [wave 1-2]

Table S6. Stochastic Meta-Frontier Results for Plantain Production in Ghana (1987-2017)

	**Transitional Zone**	**Forest Zone**	**Coastal Savanah**	**National**	**Meta-frontier**
**Production function**					
Land(ln[ha]) {lnI1}	0.35*** (0.106)	0.31*** (0.044)	0.24* (0.138)	0.32*** (0.038)	0.33*** (0.009)
Seed (ln[Mt]) {lnI2}	0.04 (0.062)	0.03 (0.023)	-0.06 (0.057)	0.03 (0.020)	0.03*** (0.005)
Hired labor(ln[days]) {lnI3}	0.00 (0.206)	0.07 (0.101)	-0.17 (0.292)	0.07 (0.088)	0.03 (0.025)
Family labor(ln[days]) {lnI4}	0.13*** (0.048)	0.13*** (0.019)	0.11* (0.066)	0.13*** (0.017)	0.13*** (0.004)
Pesticide (ln[liter]) {lnI5}	0.06 (0.055)	0.05*** (0.017)	0.16*** (0.049)	0.06*** (0.016)	0.06*** (0.005)
Trend {I6}	0.40* (0.214)	0.05 (0.079)	-0.13 (0.256)	0.09 (0.070)	0.17*** (0.022)
P5·lnI1·lnI1	-0.04 (0.033)	-0.01 (0.013)	-0.09** (0.038)	-0.01 (0.012)	-0.01*** (0.002)
P5·lnI2·lnI2	0.02** (0.009)	0.02*** (0.004)	-0.02 (0.012)	0.02*** (0.003)	0.02*** (0.001)
P5·lnI3·lnI3	0.13 (0.166)	-0.12 (0.084)	-0.24 (0.230)	-0.08 (0.072)	-0.04** (0.021)
P5·lnI4·lnI4	0.03*** (0.008)	0.02*** (0.003)	0.04*** (0.009)	0.02*** (0.003)	0.03*** (0.001)
P5·lnI5·lnI5	0.02** (0.010)	0.01*** (0.003)	0.04*** (0.011)	0.02*** (0.003)	0.01*** (0.001)
P5·I6·I6	0.02** (0.010)	0.01*** (0.003)	0.04*** (0.011)	0.02*** (0.003)	0.01*** (0.001)
lnI1.lnI2	0.01 (0.008)	0.01* (0.004)	0.00 (0.010)	0.01** (0.003)	0.01*** (0.001)
lnI1.lnI3	0.11** (0.050)	-0.01 (0.021)	0.00 (0.057)	0.01 (0.019)	0.02*** (0.005)
lnI1.lnI4	0.00 (0.008)	0.00 (0.003)	-0.02** (0.009)	0.00 (0.003)	0.00*** (0.001)
lnI1.lnI5	0.00 (0.008)	-0.01*** (0.003)	0.02** (0.009)	-0.01** (0.003)	-0.01*** (0.001)
lnI1.I6	0.01*** (0.003)	0.01*** (0.001)	0.01 (0.004)	0.01*** (0.001)	0.01*** (0.000)
lnI2.lnI3	0.01 (0.017)	-0.01 (0.008)	-0.01 (0.023)	-0.01 (0.007)	-0.01*** (0.002)
lnI2.lnI4	-0.01** (0.002)	0.00** (0.001)	0.00 (0.005)	0.00*** (0.001)	0.00*** (0.000)
lnI2.lnI5	0.00* (0.003)	0.00 (0.001)	0.00 (0.003)	0.00* (0.001)	0.00*** (0.000)
lnI2.I6	0.00** (0.002)	0.00*** (0.001)	0.00* (0.002)	0.00*** (0.000)	0.00*** (0.000)
lnI3.lnI4	0.02 (0.016)	0.00 (0.007)	0.02 (0.019)	0.01 (0.006)	0.01*** (0.001)
lnI3.lnI5	-0.01 (0.015)	0.01 (0.007)	-0.02 (0.018)	0.00 (0.006)	0.00* (0.002)
lnI3.I6	0.02*** (0.006)	0.00 (0.003)	0.01 (0.007)	0.00 (0.003)	0.01*** (0.001)
lnI4.lnI5	0.00 (0.002)	0.00 (0.001)	0.00 (0.003)	0.00 (0.001)	0.00 (0.000)
lnI4.I6	0.00 (0.001)	0.00 (0.000)	0.00* (0.001)	0.00 (0.000)	0.00*** (0.000)
lnI5.I6	0.00 (0.001)	0.00** (0.000)	0.00 (0.001)	0.00** (0.000)	0.00*** (0.000)
Constant	-1.32 (2.626)	-0.05 (1.141)	0.81 (3.506)	-0.20 (0.944)	-1.08** (0.483)
Source (base=GLSS7)					
GLSS1	2.27 (2.628)	0.46 (1.120)	-0.06 (3.494)	0.84 (0.936)	2.00*** (0.436)
GLSS2	2.01 (2.472)	0.38 (1.074)	-1.01 (3.270)	0.65 (0.893)	2.06*** (0.435)
GLSS3	0.55 (2.085)	-0.03 (0.954)	-0.14 (2.720)	0.10 (0.790)	0.87** (0.426)
GLSS4	-1.40 (1.515)	-0.06 (0.751)	-0.19 (1.787)	-0.21 (0.615)	0.12 (0.348)
GLSS5	-1.88* (1.107)	0.08 (0.558)	0.30 (1.111)	-0.13 (0.447)	-0.17 (0.132)
GLSS6	-0.94* (0.528)	-0.06 (0.258)	0.79 (0.514)	-0.11 (0.209)	-0.31*** (0.107)
GSPS1	-1.39* (0.781)	-0.10 (0.402)	0.68 (0.772)	-0.15 (0.323)	-0.39** (0.167)
GSPS2	-0.14 (0.365)	-0.06 (0.178)	-0.19 (0.409)	-0.07 (0.146)	-0.02 (0.059)

**Uncertainty function**					
Mean	-1.14*** (0.150)	-0.61*** (0.126)	-0.90*** (0.147)	-0.68*** (0.084)	-3.71*** (0.199)
Variance	0.56 (0.042)	0.74 (0.046)	0.64 (0.047)	0.71 (0.030)	0.16 (0.016)

Significance levels: * p<0.10, ** p<0.05, ***p<0.01; Data Sources: Ghana Living Standards Surveys [wave 1-7] and Ghana Socioeconomic Panel Survey [wave 1-2]

Table S7. Determinants of Starchy Staple Technical Inefficiency/ Technology Gap in Ghana (1987-2017)

	Female	Age (years)		Education (years)		Land owned	Extension	Credit	Mechanization
	(dummy)	Level	Squared	Level	Squared	(dummy)	(dummy)	(dummy)	(dummy)
Cassava									
Guinea Savanah	0.50* (0.263)	0.52 (0.316)	-0.82 (0.898)	0.13 (0.244)	0.01 (0.027)	-0.58*** (0.206)	0.26 (0.215)	0.80*** (0.250)	0.32 (0.452)
Transitional Zone	0.37*** (0.116)	0.12 (0.184)	1.06*** (0.372)	0.10 (0.130)	0.01 (0.015)	0.03 (0.114)	-0.26* (0.135)	0.45*** (0.167)	-
Forest Zone	0.16** (0.077)	0.24** (0.125)	0.40 (0.313)	0.05 (0.074)	0.00 (0.008)	-0.12* (0.069)	-0.10 (0.082)	0.00 (0.110)	0.36 (0.326)
Coastal Savanah	0.29* (0.152)	0.26 (0.222)	-0.08 (0.501)	-0.11 (0.171)	-0.01 (0.020)	0.14 (0.151)	-	0.24 (0.202)	-0.03 (0.380)
National	0.28*** (0.054)	0.28*** (0.085)	0.39* (0.206)	0.09* (0.055)	0.01 (0.006)	-0.11** (0.050)	-0.06 (0.060)	0.19*** (0.073)	0.12 (0.164)
Meta-frontier	0.15 (0.103)	0.01 (0.098)	0.02 (0.227)	0.07 (0.070)	0.01 (0.008)	0.06 (0.068)	-0.08 (0.106)	0.27*** (0.072)	0.42*** (0.122)
Yam									
Sudan Savanah	-1.94* (1.120)	-0.06 (0.562)	1.31 (1.263)	1.49 (0.910)	0.18* (0.110)	-2.22* (1.202)	-0.40 (1.185)	-0.27 (0.388)	-1.95** (0.964)
Guinea Savanah	0.33* (0.197)	-0.16 (0.161)	0.20 (0.305)	0.15 (0.135)	0.02 (0.016)	-0.16 (0.133)	0.03 (0.117)	0.22 (0.139)	-0.79** (0.401)
Transitional Zone	0.36** (0.151)	0.29 (0.194)	0.77* (0.443)	0.53*** (0.150)	0.06*** (0.018)	0.07 (0.131)	-0.20 (0.155)	0.41** (0.182)	-0.47 (0.544)
Forest Zone	0.01 (0.167)	0.15 (0.256)	-0.08 (0.690)	-0.11 (0.155)	-0.02 (0.019)	0.02 (0.164)	0.26 (0.201)	-0.11 (0.191)	-0.96* (0.493)
National	0.41*** (0.077)	0.38*** (0.098)	0.24 (0.223)	0.22*** (0.071)	0.02** (0.008)	-0.02 (0.069)	-0.15** (0.076)	0.19** (0.077)	-0.77*** (0.201)
Meta-frontier	0.22 (0.228)	1.20*** (0.321)	0.46 (0.781)	0.49 (0.329)	-0.57** (0.283)	0.23 (0.222)	-0.44* (0.252)	0.38** (0.189)	-293.03 (0.000)
Cocoyam									
Transitional Zone	0.44* (0.245)	-0.70 (0.429)	-0.12 (1.033)	-0.20 (0.222)	-0.02 (0.025)	0.92*** (0.338)	0.37 (0.378)	0.58* (0.328)	-16169.27 (0.000)
Forest Zone	0.10 (0.122)	0.34* (0.197)	0.38 (0.410)	0.13 (0.110)	0.01 (0.013)	-0.17 (0.109)	0.07 (0.119)	0.06 (0.138)	-0.39 (0.448)
National	0.27** (0.129)	0.25 (0.199)	0.53 (0.419)	0.09 (0.112)	0.01 (0.013)	0.00 (0.118)	0.06 (0.127)	0.11 (0.151)	-0.63 (0.658)
Meta-frontier	0.08 (0.142)	-0.14 (0.255)	0.63 (0.506)	0.27 (0.170)	0.03 (0.019)	-0.16 (0.124)	-0.19 (0.142)	-0.02 (0.173)	-0.55 (0.838)
Plantain									
Transitional Zone	-0.04 (0.162)	0.31 (0.246)	0.53 (0.582)	0.04 (0.157)	0.00 (0.018)	-0.24* (0.137)	-0.40** (0.192)	-0.06 (0.210)	-3.66 (8.746)
Forest Zone	-0.01 (0.080)	0.21* (0.121)	0.48 (0.305)	0.04 (0.080)	0.00 (0.009)	-0.23*** (0.085)	-0.13 (0.091)	0.02 (0.100)	-
Coastal Savanah	0.05 (0.282)	0.06 (0.364)	1.19 (1.084)	0.36 (0.270)	0.04 (0.032)	0.01 (0.249)	-0.21 (0.363)	-0.50 (0.418)	0.55 (1.033)
National	-0.02 (0.067)	0.20** (0.100)	0.39 (0.244)	0.06 (0.065)	0.01 (0.007)	-0.22*** (0.064)	-0.14* (0.076)	-0.03 (0.081)	-0.40 (0.355)
Meta-frontier	-0.08 (0.108)	0.06 (0.128)	-0.32 (0.289)	0.03 (0.096)	0.00 (0.011)	-0.07 (0.157)	-0.39 (0.244)	-0.17 (0.142)	1.04** (0.429)

Significance levels: * p<0.10, ** p<0.05, ***p<0.01

Data Sources: Ghana Living Standards Surveys [wave 1-7] and Ghana Socioeconomic Panel Survey [wave 1-2

Table S8: Ghanaian Starchy Staple Production Technology Level and Technical Efficiency Parameters Across Seasons (1987-2017)

Season	TGR	TE	MTE	GAP	TGR	TE	MTE	GAP
	Cassava				Yam			
1987/88	0.758	0.455	0.344	24.19%	1.000	0.401	0.402	0.00%
1988/89	0.998	0.494	0.495	0.21%	0.994	0.571	0.572	0.56%
1990/91	0.994	0.790	0.788	0.56%	0.935	0.466	0.443	6.52%
1991/92	0.994	0.798	0.795	0.57%	0.938	0.450	0.432	6.23%
1997/98	0.991	0.697	0.694	0.86%	0.930	0.808	0.718	6.99%
1998/99	0.991	0.691	0.683	0.93%	0.948	0.659	0.607	5.25%
2004/05	0.976	0.735	0.716	2.40%	0.892	0.563	0.502	10.84%
2005/06	0.978	0.738	0.724	2.21%	0.897	0.568	0.507	10.27%
2009/10	0.729	0.749	0.536	27.08%	0.966	0.535	0.518	3.38%
2011/12	0.777	0.629	0.479	22.28%	0.855	0.512	0.439	14.55%
2012/13	0.798	0.629	0.495	20.20%	0.847	0.490	0.408	15.27%
2014/15	1.000	0.580	0.579	0.00%	0.719	0.428	0.319	28.12%
2015/16	0.899	0.445	0.401	10.07%	1.000	0.496	0.491	0.00%
2016/17	0.900	0.458	0.413	9.97%	1.000	0.550	0.540	0.00%

Season	Cocoyam				Plantain			
1987/88	0.981	0.565	0.559	1.94%	0.730	0.411	0.303	26.99%
1988/89	1.000	0.533	0.537	0.00%	0.537	0.405	0.211	46.31%
1990/91	0.652	0.496	0.320	34.76%	0.863	0.712	0.613	13.75%
1991/92	0.575	0.642	0.298	42.54%	0.842	0.681	0.572	15.82%
1997/98	0.783	0.646	0.492	21.73%	0.857	0.670	0.568	14.25%
1998/99	0.816	0.639	0.500	18.43%	0.844	0.680	0.573	15.56%
2004/05	0.784	0.412	0.333	21.61%	0.903	0.665	0.599	9.69%
2005/06	0.796	0.411	0.320	20.36%	0.915	0.629	0.573	8.45%
2009/10	1.000	0.563	0.557	0.00%	0.994	0.737	0.733	0.56%
2011/12	0.667	0.435	0.292	33.32%	1.000	0.591	0.582	0.00%
2012/13	0.679	0.418	0.286	32.15%	1.000	0.580	0.575	0.00%
2014/15	0.831	0.561	0.459	16.87%	0.782	0.548	0.424	21.85%
2015/16	0.748	0.096	0.066	25.18%	0.834	0.442	0.360	16.55%
2016/17	0.826	0.271	0.221	17.36%	0.855	0.454	0.385	14.54%

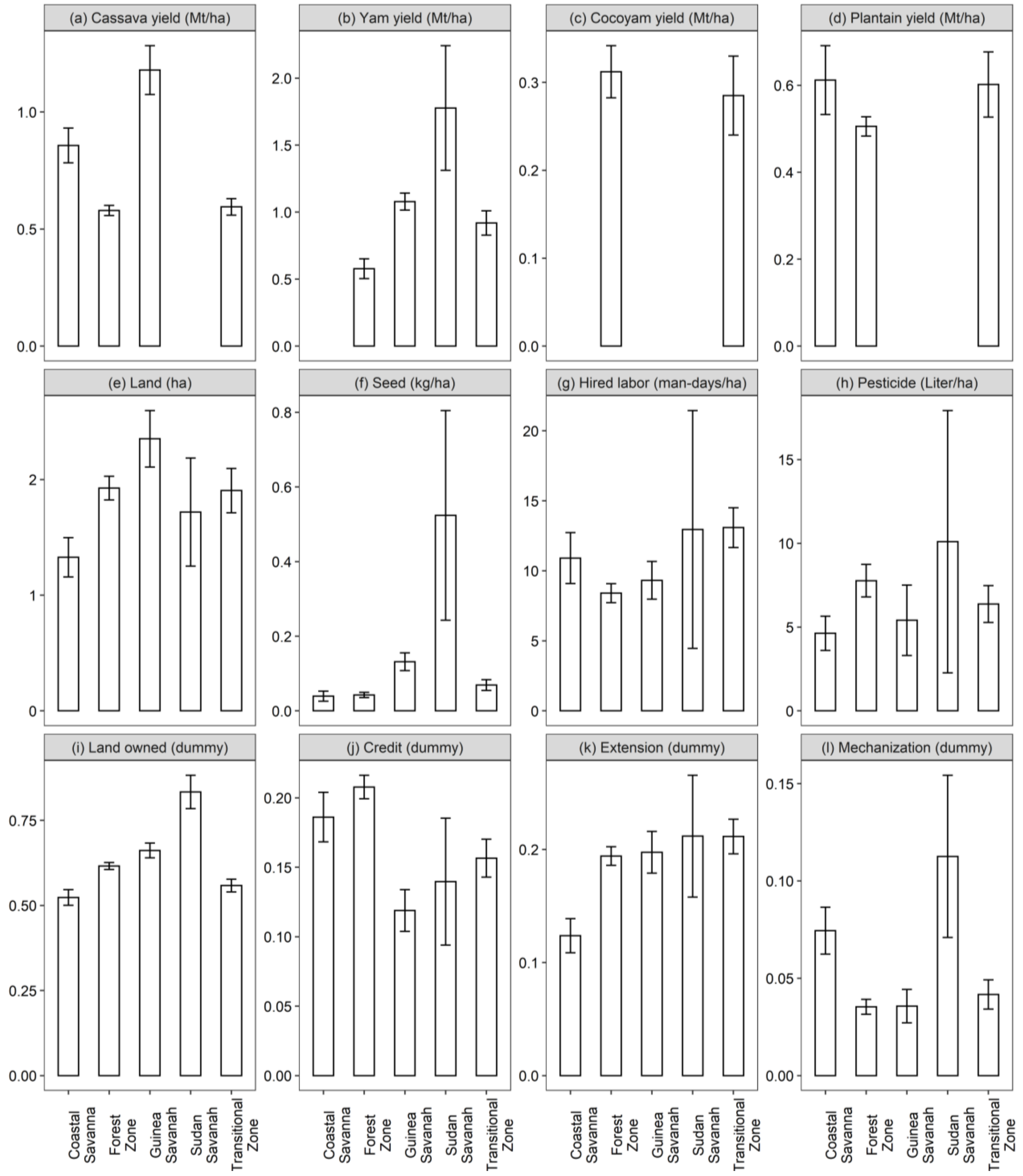

Figure S1a: Ecological variation in Starchy Staple production yield, input, and enabling environment factors in Ghana (1987-2017)

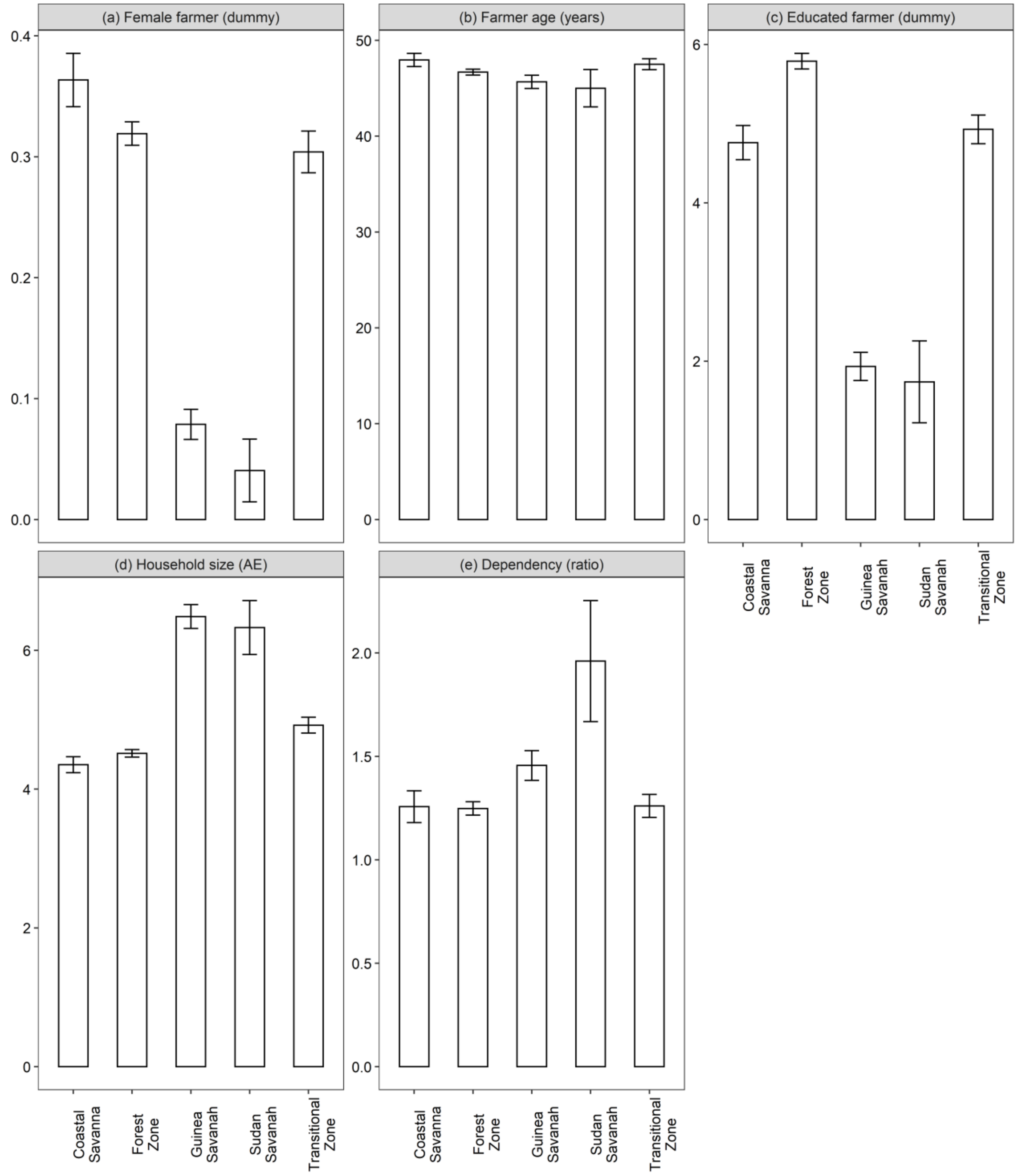

Figure S1b: Ecological variation in Starchy Staple Farmer and Household Demographics factors in Ghana (1987-2017)

Note S1. Construction of variables

Female (dummy): Across all surveys, this variable takes on a value of unity if the actual farmer is female, zero otherwise.

Age (years): Across all surveys, this variable was taken as the recorded age of the farmer whose production information was analyzed.

Education (years): Across all surveys, this variable was taken as the recorded years of education of the farmer whose production information was analyzed.

Land owned (dummy): Across all surveys, this variable takes on a value of unity if the actual farmer-owned portions of the land under cultivation.

Land (ha): Across all surveys, this variable is taken as the farmer recalled land under production, converted to hectares.

Yield (kg/ha): Across all surveys, this value was taken as the quantity of harvest divided by the land under cultivation of a given legume.

Family labor (AE): Across all surveys, family labor was calculated as the total adult equivalence (AE) attributable to members aged 15 years and older.

Hired labor (man-days): For GSPS, input usage was reported at the plot level, thus the quantity of hired labor was taken as the farmer recalled quantity of paid labor in man-days, multiplied by the ratio of production value to total production value for a given plot. These were then summed for a given farmer and legume. Information on inputs in all seven GLSS's were collected at the household as expenditures. Consequently, for each farmer, the expenditure for hired labor was calculated as the respective share of that farmer's crop production value in total household crop harvest value; subsequently, these were divided by the annual minimum agricultural daily wage to approximate the man-days of hired labor used.

Pesticide (Liter): Same as hired labor, the only exception is that, where necessary, the annual price for fertilizer (GHC/kg) was used to convert pesticide expenditures to liters.

Household Size (AE): Across all surveys, household size in adult male equivalence (AE) was computed by dividing the total energy requirements of the household by 2,250 kcal.

Dependency (ratio): Given the AE of its members, the dependency ratio of the household was then calculated as the total AE attributable to members aged between 17 and 70 years divided by that for those aged below 18 years or above 69 years.

Credit (dummy): Across all surveys, this variable takes on a value of unity if at least one household member applied for a loan or received inputs on credit, zero if not, and two if no credit information was collected in the survey. A value of two was assigned to data drawn from the GLSS1, GLSS2, GLSS3, and GLSS4 surveys since they were never collected. In this case, the coefficient on category one is taken as the credit effect in any econometric estimation.

The extension (dummy): Across all surveys, this variable takes on a value of unity if the actual farmer is in an enumeration area (as defined by the survey) in which at least one farmer reported having received extension services from MoFA or NGOs. Also, where community information was collected, this variable takes on a value of unity if there was an extension agent or office in that community. This variable takes on a value of zero if the above conditions are not met.

Note S2. Test for Model Specification

***Skewness test*:** The Coelli (1995) and Schmidt and Lin (1984) skewness test for OLS residuals are negative for all the models, suggesting that the production variation of starchy staples across Ghana's ecologies are negatively skewed. The Gutierrez et. al. (2001) test confirmed the conclusions of the Schmidt and Lin (1984) and Coelli (1995) tests. The outcome of these tests indicates that the negative skewed error needed for the justification of SFA is strong. Furthermore, the null hypotheses $H_o\colon \boldsymbol{\alpha} = 0$ was also rejected, thus, providing further justification for the technical inefficiency functions.

***The similarity of production frontiers across ecologies*:** To statistically check the similarities of the production frontiers for a given starchy staple across the different ecologies, the study applied a generalized likelihood ratio test. The log-likelihood values under the null hypothesis of a uniform technology were all smaller than that of the alternate hypothesis of heterogeneous technology. The differences in the log-likelihood values led to test statistics that soundly rejected the null hypothesis, and thus, support the fact that starchy staple farmers are operating under heterogeneous technologies along ecological lines.

Production variance: The proportion of production variance due to inefficiency (γ) ranged from 0.20 to 0.99 for the ecology frontiers; for a given crop, this was always higher than that of the Meta-frontier. The γ values suggest that a considerable amount of the observed variation in output for the ecology-frontiers [Meta-frontier] could be attributed to inefficient use of inputs [technological gaps]. The range of γ for the Meta-frontier also suggests that the observed variation in output, given the ecology-frontiers, could not be attributed to idiosyncrasies. Finally, the SFA model chi-squared test statistics indicate that the models are all significant. In Table C1, we report the summary of estimates for the ecology- and meta-frontier production elasticities for the starchy staples and discuss the details in the following subsections along crop-specific lines.

Note S3. Drivers of Technical Inefficiency and Technology gap

In assessing the factors that influence technical efficiency and technology gaps of starchy staples in Ghana, the key variables considered were farmer characteristics (age, education, and gender) and institutional variables (land ownership, extension service access, credit access, and mechanization service utilization). Table D1 reports the estimates across crops and ecological zones. Results for the case of technical inefficiency are captured by the ecology frontiers and that of the technology gap is captured by the meta-frontier. With TE, negative [positive] values indicate that the variables reduce [increase] technical inefficiency. For the case of TGR, negative [positive] values indicate that the variables reduce [increase] the technology gap.

The following caveats are to be noted in interpreting the effects of these variables on technical inefficiency and technological gaps. First, these variables were harmonized across different surveys and used mainly as controls in estimating the drivers of differences in starchy staples production outcomes in Ghana. Due to this level of aggregation, we are not confident that there is enough exogenous variation in the control variables to attach real causal interpretation to them. Notwithstanding, given this study's focus of establishing which of technological gaps or technical inefficiency contributes the most to production shortfalls, we believe the outcomes of these farmer characteristics and institutional factors can help to direct policies. To this end, we interpret these variables in terms of general associations and not in terms of individual coefficients.

First, the results show that females are doubly disadvantaged in the production of starchy staples as they are both less technically efficient and have higher technology gaps when compared to their male counterparts. These phenomena are well established by similar findings in the literature for rice (Owusu, Donkor, and Owusu-Sekyere, 2018), yam (Owusu Asante, Villano, and Battese, 2014), and vegetables (Tsiboe, Asravor, and Osei, 2019).

Turning to the age of the farmer, the results show that age has generally positive level and long-run associations with farmers' TE and technological gaps. Thus, older farmers tend to be more inefficient in starchy staples production and express greater gaps in adopting modern technologies. Owusu Asante et al. (2014) found similar effects of age on yam farmers' technical inefficiency in Ghana's Ashanti and Brong-Ahafo regions. Similarly, farmers' years of education showed mostly negative short- and long-run associations with technical efficiency and adoption of superior technologies.

Where statistically significant, general results for land ownership shows that farm households that own lands are more technically efficient than non-owners of land. This highlights the positive effect of tenure security: we would expect landowners to be more motivated to undertake management practices that improve soil fertility and land structure, and that eventually lead to more efficient crop production than non-owners of land (Besseah and Kim, 2014). On the other hand, the effect of ownership on bridging technology gaps appears inconclusive. On its own, land ownership may not be enough incentive to adopt and maintain the application of modern technologies. Modern production technologies may require significant extra labour and capital investment to apply. In such instances, resource-constrained farmers may rather opt for conservative land management techniques that improve technical efficiency. Access to agricultural extension services often plays a key role in helping farmers improve their crop production by promoting good agronomic practices and the adoption of better technology that improve

efficiency. Our results confirm this a priori expectation by their generally negative associations with TE and TGR. As in this study, Owusu Asante et al. (2014) also found that extension access reduced production inefficiency.

Access to credit is expected to enable farmers to acquire supporting inputs necessary to increase efficiency and productivity. The results in Table D1 show generally positive associations with both TE and TGR. This implies that farmers with access to credit are not only less efficient, but also less likely to apply the best production technologies relative to their credit-rationed counterparts. The counterintuitive finding that credit decreases TE and increases TGR is difficult to explain from the available data, but one possible prediction could relate to the exact use of the credit accessed. Do farmers invest the credit funds in their starchy staple production? Oftentimes, farmers use the credit for other purposes than farm investment (Appiah-Twumasi, Donkoh, and Ansah, 2019). For mechanization, the results show that access correlates directly to reduced technical inefficiency but inversely with TGR. This mixed result is not surprising and may be reflective of the heterogenous access to mechanized services across production ecologies and among farmers in Ghana (Ministry of Food and Agriculture [MoFA], 2019). That said, we are mindful of claiming too much inference to this finding given the conditions.

References

Appiah-Twumasi, M., S. A. Donkoh, and I. G. K. Ansah. ‘Farmer innovations in financing smallholder maize production in Northern Ghana’. Agricultural Finance Review 80,3(December 2019):421–436.

Coelli, T. ‘Estimators and hypothesis tests for a stochastic frontier function: A Monte Carlo analysis’. Journal of Productivity Analysis 6,3(September 1995):247–268.

Gutierrez, R. G., S. Carter, and D. M. Drukker. ‘On boundary-value likelihood-ratio tests’. Stata Technical Bulletin 10,60(2001).

Ministry of Food and Agriculture [MOFA]. ‘Agriculture in Ghana: Facts and figures (2018)’. Document Provided by Ministry of Agriculture, Accra, Ghana. Accra,Ghana.

Owusu Asante, B., R. A. Villano, and G. E. Battese. The effect of the adoption of yam minisett technology on the technical efficiency of yam farmers in the forest-savanna transition zone of Ghana. African Journal of Agricultural and Resource Economics. 2014.

Owusu, V., E. Donkor, and E. Owusu-Sekyere. ‘Accounting for the Gender Technology Gap Amongst Smallholder Rice Farmers in Northern Ghana’. Journal of Agricultural Economics 69,2(June 2018):439–457.

Schmidt, P., and T.-F. Lin. ‘Simple tests of alternative specifications in stochastic frontier models’. Journal of Econometrics 24,3(March 1984):349–361.

Tsiboe, F., J. Asravor, and E. Osei. ‘Vegetable Production Technical Efficiency and Technology Gaps in Ghana’. African Journal of Agricultural and Resource Economics 14,4(2019).
