# Supplementary material for: Starchy staples production shortfalls in Ghana: Technical inefficiency effects outweigh technological differences across ecologies
Source: PLoS One. 2023 Apr 24;18(4):e0284780. doi: 10.1371/journal.pone.0284780 (PMC10124864; doi:10.1371/journal.pone.0284780)
Supplement: Attachment — Submitted filename: PONE-D-22-23407_R2.pdf [file pone.0284780.s007.pdf]

PLOS ONE

Starchy staples production shortfalls in Ghana: Technical inefficiency effects outweigh technological differences across ecologies

--Manuscript Draft--

**Manuscript Number:**	PONE-D-22-23407R2
**Article Type:**	Research Article
**Full Title:**	Starchy staples production shortfalls in Ghana: Technical inefficiency effects outweigh technological differences across ecologies
**Short Title:**	Starchy staples production shortfalls in Ghana
**Corresponding Author:**	Isaac Gershon Kodwo Ansah, Ph.D<br>University for Development Studies<br>Tamale, Northern Region GHANA
**Keywords:**	starchy staples; technological gap ratio; technical inefficiency; Ghana
**Abstract:**	Starchy staples are a major source of livelihood support for farmers, traders, and processors who participate in these crops' value chains, while also providing staple food to many people, especially the less affluent in society. Despite this position, the productivity figures of starchy staples are low. We use a unique data set and meta-frontier efficiency analysis to assess whether the production shortfalls of major starchy staple crops in Ghana could be attributed to technical inefficiency, technology gaps or both. Results show strong evidence of about 50% production shortfall for cassava, yam, cocoyam, and plantain. For cassava production, the Guinea Savannah zone has the most superior technology, with a technology gap ratio of 0.92, while yam production is more technically efficient in the Sudan Savannah zone, with a technical efficiency score of 0.67. Cocoyam production is more technically efficient (0.56) in the Transition zone, but yam is more technically efficiently produced in the Coastal Savannah zone of Ghana. These results show that production shortfall is more influenced by pure farmer technical inefficiencies (about 45%) rather than by technology gaps (about 20%) along ecological lines. Thus, the sector could benefit from improvements in farmer managerial skills and efficient use of existing technologies.
**Order of Authors:**	Isaac Gershon Kodwo Ansah, Ph.D
	Mark Appiah-Twumasi, MD
	Francis Tsiboe, Ph.D
**Response to Reviewers:**	Please, w have provided a response file as an attachment to the submission.<br>Thank you.
**Additional Information:**	
**Question**	**Response**
**Financial Disclosure**<br><br>Enter a financial disclosure statement that describes the sources of funding for the work included in this submission. Review the submission guidelines for detailed requirements. View published research articles from *PLOS ONE* for specific examples.<br><br>This statement is required for submission and **will appear in the published article** if the submission is accepted. Please make	The authors received no specific funding for this work.

Powered by Editorial Manager® and ProduXion Manager® from Aries Systems Corporation

sure it is accurate.<br><br>**Unfunded studies**<br>Enter: *The author(s) received no specific funding for this work.*<br><br>**Funded studies**<br>Enter a statement with the following details:<br>• Initials of the authors who received each award<br>• Grant numbers awarded to each author<br>• The full name of each funder<br>• URL of each funder website<br>• Did the sponsors or funders play any role in the study design, data collection and analysis, decision to publish, or preparation of the manuscript?<br>• **NO** - Include this sentence at the end of your statement: *The funders had no role in study design, data collection and analysis, decision to publish, or preparation of the manuscript.*<br>• **YES** - Specify the role(s) played.<br><br>* typeset	
**Competing Interests**<br><br>Use the instructions below to enter a competing interest statement for this submission. On behalf of all authors, disclose any competing interests that could be perceived to bias this work—acknowledging all financial support and any other relevant financial or non-financial competing interests.<br><br>This statement is required for submission and **will appear in the published article** if the submission is accepted. Please make sure it is accurate and that any funding sources listed in your Funding Information later in the submission form are also declared in your Financial Disclosure statement.<br><br>View published research articles from *PLOS ONE* for specific examples.	The authors have declared that no competing interests exist.

**NO authors have competing interests**<br><br>Enter: *The authors have declared that no competing interests exist.*<br><br>**Authors with competing interests**<br><br>Enter competing interest details beginning with this statement:<br><br>*I have read the journal's policy and the authors of this manuscript have the following competing interests: [insert competing interests here]*<br><br>* typeset	
**Ethics Statement**<br><br>Enter an ethics statement for this submission. This statement is required if the study involved:<br><br>• Human participants<br>• Human specimens or tissue<br>• Vertebrate animals or cephalopods<br>• Vertebrate embryos or tissues<br>• Field research<br><br>Write "N/A" if the submission does not require an ethics statement.<br><br>General guidance is provided below. Consult the submission guidelines for detailed instructions. **Make sure that all information entered here is included in the Methods section of the manuscript.**	N/A

Powered by Editorial Manager® and ProduXion Manager® from Aries Systems Corporation

**Format for specific study types**<br><br>**Human Subject Research (involving human participants and/or tissue)**<br>• Give the name of the institutional review board or ethics committee that approved the study<br>• Include the approval number and/or a statement indicating approval of this research<br>• Indicate the form of consent obtained (written/oral) or the reason that consent was not obtained (e.g. the data were analyzed anonymously)<br><br>**Animal Research (involving vertebrate animals, embryos or tissues)**<br>• Provide the name of the Institutional Animal Care and Use Committee (IACUC) or other relevant ethics board that reviewed the study protocol, and indicate whether they approved this research or granted a formal waiver of ethical approval<br>• Include an approval number if one was obtained<br>• If the study involved *non-human primates*, add *additional details* about animal welfare and steps taken to ameliorate suffering<br>• If anesthesia, euthanasia, or any kind of animal sacrifice is part of the study, include briefly which substances and/or methods were applied<br><br>**Field Research**<br><br>Include the following details if this study involves the collection of plant, animal, or other materials from a natural setting:<br>• Field permit number<br>• Name of the institution or relevant body that granted permission	
**Data Availability**<br><br>Authors are required to make all data underlying the findings described fully available, without restriction, and from the time of publication. PLOS allows rare exceptions to address legal and ethical concerns. See the PLOS Data Policy and FAQ for detailed information.	No - some restrictions will apply

A Data Availability Statement describing where the data can be found is required at submission. Your answers to this question constitute the Data Availability Statement and **will be published in the article**, if accepted.<br><br>**Important:** Stating 'data available on request from the author' is not sufficient. If your data are only available upon request, select 'No' for the first question and explain your exceptional situation in the text box.<br><br>Do the authors confirm that all data underlying the findings described in their manuscript are fully available without restriction?	
**Describe where the data may be found in full sentences. If you are copying our sample text, replace any instances of XXX with the appropriate details.**<br><br>• If the data are **held or will be held in a public repository**, include URLs, accession numbers or DOIs. If this information will only be available after acceptance, indicate this by ticking the box below. For example: *All XXX files are available from the XXX database (accession number(s) XXX, XXX.*).<br>• If the data are all contained **within the manuscript and/or Supporting Information files**, enter the following: *All relevant data are within the manuscript and its Supporting Information files.*<br>• If neither of these applies but you are able to provide **details of access elsewhere**, with or without limitations, please do so. For example:<br><br>*Data cannot be shared publicly because of [XXX]. Data are available from the XXX Institutional Data Access / Ethics Committee (contact via XXX) for researchers who meet the criteria for access to confidential data.*<br><br>*The data underlying the results presented in the study are available from (include the name of the third party*	The data underlying the results presented in the study are available from Ghana Statistical Service and can be accessed freely online via https://www.statsghana.gov.gh/gssdatadownloadspage.php.<br><br>Replication materials are available on GitHub at https://github.com/ftsiboe/Agricultural-Productivity-in-Ghana

and contact information or URL).<br>• This text is appropriate if the data are owned by a third party and authors do not have permission to share the data.<br><br>* typeset	
Additional data availability information:	Tick here if the URLs/accession numbers/DOIs will be available only after acceptance of the manuscript for publication so that we can ensure their inclusion before publication.

Powered by Editorial Manager® and ProduXion Manager® from Aries Systems Corporation

Manuscript

Click here to access/download;Manuscript;v03_Roots_TE_GH_MT_FT - PloS

Starchy staples production shortfalls in Ghana: Technical inefficiency effects outweigh technological differences across ecologies

Isaac Gershon Kodwo Ansah [a*], Mark Appiah-Twumasi[b], Francis Tsiboe[c]

[a] Department of Economics, School of Economics, University for Development Studies, Tamale, Ghana.

[b] Department of Agricultural and Food Economics, Faculty of Agriculture, Food and Consumer Sciences, University for Development Studies, Tamale, Ghana.

[c] USDA, Economic Research Service, 805 Pennsylvania Avenue, Kansas City, MO 64105, USA

* Corresponding author at: Department of Economics, School of Economics, University for Development Studies, Tamale, Ghana. E-mail address: agershon@uds.edu.gh (Isaac Gershon Kodwo Ansah) (ORCID ID: 0000-0001-5071-6224)

Data Availability: Replication materials are available on GitHub at https://github.com/ftsiboe/Agricultural-Productivity-in-Ghana

Funding: The authors did not receive any funding for this manuscript.

Acknowledgments:

The authors gratefully acknowledge the Ghana Statistical Service, Institute of Statistical Social and Economic Research (ISSER), Economic Growth Center, and the International Food Policy Research Institute (IFPRI) for making the dataset available for the study. All errors are the responsibility of the authors. The findings and conclusions in this publication are those of the authors and should not be construed to represent any official USDA or U.S. Government determination or policy. This research was supported in part by the U.S. Department of Agriculture, Economic Research Service.

Abstract

Starchy staples are a major source of livelihood support for farmers, traders, and processors who participate in these crops' value chains, while also providing staple food to many people, especially the less affluent in society. Despite this position, the productivity figures of starchy staples are low. We use a unique data set and meta-frontier efficiency analysis to assess whether the production shortfalls of major starchy staple crops in Ghana could be attributed to technical inefficiency, technology gaps or both. Results show strong evidence of about 50% production shortfall for cassava, yam, cocoyam, and plantain. For cassava production, the Guinea Savannah zone has the most superior technology, with a technology gap ratio of 0.92, while yam production is more technically efficient in the Sudan Savannah zone, with a technical efficiency score of 0.67. Cocoyam production is more technically efficient (0.56) in the Transition zone, but yam is more technically efficiently produced in the Coastal Savannah zone of Ghana. These results show that production shortfall is more influenced by pure farmer technical inefficiencies (about 45%) rather than by technology gaps (about 20%) along ecological lines. Thus, the sector could benefit from improvements in farmer managerial skills and efficient use of existing technologies.

Keywords: starchy staples; technological gap ratio; technical inefficiency; Ghana

JEL codes: D24, Q18, Q12.

Starchy staples production shortfalls in Ghana: Technical inefficiency effects outweigh technological differences across ecologies

1. Introduction

In 2022, the Food and Agriculture Organization's document on the State of Food Security and Nutrition in the World projected that between 702 and 828 million people faced hunger in 2021, with Africa and Asia being the main hosts of these challenges [1]. In Ghana, a report by GSS/MOFA/WFP/FAO [2] indicate that 11.7% of Ghana's population (3.6 million people) were food insecure in 2020. Out of these, 5.2% were severely food insecure, and 6.5% were moderately food insecure; also, 78% of the food insecure were in rural areas. The report further notes that there are regional heterogeneities in the distribution of food insecurity in Ghana, with most food insecure people living in the Guinea Savannah and Deciduous Forest zones. In explaining the possible causes of these rising food insecurity challenges, the FAO [1] report identified poor access to food due to low domestic production, poor distribution and high cost of imports, among other factors. Since the primary source of food in these areas, Africa especially, is domestic production, this creates the urgent need to examine the pathways through which food is made available and accessible locally to the vulnerable segment of the population.

In many parts of the developing world, smallholder farmers mostly organize the production of staples. The 2022 FAO report on "The State of Food and Agriculture" point to low agricultural productivity as characterizing smallholder production systems [3]. Based on global projections, Pradhan et al. [4] conclude that Africa has the highest crops yield gaps, and that closing these gaps has the potential to improve food and nutrition security in Africa. Thus, productivity growth and resource use efficiency are important causal pathways to increased food production and access for the poor in these areas. Notably, Pradhan et al. [4] attribute the wide yield gaps in crops, especially

those cultivated under rainfed, to biophysical factors (e.g., soil quality, agro-climatic, etc.) and socioeconomic factors (e.g., weather-induced yield variability that affect risk attitudes, market access, etc.). Besides direct food availability, agro-industrial development rests so much on the availability of raw materials to supply industrial needs. How could the industrial need be met if what is available cannot satisfy direct consumption demand? Therefore, productivity growth in these crops is crucial to foster rapid economic transformation in the developing world.

The production of cassava (*Manihot esculenta*), cocoyam (*Colocasia esculenta*), plantain (*Musa sapientum*) and yam (*Dioscorea alata*) (henceforth called starchy staples) plays important roles in the Ghanaian economy. Starchy staples are a major source of livelihood support for farmers, traders, and processors who participate in these crops' value chains, while also providing staple food to many people, especially the less affluent in society. Despite this position, the productivity figures of starchy staples are low. There are non-ignorable productivity gaps between actual and potential yields. In 2018 the national average yields of cassava, cocoyam, plantain, and yam were all over 50% lower than their potential yields, which were estimated at 45Mt/ha for cassava, 38Mt/ha for plantain, 52Mt/ha for yam, and 20Mt/ha for cocoyam [5].

These yield gaps ultimately culminate in production shortfalls which tend to vary widely across regions and ecological zones in Ghana. Such variations indicate that the production shortfalls might be location-specific, crop-specific, and farmer-specific. The large production shortfalls generate costs to the country in terms of wasted resources amid scarcity as well as creating environmental challenges. These production shortfalls, therefore, border on the efficiency of resource utilization (e.g., Ladha et al., [6]) which would require attention. Resource use efficiency, in the layman’s understanding, means *doing more with less*. A transition from doing less with more (inefficiency of resource utilization) to doing more with less (efficiency of resource utilization)

allows the creation of more economic value with less natural, human, and man-made resources, and eventually enhances household welfare. How can this be achieved if we have limited knowledge of what drives these production shortfalls, or their nature and distribution thereof?

Several studies relevant to Ghanaian agriculture have investigated the primary drivers of resource use efficiency for rice [7–12], maize [12–15], legumes [16–18], cocoa [19–22], and vegetables [23], but with little attention on production shortfalls. An important category of crops that has received less attention despite the important role they play in the food security of households is the starchy staples. A few studies like [24] analyzed starchy staples data for only one season, paying no attention to the possibility of heterogeneous technology adoption within Ghana. Like their peers cultivating vegetables [23], cereals [11,12,14], and legumes [25], starchy staples farmers could be operating under different technologies constrained by the ecology and/or enabling environment. According to the literature, failure to account for these technological differences could lead to falsely attributing production shortfalls due to technology gaps to technical inefficiency [26].

In the last two decades, research on productivity and efficiency analyses in Ghana is highly skewed toward cereal crops. In principle, the starchy staples have received little attention, against the backdrop of the critical roles these crops play, and the significant yield gaps observed in practice. Except for a few studies [12,22,23,25,27], all efficiency studies in Ghana used data based on a single season, and data size is limited to a few villages or at best a region. This data limitation and lack of locational heterogeneity masks important lessons that could emerge from analyzing a more comprehensive and heterogeneous sample. For example, knowledge on whether technology gaps across ecologies or resource use inefficiencies among farmers contribute most to production shortfalls and how these evolve is not yet clear. Such incomplete information might limit scientific

progress, and serve as a barrier to societal development, in the sense that policymakers may be inadequately or wrongly informed. Focusing on starchy staples, this study addresses most of these limitations by accommodating multiple seasons and heterogeneity in ecological technology gaps.

Particularly, this study applies Huang et al. [28] method of estimating meta-stochastic-frontier to a sample of 15,420 starchy staple farmers drawn from nine cross-sectional population-based surveys periodically fielded throughout Ghana from 1987 to 2017. The aim is to examine whether production shortfalls are primarily driven by resource use efficiency among farmers or due to technological gaps across ecological zones in Ghana, as well as their nature and distribution thereof. The pooled data has the broadest coverage of starchy staple households across time and space than previous studies conducted on Ghana. As such, it presents a unique opportunity for utilizing the meta-stochastic frontier to ascertain the ecological and temporal dynamics of starchy staple resource use efficiency in Ghana.

Our results show significant production shortfalls in starchy staples in Ghana, which could be attributed more to pure farmer technical inefficiencies rather than technology gaps. The production technologies used by starchy staple farmers are quite similar across ecologies, but some ecological zones perform better than others. The chronic pure farmer technical inefficiency effects suggest that the production shortfalls could be reduced by addressing the causes of inefficiency and improving the efficient use of existing technologies. These results are important because the achievement of Sustainable Development Goals 2.1 (ensuring access to food for all) and 2.2 (ending all forms of malnutrition) would, at least partly, require the realization of Sustainable Development Goal 12, which leverages the efficient use of natural resources to create a circular economic growth [29].

The rest of the paper is structured as follows. The next section presents an overview of the study area and data. In Section 3, we discuss how the Meta-Stochastic Frontier is used to assess shortfalls in starchy staple production in Ghana. Section 4 presents the results while Section 5 discusses the findings. We present concluding remarks in Section 6.

2. Research area and data

2.1 Study area

Ghana covers a total land area of 24 million hectares of which 20.7% is considered arable, 40.7% under forests, 11.8% under permanent crops, and 26.8% for other uses. Ghana's ecology is classified as Rain Forest, Semi-Deciduous Forest, Coastal Savannah, Transition, Guinea Savannah, and Sudan Savannah zones. The aridity of these ecologies increases from south to north. Farming systems across these ecologies are highly heterogeneous. However, the highest-yielding ecologies across all the starchy staples are Semi-Deciduous Forest and Transition zones, and the lowest-yielding ones are Sudan Savannah and Rain-Forest zones. Given their well-balanced annual rainfall and modest temperatures, Semi-Deciduous Forest and Transition zones have the optimal conditions to grow starchy staples. Thus, it is not surprising that they also contain most of the cultivated lands allocated to starchy staples as shown in Tables S1 and S2 (all tables, figures, and notes with a leading S indicate supplemental materials in the online appendix).

2.2 Data sources

The study uses household level data drawn from two sources: (1) the two waves of the Ghana Socioeconomic Panel Survey (GSPS) fielded in 2009/10 and 2014/15 [30]; and (2) all seven Ghana Living Standards Surveys (GLSS) fielded between 1987-2017 and publicly available at the Ghana Statistical Service (GSS) National Data Archive. The surveys were based on nationally

representative samples, focusing on the household as the key socio-economic unit, and provides insights into Ghana's living conditions. The GSPS was collected by the Economic Growth Center (EGC) at Yale University and the Institute of Statistical, Social, and Economic Research (ISSER), and the GLSS by GSS. In terms of the raw data collection, both surveys followed a two-stage stratified sampling design, where enumeration areas and households were selected in the first and second stages, respectively. The harmonization details of these datasets to construct farmer-level data are published in Tsiboe [31]. It is worth noting that while the GSPS constitutes a panel, households are resampled for each GLSS round, hence repeated cross-sectional data. Consequently, since a large proportion of the data is from the GLSS (84%), the harmonized data can be viewed as a cross-sectional sample of the population of Ghanaian starchy staple farmers at roughly five-year intervals.

The final sample used in this study was limited to starchy staple farmers drawn from the various surveys, with yield (kg/ha) above the 5th and below the 90th percentiles by survey, crop, and region. The final sample, therefore, consisted of 15,405 farmers from 15,133 households, out of which 11,585, 3,468, 1,834, and 6,226 farmers cultivate cassava, yam, cocoyam, and plantain, respectively. All of Ghana's ecologies are well represented in the final sample, and the dataset captures 14 growing seasons. Nonetheless, due to sparse data, the Rain Forest and Semi-Deciduous Forest zones were combined into the Forest ecological zone. Note S1 in the online appendix documents the definition of all the variables used in this study.

2.3 Descriptive statistics

Table 1 provides the descriptive statistics of the household level characteristics, showing their temporal, ecological, and crop variation. Except for land and yield, average values were estimated across all ecologies and by crop. In assessing the differences in these variables across ecology and

starchy staples, we used linear regression for continuous variables and a probit model for dummies. A trend variable, and a fixed effect for ecology and crop, as well as their interactions, were included in the estimations. Plots of differences in the household-level variables across ecologies are further elaborated in Figure S1 (*a* and *b*).

From Table 1, an average household contains about five members in adult equivalence (AE) units, consistent with national estimations of 4.4 and 5.1 in the 2010 and 2000 censuses respectively [32]. The dependency ratio, with an average rate of 1.29, measures the extent of pressure on an earning individual in the household. Averagely, the starchy staple farmer in Ghana is a 47-year-old male with five years of formal education and a 61% chance of owning his main farm plot. Regarding starchy staples, we found no significant variation between males and females (1=female), education, and land ownership; but in terms of ecological zones, we found significant variation in these variables. Age, on the other hand, showed no significant ecological or crop variation. In terms of their temporal dynamics from 1987 to 2017, while the probability of observing a female starchy staple farmer significantly declined by 0.18% annually, that of age and years of formal education increased by 0.47% and 1.59% annually, respectively.

Table 1. Summary statistics of starchy staple producing farmers in Ghana (1987-2017**)**

Variable	Mean (SD)	Trend (%)
Household [a]		
Size (AE)	4.83 † (2.887)	0.10 [0.055]
Dependency (ratio)	1.29 (1.561)	0.16 [0.113]
Farmer [b]		
Female (dummy)	0.29 † (0.454)	-0.18* † [0.031]
Age (years)	46.84 (14.872)	0.47* [0.029]
Education (years)	5.01 † (4.820)	1.59* † ‡ [0.103]
Land owned (dummy)	0.61 † (0.489)	-0.03 † [0.033]
Land (ha) [b]		
Cassava	1.17 † (2.753)	-54.18 † [59.567]
Yam	1.57 † (3.655)	-13.88* † [2.141]
Cocoyam	1.55 (3.883)	10.54 [832.372]
Plantain	1.19 † (2.611)	-7.18 † [105.680]

Yield (Mt/ha) [b]		
Cassava	0.66 † (1.080)	2.78* † [0.220]
Yam	0.96 (1.540)	-38.47 † [3146.763]
Cocoyam	0.31 † (0.547)	0.15 [0.487]
Plantain	0.53 † (0.838)	1.57* † [0.296]
Input use [b]		
Planting material (Mt/ha)	0.06 † ‡ (0.428)	-5.68 † ‡ [20.428]
Family labor (AE)	2.74 † (1.544)	0.45* [0.054]
Hired labor (man-days/ha)	11.29 ‡ (43.200)	1.24* † ‡ [0.350]
Pesticide (Liter/ha)	7.83 † (49.505)	-36.04 † ‡ [2059.161]
Enabling environment [a]		
Credit (dummy)	0.20 † ‡ (0.399)	-0.06* ‡ [0.028]
Mechanization (dummy)	0.04 † ‡ (0.201)	-0.21* ‡ [0.015]
Extension (dummy)	0.19 (0.392)	0.75* † ‡ [0.029]

* Indicates significance at $p<0.05$; † and ‡ indicate statistically significant ($p<0.05$) differences in the variables across ecology and crop, respectively. The differences were determined via a linear regression for continuous variables and a probit model for dummies. A trend variable, and a fixed effect for ecology and crop, as well as their interactions, were included in the estimation.

[a] Household sample size; Cassava [11,459], Yam [3,439], Cocoyam [1,830], Plantain [6,166], Pooled [15,133]

[b] Farmer sample size; Cassava [11,585], Yam [3,468], Cocoyam [1,834], Plantain [6,226], Pooled [15,405]

Data Sources: Ghana Living Standards Surveys [wave 1-7] and Ghana Socioeconomic Panel Survey [wave 1-2]

In terms of production scale, the value of mean land area confirms the widely held notion that agriculture in Ghana is operated on a smallholder basis. Generally, yam (1.57 ha) has received the largest plot allocation for the period 1987-2017 while cassava (1.17 ha) received the lowest. Starchy staple farm sizes are statistically different across ecologies and have declined over the period (1987-2017). Given these scales of production, yields were estimated at 0.66, 0.96, 0.31, and 0.53 Mt/ha for cassava, yam, cocoyam, and plantain, respectively. Except for cocoyam, these yields also vary by ecology. Nonetheless, except for yam, all crops show an annual upward trend in yields from 1987 to 2017.

Quantities and types of inputs used in production significantly determine technical efficiency and technological levels. Technical efficiency relates to how much output can be obtained from a given input, such as a worker or a machine, or a specific combination of inputs. In other words, it the effectiveness with which a given input set is converted to produce an output. If the farmer is unsuccessful in converting the inputs to the desired outputs, the farmer is said to be technically

inefficient. The extent of inefficiency is measured by how much of the desired output is lost due to improper combination of the outputs. Maximum technical efficiency occurs when output is maximised from a given quantity of inputs. The starchy staple production functions analyzed in this study focused on the technical transformation of land, planting materials, family and hired labor, and pesticides into outputs. The planting material usage rate was estimated at 0.06 Mt/ha, with the Sudan Savannah ecology having the highest and Coastal Savannah having the lowest. On average, three household members provided labor for starchy staple production, and this has significantly increased by about 0.45% annually since 1987. Hired labor is used at a rate of about 11 man-days/ha, which is equivalent to an annual increase of about 1.24% since 1987. The highest usage of hired labor is for farms in the Sudan Savannah zone, followed by Transition, Coastal Savannah, Guinea Savannah, and Forest zones, in that order. Farmers in the sample used about 8 liters of pesticides per hectare. Analyzing pesticide use across ecological zones and time shows consistently low usage in the Coastal Savannah relative to the Sudan Savannah and Forest zones for all crops.

Access to credit, mechanization, and extension (measured as dummies) depicts factors that reflect the enabling environment for production success. Access to these production support services is reported in the literature to have diverse effects on technical inefficiency. The rates of access to credit and extension are estimated at 20% and 19% respectively while that for mechanization is a paltry 4%. Furthermore, Table 1 shows that the rate of access to both credit and mechanization has significantly declined annually by 0.06% and 0.21% for credit and mechanization respectively but increased for extension by 0.75% from 1987 to 2017. We present the heterogenous effects of these variables on technical inefficiency across crops and ecological technology gaps in Section 4.

3. Econometric strategy

Several studies have shown that farmers in Ghana operate under spatially differentiated technologies [11,33] which have been linked to variability in Ghana's agroecology. We present Figures in the online appendix and statistical tests in Table 2 that suggest that production characteristics do vary across ecologies and starchy staples. Thus, it is important to account for these spatial heterogeneities driven by agroecological zones when investigating the production performance of starchy staple farmers operating at different levels relative to the best practice frontier. According to Battesse et al. [26], not accounting for these technological differences could lead to false attribution of production shortfalls. In the estimation of our empirical model, we group farmers into agroecological technology groups and apply the MSF following Huang et al. [28] two-step method as outlined below.

For each starchy staple in each ecology, we assumed a homogenous farm production technology, which, when coupled with best management practice, situates farmers at various points along their respective ecology Stochastic Frontier (SF). However, due to technical inefficiency and/or idiosyncratic shocks (i.e., downside production risk), one may observe some farmers below the SF [34]. Furthermore, some farmers may also be observed above the SF solely due to upside production risk.

Previous studies have shown that Ghanaian crop production at the micro-level is best captured by the Cobb-Douglas [8,10], or Translog [11,22] production function. However, due to its relative flexibility, this study implements the MSF assuming that $f_t^j(\cdot)$ is Translog. Nonetheless, since Cobb-Douglas is nested within the Translog, the former was tested after estimation and was soundly rejected at p<0.01 for all models (see Table 2). Thus, the Translog specification is used. The SF production function for the jth agro-ecology is specified as:

$$f^j(x_i) = \ln y_{ijt} = \beta_{0r} + \sum_k \beta_{kj} \ln x_{kijt} + \frac{1}{2}\sum_s \sum_k \beta_{skj} log \ln x_{kijt} \ln x_{sijt} + v_{ijt} - u_{ijt}$$

$$u_{ijt} \sim N^+\left[0, \exp\left(\boldsymbol{w}_{ijt}\boldsymbol{\alpha}\right)\right], \qquad v_{ijt} \sim N(0, \sigma_v^2) \qquad (1)$$

where, y_{ijt} is total production (Mt) for the ith farmer in ecology j at time t. Each x_{kijt} represents the kth input (i.e., land, planting material, family and hired labor, and pesticides) used by the ith farmer and a trend variable. As Van Nguyen et al. [35] report, outcomes from the SF, especially with efficiency estimates, are severely affected by the distribution assumptions. Therefore, we considered all possible distributions but eventually had to settle on a half-normal distribution (i.e., $u_{ijt} \sim N^+\left[0, \exp\left(\boldsymbol{w}_{ijt}\boldsymbol{\alpha}_j\right)\right]$), since the truncated normal and exponential distributions presented issues of convergence. Following Tsiboe et al. [23], $\boldsymbol{w}_{ijt}$ contained farmer characteristics (age, education, and gender), institutional factors (land ownership, credit, and extension), a trend and a constant term. Given the SF production function for the jth agro-ecology, the "pure farmer technical efficiency" (TE) of the ith farmer is calculated as:

$$TE_i = E[\exp(-u_i) \,|\hat{\varepsilon}_i] \qquad (2)$$

To implement Huang et al's. [28] two-step MSF method, Equation (1) is initially estimated separately for each agro-ecology (j), and then the predicted output levels from the agro-ecology frontiers are used as the observation for MSF (pooled stochastic frontiers) in the second step. The conventional one-sided error term (u_{iM}) for the MSF serves as the estimate for any technology gaps among the different agro-ecology. The MSF [$f^M(x_i)$] which envelops all agro-ecology-specific stochastic frontiers [$f^j(x_i)$] is specified in Equation (3) as

$$f^M(x_i) = \ln \hat{y}_{ijt} = \beta_{0r} + \sum_k \beta_{kM} \ln x_{kiMt} + \frac{1}{2}\sum_s \sum_k \beta_{skM} log \ln x_{kiMt} \ln x_{siMt} - u_{iMt} \qquad (3)$$

Where $u_{iM} \sim N^{+}\left(0, \exp(\boldsymbol{w}_i \boldsymbol{\alpha}_M)\right)$ is strictly positive, implying that $f^j(x_i) \le f^M(x_i)$. Consequently, the ratio of agro-ecology j's stochastic frontier to the MSF is the technology gap ratio (TGR), represented as

$$TGR_i = \frac{f^j(x_i)}{f^M(x_i)} = e^{-u_{iM}} \le 1 \qquad (4)$$

The TGR depends on the accessibility and adoption level of the available MSF which in turn depends on farm-specific circumstances. The meta-technical efficiency (MTE), a measure of overall performance, represents each farmer's technical efficiency with respect to the meta frontier production technology. MTE can be decomposed into the TE (which is the technical efficiency measured with respect to the zone-specific frontier) and the TGR (which is the difference between the best available technology and the technology set adopted). Accordingly, each farmer's MTE is given by equation (5) as follows:

$$MTE_i = f^j(x_i)[f^M(x_i)e^{v_i}]^{-1} = TGR_i \times TE_i \qquad (5)$$

The MSF is estimated separately for each crop, so we use the physical measure of total output and input (log-transformed) used by the farmer. Thus, all outputs and inputs are not standardized by farm size. The parameters of the ecology- and meta-frontiers were estimated via maximum likelihood, using the "*frontier*" command in Stata 15. Furthermore, estimation variables were standardized by their sample means by survey and crop. Given the parameters, input elasticities were estimated as the first derivative of the frontiers with respect to that input, evaluated at their means. The production returns to scale (RTS) were then estimated as the sum of the input elasticities. The standard errors for the elasticities were estimated via the delta method. Ecology-specific TE, TGR, and MTE were estimated using equations (2), (4), and (5), respectively.

4. Econometric results

The core objective of this paper is to understand whether production shortfalls existing among the starchy staples in Ghana are driven by technological gaps, technical inefficiency, or both. This results section has four parts. First, we present results on diagnostic tests of the empirical model specification before proceeding to the estimates of inputs elasticities, returns to scale (RTS), and technical change for each crop, ecology, and the meta-frontier in the second part. This is followed by the spatiotemporal trends of TE, TGR, MTE, and technology gaps in the third part. The final part presents results on drivers of technical inefficiency and technology gaps. Estimates of the production functions for each crop (Tables S3-S6), ecological variations in production yield, input, and enabling environment factors, and in starchy staple farmer and household demographic factors in Ghana (Figures S1a and S1b) are presented in the online appendix.

4.1 Diagnostic tests of the empirical model specification

The tests results for functional form specification, skewed error specification, production variance due to technical inefficiency, and hypothesis tests that variables in the inefficiency functions of ecology-specific and meta-frontier models do not affect starchy staple inefficiency are presented in Table 2.

The Cobb-Douglas and Translog functional forms are the most popular choices when specifying MSF production functions. This study tested the null hypothesis that cross terms in the translog functional form are jointly equal to zero. The test result for all crops, ecologies, and the meta-frontier soundly rejected this hypothesis at 1% level, showing that the translog function better fits the data. In the literature, the translog function is generally preferred because of its flexibility. For example, both Asravor et al. [8] and Tsiboe [19] used the translog specification for the MSF for rice and cocoa production in Ghana.

The results from the Coelli [36] skewness test for negative OLS residuals and the Gutierrez et al. [37] likelihood ratio tests for technical inefficiency both reject the null hypothesis of no one-sided error and show that the MSF modeled for starchy staple technical inefficiency functions is justified. Further, the null hypothesis that the inefficiency variance is not influenced by variables in the inefficiency function ($H_o\colon \boldsymbol{\alpha} = 0$) was also rejected for all crops, ecologies, and the meta-frontier. The proportion of production variance due to inefficiency (γ) ranged from 0.50 to 0.99 for the ecology frontiers; for a given crop, this was always higher than that of the meta-frontier.

Table 2. Hypothesis tests for ecology- and meta-frontier models for starchy staple production in Ghana (1987-2017)

	Sample size	Log-likelihood	Cobb-Douglas test	Coelli, (1995) [ab] skewness test	Gutierrez (2001) [a] LR test	Inefficiency variance [σ_u]	Total variance [$\sigma^2 = \sigma_u^2 + \sigma_v^2$]	Gamma [$\gamma = \sigma_u^2/\sigma^2$]	Inefficiency function test	Model significance
Cassava										
Guinea Savanah	801	-919	227.02***	-6.02*	11.28***	0.87 (0.092)	1.10 (0.119)	0.68*** (0.076)	37.20***	710.21***
Transitional Zone	2,064	-2,735	307.98***	-3.52*	13.96***	0.94 (0.088)	1.46 (0.118)	0.61*** (0.066)	88.27***	1194.47***
Forest Zone	7,023	-9,129	553.51***	-5.12*	19.05***	0.77 (0.061)	1.20 (0.065)	0.50*** (0.053)	109.64***	3941.36***
Coastal Savanah	1,697	-2,154	211.58***	-2.84*	6.13**	0.80 (0.114)	1.21 (0.126)	0.53*** (0.097)	403.63***	1390.95***
National	11,585	-15,347	1013.19***	-9.07*	66.22***	0.89 (0.038)	1.37 (0.048)	0.58*** (0.030)	196.70***	6319.90***
Meta-frontier	11,585	-166	16605.98***	4555.61	-	0.00 (0.036)	0.05 (0.001)	0.00 (0.001)	563.00***	91075.02***
Yam										
Sudan Savanah	222	-233	164.54***	-0.23	0.52	0.87 (0.402)	1.12 (0.480)	0.68** (0.339)	534.06***	500.60***
Guinea Savanah	1,472	-1,732	400.32***	-9.80*	34.53***	1.04 (0.064)	1.34 (0.103)	0.81*** (0.041)	25.54*	2378.83***
Transitional Zone	943	-1,288	199.15***	-4.89*	32.23***	1.35 (0.091)	2.16 (0.195)	0.84*** (0.042)	1666.17***	1328.45***
Forest Zone	831	-1,212	170.34***	-3.31*	23.13***	1.41 (0.115)	2.51 (0.251)	0.79*** (0.054)	48.43***	772.00***
National	3,468	-4,936	515.54***	-12.13*	226.81***	1.53 (0.040)	2.67 (0.104)	0.88*** (0.014)	213.99***	4669.74***
Meta-frontier	3,468	-1,994	3948.97***	-197.43*	742.33***	0.72 (0.011)	0.53 (0.015)	0.97*** (0.003)	14048.06***	38680.49***
Cocoyam										
Transitional Zone	422	-597	99.75***	-1.84	9.11***	1.38 (0.171)	2.40 (0.359)	0.79*** (0.084)	23.91**	322.93***
Forest Zone	1,412	-2,046	75.45***	-3.13*	25.43***	1.36 (0.104)	2.37 (0.212)	0.78*** (0.053)	42.48***	495.20***
National	1,834	-2,721	113.04***	-2.54*	20.51***	1.27 (0.106)	2.29 (0.195)	0.71*** (0.060)	54.73***	611.93***
Meta-frontier	1,834	-377	1587.92***	658.26	-	0.00 (0.082)	0.08 (0.003)	0.00 (0.004)	890.54***	8960.81***
Plantain										
Transitional Zone	740	-985	125.50***	-5.65*	39.00***	1.38 (0.085)	2.21 (0.195)	0.86*** (0.035)	63.15***	760.94***
Forest Zone	4,982	-6,564	357.18***	-6.48*	36.32***	0.97 (0.057)	1.46 (0.079)	0.64*** (0.042)	46.45***	3483.09***
Coastal Savanah	504	-576	109.18***	0.28	-	0.03 (0.690)	0.61 (0.046)	0.00 (0.064)	1138.60***	390.07***
National	6,226	-8,275	447.69***	-9.27*	78.14***	1.07 (0.044)	1.62 (0.068)	0.70*** (0.029)	88.04***	4366.28***
Meta-frontier	6,226	1,264	9511.74***	-1939.14*	1062.47***	0.40 (0.006)	0.19 (0.005)	0.83*** (0.008)	544.13***	113229.19***

Significance levels: * $p<0.10$, ** $p<0.05$, *** $p<0.01$

[a] Null hypothesis of no one-sided error (i.e., no inefficiency) was tested

[b] Values less than the critical value of -1.96 confirm the rejection of the null hypothesis.

Data Sources: Ghana Living Standards Surveys [wave 1-7] and Ghana Socioeconomic Panel Survey [wave 1-2]

The γ values suggest that a considerable amount of the observed variation in output for the ecology-frontiers could be attributed to inefficient use of inputs. The range of γ for the meta-frontier also suggests that the observed variation in output, given the ecology frontiers, could not be attributed to idiosyncrasies.

To statistically check the similarities of the production frontiers for a given starchy staple across the different ecologies, the study applied a generalized likelihood ratio test. The log-likelihood values under the null hypothesis of a uniform technology were all smaller than that of the alternate hypothesis of heterogeneous technology. The test statistics led to sound rejection of the null hypothesis, implying that starchy staple farmers are operating under heterogeneous technologies along ecological lines.

Finally, the SF model chi-squared test statistics indicate that the models are all significant. These justify further inquiry into whether technology gaps across ecologies or resource use inefficiencies among farmers contribute more to production shortfalls. We report the summary of estimates for the ecology- and meta-frontier production elasticities in Table 3 and TGR, TE, and MTE for starchy staples production in Table 4. Detailed results from these are discussed in the following subsections along crop-specific lines.

4.2 Output elasticities with respect to inputs, returns to scale and technical change.

Given that earlier results confirmed heterogeneous production technologies along ecological lines, we estimated separate ecology-frontier and meta-frontier models for each crop and estimated local and national returns to scale (RTS) parameters from these models to test the null hypothesis of constant RTS from each model. The estimated elasticities for ecology-specific and meta-frontier models for all starchy staples are presented in this order in Table 3.

4.2.1 Cassava production

Cassava is produced in all ecological zones under study except Sudan Savannah. The elasticities for land are consistently statistically significant and contribute the most to explaining changes in cassava production efficiency across all ecologies and the meta-frontier. Land elasticity is highest in the Coastal Savannah zone (0.41) and lowest in the Guinea Savannah (0.31) while the national average land elasticity of 0.39 is like the meta-frontier estimate. Family labor contributes the next highest elasticities followed by hired labor. Again, the estimate for family labor is highest in the Coastal Savana (0.17) but the lowest for Transition zone (0.10). The national and meta-frontier elasticities average about 0.16 each. The estimated elasticities for hired labor are comparable across ecologies and the meta-frontier with the Guinea Savannah zone (0.05) slightly outperforming the rest (0.04). Where statistically significant, the elasticities for planting materials and pesticides reduce both the production frontiers and ecological technologies for cassava production. The negative effect for planting material is highest in the Guinea Savannah zone (-0.13) and is, at least, three times the estimated national (-0.03) and meta-frontier (-0.04) effects. The negative effect of pesticide use on cassava output is even more distressing since the estimated elasticity for Coastal Savannah (-0.09) outstrips the national and meta-frontier elasticities by about nine-folds.

Taken together, cassava production exhibits decreasing RTS for all ecologies and at the national and meta-frontier levels. The ecology RTS for cassava production ranges from 0.39 in the Guinea Savannah to 0.53 in both the Transitional and Forest zones. The national (0.55) and meta-frontier (0.54) RTS for cassava align with the agro-ecology findings.

Table 3. Elasticities for ecology- and meta-frontier models for starchy staple production in Ghana (1987-2017)

	Land elasticity	Planting material elasticity	Family labor elasticity	Hired Labor elasticity	Pesticide elasticity	Returns to scale [a]
Cassava						
Guinea Savanah	0.31*** (0.030)	-0.13*** (0.025)	0.16*** (0.063)	0.05*** (0.008)	0.00 (0.014)	0.39*** (0.069)
Transitional Zone	0.36*** (0.019)	0.02 (0.018)	0.10** (0.042)	0.04*** (0.009)	0.01 (0.007)	0.53*** (0.047)
Forest Zone	0.40*** (0.010)	-0.04*** (0.010)	0.14*** (0.023)	0.04*** (0.003)	-0.01* (0.004)	0.53*** (0.026)
Coastal Savanah	0.41*** (0.020)	-0.05*** (0.019)	0.17*** (0.046)	0.04*** (0.007)	-0.09*** (0.018)	0.47*** (0.059)
National	0.39*** (0.008)	-0.03*** (0.008)	0.16*** (0.018)	0.04*** (0.003)	-0.01** (0.004)	0.55*** (0.020)
Meta-frontier	0.39*** (0.002)	-0.04*** (0.002)	0.16*** (0.005)	0.04*** (0.001)	-0.01*** (0.001)	0.54*** (0.006)
Yam						
Sudan Savanah	0.44*** (0.068)	-0.08** (0.032)	0.18* (0.103)	0.01 (0.015)	-0.03 (0.020)	0.51*** (0.133)
Guinea Savanah	0.59*** (0.025)	-0.08*** (0.019)	0.02 (0.047)	0.02*** (0.005)	-0.02 (0.013)	0.52*** (0.056)
Transitional Zone	0.55*** (0.035)	0.00 (0.020)	-0.05 (0.061)	0.10*** (0.016)	0.02 (0.011)	0.61*** (0.072)
Forest Zone	0.32*** (0.030)	-0.08* (0.043)	0.20*** (0.076)	0.09*** (0.014)	-0.01 (0.014)	0.53*** (0.091)
National	0.55*** (0.019)	-0.06*** (0.013)	0.12*** (0.035)	0.05*** (0.005)	-0.02*** (0.007)	0.64*** (0.040)
Meta-frontier	0.51*** (0.009)	-0.07*** (0.005)	0.10*** (0.019)	0.06*** (0.002)	-0.01*** (0.003)	0.58*** (0.021)
Cocoyam						
Transitional Zone	0.19*** (0.040)	-0.18** (0.083)	0.21** (0.104)	0.08*** (0.022)	-0.03 (0.034)	0.27*** (0.134)
Forest Zone	0.42*** (0.041)	-0.10*** (0.037)	0.08 (0.059)	0.04*** (0.010)	0.02 (0.016)	0.45*** (0.076)
National	0.36*** (0.027)	-0.10*** (0.035)	0.09* (0.052)	0.05*** (0.009)	0.01 (0.014)	0.42*** (0.066)
Meta-frontier	0.38*** (0.005)	-0.10*** (0.015)	0.11*** (0.015)	0.05*** (0.003)	0.01*** (0.004)	0.46*** (0.023)
Plantain						
Transitional Zone	0.44*** (0.031)	-0.04 (0.032)	0.10 (0.066)	0.06*** (0.011)	0.02 (0.015)	0.59*** (0.073)
Forest Zone	0.47*** (0.014)	-0.03** (0.013)	0.14*** (0.027)	0.04*** (0.004)	0.00 (0.005)	0.62*** (0.031)
Coastal Savanah	0.37*** (0.032)	0.06* (0.033)	0.12* (0.073)	0.07*** (0.015)	-0.05** (0.023)	0.58*** (0.098)
National	0.47*** (0.012)	-0.02* (0.011)	0.14*** (0.025)	0.04*** (0.004)	0.00 (0.005)	0.63*** (0.028)
Meta-frontier	0.47*** (0.002)	-0.03*** (0.002)	0.14*** (0.006)	0.04*** (0.001)	0.01*** (0.001)	0.63*** (0.007)

Significance levels: * p<0.10, ** p<0.05, ***p<0.01
[a] Null hypothesis of constant returns to scale was tested.
Data Sources: Ghana Living Standards Surveys [wave 1-7] and Ghana Socioeconomic Panel Survey [wave 1-2]

4.2.2 Yam production

From Figure 2 and Table 3, yam is produced in all agro-ecologies except Coastal Savannah. Like cassava, land contributes the most to explaining variations in yam output followed by family labor and hired labor. Pesticides and planting materials maintain their negative effects on output and ecology technology. Guinea Savannah (0.59) shows the most while Coastal Savannah (0.32) shows the least responsiveness to marginal changes in area cultivated. The elasticity of family labor in the Forest zone (0.20) is about 60% higher than the national elasticity (0.12) and twice that of the meta-frontier. Although family labor showed no effect in the Transition zone, hired labor elasticity (0.10) is twice the national average. Where statistically significant, planting material had negative effects on yam output at the ecological and meta-frontier levels. The elasticities of yam output with respect to changes in planting material clustered around -0.08 in the forest ($p<0.10$), Sudan Savannah ($p<0.05$), and Guinea Savannah ($p<0.01$) zones: seed yam showed no effect on output in the Transition zone. Like planting material, pesticide had generally negative effects on yam output (except in the Transition zone) but none of the ecology-specific elasticities were statistically significant. These results reflect the heterogenous production technologies for the same crop in different ecologies. At the meta-frontier level, both planting material (-0.07) and pesticide (-0.01) showed negative effects on yam output. Like cassava production, the RTS for yam also reflects decreasing returns to scale in all ecologies and the meta-frontier although the national level estimate (0.64) is the highest among all crops.

4.2.3 Cocoyam production

Cocoyam production in Ghana happens largely in the Transition and Forest ecological zones. These areas have more suitable biological conditions for this crop, but the estimated elasticities show marked heterogeneities in input use and production technologies. For example, land elasticity in the Forest zone (0.42) is more than twice the effect in the Transition zone (0.19); the

elasticity of family labor in the Transition zone (0.21) is the highest of all inputs in that zone but family labor shows no effect on cocoyam output in the Forest zone. Hired labor shows complementary relationships with family labor in both ecologies but its effect in the Transition zone is twice (0.08) that of the Forest zone. Pesticide application does not significantly influence cocoyam output while planting material maintains its negative output effects in both ecologies (Transition =-0.18; Forest = -0.10) and in the meta-frontier (-0.10). These negative effects are reflected in the low RTS for the Transition zone (0.27). Cumulatively, the meta-frontier RTS for cocoyam production (0.46) is the least among the starchy staples and implies that a 100% increase in all inputs will only yield a 46% increase in cocoyam output.

4.2.4 Plantain production

Plantain is mainly cultivated in the Transition, Forest, and Coastal Savannah zones of Ghana. Its ecology-specific and meta-frontier elasticities show that land contributes most to output, and the effect is more pronounced in the Forest zone (0.47) but lowest in the Coastal Savannah zone (0.37). The estimated land elasticity of 0.47 in the Forest zone is consistent with its analogues at the national and meta-frontier levels. Similar observations can be made for the elasticities of family labor where the estimate for Forest zone (0.14) is the same for the national and meta-frontier level. Interestingly, the elasticities of family labor (0.10) and planting material (-0.04) in the Transition zone have no significant effect on plantain output as was the case for yam production in that zone. Hired labor showed consistently significant and positive effects on plantain output with the elasticities ranging from 0.04 in the Forest zone to 0.07 in the Coastal Savannah while planting material and pesticide elasticities continued their largely negative effects. Interestingly, the only statistically significant effect of pesticide elasticity (-0.05) was found in the Coastal Savannah zone and, that zone also recorded the only positive effect of planting materials on plantain output. Like

the others, plantain production also showed decreasing RTS at the national and meta-frontier levels although the estimates for the latter (0.63, $p<0.01$) was the highest of all crops under study.

4.3 Technical efficiency, technology gap, and meta-technical efficiency of starchy staples

The summary of TE, TGR, and MTE estimates for agro-ecological zones are presented in Table 4, while Figures 1 and 2 present the corresponding plots. As indicated, these efficiency parameters are estimated from equations 2, 4, and 5. Detailed results from these estimations are presented for each starchy staple.

Table 4**.** Ghanaian starchy staple production technology level and technical efficiency across ecologies (1987-2017)

	Technology gap ratio (TGR)	Technical efficiency (TE)	Meta-technical efficiency (MTE)	Technology gap
Cassava				
Guinea Savanah	0.92*** (0.002)	0.64*** (0.007)	0.60*** (0.006)	8.50%
Transitional Zone	0.90*** (0.001)	0.58*** (0.004)	0.54*** (0.003)	9.69%
Forest Zone	0.87*** (0.000)	0.64*** (0.001)	0.56*** (0.001)	12.82%
Coastal Savanah	0.84*** (0.003)	0.72*** (0.005)	0.59*** (0.003)	16.31%
Mean	0.89	0.65	0.58	11.44%
Yam				
Sudan Savanah	0.81*** (0.009)	0.67*** (0.018)	0.55*** (0.016)	18.72%
Guinea Savanah	0.81*** (0.003)	0.54*** (0.005)	0.43*** (0.004)	18.70%
Transitional Zone	0.70*** (0.004)	0.49*** (0.007)	0.34*** (0.005)	30.00%
Forest Zone	0.55*** (0.005)	0.51*** (0.008)	0.24*** (0.005)	45.00%
Mean	0.65	0.54	0.33	35.11%
Cocoyam				
Transitional Zone	0.77*** (0.009)	0.56*** (0.011)	0.38*** (0.009)	22.66%
Forest Zone	0.82*** (0.002)	0.48*** (0.005)	0.39*** (0.004)	17.76%
Mean	0.80	0.47	0.36	20.21%
Plantain				
Transitional Zone	0.94*** (0.002)	0.50*** (0.008)	0.47*** (0.007)	6.39%
Forest Zone	0.87*** (0.000)	0.58*** (0.002)	0.51*** (0.002)	13.01%
Coastal Savanah	0.75*** (0.005)	0.68*** (0.009)	0.50*** (0.008)	25.07%
Mean	0.85	0.58	0.50	15.13%
Overall mean	0.791	0.553	0.428	20.94%

Significance levels: * $p<0.10$, ** $p<0.05$, *** $p<0.01$
[a] Null hypothesis of constant returns to scale was tested.
Data Sources: Ghana Living Standards Surveys [wave 1-7] and Ghana Socioeconomic Panel Survey [wave 1-2]

4.3.1 Cassava production

The technology gap (ranging from 0 to 1) is the difference between the best technology available in the production of a particular crop and the technology set adopted by a farmer. TGR values

closer to one mean there is little room for improvement given the existing production technology in the country. The choice of the adopted technology is determined by environmental and socio-economic factors. The mean TGR for cassava, estimated at 0.89, indicates that the technological gap averaged over all ecological zones is 11%. Figure 1, panel a1 shows that the TGR for cassava increased from 0.72 in 1987 to 0.98 in 1998 and dropped to an average of 0.85 between 2004 and 2012. Afterward, it increased until settling at 0.88 in 2017. Given the high mean TGR across the sample, it is not surprising that the ecology specific TGRs are all above 80% with the highest in Guinea Savannah (0.92), reflecting a superior technology for that ecology. This is followed by the Transition zone (0.90), the Forest zone (0.87), and then Coastal Savannah (0.84). The TGR for cassava is relatively high when compared to 0.75 for tomato [23] and 0.74 for rice [11].

In terms of TE, the mean ecology-specific estimate for cassava is 0.65 across the entire sample. Trend-wise, the TE for cassava increased by about 90% from 0.46 in 1987 to a peak of 0.85 in 1991 but has been declining since then by about 5% annually to 0.46 again in 2017 (Figure 1, panel b1). Figure 2 shows that the mean TE for cassava is highest in Coastal Savannah (0.72) and followed by Forest/Guinea Savannah zones (0.64) and then Transition zone (0.58). An important caveat about these TE scores is that they do not rank the farms based on the cassava meta-frontier, so we discuss the MTE results next.

After accounting for ecology-specific production technologies, the mean MTE for cassava across the entire sample is 0.58. Specifically, the most technically efficient cassava farmers relative to the meta-frontier are those in the Guinea Savannah with an MTE of 0.60.

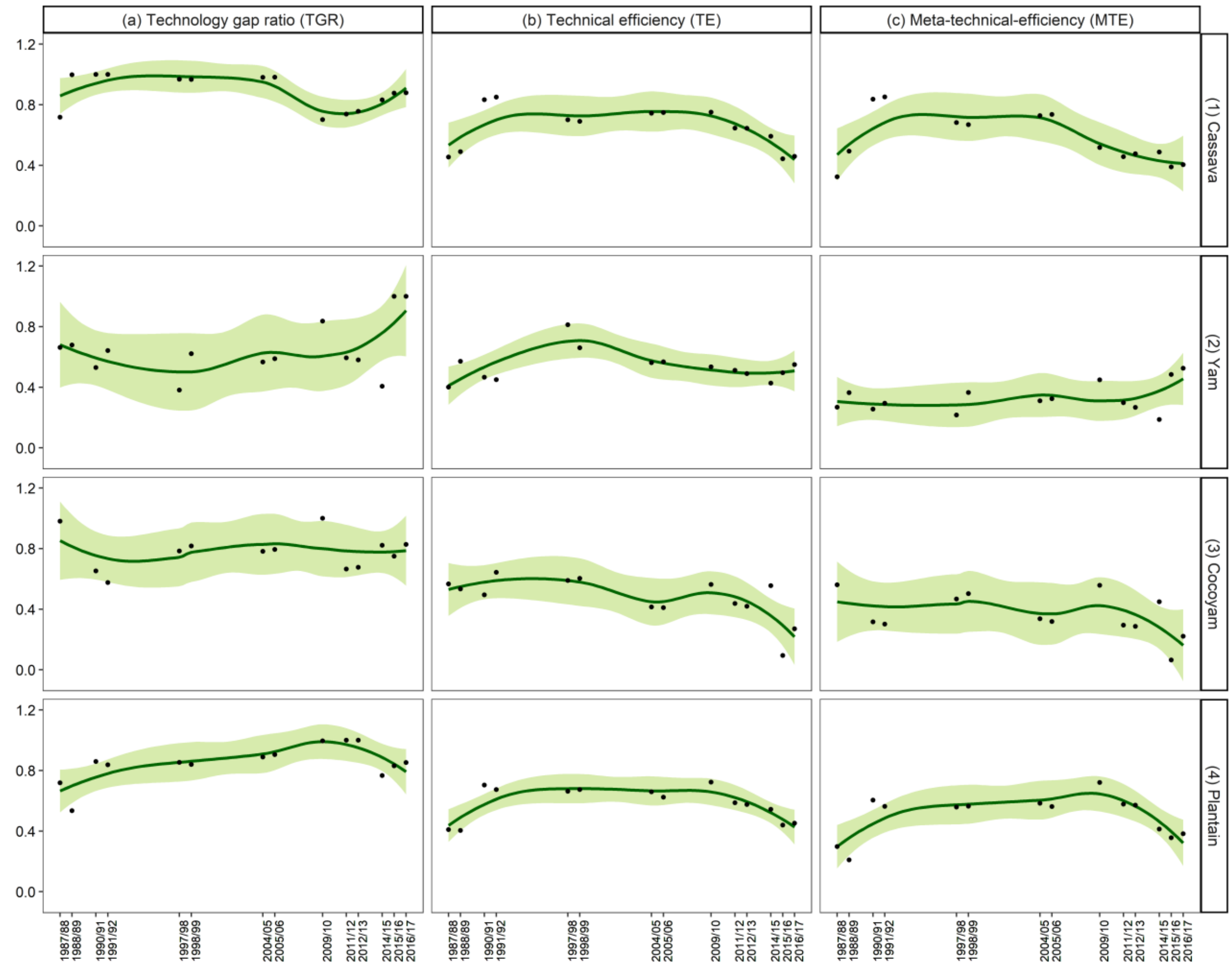

Figure 1. Ghanaian Starchy Staple Production Technology Level and Technical Efficiency Parameters Across Seasons (1987-2017)

Notes: Farmer-level scores were first estimated via a Meta−stochastic−frontier (MSF) analysis applied separately to 9 population−based surveys that represent 30 years of farmer-level data collection in Ghana the surveys used included Ghana Living Standards Surveys [wave 1−7] and Ghana Socioeconomic Panel Survey [wave 1−2]. The farmer-level elasticities were subsequently averaged across seasons via a regression framework to account for controls. Each point on a sub−panel represents the mean of the estimates. Given the seasonal means, the fitted line was done locally using neighborhood points, weighted by distance. The size of the neighborhood was set to 75% of the points with a tri−cubic weighting. The gray region is the 95% confidence interval of the fitted line.

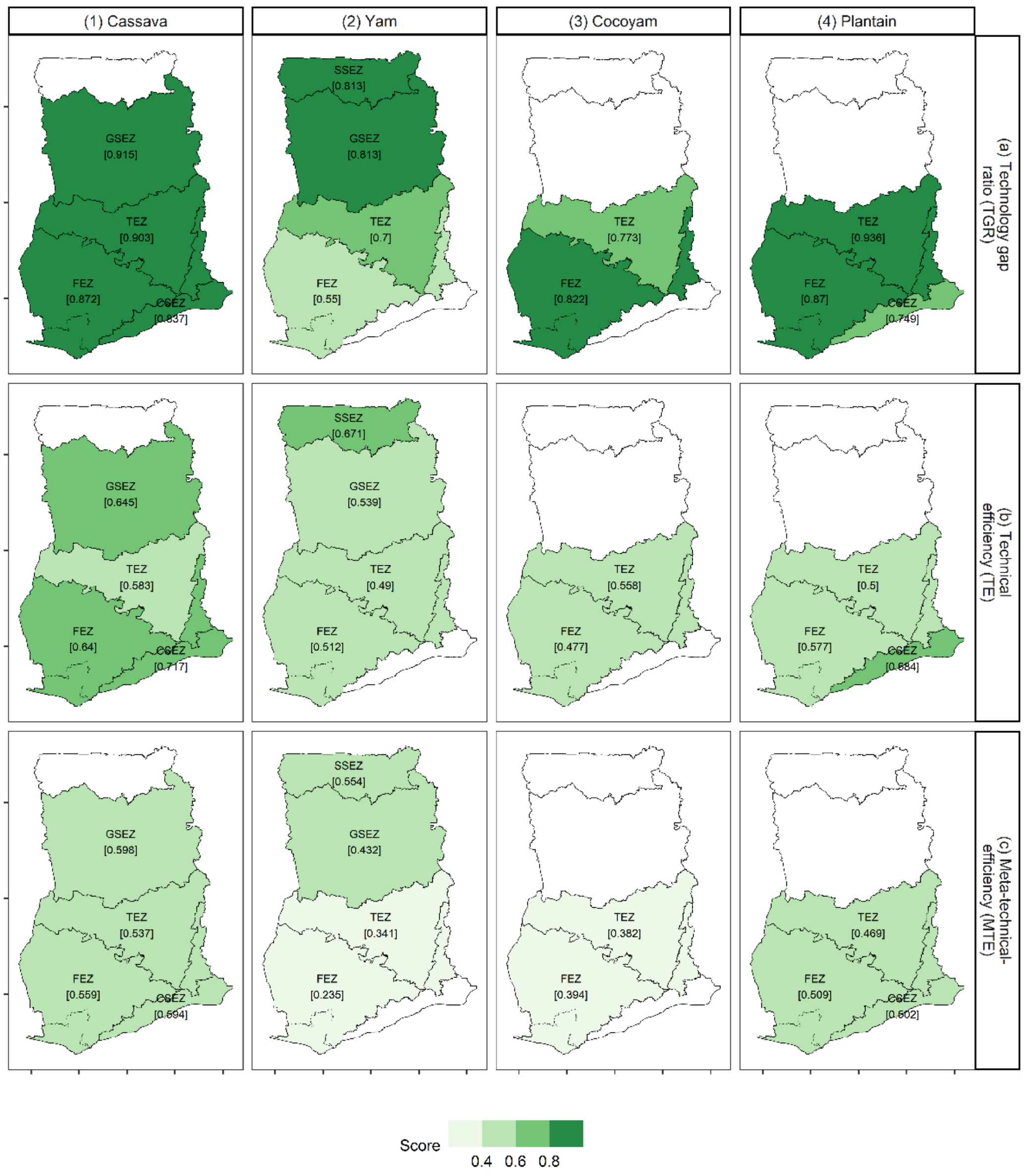

Sudan Savanna=SSEZ; Guinea Savanna=GSEZ; Transitional Zone= TEZ;Forest Zone=FEZ

Figure 2. Ghanaian Starchy Staple Production Technology Level and Technical Efficiency Across Ecologies (1987-2017)

Notes: Farmer-level scores were estimated via a Meta−stochastic−frontier (MSF) analysis applied separately to 10 population−based surveys that represent 30 years of farmer-level data collection in Ghana the surveys used included Ghana Living Standards Surveys [wave 1−7] and Ghana Socioeconomic Panel Survey [wave 1−2].

Figure 2 shows that the Guinea Savannah farmers are followed by their peers in Coastal Savannah (0.59), Forest zone (0.56), and then Transition zone (0.54). Figure 1, panel c1 shows that the MTE

increased from 0.32 in 1987 to 0.85 in 1991 due to improvements in technology adoption. However, from 1991 to 2017, MTE declined at the same rate as pure farmer technical efficiency to 0.40. We return to the implications and policy learning points from these findings in the Discussion section.

4.3.2 Yam production

The mean TGR for yam, estimated at 0.65, points to an average ecological technology gap of about 35%. Figure 1, panel a2 shows that the TGR for yam has increased steadily over the study period but this generalization masks significant dips and peaks over time. For example, the TGR for yam dropped by about 4.25% annually in the decade between 1987 (0.66) and 1997 (0.38); this rebounded to 0.62 in 1998 only to sink to 0.41 in 2014 before hitting another gear shift to peak at 1.00 in both 2016 and 2017. At the ecology level, the highest TGR is estimated at 0.81 for Sudan Savannah/Guinea Savannah, followed by the Transition zone (0.70), and then the Forest zone (0.55).

Ecology-specific TE for yam is estimated at a mean of 0.54 and has remained relatively constant over the study period (Figure 1, panel b2). Yam TE follows similar patterns as the TGR, the only difference being that the estimate for the Forest zone (0.51) is higher than the Transition zone (0.49) (see Figure 2). The TE values for yam estimated in this study are relatively low when compared to values of 0.85 and 0.89 estimated by Owusu Asante et al. [24] for Ashanti and Brong Ahafo regions, respectively (all approximately located in the Transition zone). Several things distinguish this study from Owusu Asante et al. [24] that could account for the differences in TE. First, our study contains production data from multiple regions/ecologies throughout Ghana and multiple production seasons, while Owusu Asante et al. [24] used data from only 375 yam farmers in one ecology and for only one season. Thus, the values estimated by Owusu Asante et al. [24]

do not holistically capture the spatiotemporal dynamics of yam TE as this study does. Secondly, Owusu Asante et al. [24] utilized data that is over-represented by those farmers (58%) using yam mini-sett technology which the authors show induces positive and significant effects on the TE of some of its adopters. By the nature of its data, this study is not able to pinpoint the specific technologies used by each farmer. However, farmers in this study sample use several technologies which may include yam mini-sett technology.[1]

Accounting for ecology-specific production technologies shows that the mean yam MTE is 0.33. Like the TE, the MTE for yam is also constant across the study period but improves from southern to northern Ghana. Specifically, the most technically efficient yam farmers relative to the industrial production frontier are those in the Sudan Savannah zone, with an MTE of 0.55. Figure 2 shows that Sudan Savannah farmers are followed by their peers in the Guinea Savannah (0.43), the Transition (0.34), and then the Forest (0.24) zones. Even though there is a technological gap of 28%, the overall results for yam show that yam MTE could be enhanced by improving TE.

4.3.3 Cocoyam production

The mean TGR for cocoyam, estimated at 0.80, indicates that the ecological technology gap is about 20%. The temporal trend from Figure 1, panel a3 shows that except for dips in 1991/1992 and again in 2011/2012, the TGR for cocoyam has remained close to its mean value of 0.80 over the study period. This mimics the yam TGR dynamics, but at a lower magnitude. At the ecology level, the TGR for cocoyam is higher in the Forest zone (0.82) than in the Transition zone (0.77).

[1] TE scores are relative measures that track efficiency relative to the most efficient observations within a particular sample. Thus, a caveat is that any difference between this study's estimates and previous works could simply be reflecting differences in heterogeneity across samples.

Ecology-specific TE for cocoyam is estimated at a mean of 0.47 across the entire sample. Unlike the TGR, the cocoyam TE has been declining at a rate of about 5% from 1987 to 2017 (Figure 1, panel b3). Contrary to the TGR, cocoyam TE is higher in the Transition (0.56) than in the Forest (0.48) zone, as depicted in Figure 2. The dynamics in TGR and TE culminate in an estimated mean MTE of 0.36 for cocoyam. This follows the temporal trend exhibited by TE but improves from northern to southern Ghana. The overall results for cocoyam show that production could be enhanced by improving TE.

4.3.4 Plantain production

The mean TGR for plantain (0.85) is second only to cassava among the starchy staples considered. This indicates a plantain technology gap of about 15%. Figure 1, panel a4 shows that the TGR for plantain peaked in the period between 2004/05 and 2012/13 and has generally increased by about 3% annually from 0.72 in 1987 to 0.85 in 2017. At the ecology level, plantain TGR decreases from Transition zone (0.94), and Forest zone (0.87) to the Coastal Savannah zone (0.75). Thus, plantain farmers in the Transition zone are closest to the meta-frontier, while those in the Coastal Savannah zone can increase their productivity by about 25% if they adopt the best available technology.

Ecology-specific TE for plantain is estimated at a mean of 0.54 across the entire sample. The TE for plantain has remained constant over the study period (Figure 1, panel b4). The ecological zone ranking (high to low) of plantain TE is from Coastal Savannah (0.68), Forest (0.58), to the Transition (0.50) zones. In terms of MTE, the mean for plantain is 0.49. The MTE ranking (high to low) for plantain is from Forest (0.51) and Coastal Savannah (0.51) to the Transition zone (0.47). Plantain production could be enhanced by improving TE. The low MTE estimates for plantain production in this study are indicative of what has been reported in the literature. For instance,

MoFA [5] indicated that actual average plantain yields represent only 32% of what is achievable; even the best-performing district attains less than 50% of the estimated potential yield.

4.4 Which factors affect starchy staple technical inefficiency and technology gaps in Ghana?

Table S7 reports the estimates of factors that influence TE and TGR across crops and ecologies. Results for the case of TE are captured by the ecology frontiers and that of the TGR is captured by the meta-frontier. With TE, negative [positive] values indicate that the variables reduce [increase] technical inefficiency. For the case of TGR, negative [positive] values indicate that the variables reduce [increase] the technology gap.

The following caveats are to be noted in interpreting results from the effects of farmer-specific and institutional variables on TE and TGR presented in Table S7. First, these variables were harmonized across different surveys and used mainly as controls in estimating the drivers of differences in starchy staples production outcomes in Ghana. Due to this level of aggregation, we assume only modest exogenous variation in the control variables and therefore do not claim causality. Notwithstanding, given this study's focus on establishing which of technological gaps or technical inefficiencies contribute more to production shortfalls, we believe the outcomes of these farmer characteristics and institutional factors can help to direct policies by examining their qualitative association with the outcomes of interest. To this end, we discuss these variables in terms of general associations and not in terms of individual coefficients.

4.4.1 Farmer Characteristics

Except for yam production in the Sudan Savannah zone, male farmers report higher TE scores than female farmers across crops and ecologies, but sex had no significant effect on the TE of plantain production. Also, the effect of sex on TGR was only limited to yam production where the results showed a technology gap of 0.47 between males and females. These results show that women are

doubly disadvantaged in starchy staples production as they are both less technically efficient and have higher technology gaps when compared to their male counterparts. These phenomena are supported by literature for rice [10], yam [24], and vegetables [23]. Results in Table S7 show that age reduces inefficiencies in starchy staple production. At the national level, these effects are more pronounced in yam production, modest for cassava and plantain, and nonexistent for cocoyam. Thus, age has generally positive associations with TE and technological gaps. The nonlinear effect of age is only present for cassava production. Older farmers tend to be more inefficient in starchy staples production and exhibit wider gaps in adopting modern technologies. Owusu Asante et al. [24] found similar effects of age on yam farmers' technical inefficiency in Ghana's Ashanti and Brong-Ahafo regions. Education also showed non-linear effects only for yam production efficiency but not for the other starchy staples. Mostly, education was negatively associated with technical efficiency and adoption of superior technologies for yam production.

4.4.2 Institutional Factors

Where statistically significant, general results for land ownership show that farm households that own lands are more technically efficient than those farming on rented or communal lands. Land tenure security negatively influences farmers' technical inefficiency for all crops and ecologies except cocoyam farmers in the Transition zone. This highlights the positive effect of tenure security: we would expect landowners to be less risk averse and more incentivized to invest in management practices that improve soil fertility and land structure, and that eventually would lead to more efficient crop production than non-owners of land [10]. On the other hand, the effect of ownership on bridging technology gaps appears inconclusive. On its own, land ownership may not be an enough incentive to adopt and maintain the application of modern technologies. Modern production technologies may require significant extra labor and capital investment to apply. In

such instances, resource-constrained farmers may rather opt for conservative land management techniques that assure a satisfactory level of output.

Access to agricultural extension services often plays a key role in helping farmers improve their crop production by promoting good agronomic practices and the adoption of better technology that improves efficiency. Our results confirm this *a priori* expectation by their generally negative associations with TE and TGR. Access to extension services reduced both technical inefficiency and technology gaps of yam and plantain production, but these effects were insignificant for cassava and cocoyam. As in this study, Owusu Asante et al. [24] also found that extension access reduced production inefficiency. Similarly, Egyir et al. [38] found that access to extension services increased plantain farmers' adoption of agro-chemicals which ultimately improved yields.

Access to credit is expected to enable farmers to acquire supporting inputs necessary to increase efficiency and productivity. The results in Table S7 show generally positive associations with both TE and TGR. Access to production credit increased both technical inefficiencies and technology gaps for cassava and yam production, but reduced technology gaps for plantain production. This counterintuitive finding on the negative effect of credit on TE and TGR is difficult to explain from the available data, but a possible prediction relates to the exact use of the accessed credit amounts. Do farmers invest credit funds in their starchy staple production? Oftentimes, farmers use the credit for other purposes than farm investment [39].

Access to mechanized services showed heterogeneous effects on starchy staple production. While mechanized services increased the technology gaps for plantain and cassava, it reduced technical inefficiency and technology gaps for yam production. This mixed result is not surprising and may be reflective of the heterogenous access to mechanized services across production ecologies and

among farmers in Ghana [40]. That said, we are mindful of claiming too much inference to this finding given the conditions.

5. Discussion

The literature identifies technical inefficiencies and technology gaps as the main causes of low productivity in smallholder agricultural systems. Our discussion is tilted towards addressing the main question of the paper, which is to ascertain whether starchy staples production shortfalls in Ghana are predominantly driven by technical inefficiencies, technological gaps or both. As indicated, the technological gap depends on farmers' access to and adoption of the best production technology and shows how close the production technology in an ecology is to the best technology set. On the other hand, technical efficiency is driven by the ability and willingness of the farmer to properly use and combine the available input sets. Thus, management practices and enabling environment as opposed to new technologies, are key determinants of technical efficiency.

The results in Table 4 show that for cassava, yam, and plantain, agroecological technologies in northern Ghana tend to be closer to the meta-frontier technology indicating low technological gaps; however, the technology gaps increase consistently as one traverses southwards. Unlike the technological gaps, technical inefficiency scores for the various agro-ecologies did not seem to follow any pattern across space or time. Acheampong et al. [41] examined cocoyam farmers' rates of adopting improved technologies and found that farmers in the Transition zone had higher adoption rates than those in the Forest zone. Their results further showed that the high adoption of complementary production techniques like appropriate planting distance, timely weed control, and weedicide application in the Transition zone improved the TGR for that zone.

The MTE provides a basis for comparing the efficiency of starchy staple production for all agro-ecologies relative to the best available technology. The key observation from these results is that the technical inefficiency effects across all ecologies, as well as at the national level, outweigh the technological gap effects. Thus, even though both technical inefficiency and technological gaps exist across all ecologies, technical inefficiencies are always higher than technological gaps. These results suggest more room for improving the efficiency of producing all crops in all agro-ecologies. For example, while cassava production in the Guinea Savannah zone is the most efficient relative to the meta-frontier, production levels can still be increased by about 40% if the inefficiency effects are eliminated, given the prevailing input levels and production technologies. What could be the possible explanation for the trends we observe, where technical inefficiency effects tend to outweigh the technological gaps?

Over the years, the interest of policies in many developing countries, including Ghana, has been to improve production levels mainly through the introduction of new technologies or input subsidies [42,43] while less attention is given to mechanisms that can increase the efficiency of production. For instance, while Ghana cocoa sector has benefited from several fertilizer subsidy schemes, it is well documented that inappropriate use of the subsidized fertilizer in terms of timing and application rates was a common production problem [44]. It has also been found that providing subsidized fertilizer in addition to training on best management practices via farmer field schools results in relatively more benefits in terms of increased yields than providing subsidized fertilizer alone [45]. These observations suggest that increasing farmer access to improved/new technology (improved crop varieties, fertilizer, etc..,) solves only one part of the problem: human capital investments and training on proper technology usage are also needed. However, such add-on outreach activities often are not the focus of most programs or they get sidelined during the

implementation stages of government policies (as was the case of the Root and Tuber Improvement Program [RTIP] (1999-2004)] and its successor, Root and Tuber Improvement and Marketing Program [RTIMP] (2005-2015) [46,47]. The focus of the RTIP was on the multiplication and distribution of improved planting materials and integrated pest management, with less attention given to complementary mechanisms that increase technical efficiency.

A closer look at the performances of individual starchy staples reinforces the roles of policies and complementary inputs in efficient crop production. From the MTE scores, the order of best performing starchy staples were cassava, plantain, cocoyam, and yam. The higher efficiency scores for cassava might be attributed to the prominent attention it received in policies and programs such as the President's Special Initiative (PSI) on agribusiness and cassava (2002-2004), the RTIP and RTIMP, as well as the West Africa Agricultural Productivity Program [WAAPP] (2007-2017) that aimed at increasing productivity of starchy staples in Ghana [54].

The finding that yam is the most inefficiently produced starchy staple in Ghana is worrying for several reasons. Ghana is the world's second-largest producer of yam (behind Nigeria) but the leading exporter to the global market [48,49]. Given that about 94% of yam produced in Ghana is destined for the international market, addressing the causes of production shortfalls has the potential to improve the livelihoods of actors in the yam value chain. At the local levels, yam holds keystone positions in the cultural expression of several tribes in Ghana [50,51] and remains an essential food security crop. For instance, yam consumption per capita stood at 125kg in 2015 [49]; however, about 40% of yam harvested in the Guinea Savannah zone (the largest yam producing area) is lost through poor postharvest handling [48]. This, coupled with resource use inefficiencies, threatens the capacity of yam to contribute to achieving Ghana's SDG 2 targets.

The finding that persistent technical inefficiency is relatively more of a challenge to productivity improvement than technology gaps across all starchy staples considered in this study means that critical attention must be given to the enabling factors that foster productivity growth rather than searching for new technologies all the time. Already, smallholder farmers are risk averse, which affect their production decisions and adoption of new technologies [52]. Therefore, continuously exposing these farmers to new technologies without first improving the enabling environment that foster their adoption may be counterproductive. Studies have found that management and institutional variables such as extension services are important drivers of technology adoption and productivity [38,53]. Our results in Table S7 support this assertion, as we found that both TE and TGR for some starchy staples are significantly affected by access to extension services.

6. Conclusions

In developing countries, many farmers unwittingly waste significant productive resources through production and technological inefficiencies which entrench poverty and food insecurity. Changing the status quo and fostering rapid economic transformation would require sustained improvements in agricultural productivity. In this study, we take advantage of a large Ghanaian dataset that spans over three decades and examine the spatiotemporal trends of production gaps of one of the most important staple crop categories, the starchy staples. Unlike similar studies thus far, and in addition to the unique dataset used, the method adopted accounts for technological heterogeneity across the main ecological zones in Ghana where these starchy staple crops are cultivated. Using the stochastic meta-frontier approach, technical efficiency, meta-technical efficiency, and technology gap ratios are measured across crops and ecological zones. This analysis enables us to provide a broad understanding of the major sources of production shortfalls within the starchy staples industry of Ghana.

The key findings suggest that there are significant shortfalls in starchy staples production, which could be attributed to pure farmer technical inefficiencies and less to technology gaps along agroecological lines. The results show that the current set of production technologies used by starchy staple farmers in Ghana across ecologies are quite similar as the ecological technology gap that exists is about 20% for all crops except yam which has a technology gap of 35%. On the other hand, there exist significant production shortfalls that could be attributed to pure farmer technical inefficiencies. Respectively, about 35, 46, 53, and 45% of cassava, yam, cocoyam, and plantain production is lost due solely to technical inefficiencies and these have been relatively constant or increasing from 1987 to 2017. These trends suggest that mechanisms that improve the efficiency of resource use among starchy staple farmers have not received the needed attention over time. With the large production shortfalls attributable mostly to technical inefficiency, one implication of our results is that the starchy staple crop sector could benefit from improvements in conditions that enhance farmer technical efficiencies, such as providing starchy staple farmers more access to complementary production inputs such as extension services and better agronomic practices.

Further, the results also show that for any given starchy staple crop, some ecological zones perform better than others, thereby indicating heterogeneity in both technology and production management practices. We generally find that across all the crops considered, the technology gap across ecological lines is minimal but pure farmer technical inefficiency is chronic. This means that the production shortfalls for starchy staples in Ghana could be reduced if the causes of pure farmer technical inefficiency are adequately addressed. Existing policies aimed at improving the technology level (e.g., those supplying improved planting materials and subsidized inputs) could also emphasize the efficient use of these technologies. Stakeholders could learn important lessons from the frontier enhancing technologies and practices in high-performing ecological zones,

improve them further and disseminate such improved technologies to less-performing agro-ecologies, guided, of course, by contextual factors.

References

1. FAO, IFAD, UNICEF, WFP W. The State of Food Security and Nutrition in the World 2022. FAO; 2022. doi:10.4060/cc0639en
2. GSS/MOFA/WFP/FAO. Comprehensive Food Security and Vulnerability Analysis (CFSVA) GHANA. 2020 [cited 13 Mar 2023]. Available: https://docs.wfp.org/api/documents/WFP-0000137744/download/
3. FAO. The State of Food and Agriculture 2022. FAO; 2022. doi:10.4060/cb9479en
4. Pradhan P, Fischer G, van Velthuizen H, Reusser DE, Kropp JP. Closing Yield Gaps: How Sustainable Can We Be? Gonzalez-Andujar JL, editor. PLoS One. 2015;10: e0129487. doi:10.1371/journal.pone.0129487
5. Ministry of Food and Agriculture [MOFA]. Agriculture in Ghana: Facts and figures (2018). 2019.
6. Ladha JK, Jat ML, Stirling CM, Chakraborty D, Pradhan P, Krupnik TJ, et al. Achieving the sustainable development goals in agriculture: The crucial role of nitrogen in cereal-based systems. Advances in Agronomy. 2020. pp. 39–116. doi:10.1016/bs.agron.2020.05.006
7. Abdulai A, Huffman W. Structural Adjustment and Economic Efficiency of Rice Farmers in Northern Ghana. Econ Dev Cult Change. 2000;48: 503–520. doi:10.1086/452608
8. Anang BT, Bäckman S, Sipiläinen T. Technical efficiency and its determinants in smallholder rice production in northern Ghana. J Dev Areas. 2016;50: 311–328. doi:10.1353/jda.2016.0072
9. Donkor E, Matthews N, Ogundeji AA. Efficiency of Rice Farming in Ghana: Policy Implications for Rice Sector Development. African Dev Rev. 2018;30: 149–161. doi:10.1111/1467-8268.12320
10. Owusu V, Donkor E, Owusu-Sekyere E. Accounting for the Gender Technology Gap Amongst Smallholder Rice Farmers in Northern Ghana. J Agric Econ. 2018;69: 439–457. doi:10.1111/1477-9552.12236
11. Asravor J, Wiredu A, Siddig K, Onumah E. Evaluating the Environmental-Technology Gaps of Rice Farms in Distinct Agro-Ecological Zones of Ghana. Sustainability. 2019;11: 2072. doi:10.3390/su11072072
12. Tsiboe F, Asravor J, Owusu V, Mensah-Bonsu A. Production technology, efficiency, and productivity of cereal farms: Prospects for enhancing farm performance in Ghana. Agric Resour Econ Rev. 2022; 1–31. doi:10.1017/age.2022.16

13. Abdulai S, Nkegbe PK, Donkoh SA. Technical efficiency of maize production in Northern Ghana. African J Agric Research. 2013;8: 5251–5259. doi:10.5897/AJAR2013.7753

14. Owusu V. Technical Efficiency Of Technology Adoption By Maize Farmers In Three Agro-Ecological Zones Of Ghana. Rev Agric Appl Econ. 2016;19: 39–50. doi:10.15414/raae.2016.19.02.39-50

15. Wongnaa CA, Awunyo-Vitor D. Scale efficiency of maize farmers in four agro ecological zones of Ghana: A parametric approach. J Saudi Soc Agric Sci. 2019;18: 275–287. doi:10.1016/j.jssas.2017.08.003

16. Avea A, Zhu J, Tian X, Baležentis T, Li T, Rickaille M, et al. Do NGOs and Development Agencies Contribute to Sustainability of Smallholder Soybean Farmers in Northern Ghana—A Stochastic Production Frontier Approach. Sustainability. 2016;8: 465. doi:10.3390/su8050465

17. Awunyo-Vitor D, Bakang JA, Gyan SK, Cofie S. Estimation of Farm Level Technical Efficiency of Small-Scale Cowpea Production in Ghana. Am J Agric Environ Sci. 2013;13: 1080–1087. doi:10.5829/idosi.aejaes.2013.13.08.11013

18. Etwire PM, Martey E, Dogbe W. Technical Efficiency of Soybean Farms and Its Determinants in Saboba and Chereponi Districts of Northern Ghana: A Stochastic Frontier Approach. Sustain Agric Res. 2013;2: 106. doi:10.5539/sar.v2n4p106

19. Onumah JA, Onumah EE, Al-Hassan RM, Brümmer B. Meta-frontier analysis of organic and conventional cocoa production in Ghana. Agric Econ (Czech Republic). 2013.

20. Besseah FHFA, Kim S. Technical Efficiency of Cocoa Farmers in Ghana. J Rural Dev. 2014;37: 159–182. Available: http://ageconsearch.umn.edu/record/196615?ln=en

21. Danso-Abbeam G, Baiyegunhi LJS. Technical efficiency and technology gap in Ghana's cocoa industry: accounting for farm heterogeneity. Appl Econ. 2019; 1–13. doi:10.1080/00036846.2019.1644439

22. Tsiboe F. Chronic sources of low cocoa production in Ghana: new insights from meta-analysis of old survey data. Agric Resour Econ Rev. 2021; 1–26. doi:10.1017/age.2021.3

23. Tsiboe F, Asravor J, Osei E. Vegetable Production Technical Efficiency and Technology Gaps in Ghana. African J Agric Resour Econ. 2019;14. Available: http://afjare.org/wp-content/uploads/2020/01/3.-Tsiboe-et-al.pdf

24. Owusu Asante B, Villano RA, Battese GE. The effect of the adoption of yam minisett technology on the technical efficiency of yam farmers in the forest-savanna transition zone of Ghana. African J Agric Resour Econ. 2014.

25. Tsiboe F, Aseete P, Djokoto JG. Spatiotemporal Comparison of Dry Beans and Groundnut Production Technology and Inefficiency in Ghana. Rev Agric Appl Econ. 2021;24: 76–87. doi:10.15414/raae.2021.24.01.76-87

26. Battese GE, Rao DSP, O'Donnell CJ. A Metafrontier Production Function for Estimation of Technical Efficiencies and Technology Gaps for Firms Operating Under Different Technologies. J Product Anal. 2004;21: 91–103.

doi:10.1023/B:PROD.0000012454.06094.29

27. Adaku AA, Tsiboe F, Clottey J. Taking stock of gender gaps in crop production technology adoption and technical efficiency in Ghana. Agrekon. 2023; 1–18. doi:10.1080/03031853.2022.2150664

28. Huang CJ, Huang T-H, Liu N-H. A new approach to estimating the metafrontier production function based on a stochastic frontier framework. J Product Anal. 2014. doi:10.1007/s11123-014-0402-2

29. World Bank. World Development Indicators. In: World DataBank [Internet]. 2018 [cited 24 Sep 2018] p. 136. Available: https://data.worldbank.org/products/wdi

30. Institute of Statistical Social and Economic Research (ISSER), Economic Growth Center (EGC). Ghana - Socioeconomic Panel Survey: 2009-2010. In: Microdata Library. GHA_2009_GSPS_v01_M [Internet]. 2016 [cited 9 Nov 2018]. Available: http://microdata.worldbank.org/index.php/catalog/2534

31. Tsiboe F. Nationally Representative Farm/Household Level Dataset on Crop Production in Ghana from 1987-2017. In: SSRN [Internet]. 2020. doi:10.2139/ssrn.41345181

32. Ghana Statistical Service [GSS]. 2010 Population and Housing Census Summary Results of Final Report. Accra,Ghana; 2012. Available: www. statsghana.gov.gh

33. Asante BO, Temoso O, Addai KN, Villano RA. Evaluating productivity gaps in maize production across different agroecological zones in Ghana. Agric Syst. 2019;176: 102650. doi:10.1016/j.agsy.2019.102650

34. Bokusheva R, Hockmann H. Production risk and technical inefficiency in Russian agriculture. Eur Rev Agric Econ. 2005;33: 93–118. doi:10.1093/erae/jbi036

35. Van Nguyen Q, Pascoe S, Coglan L, Nghiem S. The sensitivity of efficiency scores to input and other choices in stochastic frontier analysis: an empirical investigation. J Product Anal. 2021;55: 31–40. doi:10.1007/s11123-020-00592-8

36. Coelli T. Estimators and hypothesis tests for a stochastic frontier function: A Monte Carlo analysis. J Product Anal. 1995;6: 247–268. doi:10.1007/BF01076978

37. Gutierrez RG, Carter S, Drukker DM. On boundary-value likelihood-ratio tests. Stata Tech Bull. 2001;10. Available: https://econpapers.repec.org/article/tsjstbull/y_3a2001_3av_3a10_3ai_3a60_3asg160.htm

38. Egyir IS, Owusu- Benoah E, Anno- Nyako FO, Banful B. Assessing the factors of adoption of agrochemicals by plantain farmers in Ghana. McKague K, editor. J Enterprising Communities People Places Glob Econ. 2011;5: 83–97. doi:10.1108/17506201111119617

39. Appiah-Twumasi M, Donkoh SA, Ansah IGK. Farmer innovations in financing smallholder maize production in Northern Ghana. Agric Financ Rev. 2019;80: 421–436. doi:10.1108/AFR-05-2019-0059

40. Appiah-Twumasi M, Donkoh SA, Ansah IGK. Innovations in smallholder agricultural financing and economic efficiency of maize production in Ghana's northern region.

Heliyon. 2022;8: e12087. doi:10.1016/j.heliyon.2022.e12087

41. Acheampong P, Osei-Adu J, Amengor NE, Sagoe R. Cocoyam value chain and benchmark study in Ghana. In: A report submitted to West African Agricultural Productivity Project (WAAPP). Project Information Document. 2014.

42. Houssou N, Andam KS, Collins A-A. Can better targeting improve the effectiveness of Ghana's Fertilizer Subsidy Program?: Lessons from Ghana and other countries in Africa south of the Sahara. In: IFPRI Discussion Paper 1605. Washington, D.C.: International Food Policy Research Institute (IFPRI) [Internet]. 2017 [cited 9 May 2017]. Available: http://ebrary.ifpri.org/cdm/ref/collection/p15738coll2/id/131068

43. Tsiboe F, Egyir IS, Anaman G. Effect of fertilizer subsidy on household level cereal production in Ghana. Sci African. 2021;13: e00916. doi:10.1016/j.sciaf.2021.e00916

44. Opoko E, Dzene R, Caria S, Teal F, Zeitlin A. Impacts of Group based Microfinance in Agriculture: Evidence from Ghana's Cocoa Abrabopa Association. In: Annual conference of Center for the Study of African Economies (CSAE) 2009 [Internet]. Oxford,United Kingdom: University of Oxford; 2009. Available: http://www.csae.ox.ac.uk/conferences/2009-edia/papers/473-CariaTealZeitlin.pdf

45. Tsiboe F, Dixon BL, Nalley LL, Popp JS, Luckstead J. Estimating the impact of farmer field schools in sub-Saharan Africa: the case of cocoa. Agric Econ. 2016;47: 329–339. doi:10.1111/agec.12233

46. International Fund for Agricultural Development [IFAD]. Republic of Ghana: Root and Tuber Improvement Programme (RTIP). In: Interim Evaluation Report October 2004. Report N° 1533-GH. Independent Office of Evaluation. International Fund for Agricultural Development, West and Central Africa Division Programme Management Department. [Internet]. 2004 [cited 13 Mar 2023]. Available: file:///C:/Users/ftsib/Downloads/gh_rtip.pdf

47. International Fund for Agricultural Development [IFAD]. Republic of Ghana: Root and Tuber Improvement and Marketing Programme RTIMP Project Performance Evaluation Report. In: July 2018 Report No. 4813-GH. Independent Office of Evaluation. International Fund for Agricultural Development, West and Central Africa Division Programme Management Department. [Internet]. 2018 [cited 13 Mar 2023]. Available: https://www.ifad.org/documents/38714182/40811021/ghana_1312_2018.pdf/6e2a36e1-c84b-452d-9747-d84ac8d6c27a

48. Ansah IGK, Tetteh BKD. Determinants of Yam Postharvest Management in the Zabzugu District of Northern Ghana. Adv Agric. 2016;2016: 1–9. doi:10.1155/2016/9274017

49. Acheampong P, Owusu Danquah E, Dissanayake H, Hayford P, Weebadde C. A Socioeconomic Study of Transition Zone Yam Farmers Addressing Constraints and Exploring Opportunities for Integrating Pigeonpea into Yam Cropping Systems. Sustainability. 2019;11: 717. doi:10.3390/su11030717

50. Odotei I. Festivals in Ghana: Continuity, transformation and politicisation of tradition. Trans Hist Soc Ghana. 2002;6.

51. Ogundeji PA, Fasehun MA. Insights from Festivals and Carnivals. The Palgrave Handbook of African Oral Traditions and Folklore. Cham: Springer International Publishing; 2021. pp. 105–118. doi:10.1007/978-3-030-55517-7_5

52. Ogada M, Wilfred N, And Mahmud Y. Production risk and farm technology adoption in the rain-fed semi-arid lands of Kenya. Afjare 2010. 2010.

53. Mariano MJ, Villano R, Fleming E. Factors influencing farmers’ adoption of modern rice technologies and good management practices in the Philippines. Agric Syst. 2012;110: 41–53. doi:10.1016/j.agsy.2012.03.010

Click here to access/download

Supporting Information

Online Appendix S - Supplementary materials.docx

Starchy staples production shortfalls in Ghana: Technical inefficiency effects outweigh technological differences across ecologies

Isaac Gershon Kodwo Ansah [a*], ~~Francis Tsiboe[b],~~ Mark Appiah-~~Twumasi[c]~~Twumasi[b], Francis Tsiboe[c]

[a] Department of Economics, School of ~~Applied~~ Economics ~~and Management Sciences~~, University for Development Studies, Tamale, Ghana.

~~[b] USDA, Economic Research Service, 805 Pennsylvania Avenue, Kansas City, MO 64105, USA~~

~~[c]~~[b] Department of Agricultural and Food Economics, Faculty of Agriculture, Food and Consumer Sciences, University for Development Studies, Tamale, Ghana.

[c] USDA, Economic Research Service, 805 Pennsylvania Avenue, Kansas City, MO 64105, USA

* Corresponding author at: Department of Economics, School of ~~Applied~~ Economics ~~and Management Sciences~~, University for Development Studies, Tamale, Ghana. E-mail address: agershon@uds.edu.gh (Isaac Gershon Kodwo Ansah) (ORCID ID: 0000-0001-5071-6224)

Formatted: Left, Line spacing: single

Formatted: Not Superscript/ Subscript

Data Availability: Replication materials are available on GitHub at https://github.com/ftsiboe/Agricultural-Productivity-in-Ghana

Funding: The authors did not receive any funding for this manuscript.

Acknowledgments:

The authors gratefully acknowledge the Ghana Statistical Service, Institute of Statistical Social and Economic Research (ISSER), Economic Growth Center, and the International Food Policy Research Institute (IFPRI) for making the dataset available for the study. All errors are the responsibility of the authors. The findings and conclusions in this publication are those of the authors and should not be construed to represent any official USDA or U.S. Government determination or policy. This research was supported in part by the U.S. Department of Agriculture, Economic Research Service.

Abstract

~~This paper assesses the production shortfalls of major starchy staple crops in Ghana using a meta-frontier efficiency analysis.~~Starchy staples are a major source of livelihood support for farmers, traders, and processors who participate in these crops' value chains, while also providing staple food to many people, especially the less affluent in society. Despite this position, the productivity figures of starchy staples are low. We use a unique data set and meta-frontier efficiency analysis to assess whether the production shortfalls of major starchy staple crops in Ghana could be attributed to technical inefficiency, technology gaps or both. Results show strong evidence of about 50% production shortfall for cassava, yam, cocoyam, and plantain. ~~The~~For cassava production, the Guinea Savannah zone has the most superior technology, with a technology gap ratio of 0.92, while yam production is more technically efficient in the Sudan Savannah zone, with a technical efficiency score of 0.67. Cocoyam production is more technically efficient (0.56) in the Transition zone but yam is more technically efficiently produced in the Coastal Savannah zone of Ghana. These results show that production shortfall is ~~further shown to be~~ more influenced by pure farmer technical inefficiencies (about 45%) rather than by technology gaps (~~12~~about 20%) along ecological lines. Thus, the sector could benefit from improvements in farmer managerial skills and efficient use of existing technologies.

Formatted: Comment Text

Formatted: Font: 12 pt

Formatted: Font: 12 pt

Formatted: Font: 12 pt

Formatted: Font: 12 pt

Formatted: Font: 12 pt

Keywords: starchy staples; technological gap ratio; technical inefficiency; Ghana

JEL codes: D24, Q18, Q12.

Starchy staples production shortfalls in Ghana: Technical inefficiency effects outweigh technological differences across ecologies

1. Introduction

In ~~2018~~2022, the Food and Agriculture Organization's document on the State of Food Security and Nutrition ~~Security~~ in the ~~world reported~~World projected that ~~the number of undernourished people globally has worsened, rising from 804~~between 702 and 828 million people faced hunger in ~~2016 to 821 million, an equivalence of one out~~2021, with Africa and Asia being the main hosts of these challenges [1]. In Ghana, a report by GSS/MOFA/WFP/FAO [2] indicate that 11.7% of ~~every nine persons in the world [1].~~Ghana's population (3.6 million people) were food insecure in 2020. Out of these, 5.2% were severely food insecure, and 6.5% were moderately food insecure; also, 78% of the food insecure were in rural areas. The report further notes that there are regional heterogeneities in the distribution of food insecurity in Ghana, with most food insecure people living in the Guinea Savannah and Deciduous Forest zones. In explaining the possible causes of these rising food insecurity challenges, the FAO [1] report ~~identifies~~identified poor access to food due to low domestic production, poor distribution and high cost of imports, among other factors~~,~~ ~~with Africa and Asia being the main hosts of these challenges.~~. Since the primary source of food in these areas, Africa especially, is domestic production, this~~, coupled with lessons from border closures due to COVID-19,~~ creates the urgent need to examine the pathways through which food is made available and accessible locally to the vulnerable segment of the population.

Formatted: English (South Africa)

Formatted: English (South Africa)

In many parts of the developing world, smallholder farmers mostly organize the production of staples. The ~~2017~~2022 FAO report on "The State of Food and Agriculture" point to low agricultural productivity as characterizing smallholder production systems ~~[1]. Productivity~~[3]. Based on global projections, Pradhan et al. [4] conclude that Africa has the highest crops yield

gaps, and that closing these gaps has the potential to improve food and nutrition security in Africa. Thus, productivity growth and resource use efficiency are important causal pathways to increased food production and access for the poor in these areas. Notably, Pradhan et al. [4] attribute the wide yield gaps in crops, especially those cultivated under rainfed, to biophysical factors (e.g., soil quality, agro-climatic, etc.) and socioeconomic factors (e.g., weather-induced yield variability that affect risk attitudes, market access, etc.). Besides direct food availability, agro-industrial development rests so much on the availability of raw materials to supply industrial needs. How could the industrial need be met if what is available cannot satisfy direct consumption demand? Therefore, productivity growth in these crops is crucial to foster rapid economic transformation in the developing world.

The production of cassava (*Manihot Esculentaesculenta*), cocoyam (*Colocasia Esculentaesculenta*), plantain (*Musa Sapientumsapientum*) and yam (*Dioscorea Alataalata*) (henceforth called starchy staples) plays important roles in the Ghanaian economy. Starchy staples are a major source of livelihood support for farmers, traders, and processors who participate in these crops' value chains, while also providing staple food to many people, especially the less affluent in society. Despite this position, the productivity figures of starchy staples are low. There are non-ignorable productivity gaps between actual and potential yields. In 2018 the national average yieldyields of cassava, cocoyam, plantain, and yam waswere all over 50% lower than their potential yields, which were estimated at 45Mt/ha for cassava, 38Mt/ha for plantain, 52Mt/ha for yam, and 20Mt/ha for cocoyam [2].[1][5].

[1] Potential yield are the expected yields of crops published by Ghana's Ministry of Food and Agriculture. They are usually obtained through field experiments by agronomists/crop scientists where all the technical conditions needed

These yield gaps ultimately culminate in production shortfalls which tend to vary widely across regions and ecological zones in Ghana. Such variations indicate that the ~~yield gaps~~production shortfalls might be location-specific, crop-specific, and farmer-specific. The large ~~yield gaps~~production shortfalls generate costs to the country in terms of wasted resources amid scarcity as well as creating environmental challenges. ~~These productivity gaps, therefore, border on the efficiency of resource utilization which would require attention.~~These production shortfalls, therefore, border on the efficiency of resource utilization (e.g., Ladha et al., [6]) which would require attention. Resource use efficiency, in the layman's understanding, means *doing more with less*. A transition from doing less with more (inefficiency of resource utilization) to doing more with less (efficiency of resource utilization) allows the creation of more economic value with less natural, human, and man-made resources, and eventually enhances household welfare. How can this be achieved if we have limited knowledge of what drives these production shortfalls, or their nature and distribution thereof?

Several studies relevant to Ghanaian agriculture have investigated the primary drivers of resource use efficiency for rice ~~[3–8], maize [8–11], legumes [12–14], cocoa [15–17], and vegetables [18]. However, an important category of crops that has received less attention despite the important role they play in the food security of households is the starchy staples. A few studies like [19] analyzed starchy staples data for only one season, and they did not consider the possibility of heterogeneous technology adoption within Ghana. Like their peers cultivating vegetables [18], cereals [7,8,10], and legumes [20], starchy staples farmers could be operating under different technologies~~

~~for optimum performance of crops are provided. In 2019, these were estimated at 45Mt/ha, 38Mt/ha, 52Mt/ha, and 20Mt/ha for Cassava, Plantain, Yam, and Cocoyam respectively [2].~~

constrained by the ecology and/or enabling environment. According to the literature, failure to account for these technological differences could lead to falsely attributing production shortfalls due to technology gaps to technical inefficiency [21][7–12], maize [12–15], legumes [16–18], cocoa [19–22], and vegetables [23], but with little attention on production shortfalls. An important category of crops that has received less attention despite the important role they play in the food security of households is the starchy staples. A few studies like [24] analyzed starchy staples data for only one season, paying no attention to the possibility of heterogeneous technology adoption within Ghana. Like their peers cultivating vegetables [23], cereals [11,12,14], and legumes [25], starchy staples farmers could be operating under different technologies constrained by the ecology and/or enabling environment. According to the literature, failure to account for these technological differences could lead to falsely attributing production shortfalls due to technology gaps to technical inefficiency [26].

In the last two decades, research on productivity and efficiency analyses in Ghana is highly skewed toward cereal crops. In principle, the starchy staples have received little attention, against the backdrop of the critical roles these crops play, and the significant yield gaps observed in practice. Except for a few studies [8,17,18,20,22], all efficiency studies in Ghana used data based on a single season, and data size is limited to a few villages or at best a region. This data limitation and lack of locational heterogeneity masks important lessons that could emerge from analyzing a more comprehensive and heterogeneous sample. For example, knowledge on whether technology gaps across ecologies or resource use inefficiencies among farmers contribute most to production shortfalls and how these evolve is not yet clear. Such incomplete information might limit scientific progress, and serve as a barrier to societal development, in the sense that policymakers may be

inadequately or wrongly informed. Focusing on starchy staples, this study addresses most of these limitations by accommodating multiple seasons and heterogeneity in ecological technology gaps.

Particularly, this study applies Huang et al. [23]In the last two decades, research on productivity and efficiency analyses in Ghana is highly skewed toward cereal crops. In principle, the starchy staples have received little attention, against the backdrop of the critical roles these crops play, and the significant yield gaps observed in practice. Except for a few studies [12,22,23,25,27], all efficiency studies in Ghana used data based on a single season, and data size is limited to a few villages or at best a region. This data limitation and lack of locational heterogeneity masks important lessons that could emerge from analyzing a more comprehensive and heterogeneous sample. For example, knowledge on whether technology gaps across ecologies or resource use inefficiencies among farmers contribute most to production shortfalls and how these evolve is not yet clear. Such incomplete information might limit scientific progress, and serve as a barrier to societal development, in the sense that policymakers may be inadequately or wrongly informed. Focusing on starchy staples, this study addresses most of these limitations by accommodating multiple seasons and heterogeneity in ecological technology gaps.

Particularly, this study applies Huang et al. [28] method of estimating meta-stochastic-frontier to a sample of 15,420 starchy staple farmers drawn from nine cross-sectional population-based surveys periodically fielded throughout Ghana from 1987 to 2017. The aim is to examine whether production shortfalls are primarily driven by resource use efficiency among farmers or due to technological gaps across ecological zones in Ghana, as well as their nature and distribution thereof. The pooled data has the broadest coverage of starchy staple households across time and space than previous studies conducted on Ghana. As such, it presents a unique opportunity for

utilizing the meta-stochastic frontier to ascertain the ecological and temporal dynamics of starchy staple resource use efficiency in Ghana. ~~This is~~

Our results show significant production shortfalls in starchy staples in Ghana, which could be attributed more to pure farmer technical inefficiencies rather than technology gaps. The production technologies used by starchy staple farmers are quite similar across ecologies, but some ecological zones perform better than others. The chronic pure farmer technical inefficiency effects suggest that the production shortfalls could be reduced by addressing the causes of inefficiency and improving the efficient use of existing technologies. These results are important because the achievement of Sustainable Development Goals 2.1 (ensuring access to food for all) and 2.2 (ending all forms of malnutrition) would, at least partly, require the realization of Sustainable Development Goal 12, which leverages the efficient use of natural resources to create a circular economic growth [~~24~~29].

Field Code Changed

~~**2. Productivity and Efficiency Literature**~~

~~Globally, the methods for productivity and efficiency analyses have witnessed a clear evolution since the seminal work of Farrell [25]. From this evolution, agricultural productive efficiency has generally been grouped into technical or allocative, with the two combining to form economic efficiency. Technical Efficiency (TE), the focus of this study, is the ability of a (farm) firm to obtain the best possible output based on an optimal set of inputs. TE can be measured from an output orientation, where observed output is compared to the potential output given the input set. TE can also be measured from an input orientation where observed input levels are compared to the minimum input required to produce a given output level [26]. Both orientations can be implemented using either Stochastic Frontier Analysis (SFA) [27,28] or Data Envelopment Analysis (DEA) [29,30].~~

The two major differences between DEA and SFA are that, unlike the latter, the former does not impose a functional form on the data and assumes that output variability is deterministic as it is solely due to inefficiency. By not accounting for idiosyncratic shocks, the implied production frontier of DEA is susceptible to outliers and measurement error and has no statistical properties for inferences. Nonetheless, there has been significant progress made in dealing with the limitations of DEA regarding statistical properties and testing via bootstrapping. For the SFA, its main limitation has been the imposition of a functional form on the production process. The distribution for the inefficiency term also sometimes comes with a few challenges. However, the SFA accommodates idiosyncratic shocks and therefore makes the model less susceptible to outliers and measurement errors. With a well-specified functional form, SFA allows the researcher to undertake statistical inferences. In the Ghanaian context, while both SFA and DEA have been applied in empirical studies, SFA dominates mainly due to the unavailability of reliable input price data that is often required by DEA. In this study, reliable price data is also not available, hence we utilize the output-oriented SFA approach.

In the literature, SFA is the more popular choice for estimating the efficiency of smallholder agricultural production systems. In Africa, this method has been applied mainly for studies on cereals and legumes. Morse [31] and Ojo and Ayanwale [32] have examined the efficiency of starchy staples production using the SFA. A meta-analysis by Morse [31] on the technical efficiency of yam production in Nigeria revealed an average technical efficiency between 0.6 and 0.8. The study further recommended enhancing farmers' knowledge of input application, instead of intensifying application, as the major policy initiative for reducing technical inefficiency among farmers in Nigeria. On the other hand, Ojo and Ayanwale [32] attempted to determine the credit amount needed to bridge the inefficiency gap in plantain production by combining a Cobb-Douglas

SFA, the Harrod-Domar growth equation, and Heckman selection model to control selectivity bias in credit access. They reported a mean TE of 0.53 and a financing gap of US$263 per growing season.

Cassava production is one of Africa's most significant crop enterprises, contributing significantly to food security and the supply of raw materials for agro-industrial use in Africa and her trading partners. Nigeria is the leading global producer of cassava followed by Thailand and Brazil, but the top ten cassava producers are exclusively made of countries in the global south [33]. Hence, cassava has received the most attention of the four starchy staples in the production efficiency literature. Studies on cassava production tend to prefer other methods to the SFA in estimating its efficiency. Ogundari and Brümmer [34] justify their choice of a primal output distance function on the high level of intercropping in cassava fields in Nigeria. Rahman and Awerije [35] opted for a two-stage DEA in their study because of its ability to estimate efficiency at the production and processing stages while Fu, Qu, and Pan [36] opted for an output-oriented DEA. These studies commonly admit that cassava yields have stagnated lately due to the discontinuity of government policy by succeeding administrations and the saturated adoption of technologies by farmers.

In Ghana, most of the literature focused on applications of the SFA to cross-sectional samples of cereals, legumes, and cocoa farmers. The samples for these studies have largely been limited to districts or at best a region and for one production season. Notably, Abdulai and Huffman [3] examined how the Structural Adjustment Programme influenced the economic efficiency of rice production in northern Ghana based on a cross-section of 120 northern rice farmers for the 1992/1993 growing season. Using the Translog SFA and ordinary least squares (OLS) regression, the authors reported profit losses of 27% due to inefficiency in domestic rice production. Owusu Asante et al. [19] examined how the adoption of improved yam production technology affected

farmers' TE in Ghana's Transitional zone. Using 375 farmers from the Ashanti and Brong-Ahafo regions, the authors used a Translog SFA to model the efficiency of yam mini-sett technology adoption. The results showed that adopters in the Ashanti region were more efficient than those in Brong-Ahafo. Anang et al. [4] used Cobb-Douglas SFA to model rice production technical inefficiency in the Upper East and Northern regions of Ghana. Based on a sample of 300 rice farmers that cultivated in the 2013/2014 season, TE was estimated at 62%.

Extensions of the SFA accommodated the possibility that Decision Making Units (DMUs) do not operate with identical technologies. Differences in technology could be due to resource availability, government regulation, input prices, and the environment. From the literature, failure to account for technological differences in each DMU's circumstance could lead to falsely attributing technological gap-related production shortfalls to inefficient input use [21]. Consequently, since Hayami [37]introduced the Meta-Stochastic frontier (MSF) approach and further developments [38,39], it has been used to capture technological differences among DMUs.

The literature on MSF has grown by incorporating the possibility of technological heterogeneity in the analysis. However, except for a few studies [18,20,40], the Ghanaian literature is limited to districts/regions for one production season. For instance, Owusu et al. [6] assessed the gender technology gap among smallholder rice farmers by estimating a two-stage Cobb-Douglas MSF function to generate TE, Meta-Technical Efficiency (MTE), and Technology Gap Ratio (TGR) for male and female rice farmers. TE estimates from their first stage showed that male farmers were marginally more efficient because of higher seeding and fertilizer application rates; however, the MTE and TGR scores from the second stage showed that female-managed farms were closer to the MSF than that of their male counterparts after accounting for group heterogeneities. However, by incorporating data from multiple seasons, a recent study also using MSF showed that from

1987-2017, the overall crop production gender gap in Ghana has reduced [22]; thus, highlighting the merits of incorporating data from multiple seasons.

Owusu [10] investigated the impact of improved maize variety adoption on TE and productivity from three agroecological zones using a Translog SFA combined with a matching estimator to correct for selectivity bias. The results showed that adopters in the Semi-Deciduous Forest Zone were between 25% to 36% more efficient than non-adopters, while in the Guinea Savannah Zone adopters were between 15% to 26% more efficient than non-adopters. In the Transitional Zone, adoption was found to diminish farmers' TE by 6-8%, which led to productivity losses of 12-19%. The author attributed differences in TE and productivity effects to differences in levels of adoption of complementary inputs among farmers in the various agro-ecological zones. A similar study also reported TGRs and MTE differences for rice farms in the Transitional and Guinea Savannah Zones of Ghana [7].

One of the few studies [18] that examined production throughout Ghana over growing seasons between 1987-2017 and along ecological lines showed that TEs for okra, pepper, and tomato were 0.54, 0.74, and 0.58 respectively. Like this study, Tsiboe et al. [18] used harmonized data from seven cross-sectional population-based surveys fielded throughout Ghana between 1987-2017 and were able to show that TE has generally increased for okra and pepper but remained constant for tomatoes. Furthermore, Tsiboe et al. [18] showed that ecological gaps are very small for pepper, modest for tomato, and high for okra.

3. Research area and data

3.1 Study AreaThe rest of the paper is structured as follows. The next section presents an overview of the study area and data. In Section 3, we discuss how the Meta-Stochastic Frontier is used to

assess shortfalls in starchy staple production in Ghana. Section 4 presents the results while Section 5 discusses the findings. We present concluding remarks in Section 6.

2. Research area and data

2.1 Study area

Ghana covers a total land area of 24 million hectares of which 20.7% is considered arable, 40.7% under forests, 11.8% under permanent crops, and 26.8% for other uses. Ghana's ecology is classified as Rain Forest~~ (RFEZ),~~, Semi-Deciduous Forest~~ (SFEZ),~~, Coastal ~~Savanna (CSEZ), Transitional Zone (TEZ),~~Savannah, Transition, Guinea ~~Savanna (GSEZ),~~Savannah, and Sudan ~~Savanna (SSEZ).~~Savannah zones. The aridity of these ecologies increases from south to north. Farming systems across these ecologies are highly heterogeneous. However, the highest-yielding ecologies across all the starchy staples are ~~SFEZ~~Semi-Deciduous Forest and ~~TEZ~~Transition zones, and the ~~low~~lowest-yielding ones are ~~SSEZ~~Sudan Savannah and ~~RFEZ.~~Rain-Forest zones. Given their well-balanced annual rainfall and modest temperatures, ~~SFEZ~~Semi-Deciduous Forest and ~~TEZ~~Transition zones have the optimal conditions to grow starchy staples. Thus, it is not surprising that they also contain most of the cultivated lands allocated to starchy staples as shown in Tables S1 and S2 (all tables, figures, and notes with a leading S indicate supplemental materials in the online appendix).

~~3.~~2 ~~Household Level.~~.2 Data ~~Sources~~sources

The study uses household level data drawn from two sources: (1) the two waves of the Ghana Socioeconomic Panel Survey (GSPS) fielded in 2009/10 and 2014/15 ~~[41]~~[30]; and (2) all seven Ghana Living Standards Surveys (GLSS) fielded between 1987-2017 and publicly available at the Ghana Statistical Service (GSS) National Data Archive~~ (NADA).~~. The surveys were based

on nationally representative samples, focusing on the household as the key socio-economic unit, and provides insights into Ghana's living conditions. The GSPS was collected by the Economic Growth Center (EGC) at Yale University and the Institute of Statistical, Social, and Economic Research (ISSER), and the GLSS by GSS. In terms of the raw data collection, both surveys followed a two-stage stratified sampling design, where enumeration areas and households were selected in the first and second stages, respectively. The harmonization details of these datasets to construct farmer-level data are published in Tsiboe ~~[40].~~[31]. It is worth noting that while the GSPS constitutes a panel, households are resampled for each GLSS round, hence repeated cross-sectional data. Consequently, since a large proportion of the data is from the GLSS (84%), the harmonized data ~~by Tsiboe [40]~~ can be viewed as a cross-sectional sample of the population of Ghanaian starchy staple farmers at roughly five-year intervals.

The final sample used in this study was limited to starchy staple farmers drawn from the various surveys, with yield (kg/ha) above the 5th and below the 90th ~~percentile~~percentiles by survey, crop, and region. The final sample, therefore, consisted of 15,405 farmers from 15,133 households, out of which 11,585, 3,468, 1,834, and 6,226 farmers cultivate cassava, yam, cocoyam, and plantain, respectively. All of Ghana's ecologies are well represented in the final sample, and the dataset captures 14 growing seasons. Nonetheless, due to sparse data, the ~~RFEZ~~Rain Forest and ~~SFEZ are~~Semi-Deciduous Forest zones were combined into ~~a~~the Forest ~~Ecology (FEZ).~~ecological zone. Note S1 in the online appendix documents the definition of all the variables used in this study.

~~3~~2.3 Descriptive statistics

Table 1 provides the descriptive statistics of the household level characteristics, showing their temporal, ecological, and crop variation. Except for land and yield, average values were estimated across all ecologies and by crop. In assessing the differences in these variables across ecology and

Formatted: Superscript

Formatted: Superscript

starchy staples, we used linear regression for continuous variables and a probit model for dummies. A trend variable, and a fixed effect for ecology and crop, as well as their interactions, were included in the estimations. Plots of differences in the household-level variables across ecologies are further elaborated in Figure S1 (*a* and *b*).

From Table 1, an average household contains about five members in adult equivalence (AE) units, consistent with national estimations of 4.4 and 5.1 in the 2010 and 2000 censuses respectively ~~[42]. The dependency ratio, with an average rate of 1.30~~[32]. The dependency ratio, with an average rate of 1.29, measures the extent of pressure on an earning individual in the household. Averagely, the starchy staple farmer in Ghana is a 47-year-old male with five years of formal education and a 61% chance of owning his main farm plot. Regarding starchy staples, we found no significant variation between males and females (1=female), education, and land ownership; but in terms of ecological zones, we found significant variation in these variables. Age, on the other hand, showed no significant ecological or crop variation. In terms of their temporal dynamics from 1987 to 2017, while the probability of observing a female starchy staple farmer significantly declined by 0.~~17~~18% annually, that of age and years of formal education increased by 0.47% and 1.~~58~~59% annually, respectively.

Table 1. Summary statistics of starchy staple producing farmers in Ghana (1987-2017)

Variable	Mean (SD)	Trend (%)
Household [a]		
Size (AE)	4.83 † (2.887)	0.10 [0.055]
Dependency (ratio)	1.29 (1.561)	0.16 [0.113]
Farmer [b]		
Female (dummy)	0.29 † (0.454)	-0.18* † [0.031]
Age (years)	46.84 (14.872)	0.47* [0.029]
Education (years)	5.01 † (4.820)	1.59* † ‡ [0.103]
Land owned (dummy)	0.61 † (0.489)	-0.03 † [0.033]
Land (ha) [b]		
Cassava	1.17 † (2.753)	-54.18 † [59.567]
Yam	1.57 † (3.655)	-13.88* † [2.141]

Formatted: Font: Not Italic

Formatted Table

Cocoyam	1.55 (3.883)	10.54 [832.372]
Plantain	1.19 † (2.611)	-7.18 † [105.680]
Yield (Mt/ha) [b]		
Cassava	0.66 † (1.080)	2.78* † [0.220]
Yam	0.96 (1.540)	-38.47 † [3146.763]
Cocoyam	0.31 † (0.547)	0.15 [0.487]
Plantain	0.53 † (0.838)	1.57* † [0.296]
Input use [b]		
Planting material (Mt/ha)	0.06 † ‡ (0.428)	-5.68 † ‡ [20.428]
Family labor (AE)	2.74 † (1.544)	0.45* [0.054]
Hired labor (man-days/ha)	11.29 ‡ (43.200)	1.24* † ‡ [0.350]
Pesticide (Liter/ha)	7.83 † (49.505)	-36.04 † ‡ [2059.161]
Enabling environment [a]		
Credit (dummy)	0.20 † ‡ (0.399)	-0.06* ‡ [0.028]
Mechanization (dummy)	0.04 † ‡ (0.201)	-0.21* ‡ [0.015]
Extension (dummy)	0.19 (0.392)	0.75* † ‡ [0.029]

* Indicates significance at $p<0.05$; † and ‡ indicate statistically significant ($p<0.05$) differences in the variables across ecology and crop, respectively. The differences were determined via a linear regression for continuous variables and a probit model for dummies. A trend variable, and a fixed effect for ecology and crop, as well as their interactions, were included in the estimation.

[a] Household sample size; Cassava [11,459], Yam [3,439], Cocoyam [1,830], Plantain [6,166], Pooled [15,133]

[b] Farmer sample size; Cassava [11,585], Yam [3,468], Cocoyam [1,834], Plantain [6,226], Pooled [15,405]

Data Sources: Ghana Living Standards Surveys [wave 1-7] and Ghana Socioeconomic Panel Survey [wave 1-2]

Formatted: Space Before: 6 pt, Don't adjust space between Latin and Asian text, Don't adjust space between Asian text and numbers

In terms of production scale, the value of mean land area confirms the widely held notion that agriculture in Ghana is operated on a smallholder basis. Generally, yam (1.57 ha) has received the largest plot allocation for the period 1987-2017 while cassava (1.17 ha) received the lowest. Starchy staple farm sizes are statistically different across ecologies and have declined over the period (1987-2017). Given these scales of production, yields were estimated at 0.66, 0.96, 0.31, and 0.53 Mt/ha for cassava, yam, cocoyam, and plantain, respectively. Except for cocoyam, these yields also vary ~~along ecological lines.~~by ecology. Nonetheless, except for yam, ~~they~~ all crops show an annual upward trend in yields from 1987 to 2017. ~~Figure S1 (*a and b*) provides further details on how the household-level variables differ across ecologies and crops.~~

~~In terms~~Quantities and types of ~~input utilization, the~~inputs used in production significantly determine technical efficiency and technological levels. The starchy staple production functions analyzed in this study focused on the technical transformation of land, planting materials, family

and hired labor, and pesticides into outputs. The planting material usage rate was estimated at 0.06 Mt/ha, with the ~~SSEZ~~Sudan Savannah ecology having the highest and ~~CSEZ~~Coastal Savannah having the lowest. On average, three household members provided labor for starchy staple production, and this has significantly increased by about 0.45% annually since 1987. Hired labor is used at a rate of about 11 man-days/ha, which is equivalent to an annual increase of about 1.24% since 1987. The highest usage of hired labor is for farms in the ~~SSEZ~~Sudan Savannah zone, followed by ~~TEZ, CSEZ, GSEZ~~Transition, Coastal Savannah, Guinea Savannah, and ~~FEZ~~Forest zones, in that order. Farmers in the sample used about 8 liters of pesticides per hectare. Analyzing pesticide use across ecological zones and time shows consistently low usage in the ~~CSEZ~~Coastal Savannah relative to the ~~SSEZ~~Sudan Savannah and ~~FEZ~~Forest zones for all crops.

Access to credit, mechanization, and extension (measured as dummies) depicts factors that reflect the enabling environment for production success. Access to ~~credit and extension~~these production support services ~~are~~is reported in the literature to have diverse effects on technical inefficiency. The ~~rate~~rates of access to credit ~~or~~and extension ~~is~~are estimated at 20% and 19~~%; however, credit access~~% respectively while that for mechanization is ~~higher in FEZ and CSEZ than in the GSEZ and SSEZ.~~a paltry 4%. Furthermore, Table 1 shows that the rate of access to both credit and mechanization has significantly declined annually by 0.~~19~~06% and 0.21% for credit and mechanization respectively but increased for extension by 0.75% from 1987 to 2017.

~~4. Methodology~~

~~4. Theoretical framework~~

~~The prototypical output-oriented SFA approach assumes that given their input set, DMUs utilizing a homogeneous~~ We present the heterogenous effects of these variables on technical inefficiency across crops and ecological technology gaps in Section 4.

3. Econometric strategy

Several studies have shown that farmers in Ghana operate under spatially differentiated technologies [11,33] which have been linked to variability in Ghana's agroecology. We present Figures in the online appendix and statistical tests in Table 2 that suggest that production characteristics do vary across ecologies and starchy staples. Thus, it is important to account for these spatial heterogeneities driven by agroecological zones when investigating the production performance of starchy staple farmers operating at different levels relative to the best practice frontier. According to Battesse et al. ~~will put them~~[26], not accounting for these technological differences could lead to false attribution of production shortfalls. In the estimation of our empirical model, we group farmers into agroecological technology groups and apply the MSF following Huang et al. [28] two-step method as outlined below.

For each starchy staple in each ecology, we assumed a homogenous farm production technology, which, when coupled with best management practice, situates farmers at various points along ~~the~~their respective ecology Stochastic Frontier (SF). However, due to technical inefficiency and/or idiosyncratic shocks (i.e., downside production risk), one may observe some ~~DMUs~~farmers below the SF~~.~~ [34]. Furthermore, some ~~DMUs~~farmers may also be observed above the SF solely due to upside production risk. ~~The SFA is formulated as:~~

Previous studies have shown that Ghanaian crop production at the micro-level is best captured by the Cobb-Douglas [8,10], or Translog [11,22] production function. However, due to its relative flexibility, this study implements the MSF assuming that $f_t^j(\cdot)$ is Translog. Nonetheless, since Cobb-Douglas is nested within the Translog, the former was tested after estimation and was

soundly rejected at p<0.01 for all models (see Table 2). Thus, the Translog specification is used. The SF production function for the jth agro-ecology is specified as:

$$f^j(x_i) = \ln y_{ijt} = \beta_{0r} + \sum_k \beta_{kj} \ln x_{kijt} + \frac{1}{2}\sum_s \sum_k \beta_{skj} log \ln x_{kijt} \ln x_{sijt} + v_{ijt} - u_{ijt}$$

$$u_{ijt} \sim N^+[0, \exp(\boldsymbol{w}_{ijt}\boldsymbol{\alpha})], \quad v_{ijt} \sim N(0, \sigma_v^2) \qquad (1)$$

where, y_{ijt} is total production (Mt) for the ith farmer in ecology j at time t. Each x_{kijt} represents the kth input (i.e., land, planting material, family and hired labor, and pesticides) used by the ith farmer and a trend variable. As Van Nguyen et al. [35] report, outcomes from the SF, especially with efficiency estimates, are severely affected by the distribution assumptions. Therefore, we considered all possible distributions but eventually had to settle on a half-normal distribution (i.e., $u_{ijt} \sim N^+[0, \exp(\boldsymbol{w}_{ijt}\boldsymbol{\alpha}_j)]$), since the truncated normal and exponential distributions presented issues of convergence. Following Tsiboe et al. $y_i = f(x_i)e^{\varepsilon_i}$, $\varepsilon_i = v_i - u_i$ (1)

where, y_i is the output level when x_i inputs are used with the technology $f(x_i)$. The deviations from the SF due to technical inefficiency and production risk are represented by u_i and v_i, respectively. Consequently, the assumptions made on the distribution of the deviations (v_i and u_i) underpin the estimation of Equation (1). Generally, v_i is assumed to follow a normal distribution with zero mean and variance $\sigma_{v_i}^2$ [$v_i \sim N(0, \sigma_v^2)$]. However, due to its negative skewness, u_i is assumed to follow either half-normal, exponential, truncated, or gamma distributions [26]. It is important to mention that the distribution of u_i affects the estimates of TE [43].

In the context of SFA, the technical inefficiency component (u_i) has been shown to be influenced by exogenous variables other than inputs, and its variance has been redefined as $\sigma_{u_i}^2 = \exp(\boldsymbol{w}_i\boldsymbol{\alpha})$;

where $\boldsymbol{w}_i$ and $\boldsymbol{\alpha}$ are the vectors of explanatory variables and parameters [44], respectively. Under these modifications, rejecting $H_o\colon \boldsymbol{\alpha} = 0$ provides justification that exogenous factors explain inefficiency [28]. The TE of the ith DMU is calculated as:

[23], $\boldsymbol{w}_{ijt}$ contained farmer characteristics (age, education, and gender), institutional factors (land ownership, credit, and extension), a trend and a constant term. Given the SF production function for the jth agro-ecology, the "pure farmer technical efficiency" (TE) of the ith farmer is calculated as:

$$TE_i = E[\exp(-u_i)\,|\hat{\varepsilon}_i] \qquad (2)$$

Assuming that DMUs can be grouped into technological groups, Equation (1) can be estimated as a Meta-Stochastic frontier (MSF) following Huang et al. [23] two-step method. Equation (1) is initially estimated separately for each sub-group (*j*), and then the predicted output levels from the sub-group frontiers are used as the observation for MSF (pooled stochastic frontiers) in the second step. The conventional one-sided error term (u_i^M) for the MSF serves as the estimate for any technology gaps among the different sub-groups. The MSF which envelops all sub-group-specific stochastic frontiers [$f^j(x_i)$] is specified in Equation (3) as

$$f^j(x_i) = f^M(x_i)e^{-u_i^M}, \qquad u_i^M \sim N^+(0, \exp(\boldsymbol{w}_i\boldsymbol{\alpha})) \qquad (3)$$

where u_i^MTo implement Huang et al's. [28] two-step MSF method, Equation (1) is initially estimated separately for each agro-ecology (*j*), and then the predicted output levels from the agro-ecology frontiers are used as the observation for MSF (pooled stochastic frontiers) in the second step. The conventional one-sided error term (u_{iM}) for the MSF serves as the estimate for any technology gaps among the different agro-ecology. The MSF [$f^M(x_i)$] which envelops all agro-ecology-specific stochastic frontiers [$f^j(x_i)$] is specified in Equation (3) as

$$f^{M}(x_i) = \ln \hat{y}_{ijt} = \beta_{0r} + \sum_k \beta_{kM} \ln x_{kiMt} + \frac{1}{2}\sum_s \sum_k \beta_{skM} log \ln x_{kiMt} \ln x_{siMt} - u_{iMt} \quad (3)$$

Where $u_{iM} \sim N^{+}\left(0, \exp(\boldsymbol{w}_i \boldsymbol{\alpha}_M)\right)$ is strictly positive, implying that $f^{j}(x_i) \leq f^{M}(x_i)$. Consequently, the ratio of ~~the group~~agro-ecology j's stochastic frontier to the MSF is the technology gap ratio (TGR~~,~~), represented as

$$TGR_i = \frac{f^{j}(x_i)}{f^{M}(x_i)} = e^{-u_i^M} \frac{f^{j}(x_i)}{f^{M}(x_i)} = e^{-u_{iM}} \leq 1$$

(4)

The TGR depends on the accessibility and adoption level of the available MSF which in turn depends on ~~DMU~~farm-specific circumstances. The meta-technical efficiency (MTE), a measure of overall performance, represents each ~~DMU's~~farmer's technical efficiency with respect to the meta frontier production technology. MTE can be decomposed into the TE (which is the technical efficiency measured with respect to the zone-specific frontier) and the TGR (which is the difference between the best available technology and the technology set adopted). Accordingly, each ~~DMU's~~farmer's MTE is given by equation (5) as follows:

$$MTE_i = f^{j}(x_i)[f^{M}(x_i)e^{v_i}]^{-1} = TGR_i \times TE_i \quad (5)$$

~~**4.1 Empirical model specification**~~

~~Previous studies have shown that Ghanaian crop production at the micro level is best captured by the Cobb-Douglas [4,6], or Translog [7,17] production function.~~The MSF is estimated separately for each crop, so we use the physical measure of total output and input (log-transformed) used by the farmer. Thus, all outputs and inputs are not standardized by farm size. ~~However, due to its relative flexibility, this study implements the MSF assuming that $f_i^j(\cdot)$ is Translog. Nonetheless, since Cobb-Douglas is nested within the Translog, the former was tested after estimation and was~~

soundly rejected at p<0.01 for all models (see Table 2). Thus, the Translog specification is appropriate for the data, and is represented as

$$\ln y_{ijt} = \beta_{0r} + \sum_k \beta_{kj} \ln x_{kijt} + \frac{1}{2}\sum_s \sum_k \beta_{skj} log \ln x_{kijt} \ln x_{sijt} + v_{ijt} - u_{ijt}$$

$$u_{ijt} \sim N^{+}[0, \exp(\boldsymbol{w}_{ijt}\boldsymbol{\alpha})], \quad v_{ijt} \sim N(0, \sigma_v^2) \qquad (6)$$

where, y_{jit} is total production (Mt) for the ith farmer in ecology j at time t. Each x_{kjit} represents the kth input (i.e., land, planting material, family and hired labor, and pesticides) used by the ith farmer and a trend variable. As Van Nguyen et al. [43] report, outcomes from the SFA, especially with efficiency estimates, are severely affected by the distribution assumptions. Therefore, we considered all possible distributions but eventually had to settle on a half-normal distribution (i.e., $u_{ijt} \sim N^{+}[0, \exp(\boldsymbol{w}_{ijt}\boldsymbol{\alpha})]$), since the truncated normal and exponential distributions presented issues of convergence. Following Tsiboe et al. [18], $\boldsymbol{w}_{ijt}$ contained farmer characteristics (age, education, and gender), institutional factors (land ownership, credit, and extension), a trend and constant term.

The parameters of the ecology- and meta-frontiers were estimated via maximum likelihood, using the "*frontier*" command in Stata 15. Furthermore, estimation variables were standardized by their sample means by survey and crop. Given the parameters, input elasticities were estimated as the first derivative of the frontiers with respect to that input, evaluated at their means. The production returns to scale (RTS) were then estimated as the sum of the input elasticities. The standard errors for the elasticities were estimated via the delta method. Ecology-specific technical TE, TGR, and MTE were estimated using equations (2), (4), and (5), respectively. Estimates were summarized by ecology and season.

~~**Several studies have shown that farmers in Ghana operate under spatially differentiated technologies [7,45] which have been linked to variability in Ghana's agroecology. We present Figures in the appendix and statistical tests in Table 1**~~**4. Econometric results**

~~that suggest that production characteristics do vary across ecologies and starchy staples. Thus, it is important to account for these spatial heterogeneities driven by agroecological zones when investigating the production performance of starchy staple farmers operating at different levels relative to the best practice frontier. According to Battesse et al. [21], not accounting for these technological differences could lead to false attribution of production shortfalls. In the estimation of our empirical model, we group farmers into agroecological technology groups and apply the Meta-Stochastic frontier (MSF) following Huang et al. [23] two-step method as outlined in the theoretical framework section above. The MSF is estimated separately for each crop, so we use the physical measure of total output and input (log-transformed) used by the farmer. Thus, all outputs and inputs are not standardized by farm size.~~

~~**5. Results and Discussions**~~

The core objective of this paper is to understand whether production shortfalls existing among the starchy staples in Ghana are driven by technological gaps, technical inefficiency, or both. ~~Thus, the discussion in this~~This results section ~~seeks to accomplish that purpose. In the interest of space~~has four parts. First, we ~~only~~ present ~~the~~results on diagnostic ~~test~~tests of the empirical model~~,~~ specification before proceeding to the estimates of inputs elasticities, returns to scale (RTS), and ~~estimates of TE, TGR~~technical change for each crop, ecology, and the meta-frontier in the second part. This is followed by the spatiotemporal trends of TE, TGR, MTE~~-~~, and technology gaps in the ~~main text in Tables 2, 3, and 4, respectively.~~third part. The ~~paper~~final part presents ~~the estimates~~results on drivers of technical inefficiency and technology gaps. Estimates of the

production functions for each crop (Tables S3-S6) and drivers of technical inefficiency (Table S7) in the online appendix. Discussions on the drivers of technical inefficiency), ecological variations in production yield, input, and technology gaps enabling environment factors, and in starchy staple farmer and household demographic factors in Ghana (Figures S1a and S1b) are also presented in Note S3 in the online appendix. Temporal and spatial trends of TE, TGR, and MTE are also presented in Figures 2-3, and Table S8.

The diagnostics on the negative skewed error needed for the justification of SFA shown in Table 2 confirmed the appropriateness**4.1 Diagnostic tests of the empirical model. specification**

Formatted: Font: Bold

The tests results for functional form specification, skewed error specification, production variance due to technical inefficiency, and hypothesis tests that variables in the inefficiency functions of ecology-specific and meta-frontier models do not affect starchy staple inefficiency are presented in Table 2.

The Cobb-Douglas and Translog functional forms are the most popular choices when specifying MSF production functions. This study tested the null hypothesis that cross terms in the translog functional form are jointly equal to zero. The test result for all crops, ecologies, and the meta-frontier soundly rejected this hypothesis at 1% level, showing that the translog function better fits the data. In the literature, the translog function is generally preferred because of its flexibility. For example, both Asravor et al. [8] and Tsiboe [19] used the translog specification for the MSF for rice and cocoa production in Ghana.

The results from the Coelli [36] skewness test for negative OLS residuals and the Gutierrez et al. [37] likelihood ratio tests for technical inefficiency both reject the null hypothesis of no one-sided error and show that the MSF modeled for starchy staple technical inefficiency functions is justified.

Further, test results on the similaritynull hypothesis that the inefficiency variance is not influenced

by variables in the inefficiency function (H_o: $\boldsymbol{\alpha} = 0$) was also rejected for all crops, ecologies, and the meta-frontier.

The proportion of production variance due to inefficiency (γ) ranged from 0.50 to 0.99 for the ecology frontiers; for a given crop, this was always higher than that of the meta-frontier.

Table 2. Hypothesis tests for ecology- and meta-frontier models for starchy staple production in Ghana (1987-2017)

	Sample size	Log-likelihood	Cobb-Douglas test	Coelli, (1995) [ab] skewness test	Gutierrez (2001) [a] LR test	Inefficiency variance $[\sigma_u]$	Total variance $[\sigma^2 = \sigma_u^2 + \sigma_v^2]$	Gamma $[\gamma = \sigma_u^2/\sigma^2]$	Inefficiency function test	Model significance
Cassava										
Guinea Savanah	801	-919	227.02***	-6.02*	11.28***	0.87 (0.092)	1.10 (0.119)	0.68*** (0.076)	37.20***	710.21***
Transitional Zone	2,064	-2,735	307.98***	-3.52*	13.96***	0.94 (0.088)	1.46 (0.118)	0.61*** (0.066)	88.27***	1194.47***
Forest Zone	7,023	-9,129	553.51***	-5.12*	19.05***	0.77 (0.061)	1.20 (0.065)	0.50*** (0.053)	109.64***	3941.36***
Coastal Savanah	1,697	-2,154	211.58***	-2.84*	6.13**	0.80 (0.114)	1.21 (0.126)	0.53*** (0.097)	403.63***	1390.95***
National	11,585	-15,347	1013.19***	-9.07*	66.22***	0.89 (0.038)	1.37 (0.048)	0.58*** (0.030)	196.70***	6319.90***
Meta-frontier	11,585	-166	16605.98***	4555.61	-	0.00 (0.036)	0.05 (0.001)	0.00 (0.001)	563.00***	91075.02***
Yam										
Sudan Savanah	222	-233	164.54***	-0.23	0.52	0.87 (0.402)	1.12 (0.480)	0.68** (0.339)	534.06***	500.60***
Guinea Savanah	1,472	-1,732	400.32***	-9.80*	34.53***	1.04 (0.064)	1.34 (0.103)	0.81*** (0.041)	25.54*	2378.83***
Transitional Zone	943	-1,288	199.15***	-4.89*	32.23***	1.35 (0.091)	2.16 (0.195)	0.84*** (0.042)	1666.17***	1328.45***
Forest Zone	831	-1,212	170.34***	-3.31*	23.13***	1.41 (0.115)	2.51 (0.251)	0.79*** (0.054)	48.43***	772.00***
National	3,468	-4,936	515.54***	-12.13*	226.81***	1.53 (0.040)	2.67 (0.104)	0.88*** (0.014)	213.99***	4669.74***
Meta-frontier	3,468	-1,994	3948.97***	-197.43*	742.33***	0.72 (0.011)	0.53 (0.015)	0.97*** (0.003)	14048.06***	38680.49***
Cocoyam										
Transitional Zone	422	-597	99.75***	-1.84	9.11***	1.38 (0.171)	2.40 (0.359)	0.79*** (0.084)	23.91**	322.93***
Forest Zone	1,412	-2,046	75.45***	-3.13*	25.43***	1.36 (0.104)	2.37 (0.212)	0.78*** (0.053)	42.48***	495.20***
National	1,834	-2,721	113.04***	-2.54*	20.51***	1.27 (0.106)	2.29 (0.195)	0.71*** (0.060)	54.73***	611.93***
Meta-frontier	1,834	-377	1587.92***	658.26	-	0.00 (0.082)	0.08 (0.003)	0.00 (0.004)	890.54***	8960.81***
Plantain										
Transitional Zone	740	-985	125.50***	-5.65*	39.00***	1.38 (0.085)	2.21 (0.195)	0.86*** (0.035)	63.15***	760.94***
Forest Zone	4,982	-6,564	357.18***	-6.48*	36.32***	0.97 (0.057)	1.46 (0.079)	0.64*** (0.042)	46.45***	3483.09***
Coastal Savanah	504	-576	109.18***	0.28	-	0.03 (0.690)	0.61 (0.046)	0.00 (0.064)	1138.60***	390.07***
National	6,226	-8,275	447.69***	-9.27*	78.14***	1.07 (0.044)	1.62 (0.068)	0.70*** (0.029)	88.04***	4366.28***
Meta-frontier	6,226	1,264	9511.74***	-1939.14*	1062.47***	0.40 (0.006)	0.19 (0.005)	0.83*** (0.008)	544.13***	113229.19***

Significance levels: * $p<0.10$, ** $p<0.05$, *** $p<0.01$

[a] Null hypothesis of no one-sided error (i.e., no inefficiency) was tested

[b] Values less than the critical value of -1.96 confirm the rejection of the null hypothesis.

Data Sources: Ghana Living Standards Surveys [wave 1-7] and Ghana Socioeconomic Panel Survey [wave 1-2]

The γ values suggest that a considerable amount of the observed variation in output for the ecology-frontiers could be attributed to inefficient use of inputs. The range of γ for the meta-frontier also suggests that the observed variation in output, given the ecology frontiers, could not be attributed to idiosyncrasies.

To statistically check the similarities of the production frontiers for a given starchy staple across the different ecologies, the study applied a generalized likelihood ratio test. The log-likelihood values under the null hypothesis of a uniform technology were all smaller than that of the alternate hypothesis of heterogeneous technology. across ecologies support the factThe test statistics led to sound rejection of the null hypothesis, implying that starchy staple farmers are operating under heterogeneous technologies in the various ecologies. This justifiesalong ecological lines.

Finally, the SF model chi-squared test statistics indicate that the models are all significant. These justify further inquiry into whether technology gaps across ecologies or resource use inefficiencies among farmers contribute more to production shortfalls. The major learning points include the wide range of the estimated γ values which suggest that considerable amounts of the observed crop output variation for the ecology-frontiers [meta-frontier] could be attributed to inefficient use of inputs [technological gaps].We report the summary of estimates for the ecology- and meta-frontier The range of γ for the meta-frontier also suggests that the observed variation in output, given the ecology frontiers, could not be attributed to idiosyncrasies. Also, the SFA model chi-squared test statistics indicate that the models are all significant. A specific discussion of relevant diagnostics is presented in Note S2.

5.1 Elasticities and Meta-Frontier Results of Ghanaian Starchy Staples Production

~~Based on~~ production elasticities ~~for cassava in Table 3,~~in Table 3 and TGR, TE, and MTE for starchy staples production in Table 4. Detailed results from these are discussed in the following subsections along crop-specific lines.

4.2 Output elasticities with respect to inputs, returns to scale and technical change.

Given that earlier results confirmed heterogeneous production technologies along ecological lines, we estimated separate ecology-frontier and meta-frontier models for each crop and estimated local and national returns to scale (RTS) parameters from these models to test the null hypothesis of constant RTS from each model. The estimated elasticities for ecology-specific and meta-frontier models for all starchy staples are presented in this order in Table 3.

4.2.1 Cassava production

Cassava is produced in all ecological zones under study except Sudan Savannah. The elasticities for land ~~is the most operationally~~are consistently statistically significant ~~input contributing the~~ and contribute the most to explaining changes in cassava production efficiency across all ecologies and the meta-frontier. Land elasticity is highest ~~to output across all ecological zones. The contributions of family~~in the Coastal Savannah zone (0.41) and lowest in the Guinea Savannah (0.31) while the national average land elasticity of 0.39 is like the meta-frontier estimate. Family labor~~,~~ contributes the next highest elasticities followed by hired labor~~,~~. Again, the estimate for family labor is highest in the Coastal Savana (0.17) but the lowest for Transition zone (0.10). The national and meta-frontier elasticities average about 0.16 each. The estimated elasticities for hired labor are comparable across ecologies and the meta-frontier with the Guinea Savannah zone (0.05) slightly outperforming the rest (0.04). Where statistically significant, the elasticities for planting materials and pesticides reduce both the production frontiers and ecological technologies for cassava production. The negative effect for planting material~~, and pesticide use follow in that order across~~

~~all ecological zones. The returns to scale (RTS) estimates for all ecologies and the meta-frontier confirm decreasing returns to scale (DRS).~~ is highest in the Guinea Savannah zone (-0.13) and is, at least, three times the estimated national (-0.03) and meta-frontier (-0.04) effects. The negative effect of pesticide use on cassava output is even more distressing since the estimated elasticity for Coastal Savannah (-0.09) outstrips the national and meta-frontier elasticities by about nine-folds.

Taken together, cassava production exhibits decreasing RTS for all ecologies and at the national and meta-frontier levels. The ecology RTS for cassava production ranges from 0.39 in the Guinea Savannah to 0.53 in both the Transitional and Forest zones. The national (0.55) and meta-frontier (0.54) RTS for cassava align with the agro-ecology findings.

Table 3. Elasticities for ecology- and meta-frontier models for starchy staple production in Ghana (1987-2017)

	Land elasticity	Planting material elasticity	Family labor elasticity	Hired Labor elasticity	Pesticide elasticity	Returns to scale [a]
Cassava						
Guinea Savanah	0.31*** (0.030)	-0.13*** (0.025)	0.16*** (0.063)	0.05*** (0.008)	0.00 (0.014)	0.39*** (0.069)
Transitional Zone	0.36*** (0.019)	0.02 (0.018)	0.10** (0.042)	0.04*** (0.009)	0.01 (0.007)	0.53*** (0.047)
Forest Zone	0.40*** (0.010)	-0.04*** (0.010)	0.14*** (0.023)	0.04*** (0.003)	-0.01* (0.004)	0.53*** (0.026)
Coastal Savanah	0.41*** (0.020)	-0.05*** (0.019)	0.17*** (0.046)	0.04*** (0.007)	-0.09*** (0.018)	0.47*** (0.059)
National	0.39*** (0.008)	-0.03*** (0.008)	0.16*** (0.018)	0.04*** (0.003)	-0.01** (0.004)	0.55*** (0.020)
Meta-frontier	0.39*** (0.002)	-0.04*** (0.002)	0.16*** (0.005)	0.04*** (0.001)	-0.01*** (0.001)	0.54*** (0.006)
Yam						
Sudan Savanah	0.44*** (0.068)	-0.08** (0.032)	0.18* (0.103)	0.01 (0.015)	-0.03 (0.020)	0.51*** (0.133)
Guinea Savanah	0.59*** (0.025)	-0.08*** (0.019)	0.02 (0.047)	0.02*** (0.005)	-0.02 (0.013)	0.52*** (0.056)
Transitional Zone	0.55*** (0.035)	0.00 (0.020)	-0.05 (0.061)	0.10*** (0.016)	0.02 (0.011)	0.61*** (0.072)
Forest Zone	0.32*** (0.030)	-0.08* (0.043)	0.20*** (0.076)	0.09*** (0.014)	-0.01 (0.014)	0.53*** (0.091)
National	0.55*** (0.019)	-0.06*** (0.013)	0.12*** (0.035)	0.05*** (0.005)	-0.02*** (0.007)	0.64*** (0.040)
Meta-frontier	0.51*** (0.009)	-0.07*** (0.005)	0.10*** (0.019)	0.06*** (0.002)	-0.01*** (0.003)	0.58*** (0.021)
Cocoyam						
Transitional Zone	0.19*** (0.040)	-0.18** (0.083)	0.21** (0.104)	0.08*** (0.022)	-0.03 (0.034)	0.27*** (0.134)
Forest Zone	0.42*** (0.041)	-0.10*** (0.037)	0.08 (0.059)	0.04*** (0.010)	0.02 (0.016)	0.45*** (0.076)
National	0.36*** (0.027)	-0.10*** (0.035)	0.09* (0.052)	0.05*** (0.009)	0.01 (0.014)	0.42*** (0.066)
Meta-frontier	0.38*** (0.005)	-0.10*** (0.015)	0.11*** (0.015)	0.05*** (0.003)	0.01*** (0.004)	0.46*** (0.023)
Plantain						
Transitional Zone	0.44*** (0.031)	-0.04 (0.032)	0.10 (0.066)	0.06*** (0.011)	0.02 (0.015)	0.59*** (0.073)
Forest Zone	0.47*** (0.014)	-0.03** (0.013)	0.14*** (0.027)	0.04*** (0.004)	0.00 (0.005)	0.62*** (0.031)
Coastal Savanah	0.37*** (0.032)	0.06* (0.033)	0.12* (0.073)	0.07*** (0.015)	-0.05** (0.023)	0.58*** (0.098)
National	0.47*** (0.012)	-0.02* (0.011)	0.14*** (0.025)	0.04*** (0.004)	0.00 (0.005)	0.63*** (0.028)
Meta-frontier	0.47*** (0.002)	-0.03*** (0.002)	0.14*** (0.006)	0.04*** (0.001)	0.01*** (0.001)	0.63*** (0.007)

Significance levels: * p<0.10, ** p<0.05, ***p<0.01
[a] Null hypothesis of constant returns to scale was tested.
Data Sources: Ghana Living Standards Surveys [wave 1-7] and Ghana Socioeconomic Panel Survey [wave 1-2]

Like cassava, land contributes the highest returns to yam production in all ecological zones. Yam is a very dominant cash crop enterprise. Due to the laborious nature of the enterprise, farmers often employ hired labor as much as they rely on family labor. The labor activities in yam production are often specialized (e.g., making mounds) and this limits issues of moral hazard from hired labor. Hence, it is not too surprising that family labor (for SSEZ and

FEZ), as well as hired labor (GSEZ and TEZ), have the second-highest significant elasticities. Besides land and labor, the contributions of pesticides and planting materials to yam output are either not significant or negative. Estimated returns to scale follow the pattern observed for cassava; increasing all yam production inputs proportionately would yield a less-than-proportionate increase in yam output.

For cocoyam, land makes the highest contribution to output in the FEZ but is marginally outstripped by family labor in the TEZ. Along with land, hired labor makes consistently positive contributions to cocoyam output in both ecological zones; planting materials significantly reduce output while pesticides make no significant contributions. Cocoyam returns to scale follows that of cassava and yam. Again, land has the highest output elasticity across all ecological zones for plantain and is followed by family labor and hired labor. Planting materials show significant effects in FEZ (negative) and CSEZ (positive) while pesticide is only significant in CSEZ (negative). Like the other starchy staples, plantain production exhibits DRS in all ecological zones and nationally.

Next, we analyze the trends and distributions of technological gaps and

4.2.2 Yam production

From Figure 2 and Table 3, yam is produced in all agro-ecologies except Coastal Savannah. Like cassava, land contributes the most to explaining variations in yam output followed by family labor and hired labor. Pesticides and planting materials maintain their negative effects on output and ecology technology. Guinea Savannah (0.59) shows the most while Coastal Savannah (0.32) shows the least responsiveness to marginal changes in area cultivated. The elasticity of family labor in the Forest zone (0.20) is about 60% higher than the national elasticity (0.12) and twice that of the meta-frontier. Although family labor showed no effect in the Transition zone, hired labor elasticity (0.10) is twice the national average. Where statistically significant, planting material had negative effects on yam output at the ecological and meta-frontier levels. The elasticities of yam output with respect to changes in planting material clustered around -0.08 in the forest ($p<0.10$), Sudan Savannah ($p<0.05$), and Guinea Savannah ($p<0.01$) zones: seed yam showed no effect on output in the Transition zone. Like planting material, pesticide had generally negative effects on yam output (except in the Transition zone) but none of the ecology-specific elasticities were statistically significant. These results reflect the heterogenous production technologies for the same crop in different ecologies. At the meta-frontier level, both planting material (-0.07) and pesticide (-0.01) showed negative effects on yam output. Like cassava production, the RTS for yam also reflects decreasing returns to scale in all ecologies and the meta-frontier although the national level estimate (0.64) is the highest among all crops.

4.2.3 Cocoyam production

Cocoyam production in Ghana happens largely in the Transition and Forest ecological zones. These areas have more suitable biological conditions for this crop, but the estimated elasticities show marked heterogeneities in input use and production technologies. For example, land elasticity in the Forest zone (0.42) is more than twice the effect in the Transition zone (0.19); the

elasticity of family labor in the Transition zone (0.21) is the highest of all inputs in that zone but family labor shows no effect on cocoyam output in the Forest zone. Hired labor shows complementary relationships with family labor in both ecologies but its effect in the Transition zone is twice (0.08) that of the Forest zone. Pesticide application does not significantly influence cocoyam output while planting material maintains its negative output effects in both ecologies (Transition =-0.18; Forest = -0.10) and in the meta-frontier (-0.10). These negative effects are reflected in the low RTS for the Transition zone (0.27). Cumulatively, the meta-frontier RTS for cocoyam production (0.46) is the least among the starchy staples and implies that a 100% increase in all inputs will only yield a 46% increase in cocoyam output.

4.2.4 Plantain production

Plantain is mainly cultivated in the Transition, Forest, and Coastal Savannah zones of Ghana. Its ecology-specific and meta-frontier elasticities show that land contributes most to output, and the effect is more pronounced in the Forest zone (0.47) but lowest in the Coastal Savannah zone (0.37). The estimated land elasticity of 0.47 in the Forest zone is consistent with its analogues at the national and meta-frontier levels. Similar observations can be made for the elasticities of family labor where the estimate for Forest zone (0.14) is the same for the national and meta-frontier level. Interestingly, the elasticities of family labor (0.10) and planting material (-0.04) in the Transition zone have no significant effect on plantain output as was the case for yam production in that zone. Hired labor showed consistently significant and positive effects on plantain output with the elasticities ranging from 0.04 in the Forest zone to 0.07 in the Coastal Savannah while planting material and pesticide elasticities continued their largely negative effects. Interestingly, the only statistically significant effect of pesticide elasticity (-0.05) was found in the Coastal Savannah zone and, that zone also recorded the only positive effect of planting materials on plantain output. Like

the others, plantain production also showed decreasing RTS at the national and meta-frontier levels although the estimates for the latter (0.63, p<0.01) was the highest of all crops under study.

4.3 Technical efficiency, technology gap, and meta-technical ~~efficiencies based on crops in the various ecologies and highlight the implications of~~ efficiency of starchy staples

The summary of TE, TGR, and MTE estimates for agro-ecological zones are presented in Table 4, while Figures 1 and 2 present the corresponding plots. As indicated, these ~~findings. The discussions here~~efficiency parameters are ~~principally based on results in Table 4 and Figure 1. Corresponding values to Figure 1~~estimated from equations 2, 4, and 5. Detailed results from these estimations are ~~reported in Table S8. These results show the temporal and spatial dynamics of~~presented for each starchy staple~~-specific~~.

Table 4. Ghanaian starchy staple production technology level and technical efficiency~~.~~ across ecologies (1987-2017)

	Technology gap ratio (TGR)	Technical efficiency (TE)	Meta-technical efficiency (MTE)	Technology gap
Cassava				
Guinea Savanah	0.92*** (0.002)	0.64*** (0.007)	0.60*** (0.006)	8.50%
Transitional Zone	0.90*** (0.001)	0.58*** (0.004)	0.54*** (0.003)	9.69%
Forest Zone	0.87*** (0.000)	0.64*** (0.001)	0.56*** (0.001)	12.82%
Coastal Savanah	0.84*** (0.003)	0.72*** (0.005)	0.59*** (0.003)	16.31%
Mean	0.89	0.65	0.58	11.44%
Yam				
Sudan Savanah	0.81*** (0.009)	0.67*** (0.018)	0.55*** (0.016)	18.72%
Guinea Savanah	0.81*** (0.003)	0.54*** (0.005)	0.43*** (0.004)	18.70%
Transitional Zone	0.70*** (0.004)	0.49*** (0.007)	0.34*** (0.005)	30.00%
Forest Zone	0.55*** (0.005)	0.51*** (0.008)	0.24*** (0.005)	45.00%
Mean	0.65	0.54	0.33	35.11%
Cocoyam				
Transitional Zone	0.77*** (0.009)	0.56*** (0.011)	0.38*** (0.009)	22.66%
Forest Zone	0.82*** (0.002)	0.48*** (0.005)	0.39*** (0.004)	17.76%
Mean	0.80	0.47	0.36	20.21%
Plantain				
Transitional Zone	0.94*** (0.002)	0.50*** (0.008)	0.47*** (0.007)	6.39%
Forest Zone	0.87*** (0.000)	0.58*** (0.002)	0.51*** (0.002)	13.01%
Coastal Savanah	0.75*** (0.005)	0.68*** (0.009)	0.50*** (0.008)	25.07%
Mean	0.85	0.58	0.50	15.13%
Overall mean	0.791	0.553	0.428	20.94%

Significance levels: * p<0.10, ** p<0.05, ***p<0.01
[a] Null hypothesis of constant returns to scale was tested.
Data Sources: Ghana Living Standards Surveys [wave 1-7] and Ghana Socioeconomic Panel Survey [wave 1-2]

Formatted: Font: Bold

Formatted: Heading 4

Formatted Table

Formatted Table

Formatted Table

Formatted Table

Formatted: Font: 12 pt

Formatted: Space After: 8 pt, Line spacing: Double

~~5.2~~4.3.1 Cassava ~~Production~~

~~In the data, four main ecological zones were significantly involved in cassava~~ **production**~~, which are the GSEZ, TEZ, FEZ, and CSEZ. The TGR ranges from zero to one: values closer to one indicate the use of technology like the meta-frontier technology. In other words, the technology gap~~

The technology gap (ranging from 0 to 1) is the difference between the best technology available in the production of a particular crop and the technology set adopted by a farmer. TGR values closer to one mean there is little room for improvement given the existing production technology in the country. The choice of the adopted technology is determined by environmental and socio-economic factors. The mean TGR for cassava, estimated at 0.~~88~~89, indicates that the technological gap averaged over all ecological zones is ~~12~~11%. Figure 1, panel a1 shows that the TGR for cassava increased from 0.72 in 1987 to 0.98 in 1998 and dropped to an average of 0.85 between 2004 and 2012. Afterward, it increased until settling at 0.88 in 2017. Given the high mean TGR across the sample, it is not surprising that the ecology~~-~~ specific TGRs are all above 80% with the highest in ~~GSEZ~~Guinea Savannah (0.92), reflecting a superior technology for that ecology. ~~The TGR for GSEZ~~This is followed by ~~that of TEZ~~the Transition zone (0.90), ~~FEZ~~the Forest zone (0.87), and then ~~CSEZ~~Coastal Savannah (0.84). The TGR for cassava is relatively high when compared to 0.75 for tomato ~~[18]~~[23] and 0.74 for rice [~~7~~11].

~~Ecology~~In terms of TE, the mean ecology-specific ~~TE~~estimate for cassava is ~~estimated at a mean of~~ 0.~~72~~65 across the entire sample. ~~Like the TGR~~Trend-wise, the TE for cassava increased by about 90% from 0.~~44~~46 in 1987 to a peak of 0.85 in 1991 ~~and~~but has been declining since then by about 5% annually to 0.46 again in 2017 (Figure 1, panel b1). Figure 2 shows that the mean TE for cassava is highest in ~~CSEZ~~Coastal Savannah (0.72) and followed by ~~FEZ/GSEZ~~Forest/Guinea Savannah zones (0.64) and then ~~TEZ~~Transition zone (0.58). An important caveat about these TE

Formatted: Font: Bold

Field Code Changed

scores is that they do not rank the farms based on the cassava meta-frontier, so we discuss the MTE results next.

After accounting for ecology-specific production technologies, the mean MTE for cassava across the entire sample is 0.58. Specifically, the most technically efficient cassava farmers relative to the meta-frontier are those in the Guinea Savannah with an MTE of 0.60.

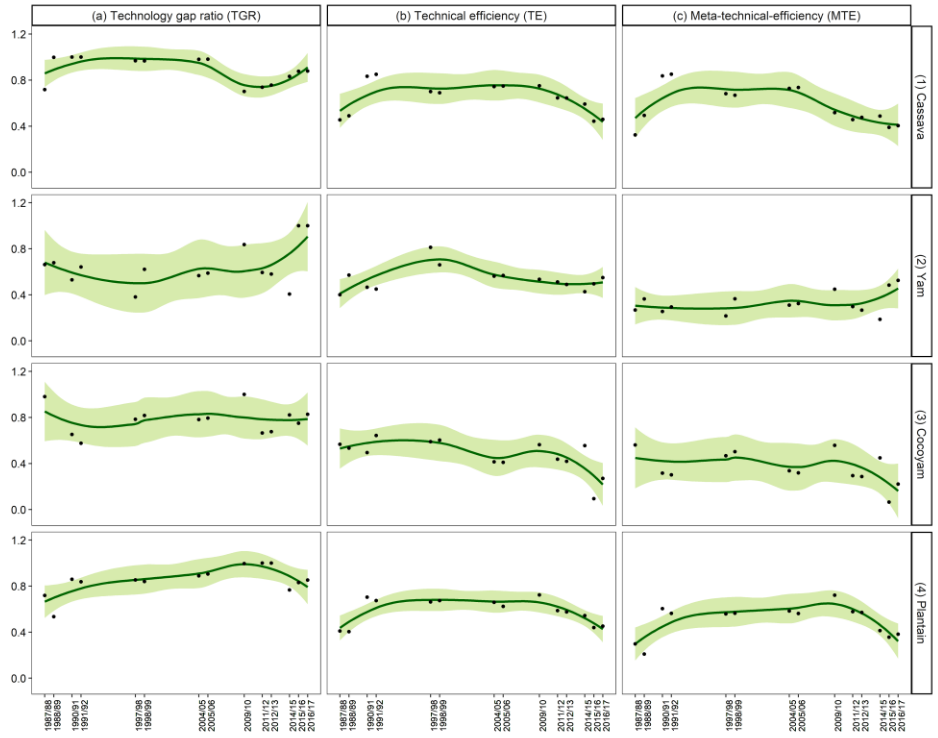

Figure 1. Ghanaian Starchy Staple Production Technology Level and Technical Efficiency Parameters Across Seasons (1987-2017)

Notes: Farmer-level scores were first estimated via a Meta−stochastic−frontier (MSF) analysis applied separately to 9 population−based surveys that represent 30 years of farmer-level data collection in Ghana the surveys used included Ghana Living Standards Surveys [wave 1−7] and Ghana Socioeconomic Panel Survey [wave 1−2]. The farmer-level elasticities were subsequently averaged across seasons via a regression framework to account for controls. Each point on a sub−panel represents the mean of the estimates. Given the seasonal means, the fitted line was done locally using

neighborhood points, weighted by distance. The size of the neighborhood was set to 75% of the points with a tri−cubic weighting. The gray region is the 95% confidence interval of the fitted line.

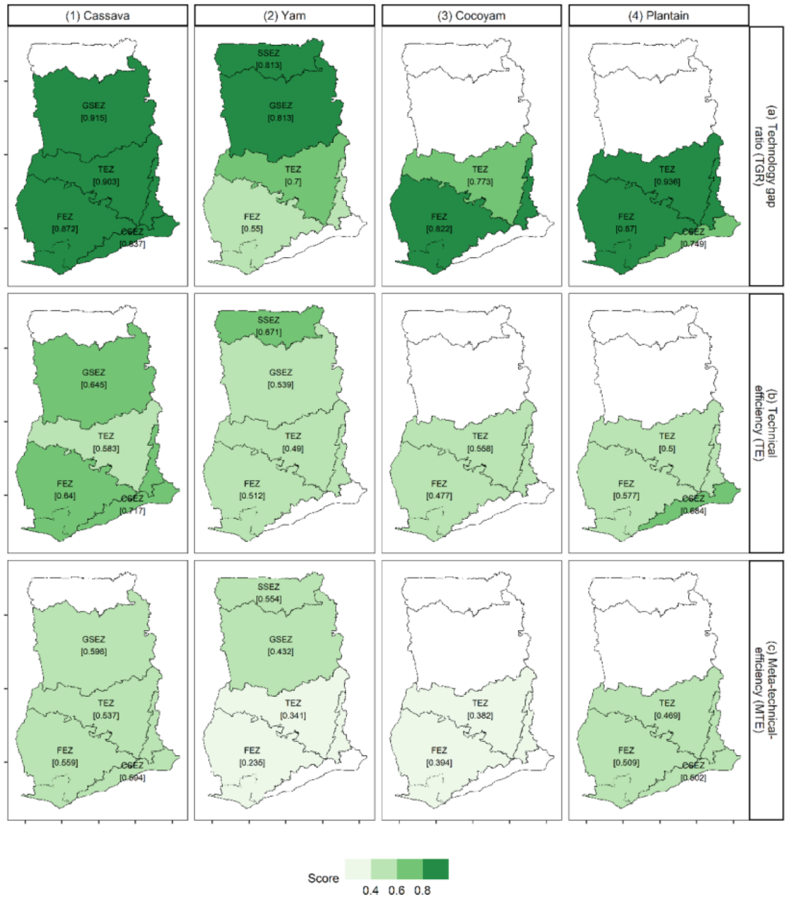

Sudan Savanna=SSEZ; Guinea Savanna=GSEZ; Transitional Zone= TEZ;Forest Zone=FEZ

Figure 2. Ghanaian Starchy Staple Production Technology Level and Technical Efficiency Across Ecologies (1987-2017)

Notes: Farmer-level scores were estimated via a Meta−stochastic−frontier (MSF) analysis applied separately to 10 population−based surveys that represent 30 years of farmer-level data collection in Ghana the surveys used included Ghana Living Standards Surveys [wave 1−7] and Ghana Socioeconomic Panel Survey [wave 1−2].

~~57. Specifically, the most technically efficient cassava farmers relative to the metafrontier are those in the GSEZ with an MTE of 0.60. Figure 2 shows that the GSEZ farmers are followed by their~~

~~peers in CSEZ (0.59), FEZ (0.56), and then TEZ (0.54). Figure 1, panel c1 shows that the MTE increased from 0.32 in 1987 to 1.00 in 1991 due to improvements in technology adoption. However, from 1991 to 2017, MTE declined at the same rate as pure farmer technical efficiency to 0.40. Generally, the interest of policies is to improve production via the introduction of new technologies or subsidize inputs [46] while less attention is given to mechanisms that can increase the efficiency of production. For instance, improved crop varieties often require complementary inputs (enabling environments such as credit to purchase fertilizer, among others) to produce optimal yields. However, such conditions often are not the focus of research [47] or government policies after introducing improved seeds to farmers or subsidized inputs. Overall, the high TGR and high resource wastage show that cassava MTE could be enhanced by improving TE.~~

~~5.3 Yam Production~~

~~Yam is significantly cultivated in four main ecological zones, which are the SSEZ, GSEZ, TEZ, and FEZ.~~ Figure 2 shows that the Guinea Savannah farmers are followed by their peers in Coastal Savannah (0.59), Forest zone (0.56), and then Transition zone (0.54). Figure 1, panel c1 shows that the MTE increased from 0.32 in 1987 to 0.85 in 1991 due to improvements in technology adoption. However, from 1991 to 2017, MTE declined at the same rate as pure farmer technical efficiency to 0.40. We return to the implications and policy learning points from these findings in the Discussion section.

4.3.2 Yam production

The mean TGR for yam, estimated at 0.~~72, is lower than that of cassava. On~~65, points to an average ~~the~~ ecological technology gap ~~for yam is~~of about ~~28~~35%. Figure 1, panel a2 shows that the TGR for yam has increased steadily over the study period~~.~~ but this generalization masks significant dips and peaks over time. For example, the TGR for yam dropped by about 4.25% annually in the

decade between 1987 (0.66) and 1997 (0.38); this rebounded to 0.62 in 1998 only to sink to 0.41 in 2014 before hitting another gear shift to peak at 1.00 in both 2016 and 2017. At the ecology level, the highest TGR is estimated for SSEZ/GSEZ (at 0.81), for Sudan Savannah/Guinea Savannah, followed by TEZthe Transition zone (0.70), and then FEZthe Forest zone (0.55).

Ecology specific TE for yam is estimated at a mean of 0.55 and has remained relatively constant over the study period (Figure 1, panel b2). Yam TE follows similar patterns as the TGR, the only difference being that FEZ (0.51) is higher than TEZ (0.49) (see Figure 2). The TE values for yam estimated in this study are relatively low when compared to values of 0.85 and 0.89 estimated by Owusu Asante et al. [19] for Ashanti and Brong Ahafo regions (all approximately located in the TEZ), respectively. Several things distinguish this study from Owusu Asante et al. (2014) that could account for the differences in TE. First, our study contains production data from multiple regions/ecologies throughout Ghana and multiple production seasons, while Owusu Asante et al. [19] used data from only 375 yam farmers from only one ecology and for only one season. Thus, the values estimated by Owusu Asante et al. [19] do not holistically capture the spatiotemporal dynamics of yam TE as this study does. Secondly, Owusu Asante et al. [19] utilized data that is over-represented by those farmers (58%) using yam mini-sett technology which the authors show induces positive and significant effects on the TE of some of its adopters. By the nature of its data, this study is not able to pinpoint the specific technologies used by each farmer. However, farmers in this study sample use several technologies which may include yam mini-sett technology.[2]

[2] TE scores are relative measures that track efficiency relative to the most efficient observations within a particular sample. Thus, as a caveat any difference between this study's estimates and previous works could simply be reflecting differences in heterogeneity across samples.

~~Accounting for ecology-specific production technologies shows that the mean yam MTE is 0.39.~~Ecology-specific TE for yam is estimated at a mean of 0.54 and has remained relatively constant over the study period (Figure 1, panel b2). Yam TE follows similar patterns as the TGR, the only difference being that the estimate for the Forest zone (0.51) is higher than the Transition zone (0.49) (see Figure 2). The TE values for yam estimated in this study are relatively low when compared to values of 0.85 and 0.89 estimated by Owusu Asante et al. [24] for Ashanti and Brong Ahafo regions, respectively (all approximately located in the Transition zone). Several things distinguish this study from Owusu Asante et al. [24] that could account for the differences in TE. First, our study contains production data from multiple regions/ecologies throughout Ghana and multiple production seasons, while Owusu Asante et al. [24] used data from only 375 yam farmers in one ecology and for only one season. Thus, the values estimated by Owusu Asante et al. [24] do not holistically capture the spatiotemporal dynamics of yam TE as this study does. Secondly, Owusu Asante et al. [24] utilized data that is over-represented by those farmers (58%) using yam mini-sett technology which the authors show induces positive and significant effects on the TE of some of its adopters. By the nature of its data, this study is not able to pinpoint the specific technologies used by each farmer. However, farmers in this study sample use several technologies which may include yam mini-sett technology.[3]

Accounting for ecology-specific production technologies shows that the mean yam MTE is 0.33. Like the TE, the MTE for yam is also constant across the study period but improves from ~~Southern~~southern to ~~Northern~~northern Ghana. Specifically, the most technically efficient yam

[3] TE scores are relative measures that track efficiency relative to the most efficient observations within a particular sample. Thus, a caveat is that any difference between this study's estimates and previous works could simply be reflecting differences in heterogeneity across samples.

farmers relative to the industrial production frontier are those in the ~~SSEZ~~Sudan Savannah zone, with an MTE of 0.55. Figure 2 shows that ~~the SSEZ~~Sudan Savannah farmers are followed by their peers in ~~GSEZ~~the Guinea Savannah (0.43), ~~TEZ~~the Transition (0.34), and then ~~FEZ~~the Forest (0.24~~).~~) zones. Even though there is a ~~substantial~~ technological gap of 28%, the overall results for yam show that yam MTE could be enhanced by improving TE.

~~These findings of inefficient~~ 4.3.3 Cocoyam production ~~outcomes~~

Formatted: Font: Bold

The mean TGR for cocoyam, estimated at 0.80, indicates that the ecological technology gap is about 20%. The temporal trend from Figure 1, panel a3 shows that except for dips in 1991/1992 and again in 2011/2012, the TGR for cocoyam has remained close to its mean value of 0.80 over the study period. This mimics the yam TGR dynamics, but at a lower magnitude. At the ecology level, the TGR for cocoyam is higher in the Forest zone (0.82) than in the Transition zone (0.77).

Ecology-specific TE for cocoyam is estimated at a mean of 0.47 across the entire sample. Unlike the TGR, the cocoyam TE has been declining at a rate of about 5% from 1987 to 2017 (Figure 1, panel b3). Contrary to the TGR, cocoyam TE is higher in the Transition (0.56) than in the Forest (0.48) zone, as depicted in Figure 2. The dynamics in TGR and TE culminate in an estimated mean MTE of 0.36 for cocoyam. This follows the temporal trend exhibited by TE but improves from northern to southern Ghana. The overall results for cocoyam show that production could be enhanced by improving TE.

4.3.4 Plantain production

The mean TGR for plantain (0.85) is second only to cassava among the starchy staples considered. This indicates a plantain technology gap of about 15%. Figure 1, panel a4 shows that the TGR for plantain peaked in the period between 2004/05 and 2012/13 and has generally increased by about 3% annually from 0.72 in 1987 to 0.85 in 2017. At the ecology level, plantain TGR decreases from

Transition zone (0.94), and Forest zone (0.87) to the Coastal Savannah zone (0.75). Thus, plantain farmers in the Transition zone are closest to the meta-frontier, while those in the Coastal Savannah zone can increase their productivity by about 25% if they adopt the best available technology.

Ecology-specific TE for plantain is estimated at a mean of 0.54 across the entire sample. The TE for plantain has remained constant over the study period (Figure 1, panel b4). The ecological zone ranking (high to low) of plantain TE is from Coastal Savannah (0.68), Forest (0.58), to the Transition (0.50) zones. In terms of MTE, the mean for plantain is 0.49. The MTE ranking (high to low) for plantain is from Forest (0.51) and Coastal Savannah (0.51) to the Transition zone (0.47). Plantain production could be enhanced by improving TE. The low MTE estimates for plantain production in this study are indicative of what has been reported in the literature. For instance, MoFA [5] indicated that actual average plantain yields represent only 32% of what is achievable; even the best-performing district attains less than 50% of the estimated potential yield.

4.4 Which factors affect starchy staple technical inefficiency and technology gaps in Ghana?

Table S7 reports the estimates of factors that influence TE and TGR across crops and ecologies. Results for the case of TE are captured by the ecology frontiers and that of the TGR is captured by the meta-frontier. With TE, negative [positive] values indicate that the variables reduce [increase] technical inefficiency. For the case of TGR, negative [positive] values indicate that the variables reduce [increase] the technology gap.

Formatted: Line spacing: Double, Tab stops: 3.83", Left

The following caveats are to be noted in interpreting results from the effects of farmer-specific and institutional variables on TE and TGR presented in Table S7. First, these variables were harmonized across different surveys and used mainly as controls in estimating the drivers of differences in starchy staples production outcomes in Ghana. Due to this level of aggregation, we assume only modest exogenous variation in the control variables and therefore do not claim

causality. Notwithstanding, given this study’s focus on establishing which of technological gaps or technical inefficiencies contribute more to production shortfalls, we believe the outcomes of these farmer characteristics and institutional factors can help to direct policies by examining their qualitative association with the outcomes of interest. To this end, we discuss these variables in terms of general associations and not in terms of individual coefficients.

4.4.1 Farmer Characteristics

Except for yam production in the Sudan Savannah zone, male farmers report higher TE scores than female farmers across crops and ecologies, but sex had no significant effect on the TE of plantain production. Also, the effect of sex on TGR was only limited to yam production where the results showed a technology gap of 0.47 between males and females. These results show that women are doubly disadvantaged in starchy staples production as they are both less technically efficient and have higher technology gaps when compared to their male counterparts. These phenomena are supported by literature for rice [10], yam [24], and vegetables [23]. Results in Table S7 show that age reduces inefficiencies in starchy staple production. At the national level, these effects are more pronounced in yam production, modest for cassava and plantain, and nonexistent for cocoyam. Thus, age has generally positive associations with TE and technological gaps. The nonlinear effect of age is only present for cassava production. Older farmers tend to be more inefficient in starchy staples production and exhibit wider gaps in adopting modern technologies. Owusu Asante et al. [24] found similar effects of age on yam farmers’ technical inefficiency in Ghana’s Ashanti and Brong-Ahafo regions. Education also showed non-linear effects only for yam production efficiency but not for the other starchy staples. Mostly, education was negatively associated with technical efficiency and adoption of superior technologies for yam production.

4.4.2 Institutional Factors

Where statistically significant, general results for land ownership show that farm households that own lands are more technically efficient than those farming on rented or communal lands. Land tenure security negatively influences farmers' technical inefficiency for all crops and ecologies except cocoyam farmers in the Transition zone. This highlights the positive effect of tenure security: we would expect landowners to be less risk averse and more incentivized to invest in management practices that improve soil fertility and land structure, and that eventually would lead to more efficient crop production than non-owners of land [10]. On the other hand, the effect of ownership on bridging technology gaps appears inconclusive. On its own, land ownership may not be an enough incentive to adopt and maintain the application of modern technologies. Modern production technologies may require significant extra labor and capital investment to apply. In such instances, resource-constrained farmers may rather opt for conservative land management techniques that assure a satisfactory level of output.

Access to agricultural extension services often plays a key role in helping farmers improve their crop production by promoting good agronomic practices and the adoption of better technology that improves efficiency. Our results confirm this *a priori* expectation by their generally negative associations with TE and TGR. Access to extension services reduced both technical inefficiency and technology gaps of yam and plantain production, but these effects were insignificant for cassava and cocoyam. As in this study, Owusu Asante et al. [24] also found that extension access reduced production inefficiency. Similarly, Egyir et al. [38] found that access to extension services increased plantain farmers' adoption of agro-chemicals which ultimately improved yields.

Formatted: Font: Italic

Access to credit is expected to enable farmers to acquire supporting inputs necessary to increase efficiency and productivity. The results in Table S7 show generally positive associations with both TE and TGR. Access to production credit increased both technical inefficiencies and technology

gaps for cassava and yam production, but reduced technology gaps for plantain production. This counterintuitive finding on the negative effect of credit on TE and TGR is difficult to explain from the available data, but a possible prediction relates to the exact use of the accessed credit amounts. Do farmers invest credit funds in their starchy staple production? Oftentimes, farmers use the credit for other purposes than farm investment [39].

Access to mechanized services showed heterogeneous effects on starchy staple production. While mechanized services increased the technology gaps for plantain and cassava, it reduced technical inefficiency and technology gaps for yam production. This mixed result is not surprising and may be reflective of the heterogenous access to mechanized services across production ecologies and among farmers in Ghana [40]. That said, we are mindful of claiming too much inference to this finding given the conditions.

Formatted: Line spacing: Double, Tab stops: 3.83", Left

5. Discussion

The literature identifies technical inefficiencies and technology gaps as the main causes of low productivity in smallholder agricultural systems. Our discussion is tilted towards addressing the main question of the paper, which is to ascertain whether starchy staples production shortfalls in Ghana are predominantly driven by technical inefficiencies, technological gaps or both. As indicated, the technological gap depends on farmers' access to and adoption of the best production technology and shows how close the production technology in an ecology is to the best technology set. On the other hand, technical efficiency is driven by the ability and willingness of the farmer to properly use and combine the available input sets. Thus, management practices and enabling environment as opposed to new technologies, are key determinants of technical efficiency.

The results in Table 4 show that for cassava, yam, and plantain, agroecological technologies in northern Ghana tend to be closer to the meta-frontier technology indicating low technological gaps; however, the technology gaps increase consistently as one traverses southwards. Unlike the technological gaps, technical inefficiency scores for the various agro-ecologies did not seem to follow any pattern across space or time. Acheampong et al. [41] examined cocoyam farmers' rates of adopting improved technologies and found that farmers in the Transition zone had higher adoption rates than those in the Forest zone. Their results further showed that the high adoption of complementary production techniques like appropriate planting distance, timely weed control, and weedicide application in the Transition zone improved the TGR for that zone.

The MTE provides a basis for comparing the efficiency of starchy staple production for all agro-ecologies relative to the best available technology. The key observation from these results is that the technical inefficiency effects across all ecologies, as well as at the national level, outweigh the technological gap effects. Thus, even though both technical inefficiency and technological gaps exist across all ecologies, technical inefficiencies are always higher than technological gaps. These results suggest more room for improving the efficiency of producing all crops in all agro-ecologies. For example, while cassava production in the Guinea Savannah zone is the most efficient relative to the meta-frontier, production levels can still be increased by about 40% if the inefficiency effects are eliminated, given the prevailing input levels and production technologies. What could be the possible explanation for the trends we observe, where technical inefficiency effects tend to outweigh the technological gaps?

Over the years, the interest of policies in many developing countries, including Ghana, has been to improve production levels mainly through the introduction of new technologies or input subsidies [42,43] while less attention is given to mechanisms that can increase the efficiency of

production. For instance, while Ghana cocoa sector has benefited from several fertilizer subsidy schemes, it is well documented that inappropriate use of the subsidized fertilizer in terms of timing and application rates was a common production problem [44]. It has also been found that providing subsidized fertilizer in addition to training on best management practices via farmer field schools results in relatively more benefits in terms of increased yields than providing subsidized fertilizer alone [45]. These observations suggest that increasing farmer access to improved/new technology (improved crop varieties, fertilizer, etc..,) solves only one part of the problem: human capital investments and training on proper technology usage are also needed. However, such add-on outreach activities often are not the focus of most programs or they get sidelined during the implementation stages of government policies (as was the case of the Root and Tuber Improvement Program [RTIP] (1999-2004)] and its successor, Root and Tuber Improvement and Marketing Program [RTIMP] (2005-2015) [46,47]. The focus of the RTIP was on the multiplication and distribution of improved planting materials and integrated pest management, with less attention given to complementary mechanisms that increase technical efficiency.

A closer look at the performances of individual starchy staples reinforces the roles of policies and complementary inputs in efficient crop production. From the MTE scores, the order of best performing starchy staples were cassava, plantain, cocoyam, and yam. The higher efficiency scores for cassava might be attributed to the prominent attention it received in policies and programs such as the President's Special Initiative (PSI) on agribusiness and cassava (2002-2004), the RTIP and RTIMP, as well as the West Africa Agricultural Productivity Program [WAAPP] (2007-2017) that aimed at increasing productivity of starchy staples in Ghana [54].

The finding that yam is the most inefficiently produced starchy staple in Ghana is worrying for several reasons. Ghana is the world's second-largest producer of yam (behind Nigeria) but the

leading exporter to the global market [48,49]. Given that about 94% of yam produced in Ghana is destined for the international market, ~~eliminating~~addressing the causes of production shortfalls has the potential to improve the livelihoods of actors in the yam value chain. At the local levels, yam holds keystone positions in the cultural expression of several tribes in Ghana [50,51] and remains an essential food security crop. For instance, yam consumption per capita stood at 125kg in 2015 [49]; however, about 40% of yam harvested in the ~~GSEZ~~Guinea Savannah zone (the largest yam producing area) is lost through poor postharvest handling [48]. This, ~~along~~coupled with resource use inefficiencies, threatens the capacity of yam to contribute to ~~Ghana attaining the~~achieving Ghana's SDG 2 targets ~~of SDG 2~~.

~~**5.4 Cocoyam Production**~~

~~Cocoyam production dominates only in two agroecological zones (TEZ and FEZ). This is hardly surprising since these areas are also famous for their high cocoa production. Traditionally, cocoyam was grown as a complement to provide shade for young cocoa seedlings [52]. Even though cocoyam production has since expanded beyond this basic use, its proliferation in the country has been largely restricted to specific areas in southern Ghana. Coupled with the fact that the cocoyam was originally introduced in the forest belt, the dominance of cocoyam production in the TEZ and FEZ has been mainly attributed to the wide vegetation cover that the forest belt provides as well as the well-distributed rainfall amounts [53]. This assertion is given credence by the fact that the FEZ (Semi-Deciduous and Rain Forests) and TEZ recorded the highest average rainfall estimates of all ecological zones in this study (Table S1).~~

~~The mean TGR for cocoyam is lower than the estimates for cassava but higher than for yam. The estimated cocoyam TGR of 0.80 indicates that the ecological technology gap is about 20%. Figure 1, panel a3 shows that the TGR for cocoyam has remained at a higher value of 0.80 over the study~~

Formatted: Font: Bold

Formatted: Font: Bold

Formatted: Font: Bold

Formatted: Font: Bold

period. This reflects the same dynamics in the yam TGR but at a lower magnitude. At the ecology level, the TGR for cocoyam is higher in the FEZ (0.82) than in the TEZ (0.77).

As indicated, TGR is dependent on the accessibility and adoption level of frontier enhancing technologies. For cocoyam production in Ghana, Acheampong, Owusu, and Nurah [47] examined the rates of adopting improved technologies, the determinants of adoption, and the benefits of adopting cocoyam technologies to farmers. The results of their analysis partly resonate with ours because they drew their sample from similar ecological zones. Among others, Acheampong, Owusu, and Nurah [47] found that only about 22% of farmers adopted improved planting material varieties although more than 60% were aware of their availability. Similarly, low rates of adoption were realized for agronomic practices such as the application of manure, inorganic fertilizer, cover cropping, minimum tillage, and row planting. However, there were high adoption levels for techniques such as appropriate planting distance, timely weed control, and weedicide application. With regards to the determinants of adoption, they found that experienced farmers and farmers whose farm plots were far removed from their homestead had lower probabilities of adopting all technologies. The opposite was observed for farmers that belonged to farmers' associations primarily because they were more aware of the benefits of new technologies through higher social integration. The study reported significant gender and ecological variations although unlike in this study, they found higher adoption among farmers in the TEZ than those in the FEZ.

Ecology-specific TE for yam is estimated at a mean of 0.52 across the entire sample. The finding that persistent technical inefficiency is relatively more of a challenge to productivity improvement than technology gaps across all starchy staples considered in this study means that critical attention must be given to the enabling factors that foster productivity growth rather than searching for new technologies all the time. Already, smallholder farmers are risk averse, which affect their

production decisions and adoption of new technologies [52]. Therefore, continuously exposing these farmers to new technologies without first improving the enabling environment that foster their adoption may be counterproductive. Studies have found that management and institutional variables such as extension services are important drivers of technology adoption and productivity [38,53]. Our results in Table S7 support this assertion, as we found that both TE and TGR for some starchy staples are significantly affected by access to extension services.

Unlike the TGR, the cocoyam TE has been declining at a rate of about 5% from 1987 to 2017 (Figure 1, panel b3). Contrary to the TGR, cocoyam TE is higher in TEZ (0.56) than in the FEZ (0.48) as depicted in Figure 2.

The dynamics in TGR and TE culminate in an estimated mean MTE of 0.39 for cocoyam. This follows the temporal trend exhibited by TE but improves from Northern to Southern Ghana. The overall results for cocoyam show that production could be enhanced by improving TE. The estimated RTS parameter for cocoyam (0.46) is the lowest for all the starchy staples considered in this study. Just like for cassava and yam, the implications are that extensification of production (for example, through area expansion) is not a feasible entry point to change the status quo.

5.5 Plantain Production

Like yam and cocoyam, plantain has traditionally been integrated as a complementary crop to cocoa. Its broad leaves and succulent stems provide ecosystem services to young cocoa seedlings. Hence, our finding that plantain cultivation takes place in three ecological zones, namely, the TEZ, FEZ, and CSEZ is to be expected as these zones describe the "cocoa belt" of Ghana. Apart from providing shade for cocoa seedlings, smallholder farm households in Ghana secure their food by integrating plantain into tree crop plantations to offset the income gap between plantation establishment and commercial yields.

Plantain is integral to the food security and poverty alleviation efforts of Ghana. Its utilization in the diets of the producing areas is varied and has been increasing in recent times (MoFA, 2019). Results from Table 3 show that land has the highest output elasticity across all ecological zones and is followed by family labor, hired labor, pesticide, and then planting materials. Like the other starchy staples, plantain production exhibits DRS in all ecological zones and nationally. The estimated RTS of 0.63 implies that a 1% increase in all inputs will yield about a 0.63% increase in plantain output. Practically, this means the cost-saving or profit returns of increasing all the factors of plantain production have been attained, given the present technology.

The mean TGR for plantain (0.90) is the highest across all the starchy staples considered. This indicates a plantain technology gap of about only 10%. Figure 1, panel a4 shows that the TGR for plantain has increased approximately 3% annually from 0.72 in 1987 to 0.85 in 2017. At the ecology level, plantain TGR decreases from TEZ (0.94), and FEZ (0.87) to CSEZ (0.75). Thus, plantain farmers in the TEZ are closest to the meta-frontier, while those in the CSEZ can increase their productivity by about 25% if they adopt the best available technology.

Ecology-specific TE for plantain is estimated at a mean of 0.54 across the entire sample. The TE for plantain has remained constant over the study period (Figure 1, panel b4). The ecological zone ranking (high to low) of plantain TE is from CSEZ (0.68), FEZ (0.58), to TEZ (0.50). In terms of MTE, the mean for plantain is 0.49. The MTE ranking (high to low) for plantain is from FEZ (0.51) and CSEZ (0.51) to TEZ (0.47). Plantain production could be enhanced by improving TE. The low MTE estimates for plantain production in this study are indicative of what has been reported in the literature. For instance, MoFA [2] indicated that actual average plantain yields represent only 32% of what is achievable; even the best-performing district attains less than 50% of the estimated potential yield.

6. Conclusions

~~Improvements in crop productivity by closing production gaps in rural economies are generally touted as an important pathway to achieving three main developmental goals, namely food security, reduced poverty, and sustainable resource utilization. Yet~~In developing countries, many farmers unwittingly waste significant productive resources through production and technological inefficiencies which ~~engender~~entrench poverty and food insecurity. Changing the status quo and fostering rapid economic transformation would require sustained improvements in agricultural productivity. In this study, we take advantage of a large Ghanaian dataset that spans over three decades and examine the spatiotemporal trends of production gaps of one of the most important staple crop categories, the starchy staples. Unlike similar studies thus far, and in addition to the unique dataset used, the method adopted accounts for technological heterogeneity across the main ecological zones in Ghana where these starchy staple crops are cultivated. Using the stochastic meta-frontier approach, technical efficiency, meta-technical efficiency, and technology gap ratios are measured across crops and ecological zones. This analysis enables us to provide a broad understanding of the ~~core drivers~~major sources of ~~productivity~~production shortfalls within the starchy staples industry~~, provide suggestions for generating major growth points, and the limitations that need critical policy attention~~ of Ghana.

The key findings suggest that there are significant shortfalls in starchy staples production~~ among agroecological zones~~, which could be ~~more~~ attributed to pure farmer technical ~~efficiencies~~inefficiencies and less to technology gaps~~. Stunningly, the~~ along agroecological lines. The results show that the current set of production technologies used by starchy staple farmers in Ghana across ecologies are quite similar as the ecological technology gap that exists is about 20% ~~across~~for all ~~four~~ crops~~.~~ except yam which has a technology gap of 35%. On the other hand, there

exist significant production shortfalls that could be attributed to pure farmer technical inefficiencies. Respectively, about ~~37, 45, 48~~35, 46, 53, and ~~46~~45% of cassava, yam, cocoyam, and plantain production is lost due solely to technical inefficiencies and these have been relatively constant or ~~at worst increased~~increasing from 1987 to 2017. These ~~dynamics~~trends suggest that ~~over time, limited attention might have been given to interventions that inure to~~mechanisms that improve the efficiency of resource use among starchy staple farmers have not received the needed attention over time. With the large production shortfalls attributable mostly to technical inefficiency, one implication of our results is that the starchy staple crop sector could benefit from improvements in conditions that enhance farmer technical efficiencies, such as providing starchy staple farmers more access to complementary production inputs such as extension services and ~~improved inputs~~better agronomic practices.

Further, the results also show that for any given starchy staple crop, some ecological zones perform better than others, thereby indicating heterogeneity in both technology and production management practices. We generally find that across all the crops considered, the technology gap across ecological lines is minimal but pure farmer technical inefficiency is chronic. This means that the production shortfalls for starchy ~~stables~~staples in Ghana could be reduced if the causes of pure farmer technical inefficiency ~~is~~are adequately addressed. Existing policies~~,~~ aimed at improving the technology level (e.g., those supplying improved planting materials and subsidized inputs) ~~should~~could also emphasize the efficient use of these technologies. Stakeholders could ~~investigate the documentation of~~learn important lessons from the frontier enhancing technologies and practices in high-performing ecological zones, improve them further and disseminate such improved technologies to less-performing agro-ecologies, guided, of course, by contextual factors.

References

1. FAO, IFAD, UNICEF, WFP~~, WHO~~ W. The State of Food Security and Nutrition in the World ~~2018. Building climate resilience for food security~~2022. FAO; 2022. doi:10.4060/cc0639en

2. GSS/MOFA/WFP/FAO. Comprehensive Food Security and ~~nutrition. Rome, FAO. 2018.~~Vulnerability Analysis (CFSVA) GHANA. 2020 [cited 13 Mar 2023]. Available: ~~www.fao~~https://docs.wfp.org/~~publications~~api/documents/WFP-0000137744/download/

~~2~~3. FAO. The State of Food and Agriculture 2022. FAO; 2022. doi:10.4060/cb9479en

4. Pradhan P, Fischer G, van Velthuizen H, Reusser DE, Kropp JP. Closing Yield Gaps: How Sustainable Can We Be? Gonzalez-Andujar JL, editor. PLoS One. 2015;10: e0129487. doi:10.1371/journal.pone.0129487

5. Ministry of Food and Agriculture [MOFA]. Agriculture in Ghana: Facts and figures (2018). 2019.

~~3~~6. Ladha JK, Jat ML, Stirling CM, Chakraborty D, Pradhan P, Krupnik TJ, et al. Achieving the sustainable development goals in agriculture: The crucial role of nitrogen in cereal-based systems. Advances in Agronomy. 2020. pp. 39–116. doi:10.1016/bs.agron.2020.05.006

7. Abdulai A, Huffman W. Structural Adjustment and Economic Efficiency of Rice Farmers in Northern Ghana. Econ Dev Cult Change. 2000;48: 503–520. doi:10.1086/452608

~~4~~8. Anang BT, Bäckman S, Sipiläinen T. Technical efficiency and its determinants in smallholder rice production in northern Ghana. J Dev Areas. 2016;50: 311–328. doi:10.1353/jda.2016.0072

~~5~~9. Donkor E, Matthews N, Ogundeji AA. Efficiency of Rice Farming in Ghana: Policy Implications for Rice Sector Development. African Dev Rev. 2018;30: 149–161. doi:10.1111/1467-8268.12320

~~6~~10. Owusu V, Donkor E, Owusu-Sekyere E. Accounting for the Gender Technology Gap Amongst Smallholder Rice Farmers in Northern Ghana. J Agric Econ. 2018;69: 439–457. doi:10.1111/1477-9552.12236

~~7~~11. Asravor J, Wiredu A, Siddig K, Onumah E. Evaluating the Environmental-Technology Gaps of Rice Farms in Distinct Agro-Ecological Zones of Ghana. Sustainability. 2019;11: 2072. doi:10.3390/su11072072

~~8~~12. Tsiboe F, Asravor J, Owusu V, Mensah-Bonsu A. Production technology, efficiency, and productivity of cereal farms: Prospects for enhancing farm performance in Ghana. Agric Resour Econ Rev. 2022; 1–31. doi:10.1017/age.2022.16

~~9~~13. Abdulai S, Nkegbe PK, Donkoh ~~S a~~SA. Technical efficiency of maize production in Northern Ghana. African J Agric ~~Reseearch~~Research. 2013;8: 5251–5259. doi:10.5897/AJAR2013.7753

~~10~~14. Owusu V. Technical Efficiency Of Technology Adoption By Maize Farmers In Three Agro-Ecological Zones Of Ghana. Rev Agric Appl Econ. 2016;19: 39–50. doi:10.15414/raae.2016.19.02.39-50

Formatted: Justified

Formatted: Justified

Formatted: Dutch (Netherlands)

Formatted: Justified

~~11~~15. Wongnaa CA, Awunyo-Vitor D. Scale efficiency of maize farmers in four agro ecological zones of Ghana: A parametric approach. J Saudi Soc Agric Sci. 2019;18: 275–287. doi:10.1016/j.jssas.2017.08.003

~~12~~16. Avea A, Zhu J, Tian X, Baležentis T, Li T, Rickaille M, et al. Do NGOs and Development Agencies Contribute to Sustainability of Smallholder Soybean Farmers in Northern Ghana—A Stochastic Production Frontier Approach. Sustainability. 2016;8: 465. doi:10.3390/su8050465

~~13~~17. Awunyo-~~vitor~~Vitor D, Bakang ~~J a~~JA, Gyan SK, Cofie S. Estimation of Farm Level Technical Efficiency of Small-Scale Cowpea Production in Ghana. Am J Agric Environ Sci. 2013;13: 1080–1087. doi:10.5829/idosi.aejaes.2013.13.08.11013

~~14~~18. Etwire PM, Martey E, Dogbe W. Technical Efficiency of Soybean Farms and Its Determinants in Saboba and Chereponi Districts of Northern Ghana: A Stochastic Frontier Approach. Sustain Agric Res. 2013;2: 106. doi:10.5539/sar.v2n4p106

~~15~~19. Onumah JA, Onumah EE, Al-Hassan RM, Brümmer B. Meta-frontier analysis of organic and conventional cocoa production in Ghana. Agric Econ (Czech Republic). 2013.

20. Besseah FHFA, Kim S. Technical Efficiency of Cocoa Farmers in Ghana. J Rural Dev. 2014;37: 159–182. Available: http://ageconsearch.umn.edu/record/196615?ln=en

~~16~~21. Danso-Abbeam G, Baiyegunhi LJS. Technical efficiency and technology gap in Ghana's cocoa industry: accounting for farm heterogeneity. Appl Econ. 2019; 1–13. doi:10.1080/00036846.2019.1644439

~~17~~22. Tsiboe F. Chronic sources of low cocoa production in Ghana: new insights from meta-analysis of old survey data. Agric Resour Econ Rev. 2021; 1–26. doi:10.1017/age.2021.3

~~18~~23. Tsiboe F, Asravor J, Osei E. Vegetable Production Technical Efficiency and Technology Gaps in Ghana. African J Agric Resour Econ. 2019;14. Available: http://afjare.org/wp-content/uploads/2020/01/3.-Tsiboe-et-al.pdf

~~19~~24. Owusu Asante B, Villano RA, Battese GE. The effect of the adoption of yam minisett technology on the technical efficiency of yam farmers in the forest-savanna transition zone of Ghana. African J Agric Resour Econ. 2014.

~~20~~25. Tsiboe F, Aseete P, Djokoto JG. Spatiotemporal Comparison of Dry Beans and Groundnut Production Technology and Inefficiency in Ghana. Rev Agric Appl Econ. 2021;24: 76–87. doi:10.15414/raae.2021.24.01.76-87

~~21~~26. Battese GE, Rao DSP, O'Donnell CJ. A Metafrontier Production Function for Estimation of Technical Efficiencies and Technology Gaps for Firms Operating Under Different Technologies. J Product Anal. 2004;21: 91–103. doi:10.1023/B:PROD.0000012454.06094.29

~~22~~27. Adaku AA, Tsiboe F, Clottey J. Taking ~~Stock~~stock of ~~Gender Gaps~~gender gaps in ~~Crop Production Technology Adoption~~crop production technology adoption and ~~Technical Efficiency~~technical efficiency in Ghana. ~~In: Working paper. Department of Agricultural Economics and Agribusiness, University of Ghana, P. O. Box 68, Legon, Accra, Ghana.~~ Agrekon. 2023; 1–18. doi:10.1080/03031853.2022.~~-~~2150664

Formatted: Justified

~~23~~28. Huang CJ, Huang T-H, Liu N-H. A new approach to estimating the metafrontier production function based on a stochastic frontier framework. J Product Anal. 2014. doi:10.1007/s11123-014-0402-2

~~24~~29. World Bank. World Development Indicators. In: World DataBank [Internet]. 2018 [cited 24 Sep 2018] p. 136. Available: https://data.worldbank.org/products/wdi

~~25. Farrell MJ. The Measurement of Productive Efficiency. J R Stat Soc Ser A. 1957;120: 253. doi:10.2307/2343100~~

~~26. Belotti F, Daidone S, Ilardi G, Atella V. Stochastic frontier analysis using Stata. Stata J. 2013;13: 719–758.~~

~~27. Meeusen W, van Den Broeck J. Efficiency Estimation from Cobb-Douglas Production Functions with Composed Error. Int Econ Rev (Philadelphia). 1977;18: 435. doi:10.2307/2525757~~

~~28. Aigner D, Lovell CAK, Schmidt P. Formulation and estimation of stochastic frontier production function models. J Econom. 1977;6: 21–37. doi:10.1016/0304-4076(77)90052-5~~

~~29. Charnes A, Cooper WW, Rhodes E. Measuring the efficiency of decision making units. Eur J Oper Res. 1978;2: 429–444. doi:10.1016/0377-2217(78)90138-8~~

~~30. Charnes A, Cooper WW, Rhodes E. Evaluating Program and Managerial Efficiency: An Application of Data Envelopment Analysis to Program Follow Through. Manage Sci. 1981;27: 668–697. doi:10.1287/mnsc.27.6.668~~

~~31. Morse S. A meta-analysis of the technical efficiency of yam production in Nigeria. J Crop Improv. 2021;35: 69–95. doi:10.1080/15427528.2020.1795772~~

~~32. Ojo MP, Ayanwale AB. Value chain financing and plantain production in Nigeria: an ex-ante approach. Financ Innov. 2019;5: 18. doi:10.1186/s40854-019-0132-6~~

~~33. Ebewore SO, Isiorhovoja RA. Knowledge Status and Disease Control Practices of Cassava Farmers in Delta State, Nigeria: Implications for Extension Delivery. Open Agric. 2019;4: 173–186. doi:10.1515/opag-2019-0017~~

~~34. Ogundari K, Brümmer B. Estimating Technical Efficiency, Input substitution and complementary effects using Output Distance Function: A study of Cassava production in Nigeria. Agric Econ Rev. 2011;12: 62–79. doi:10.22004/ag.econ.95773~~

~~35. Rahman S, Awerije BO. Technical and scale efficiency of cassava production system in Delta State, Nigeria: an application of Two-Stage DEA approach. J Agric Rural Dev Trop Subtrop. 2015;116: 59–69. Available: http://nbn-resolving.de/urn:nbn:de:hebis:34-2015011347155~~

~~36. Fu H, Qu Y, Pan Y. Efficiency of Cassava Production in China: Empirical Analysis of Field Surveys from Six Provinces. Appl Sci. 2018;8: 1356. doi:10.3390/app8081356~~

~~37. Hayami Y. Sources of Agricultural Productivity Gap among Selected Countries. Am J Agric Econ. 1969;51: 564. doi:10.2307/1237909~~

38. Hayami Y, Ruttan VW. Agricultural Productivity Differences among Countries. The American Economic Review. American Economic Association; 1970. pp. 895–911. doi:10.2307/1818289

39. Hayami Y, Ruttan VW. Agricultural development: an international perspective. Agric Dev an Int Perspect. 1971 [cited 19 Jul 2018]. Available: https://www.cabdirect.org/cabdirect/abstract/19721890134

40.30 Tsiboe F. Nationally Representative Farm/Household Level Dataset on Crop Production in Ghana from 1987-2017. In: SSRN [Internet]. 2020. doi:10.2139/ssrn.41345181

Formatted: Justified

41. Institute of Statistical Social and Economic Research (ISSER), Economic Growth Center (EGC). Ghana - Socioeconomic Panel Survey: 2009-2010. In: Microdata Library. GHA_2009_GSPS_v01_M [Internet]. 2016 [cited 9 Nov 2018]. Available: http://microdata.worldbank.org/index.php/catalog/2534

4231. Tsiboe F. Nationally Representative Farm/Household Level Dataset on Crop Production in Ghana from 1987-2017. In: SSRN [Internet]. 2020. doi:10.2139/ssrn.41345181

32. Ghana Statistical Service [GSS]. 2010 Population and Housing Census Summary Results of Final Report. Accra,Ghana; 2012. Available: www. statsghana.gov.gh

4333. Asante BO, Temoso O, Addai KN, Villano RA. Evaluating productivity gaps in maize production across different agroecological zones in Ghana. Agric Syst. 2019;176: 102650. doi:10.1016/j.agsy.2019.102650

34. Bokusheva R, Hockmann H. Production risk and technical inefficiency in Russian agriculture. Eur Rev Agric Econ. 2005;33: 93–118. doi:10.1093/erae/jbi036

35. Van Nguyen Q, Pascoe S, Coglan L, Nghiem S. The sensitivity of efficiency scores to input and other choices in stochastic frontier analysis: an empirical investigation. J Product Anal. 2021;55: 31–40. doi:10.1007/s11123-020-00592-8

Formatted: Justified

44. Caudill SB, Ford JM, Gropper DM. Frontier Estimation and Firm-Specific Inefficiency Measures in the Presence of Heteroscedasticity. J Bus Econ Stat. 1995;13: 105. doi:10.2307/1392525

45. Asante BO, Temoso O, Addai KN, Villano RA. 36. Coelli T. Estimators and hypothesis tests for a stochastic frontier function: A Monte Carlo analysis. J Product Anal. 1995;6: 247–268. doi:10.1007/BF01076978

37. Gutierrez RG, Carter S, Drukker DM. On boundary-value likelihood-ratio tests. Stata Tech Bull. 2001;10. Available: https://econpapers.repec.org/article/tsjstbull/y_3a2001_3av_3a10_3ai_3a60_3asg160.htm

38. Egyir IS, Owusu- Benoah E, Anno- Nyako FO, Banful B. Assessing the factors of adoption of agrochemicals by plantain farmers in Ghana. McKague K, editor. J Enterprising Communities People Places Glob Econ. 2011;5: 83–97. doi:10.1108/17506201111119617

39. Appiah-Twumasi M, Donkoh SA, Ansah IGK. Farmer innovations in financing smallholder maize production in Northern Ghana. Agric Financ Rev. 2019;80: 421–436. doi:10.1108/AFR-05-2019-0059

40. Appiah-Twumasi M, Donkoh SA, Ansah IGK. Innovations in smallholder agricultural financing and economic efficiency of maize production in Ghana's northern region. Heliyon. 2022;8: e12087. doi:10.1016/j.heliyon.2022.e12087

41. Acheampong P, Osei-Adu J, Amengor NE, Sagoe R. Cocoyam value chain and benchmark study in Ghana. In: A report submitted to West African Agricultural Productivity Project (WAAPP). Project Information Document. 2014.

Formatted: Justified

42. Houssou N, Andam KS, Collins A-A. Can better targeting improve the effectiveness of Ghana's Fertilizer Subsidy Program?: Lessons from Ghana and other countries in Africa south of the Sahara. In: IFPRI Discussion Paper 1605. Washington, D.C.: International Food Policy Research Institute (IFPRI) [Internet]. 2017 [cited 9 May 2017]. Available: http://ebrary.ifpri.org/cdm/ref/collection/p15738coll2/id/131068

43Evaluating productivity gaps in maize production across different agroecological zones in Ghana. Agric Syst. 2019;176: 102650. doi:10.1016/j.agsy.2019.102650

Formatted: Justified

46. Tsiboe F, Egyir IS, Anaman G. Effect of fertilizer subsidy on household level cereal production in Ghana. Sci African. 2021;13: e00916. doi:10.1016/j.sciaf.2021.e00916

47. Acheampong PP, Owusu V, Nurah G. How does Farmer Preference matter in Crop variety Adoption? The case of Improved Cassava varieties' Adoption in Ghana. Open Agric. 2018;3: 466–477. doi:10.1515/opag-2018-0052

44. Opoko E, Dzene R, Caria S, Teal F, Zeitlin A. Impacts of Group based Microfinance in Agriculture: Evidence from Ghana's Cocoa Abrabopa Association. In: Annual conference of Center for the Study of African Economies (CSAE) 2009 [Internet]. Oxford,United Kingdom: University of Oxford; 2009. Available: http://www.csae.ox.ac.uk/conferences/2009-edia/papers/473-CariaTealZeitlin.pdf

45. Tsiboe F, Dixon BL, Nalley LL, Popp JS, Luckstead J. Estimating the impact of farmer field schools in sub-Saharan Africa: the case of cocoa. Agric Econ. 2016;47: 329–339. doi:10.1111/agec.12233

46. International Fund for Agricultural Development [IFAD]. Republic of Ghana: Root and Tuber Improvement Programme (RTIP). In: Interim Evaluation Report October 2004. Report Nº 1533-GH. Independent Office of Evaluation. International Fund for Agricultural Development, West and Central Africa Division Programme Management Department. [Internet]. 2004 [cited 13 Mar 2023]. Available: file:///C:/Users/ftsib/Downloads/gh_rtip.pdf

47. International Fund for Agricultural Development [IFAD]. Republic of Ghana: Root and Tuber Improvement and Marketing Programme RTIMP Project Performance Evaluation Report. In: July 2018 Report No. 4813-GH. Independent Office of Evaluation. International Fund for Agricultural Development, West and Central Africa Division Programme Management Department. [Internet]. 2018 [cited 13 Mar 2023]. Available: https://www.ifad.org/documents/38714182/40811021/ghana_1312_2018.pdf/6e2a36e1-c84b-452d-9747-d84ac8d6c27a

48. Ansah IGK, Tetteh BKD. Determinants of Yam Postharvest Management in the Zabzugu District of Northern Ghana. Adv Agric. 2016;2016: 1–9. doi:10.1155/2016/9274017

Formatted: Justified

49. Acheampong P, Owusu Danquah E, Dissanayake H, Hayford P, Weebadde C. A Socioeconomic Study of Transition Zone Yam Farmers Addressing Constraints and Exploring Opportunities for Integrating Pigeonpea into Yam Cropping Systems. Sustainability. 2019;11: 717. doi:10.3390/su11030717

50. Odotei I. Festivals in Ghana: Continuity, transformation and politicisation of tradition. Trans Hist Soc Ghana. 2002;6.

51. Ogundeji PA, Fasehun MA. Insights from Festivals and Carnivals. The Palgrave Handbook of African Oral Traditions and Folklore. Cham: Springer International Publishing; 2021. pp. 105–118. doi:10.1007/978-3-030-55517-7_5

5252. Ogada M, Wilfred N, And Mahmud Y. Production risk and farm technology adoption in the rain-fed semi-arid lands of Kenya. Afjare 2010. 2010.

53. Mariano MJ, Villano R, Fleming E. Factors influencing farmers' adoption of modern rice technologies and good management practices in the Philippines. Agric Syst. 2012;110: 41–53. doi:10.1016/j.agsy.2012.03.010

. Acheampong P, Osei-Adu J, Amengor NE, Sagoe R. Cocoyam value chain and benchmark study in Ghana. In: A report submitted to West African Agricultural Productivity Project (WAAPP). Project Information Document. 2014.

53. Aidoo R, I JKA, Wireko-Manu FD, Wangel A. Root and Tubers in Ghana Overview and selected Research Papers. In: College of Agriculture and natural Resources, Kwame Nkrumah University of Science and Technology (KNUST). 2019.

Formatted: Justified

Tables

Table 1. Summary Statistics of Starchy Staple Producing Farmers in Ghana (1987-2017)

Variable	Mean (SD)	Trend (%)
Household[a]		
Size (AE)	4.83 † (2.887)	0.10 [0.055]
Dependency (ratio)	1.29 (1.561)	0.16 [0.113]
Farmer[b]		
Female (dummy)	0.29 † (0.454)	-0.18* † [0.031]
Age (years)	46.84 (14.872)	0.47* [0.029]
Education (years)	5.01 † (4.820)	1.59* † ‡ [0.103]
Land owned (dummy)	0.61 † (0.489)	-0.03 † [0.033]
Land (ha)[b]		
Cassava	1.17 † (2.753)	-54.18 † [59.567]
Yam	1.57 † (3.655)	-13.88* † [2.141]
Cocoyam	1.55 (3.883)	10.54 [832.372]
Plantain	1.19 † (2.611)	-7.18 † [105.680]
Yield (Mt/ha)[b]		
Cassava	0.66 † (1.080)	2.78* † [0.220]
Yam	0.96 (1.540)	-38.47 † [3146.763]
Cocoyam	0.31 † (0.547)	0.15 [0.487]
Plantain	0.53 † (0.838)	1.57* † [0.296]
Input use[b]		
Planting material (Mt/ha)	0.06 † ‡ (0.428)	-5.68 † ‡ [20.428]
Family labor (AE)	2.74 † (1.544)	0.45* [0.054]
Hired labor (man-days/ha)	11.29 ‡ (43.200)	1.24* † ‡ [0.350]
Pesticide (Liter/ha)	7.83 † (49.505)	-36.04 † ‡ [2059.161]
Enabling environment[a]		
Credit (dummy)	0.20 † ‡ (0.399)	-0.06* ‡ [0.028]
Mechanization (dummy)	0.04 † ‡ (0.201)	-0.21* ‡ [0.015]
Extension (dummy)	0.19 (0.392)	0.75* † ‡ [0.029]

* Indicates significance at p<0.05

† and ‡ indicate statistically significant (p<0.05) differences in the variables across ecology and crop, respectively. The differences were determined via a linear regression for continuous variables and a probit model for dummies. A trend variable, and a fixed effect for ecology and crop, as well as their interactions, were included in the estimation.
[a] Household sample size; Cassava [11,459], Yam [3,439], Cocoyam [1,830], Plantain [6,166], Pooled [15,133]
[b] Farmer sample size; Cassava [11,585], Yam [3,468], Cocoyam [1,834], Plantain [6,226], Pooled [15,405]
Data Sources: Ghana Living Standards Surveys [wave 1-7] and Ghana Socioeconomic Panel Survey [wave 1-2]

Formatted Table

Formatted: Space Before: 6 pt, Don't adjust space between Latin and Asian text, Don't adjust space between Asian text and numbers

Table 2. Hypothesis Tests for Ecology- and Meta-Frontier Models for Starchy Staple Production in Ghana (1987-2017)

-	Sample size	Log-likelihood	Cobb-Douglas test	Coelli, (1995)[ab] skewness test	Gutierrez (2001)[a] LR test	Inefficiency variance [σ_u]	Total variance [$\sigma^2 = \sigma_u^2 + \sigma_v^2$]	Gamma [$\gamma = \sigma_u^2/\sigma^2$]	Inefficiency function test	Model significance
Cassava										
Guinea Savanah	801	-919	227.02***	-6.02*	11.28***	0.87 (0.092)	1.10 (0.119)	0.68*** (0.076)	37.20***	710.21***
Transitional Zone	2,064	-2,735	307.98***	-3.52*	13.96***	0.94 (0.088)	1.46 (0.118)	0.61*** (0.066)	88.27***	1194.47***
Forest Zone	7,023	-9,129	553.51***	-5.12*	19.05***	0.77 (0.061)	1.20 (0.065)	0.50*** (0.053)	109.64***	3941.36***
Coastal Savanah	1,697	-2,154	211.58***	-2.84*	6.13**	0.80 (0.114)	1.21 (0.126)	0.53*** (0.097)	403.63***	1390.95***
National	11,585	-15,347	1013.19***	-9.07*	66.22***	0.89 (0.038)	1.37 (0.048)	0.58*** (0.030)	196.70***	6319.90***
Meta-frontier	11,585	-166	16605.98***	4555.61	-	0.00 (0.036)	0.05 (0.001)	0.00 (0.001)	563.00***	91075.02***
Yam										
Sudan Savanah	222	-233	164.54***	-0.23	0.52	0.87 (0.402)	1.12 (0.480)	0.68** (0.339)	534.06***	500.60***
Guinea Savanah	1,472	-1,732	400.32***	-9.80*	34.53***	1.04 (0.064)	1.34 (0.103)	0.81*** (0.041)	25.54*	2378.83***
Transitional Zone	943	-1,288	199.15***	-4.89*	32.23***	1.35 (0.091)	2.16 (0.195)	0.84*** (0.042)	1666.17***	1328.45***
Forest Zone	831	-1,212	170.34***	-3.31*	23.13***	1.41 (0.115)	2.51 (0.251)	0.79*** (0.054)	48.43***	772.00***
National	3,468	-4,936	515.54***	-12.13*	226.81***	1.53 (0.040)	2.67 (0.104)	0.88*** (0.014)	213.99***	4669.74***
Meta-frontier	3,468	-1,994	3948.97***	-197.43*	742.33***	0.72 (0.011)	0.53 (0.015)	0.97*** (0.003)	14048.06***	38680.49***
Cocoyam										
Transitional Zone	422	-597	99.75***	-1.84	9.11***	1.38 (0.171)	2.40 (0.359)	0.79*** (0.084)	23.91**	322.93***
Forest Zone	1,412	-2,046	75.45***	-3.13*	25.43***	1.36 (0.104)	2.37 (0.212)	0.78*** (0.053)	42.48***	495.20***
National	1,834	-2,721	113.04***	-2.54*	20.51***	1.27 (0.106)	2.29 (0.195)	0.71*** (0.060)	54.73***	611.93***
Meta-frontier	1,834	-377	1587.92***	658.26	-	0.00 (0.082)	0.08 (0.003)	0.00 (0.004)	890.54***	8960.81***
Plantain										
Transitional Zone	740	-985	125.50***	-5.65*	39.00***	1.38 (0.085)	2.21 (0.195)	0.86*** (0.035)	63.15***	760.94***
Forest Zone	4,982	-6,564	357.18***	-6.48*	36.32***	0.97 (0.057)	1.46 (0.079)	0.64*** (0.042)	46.45***	3483.09***
Coastal Savanah	504	-576	109.18***	0.28	-	0.03 (0.690)	0.61 (0.046)	0.00 (0.064)	1138.60***	390.07***
National	6,226	-8,275	447.69***	-9.27*	78.14***	1.07 (0.044)	1.62 (0.068)	0.70*** (0.029)	88.04***	4366.28***
Meta-frontier	6,226	1,264	9511.74***	-1939.14*	1062.47***	0.40 (0.006)	0.19 (0.005)	0.83*** (0.008)	544.13***	113229.19***

Significance levels: * $p<0.10$, ** $p<0.05$, *** $p<0.01$
[a] Null hypothesis of no one-sided error (i.e., no inefficiency) was tested
[b] Values less than the critical value of -1.96 confirm the rejection of the null hypothesis.
Data Sources: Ghana Living Standards Surveys [wave 1-7] and Ghana Socioeconomic Panel Survey [wave 1-2]

Table 3. Elasticities for Ecology- and Meta-Frontier Models for Starchy Staple Production in Ghana (1987-2017)

-	Land elasticity	Planting material elasticity	Family labor elasticity	Hired Labor elasticity	Pesticide elasticity	Returns to scale[a]	Technical change
Cassava							
Guinea Savanah	0.31*** (0.030)	-0.13*** (0.025)	0.16*** (0.063)	0.05*** (0.008)	0.00 (0.014)	0.39*** (0.069)	0.14 (0.090)
Transitional Zone	0.36*** (0.019)	0.02 (0.018)	0.10** (0.042)	0.04*** (0.009)	0.01 (0.007)	0.53*** (0.047)	-0.07 (0.057)
Forest Zone	0.40*** (0.010)	-0.04*** (0.010)	0.14*** (0.023)	0.04*** (0.003)	-0.01* (0.004)	0.53*** (0.026)	-0.01 (0.031)
Coastal Savanah	0.41*** (0.020)	-0.05*** (0.019)	0.17*** (0.046)	0.04*** (0.007)	-0.09*** (0.018)	0.47*** (0.059)	0.01 (0.052)
National	0.39*** (0.008)	-0.03*** (0.008)	0.16*** (0.018)	0.04*** (0.003)	-0.01** (0.004)	0.55*** (0.020)	-0.04* (0.023)
Meta-frontier	0.39*** (0.002)	-0.04*** (0.002)	0.16*** (0.005)	0.04*** (0.001)	-0.01*** (0.001)	0.54*** (0.006)	-0.06*** (0.006)
Yam							
Sudan Savanah	0.44*** (0.068)	-0.08** (0.032)	0.18* (0.103)	0.01 (0.015)	-0.03 (0.020)	0.51*** (0.133)	0.30 (0.197)
Guinea Savanah	0.59*** (0.025)	-0.08*** (0.019)	0.02 (0.047)	0.02*** (0.005)	-0.02 (0.013)	0.52*** (0.056)	0.03 (0.066)
Transitional Zone	0.55*** (0.035)	0.00 (0.020)	-0.05 (0.061)	0.10*** (0.016)	0.02 (0.011)	0.61*** (0.072)	0.07 (0.108)
Forest Zone	0.32*** (0.030)	-0.08* (0.043)	0.20*** (0.076)	0.09*** (0.014)	-0.01 (0.014)	0.53*** (0.091)	0.11 (0.084)
National	0.55*** (0.019)	-0.06*** (0.013)	0.12*** (0.035)	0.05*** (0.005)	-0.02*** (0.007)	0.64*** (0.040)	0.00 (0.049)
Meta-frontier	0.51*** (0.009)	-0.07*** (0.005)	0.10*** (0.019)	0.06*** (0.002)	-0.01*** (0.003)	0.58*** (0.021)	-0.06** (0.022)
Cocoyam							
Transitional Zone	0.19*** (0.040)	-0.18** (0.083)	0.21** (0.104)	0.08*** (0.022)	-0.03 (0.034)	0.27*** (0.134)	-0.65* (0.361)
Forest Zone	0.42*** (0.041)	-0.10*** (0.037)	0.08 (0.059)	0.04*** (0.010)	0.02 (0.016)	0.45*** (0.076)	0.13 (0.136)
National	0.36*** (0.027)	-0.10*** (0.035)	0.09* (0.052)	0.05*** (0.009)	0.01 (0.014)	0.42*** (0.066)	-0.08 (0.107)
Meta-frontier	0.38*** (0.005)	-0.10*** (0.015)	0.11*** (0.015)	0.05*** (0.003)	0.01*** (0.004)	0.46*** (0.023)	-0.11*** (0.039)
Plantain							
Transitional Zone	0.44*** (0.031)	-0.04 (0.032)	0.10 (0.066)	0.06*** (0.011)	0.02 (0.015)	0.59*** (0.073)	-0.01 (0.081)
Forest Zone	0.47*** (0.014)	-0.03** (0.013)	0.14*** (0.027)	0.04*** (0.004)	0.00 (0.005)	0.62*** (0.031)	-0.01 (0.039)
Coastal Savanah	0.37*** (0.032)	0.06* (0.033)	0.12* (0.073)	0.07*** (0.015)	-0.05** (0.023)	0.58*** (0.098)	-0.02 (0.106)
National	0.47*** (0.012)	-0.02* (0.011)	0.14*** (0.025)	0.04*** (0.004)	0.00 (0.005)	0.63*** (0.028)	-0.01 (0.031)
Meta-frontier	0.47*** (0.002)	-0.03*** (0.002)	0.14*** (0.006)	0.04*** (0.001)	0.01*** (0.001)	0.63*** (0.007)	0.01* (0.008)

Significance levels: * p<0.10, ** p<0.05, ***p<0.01
[a] Null hypothesis of constant returns to scale was tested
Data Sources: Ghana Living Standards Surveys [wave 1-7] and Ghana Socioeconomic Panel Survey [wave 1-2]

Formatted: Space After: 8 pt, Line spacing: Double

Formatted: Font: 12 pt

Table 4. Ghanaian Starchy Staple Production Technology Level and Technical Efficiency Across Ecologies (1987-2017)

-	Technology gap ratio (TGR)	Technical efficiency (TE)	Meta-technical efficiency (MTE)	Technology gap	
Cassava					
Guinea Savanah	0.92*** (0.002)	0.64*** (0.007)	0.60*** (0.006)	16.31%	
Transitional Zone	0.90*** (0.001)	0.58*** (0.004)	0.54*** (0.003)	12.82%	
Forest Zone	0.87*** (0.000)	0.64*** (0.001)	0.56*** (0.001)	9.69%	
Coastal Savanah	0.84*** (0.003)	0.72*** (0.005)	0.59*** (0.003)	8.50%	
Mean	0.88	0.65	0.57	11.83%	
Yam					
Sudan Savanah	0.81*** (0.009)		0.67*** (0.018)	0.55*** (0.016)	18.72%
Guinea Savanah	0.81*** (0.003)		0.54*** (0.005)	0.43*** (0.004)	18.70%
Transitional Zone	0.70*** (0.004)		0.49*** (0.007)	0.34*** (0.005)	30.00%
Forest Zone	0.55*** (0.005)	0.51*** (0.008)	0.23*** (0.005)	45.00%	
Mean	0.72	0.55	0.39	28.11%	
Cocoyam					
Transitional Zone	0.77*** (0.009)		0.56*** (0.011)	0.38*** (0.009)	22.66%
Forest Zone	0.82*** (0.002)		0.48*** (0.005)	0.39*** (0.004)	17.76%
Mean	0.80	0.52	0.39	20.21%	
Plantain					
Transitional Zone	0.94*** (0.002)	0.50*** (0.008)	0.47*** (0.007)	25.07%	
Forest Zone	0.87*** (0.000)		0.58*** (0.002)	0.51*** (0.002)	13.01%
Coastal Savanah	0.75*** (0.005)	0.68*** (0.009)	0.50*** (0.008)	6.39%	
Mean	0.90	0.54	0.49	9.70%	
Overall mean	0.791	0.553	0.428	20.85%	

Significance levels: * p<0.10, ** p<0.05, ***p<0.01
a Null hypothesis of constant returns to scale was tested
Data Sources: Ghana Living Standards Surveys [wave 1-7] and Ghana Socioeconomic Panel Survey [wave 1-2]

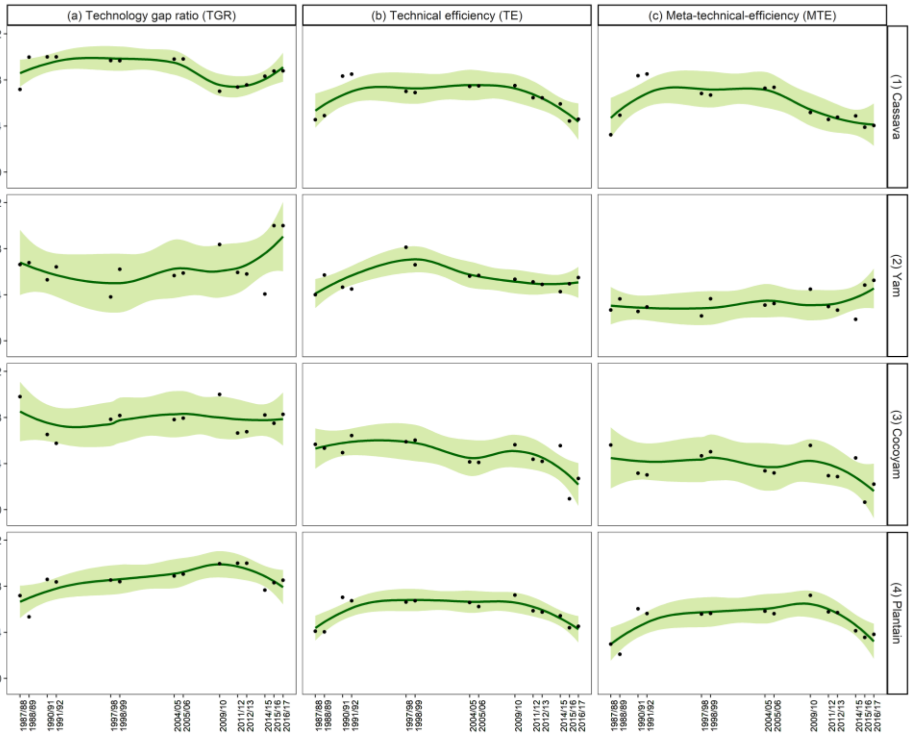

Figure 1. Ghanaian Starchy Staple Production Technology Level and Technical Efficiency Parameters Across Seasons (1987-2017)

Notes: Farmer-level scores were first estimated via a Meta–stochastic–frontier (MSF) analysis applied separately to 9 population–based surveys that represent 30 years of farmer-level data collection in Ghana the surveys used included Ghana Living Standards Surveys [wave 1–7] and Ghana Socioeconomic Panel Survey [wave 1–2]. The farmer-level elasticities were subsequently averaged across seasons via a regression framework to account for controls. Each point on a sub–panel represents the mean of the estimates. Given the seasonal means, the fitted line was done locally using neighborhood points, weighted by distance. The size of the neighborhood was set to 75% of the points with a tri–cubic weighting. The gray region is the 95% confidence interval of the fitted line.

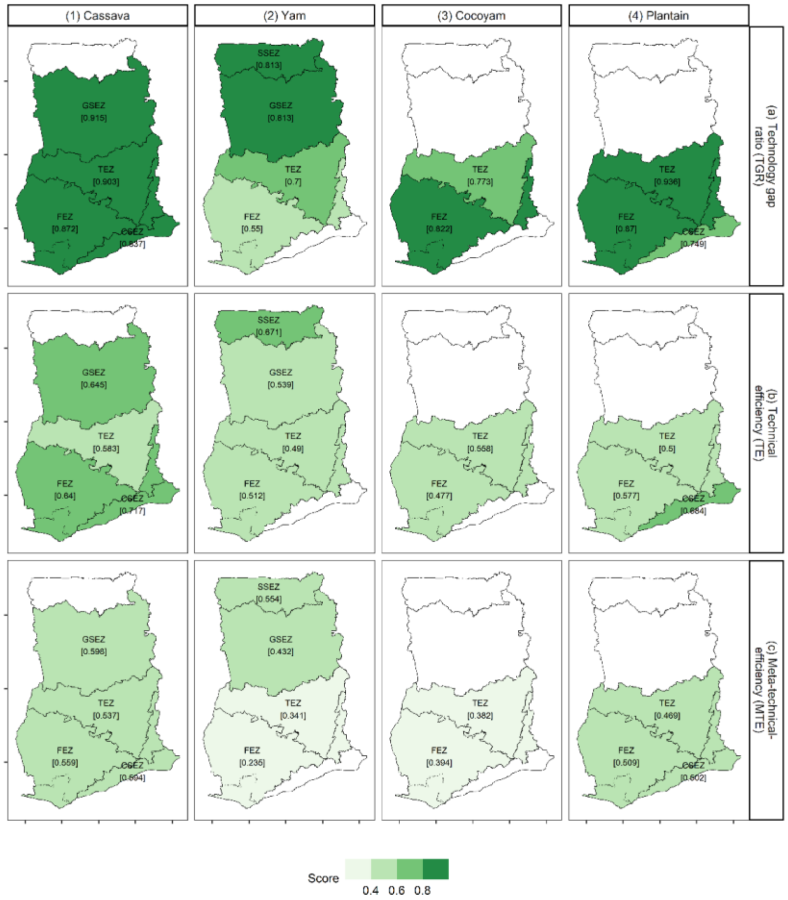

Sudan Savanna=SSEZ; Guinea Savanna=GSEZ; Transitional Zone= TEZ;Forest Zone=FEZ

~~**Figure 2.** Ghanaian Starchy Staple Production Technology Level and Technical Efficiency Across Ecologies (1987-2017)~~

~~**Notes:** Farmer-level scores were estimated via a Meta–stochastic–frontier (MSF) analysis applied separately to 10 population–based surveys that represent 30 years of farmer-level data collection in Ghana the surveys used included Ghana Living Standards Surveys [wave 1–7] and Ghana Socioeconomic Panel Survey [wave 1–2].~~

Appendix S: supplementary materials

Table S1. Ecology Population, Climatic, and Soil Characteristics in Ghana.

Variable	Sudan Savanah	Guinea Savanna	Transitional Zone	Semi-Deciduous Forest	Rain Forest	Coastal Savanna
Area (1000 km^2)	50.12	25.92	14.21	16.66	18.48	10.81
Pop. Den. (N/km^2)	59.09	162.76	140.14	89.61	37.99	115.97
Agric Pop. (%)	55.41	38.73	55.00	45.59	77.09	31.63
Rural Pop. (%)	48.50	38.39	56.68	48.42	79.46	30.48
Soil pH	4.28-7.40	4.13-7.44	4.10-7.66	4.21-7.34	4.40-6.70	4.29-8.28
Soil Org. matter (%)	0.52-6.74	0.06-7.63	0.00-11.18	0.05-11.31	0.19-13.83	0.00-4.84
Soil N (%)	0.00-0.11	0.00-0.18	0.00-0.39	0.00-0.39	0.00-0.37	0.00-0.25
Soil P (mg/kg soil)	0.00-4.54	0.00-6.35	0.00-3.41	0.00-3.92	0.00-0.26	0.00-7.51
Soil CEC	0.00-6.41	0.00-7.86	0.00-9.55	0.00-6.54	0.00-1.21	0.00-6.96
10-year avg rain						
Mean [mm/year]	943.29	1,128.52	1,244.52	1,310.65	1,443.30	1,064.89
CV [%]	17.55	10.54	9.85	9.57	13.36	10.93

All values are calculated as weighted averages (by area) using data retrieved from published reports Agriculture in Ghana: Facts and figures (2016)

Table S2. Ecology Level **Starchy Staple** Production Statistics in Ghana for 2010/2015

Ecology/Crop	Cultivating Household [a] Count (1000)	Cultivating Household [a] Share (%)	Cultivating Household [a] Size (count)	Farm size (ha) [a]	Area Share (%) [b]	Production Share (%) [b]	Yield ratio [b]
Cassava							
Semi-Deciduous Forest	1647.48 (1269.22)	59.47 (6.27)	5.06 (0.84)	0.39 (0.22)	46.54 (2.26)	49.06 (3.00)	1.00 (0.00)
Transitional Zone	558.77 (490.46)	18.48 (4.09)	5.56 (0.81)	0.81 (0.59)	26.00 (1.36)	27.42 (2.27)	1.00 (0.09)
Coastal Savanna	384.44 (396.35)	10.58 (3.16)	5.05 (1.03)	0.83 (0.60)	13.61 (0.62)	11.60 (0.67)	0.81 (0.06)
Guinea Savanna	273.88 (323.54)	6.17 (4.42)	7.81 (1.56)	2.35 (2.14)	10.77 (3.71)	10.24 (3.86)	0.92 (0.25)
Rain Forest	140.94 (105.37)	5.18 (0.76)	5.31 (0.96)	0.25 (0.15)	2.58 (0.28)	1.51 (0.22)	0.56 (0.05)
Sudan Savanah	3.07 (2.59)	0.14 (0.13)	4.57 (0.91)	3.73 (4.10)	0.58 (0.38)	0.19 (0.05)	0.35 (0.10)
Yam							
Transitional Zone	298.94 (252.41)	34.45 (8.29)	5.64 (0.86)	1.06 (0.62)	50.73 (9.70)	52.51 (10.28)	1.46 (0.04)
Guinea Savanna	441.81 (451.37)	38.86 (9.20)	7.78 (1.68)	0.98 (0.96)	31.30 (11.57)	33.77 (12.01)	1.54 (0.24)
Semi-Deciduous Forest	164.93 (113.55)	21.25 (5.56)	5.04 (0.29)	0.39 (0.22)	12.03 (2.48)	8.55 (1.81)	1.00 (0.00)
Sudan Savanah	39.68 (44.83)	3.00 (1.36)	6.67 (0.78)	2.43 (2.30)	5.27 (0.75)	4.88 (0.25)	1.32 (0.18)
Coastal Savanna	27.83 (31.93)	2.01 (1.14)	3.86 (1.57)	0.38 (0.34)	0.57 (0.15)	0.26 (0.11)	0.62 (0.08)
Rain Forest	3.85 (3.12)	0.44 (0.31)	5.60 (2.15)	0.23 (0.19)	0.10 (0.02)	0.04 (0.01)	0.50 (0.11)
Cocoyam							
Semi-Deciduous Forest	507.52 (417.81)	76.76 (3.65)	5.10 (0.95)	0.64 (0.43)	81.79 (9.17)	83.96 (1.72)	1.00 (0.00)
Transitional Zone	96.49 (75.16)	16.54 (5.19)	5.50 (1.38)	0.45 (0.27)	12.22 (1.78)	13.99 (1.69)	1.11 (0.05)
Coastal Savanna	31.59 (35.94)	3.17 (2.25)	3.99 (1.60)	0.73 (0.83)	1.38 (0.63)	1.21 (0.75)	0.82 (0.16)
Rain Forest	31.89 (35.58)	3.45 (1.81)	5.53 (1.91)	0.15 (0.12)	0.62 (0.31)	0.55 (0.41)	0.82 (0.14)
Guinea Savanna	0.33 (0.01)	0.14 (0.03)	7.40 (3.58)	55.35 (116.28)	6.39 (13.03)	0.47 (0.05)	0.69 (0.39)
Plantain							
Semi-Deciduous Forest	1259.52 (992.94)	78.26 (3.50)	5.01 (0.71)	0.36 (0.21)	80.01 (0.73)	82.38 (0.82)	1.00 (0.00)
Transitional Zone	175.63 (144.96)	10.84 (2.54)	5.12 (0.90)	0.59 (0.38)	16.85 (0.42)	15.29 (0.67)	0.88 (0.03)
Coastal Savanna	157.98 (173.84)	7.02 (3.70)	4.85 (0.85)	0.19 (0.15)	2.40 (0.53)	1.74 (0.25)	0.71 (0.08)
Rain Forest	69.62 (62.34)	3.87 (0.86)	5.40 (1.43)	0.08 (0.06)	0.72 (0.05)	0.57 (0.05)	0.77 (0.05)

[a] Calculated by authors from data retrieved from GLSS Waves 5-7

[b] Calculated by authors from data retrieved from FAO CountryStat

Table S3. Stochastic Meta-Frontier Results for Cassava Production in Ghana (1987-2017)

-	Guinea Savanah	Transitional Zone	Forest Zone	Coastal Savanah	National	Meta-frontier
**Production function**	-	-	-	-	-	-
Land(ln[ha]) {lnI1}	-0.22** (0.099)	0.23*** (0.062)	0.16*** (0.034)	0.16** (0.075)	0.17*** (0.027)	0.16*** (0.007)
Seed (ln[Mt]) {lnI2}	0.18*** (0.061)	-0.07* (0.037)	0.04** (0.019)	0.07** (0.035)	0.03** (0.015)	0.04*** (0.004)
Hired labor(ln[days]) {lnI3}	0.13 (0.189)	0.23* (0.134)	0.06 (0.073)	0.15 (0.172)	0.09 (0.059)	0.13*** (0.015)
Family labor(ln[days]) {lnI4}	0.17*** (0.044)	0.10*** (0.032)	0.10*** (0.015)	0.08*** (0.030)	0.10*** (0.012)	0.10*** (0.003)
Pesticide (ln[liter]) {lnI5}	0.10** (0.049)	0.12*** (0.031)	0.08*** (0.013)	0.16*** (0.033)	0.08*** (0.011)	0.09*** (0.003)
Trend {I6}	-0.17 (0.198)	-0.17 (0.162)	0.20*** (0.068)	-0.20* (0.118)	0.03 (0.053)	0.01 (0.013)
P5·lnI1·lnI1	-0.04 (0.038)	-0.07*** (0.023)	-0.04*** (0.012)	-0.02 (0.026)	-0.05*** (0.009)	-0.04*** (0.003)
P5·lnI2·lnI2	0.05*** (0.010)	0.00 (0.006)	0.02*** (0.003)	0.03*** (0.007)	0.02*** (0.003)	0.02*** (0.001)
P5·lnI3·lnI3	0.13 (0.171)	0.11 (0.121)	-0.06 (0.066)	-0.05 (0.136)	-0.02 (0.050)	-0.01 (0.015)
P5·lnI4·lnI4	0.04*** (0.007)	0.02*** (0.005)	0.03*** (0.003)	0.02*** (0.005)	0.03*** (0.002)	0.03*** (0.001)
P5·lnI5·lnI5	0.02*** (0.009)	0.03*** (0.006)	0.02*** (0.003)	0.04*** (0.008)	0.02*** (0.002)	0.02*** (0.001)
P5·I6·I6	0.02*** (0.009)	0.03*** (0.006)	0.02*** (0.003)	0.04*** (0.008)	0.02*** (0.002)	0.02*** (0.001)
lnI1.lnI2	0.00 (0.008)	0.01** (0.005)	0.00 (0.003)	-0.01** (0.005)	0.00 (0.002)	0.00 (0.001)
lnI1.lnI3	0.09* (0.048)	-0.01 (0.035)	-0.02 (0.018)	-0.04 (0.038)	-0.01 (0.014)	-0.01** (0.004)
lnI1.lnI4	-0.02** (0.007)	-0.01 (0.005)	0.00 (0.002)	0.01** (0.004)	0.00* (0.002)	0.00*** (0.001)
lnI1.lnI5	-0.01 (0.007)	0.01* (0.005)	-0.01*** (0.002)	0.00 (0.005)	0.00 (0.002)	0.00*** (0.001)
lnI1.I6	0.02*** (0.003)	0.01*** (0.002)	0.01*** (0.001)	0.01*** (0.002)	0.01*** (0.001)	0.01*** (0.000)
lnI2.lnI3	0.00 (0.015)	0.00 (0.011)	-0.01 (0.006)	0.01 (0.013)	0.00 (0.005)	0.00* (0.001)
lnI2.lnI4	0.00 (0.002)	0.00** (0.001)	-0.01*** (0.001)	-0.01*** (0.001)	0.00*** (0.001)	-0.01*** (0.000)
lnI2.lnI5	0.00 (0.002)	0.00** (0.002)	0.00 (0.001)	0.00 (0.002)	0.00** (0.001)	0.00*** (0.000)
lnI2.I6	0.00 (0.001)	0.01*** (0.001)	0.00*** (0.000)	0.00*** (0.001)	0.00*** (0.000)	0.00*** (0.000)
lnI3.lnI4	-0.03** (0.014)	0.03*** (0.010)	0.00 (0.005)	0.00 (0.011)	0.00 (0.004)	0.00 (0.001)
lnI3.lnI5	0.00 (0.015)	-0.01 (0.010)	0.00 (0.005)	0.00 (0.013)	0.00 (0.004)	0.00 (0.001)
lnI3.I6	-0.01 (0.006)	0.00 (0.004)	0.00 (0.002)	0.00 (0.005)	0.00 (0.002)	0.00 (0.000)
lnI4.lnI5	0.00 (0.002)	0.00 (0.001)	0.00 (0.001)	0.00 (0.001)	0.00 (0.001)	0.00*** (0.000)
lnI4.I6	0.00 (0.001)	0.00** (0.001)	0.00 (0.000)	0.00 (0.001)	0.00 (0.000)	0.00*** (0.000)
lnI5.I6	0.00* (0.001)	0.00 (0.001)	0.00 (0.000)	0.00* (0.001)	0.00* (0.000)	0.00*** (0.000)
Constant	-1.92 (2.991)	2.20 (1.939)	-0.94 (0.919)	0.13 (1.568)	0.80 (0.694)	1.36*** (0.184)
Source (base=GLSS7)						
GLSS1	2.14 (2.913)	-1.63 (1.961)	1.11 (0.917)	-0.36 (1.564)	-0.47 (0.697)	-0.75*** (0.186)
GLSS2	2.19 (2.753)	-2.41 (1.846)	0.75 (0.876)	0.21 (1.498)	-0.74 (0.662)	-1.32*** (0.177)
GLSS3	2.37 (2.348)	-1.91 (1.511)	0.07 (0.782)	0.14 (1.301)	-0.98* (0.575)	-1.58*** (0.158)
GLSS4	3.43** (1.719)	-0.86 (1.084)	-0.44 (0.634)	0.78 (1.004)	-0.77* (0.451)	-1.24*** (0.129)
GLSS5	3.43*** (1.136)	0.08 (0.793)	-0.85* (0.464)	1.25* (0.743)	-0.58* (0.333)	-0.91*** (0.095)
GLSS6	2.11*** (0.496)	0.54 (0.365)	-0.41* (0.215)	-0.22 (0.359)	-0.20 (0.156)	-0.09* (0.052)
GSPS1	2.12*** (0.759)	0.57 (0.561)	-0.91*** (0.334)	1.47*** (0.544)	-0.38 (0.240)	-0.30*** (0.069)
GSPS2	0.62 (0.382)	0.69*** (0.244)	0.10 (0.142)	-0.23 (0.294)	0.07 (0.105)	0.16*** (0.031)
**Uncertainty function**	-	-	-	-	-	-
Mean	-0.99*** (0.155)	-0.65*** (0.077)	-0.52*** (0.039)	-0.60*** (0.055)	-0.55*** (0.035)	-3.12*** (0.050)
Variance	0.61 (0.047)	0.72 (0.028)	0.77 (0.015)	0.74 (0.020)	0.76 (0.013)	0.21 (0.005)

Significance levels: * p<0.10, ** p<0.05, ***p<0.01; Data Sources: Ghana Living Standards Surveys [wave 1-7] and Ghana Socioeconomic Panel Survey [wave 1-2]

Table S4. Stochastic Meta-Frontier Results for Yam Production in Ghana (1987-2017)

-	Sudan Savanah	Guinea Savanah	Transitional Zone	Forest Zone	National	Meta-frontier
**Production function**	-	-	-	-	-	-
Land(ln[ha]) {lnI1}	0.30 (0.282)	-0.10 (0.097)	0.27*** (0.094)	0.08 (0.127)	0.18*** (0.057)	0.06** (0.028)
Seed (ln[Mt]) {lnI2}	0.32** (0.127)	0.21*** (0.041)	0.06 (0.051)	0.20** (0.079)	0.20*** (0.029)	0.22*** (0.011)
Hired labor(ln[days]) {lnI3}	1.71*** (0.450)	-0.10 (0.159)	-0.12 (0.221)	0.41 (0.331)	0.09 (0.120)	0.17** (0.073)
Family labor(ln[days]) {lnI4}	0.21** (0.103)	0.12*** (0.029)	0.19*** (0.048)	0.16*** (0.061)	0.18*** (0.024)	0.18*** (0.011)
Pesticide (ln[liter]) {lnI5}	-0.22* (0.119)	0.21*** (0.044)	0.13*** (0.044)	0.23*** (0.053)	0.17*** (0.026)	0.15*** (0.011)
Trend {I6}	0.49 (0.627)	-0.62*** (0.168)	0.05 (0.383)	0.99*** (0.182)	-0.08 (0.122)	-0.36*** (0.062)
P5·lnI1·lnI1	0.00 (0.068)	0.01 (0.025)	-0.10*** (0.030)	-0.09*** (0.032)	-0.04** (0.017)	0.00 (0.009)
P5·lnI2·lnI2	0.09*** (0.015)	0.06*** (0.008)	0.03*** (0.008)	0.04** (0.016)	0.05*** (0.005)	0.05*** (0.002)
P5·lnI3·lnI3	0.12 (0.304)	0.10 (0.137)	-0.12 (0.159)	-0.10 (0.216)	-0.05 (0.094)	-0.05 (0.038)
P5·lnI4·lnI4	0.05*** (0.017)	0.03*** (0.005)	0.04*** (0.007)	0.05*** (0.008)	0.04*** (0.004)	0.05*** (0.002)
P5·lnI5·lnI5	0.02 (0.019)	0.04*** (0.007)	0.02** (0.010)	0.03*** (0.010)	0.04*** (0.005)	0.04*** (0.002)
P5·I6·I6	0.02 (0.019)	0.04*** (0.007)	0.02** (0.010)	0.03*** (0.010)	0.04*** (0.005)	0.04*** (0.002)
lnI1.lnI2	-0.01 (0.016)	-0.02*** (0.007)	0.00 (0.007)	0.00 (0.011)	-0.02*** (0.004)	-0.02*** (0.002)
lnI1.lnI3	0.13 (0.101)	0.07 (0.044)	0.02 (0.047)	0.01 (0.055)	0.05* (0.028)	0.07*** (0.013)
lnI1.lnI4	-0.02 (0.018)	0.00 (0.006)	0.00 (0.007)	0.01 (0.009)	-0.01 (0.004)	-0.01*** (0.002)
lnI1.lnI5	-0.01 (0.016)	-0.02*** (0.007)	-0.01 (0.006)	-0.01 (0.008)	-0.01 (0.004)	-0.01*** (0.002)
lnI1.I6	-0.01 (0.009)	0.02*** (0.003)	0.01*** (0.003)	0.01** (0.003)	0.01*** (0.002)	0.01*** (0.001)
lnI2.lnI3	0.06** (0.027)	0.00 (0.011)	0.00 (0.015)	0.00 (0.027)	0.00 (0.008)	-0.01** (0.003)
lnI2.lnI4	0.00 (0.004)	0.00*** (0.001)	0.00 (0.002)	-0.01 (0.005)	0.00* (0.001)	0.00*** (0.000)
lnI2.lnI5	-0.01 (0.006)	0.00 (0.002)	0.00 (0.002)	0.01* (0.003)	0.00 (0.001)	0.00 (0.000)
lnI2.I6	0.00 (0.004)	0.00*** (0.001)	0.01*** (0.001)	0.00 (0.002)	0.00*** (0.001)	0.00*** (0.000)
lnI3.lnI4	-0.01 (0.028)	-0.01 (0.011)	-0.01 (0.016)	-0.01 (0.020)	-0.02** (0.008)	-0.01*** (0.003)
lnI3.lnI5	0.00 (0.030)	-0.02 (0.013)	0.01 (0.015)	0.01 (0.019)	-0.01 (0.009)	-0.01* (0.004)
lnI3.I6	-0.05*** (0.013)	0.00 (0.005)	0.00 (0.008)	-0.01 (0.008)	0.00 (0.004)	-0.01*** (0.003)
lnI4.lnI5	-0.01 (0.004)	0.00 (0.001)	0.00 (0.002)	0.00 (0.003)	0.00 (0.001)	0.00*** (0.000)
lnI4.I6	0.00 (0.002)	0.00 (0.001)	0.00 (0.001)	0.00*** (0.001)	0.00 (0.001)	0.00*** (0.000)
lnI5.I6	0.01*** (0.003)	0.00 (0.001)	0.00 (0.001)	0.00 (0.001)	0.00 (0.001)	0.00** (0.000)
Constant	-11.98 (9.086)	4.49* (2.390)	-1.90 (4.634)	-6.20** (2.491)	0.63 (1.699)	3.67*** (0.860)
Source (base=GLSS7)						
GLSS1	11.33 (9.002)	-3.96* (2.311)	2.87 (4.613)	7.42*** (2.515)	0.38 (1.685)	-2.56*** (0.881)
GLSS2	9.78 (8.294)	-3.68* (2.172)	1.41 (4.289)	6.37*** (2.387)	0.03 (1.587)	-2.64*** (0.803)
GLSS3	9.18 (7.044)	-1.65 (1.873)	2.07 (3.371)	3.10 (2.120)	0.36 (1.356)	-1.51** (0.662)
GLSS4	5.49 (4.128)	0.89 (1.273)	2.66 (2.007)	-1.46 (1.644)	1.01 (0.902)	0.79** (0.402)
GLSS5	4.41** (2.203)	2.44*** (0.828)	1.13 (1.322)	-2.94** (1.190)	0.80 (0.621)	1.18*** (0.256)
GLSS6	2.22*** (0.736)	1.80*** (0.356)	0.91 (0.663)	-2.46*** (0.572)	0.76*** (0.271)	1.39*** (0.100)
GSPS1	2.92** (1.191)	2.15*** (0.550)	1.23 (0.945)	-2.29*** (0.831)	0.75* (0.410)	1.12*** (0.167)
GSPS2	1.48*** (0.393)	1.92*** (0.239)	-0.53 (0.496)	-2.12*** (0.417)	1.29*** (0.186)	1.94*** (0.057)
**Uncertainty function**	-	-	-	-	-	-
Mean	-1.33*** (0.223)	-1.27*** (0.160)	-0.84*** (0.217)	-0.57*** (0.139)	-1.05*** (0.091)	-2.68*** (0.075)
Variance	0.51 (0.057)	0.53 (0.042)	0.66 (0.071)	0.75 (0.053)	0.59 (0.027)	0.26 (0.010)

Significance levels: * p<0.10, ** p<0.05, ***p<0.01; Data Sources: Ghana Living Standards Surveys [wave 1-7] and Ghana Socioeconomic Panel Survey [wave 1-2]

Table S5. Stochastic Meta-Frontier Results for Cocoyam Production in Ghana (1987-2017)

-	Transitional Zone	Forest Zone	National	Meta-frontier
**Production function**	-	-	-	-
Land(ln[ha]) {lnI1}	0.00 (0.097)	0.14 (0.092)	0.11 (0.071)	0.13*** (0.029)
Seed (ln[Mt]) {lnI2}	0.21** (0.107)	0.20*** (0.048)	0.18*** (0.046)	0.19*** (0.023)
Hired labor(ln[days]) {lnI3}	0.19 (0.322)	0.02 (0.202)	-0.04 (0.192)	-0.02 (0.071)
Family labor(ln[days]) {lnI4}	0.16** (0.080)	0.12*** (0.038)	0.13*** (0.035)	0.13*** (0.017)
Pesticide (ln[liter]) {lnI5}	0.11 (0.079)	0.01 (0.042)	0.02 (0.037)	0.02 (0.014)
Trend {I6}	-0.87 (0.865)	0.11 (0.223)	-0.09 (0.189)	-0.06 (0.067)
P5·lnI1·lnI1	-0.19*** (0.046)	-0.13*** (0.029)	-0.14*** (0.025)	-0.14*** (0.008)
P5·lnI2·lnI2	0.07*** (0.023)	0.04*** (0.010)	0.04*** (0.010)	0.04*** (0.006)
P5·lnI3·lnI3	0.27 (0.258)	-0.05 (0.168)	0.01 (0.146)	-0.06 (0.040)
P5·lnI4·lnI4	0.04*** (0.011)	0.02*** (0.006)	0.03*** (0.005)	0.02*** (0.001)
P5·lnI5·lnI5	0.03 (0.017)	-0.01 (0.008)	0.00 (0.007)	0.00 (0.002)
P5·I6·I6	0.03 (0.017)	-0.01 (0.008)	0.00 (0.007)	0.00 (0.002)
lnI1.lnI2	-0.01 (0.009)	-0.01 (0.008)	-0.01 (0.007)	-0.01** (0.004)
lnI1.lnI3	-0.01 (0.067)	0.03 (0.040)	0.01 (0.037)	0.02 (0.010)
lnI1.lnI4	0.02* (0.010)	0.01 (0.005)	0.01 (0.005)	0.00*** (0.002)
lnI1.lnI5	0.00 (0.010)	0.00 (0.006)	0.00 (0.005)	0.00 (0.001)
lnI1.I6	0.00 (0.005)	0.01** (0.002)	0.00* (0.002)	0.00*** (0.000)
lnI2.lnI3	0.00 (0.029)	0.00 (0.019)	-0.01 (0.018)	-0.02** (0.009)
lnI2.lnI4	-0.01** (0.005)	0.00 (0.003)	-0.01** (0.003)	-0.01*** (0.002)
lnI2.lnI5	0.00 (0.003)	0.00 (0.002)	0.00 (0.002)	0.00 (0.001)
lnI2.I6	0.01* (0.003)	0.00 (0.001)	0.00 (0.001)	0.00 (0.001)
lnI3.lnI4	0.04* (0.024)	0.00 (0.014)	0.01 (0.012)	0.02*** (0.004)
lnI3.lnI5	0.00 (0.027)	0.00 (0.015)	0.00 (0.013)	0.00 (0.004)
lnI3.I6	0.01 (0.010)	0.00 (0.005)	0.00 (0.005)	0.00*** (0.001)
lnI4.lnI5	0.00 (0.004)	0.00 (0.002)	0.00 (0.002)	0.00*** (0.001)
lnI4.I6	0.00*** (0.001)	0.00 (0.001)	0.00** (0.001)	0.00*** (0.000)
lnI5.I6	0.00 (0.002)	0.00 (0.001)	0.00 (0.001)	0.00*** (0.000)
Constant	18.24* (9.490)	-2.33 (3.801)	3.53 (3.063)	4.45*** (1.083)
Source (base=GLSS7)				
GLSS1	-18.15* (9.547)	3.40 (3.814)	-2.70 (3.053)	-3.46*** (1.094)
GLSS2	-17.32** (8.795)	2.84 (3.755)	-2.88 (2.990)	-3.79*** (1.085)
GLSS3	-16.01** (6.824)	3.02 (3.629)	-2.67 (2.887)	-2.92*** (1.065)
GLSS4	-10.38** (4.212)	2.33 (3.289)	-2.21 (2.576)	-2.40** (0.977)
GLSS5	-4.93* (2.975)	1.24 (2.540)	-1.10 (2.020)	-1.59** (0.743)
GLSS6	-0.94 (1.651)	0.24 (1.141)	-0.41 (0.932)	-0.44 (0.340)
GSPS1	-2.93 (2.241)	0.50 (1.727)	-1.04 (1.400)	-1.52*** (0.545)
GSPS2	-1.06 (1.158)	0.02 (0.660)	-0.56 (0.557)	-0.51*** (0.196)
**Uncertainty function**	-	-	-	-
Mean	-0.58*** (0.196)	-0.66*** (0.241)	-0.37*** (0.123)	-2.94*** (0.110)
Variance	0.75 (0.073)	0.72 (0.087)	0.83 (0.051)	0.23 (0.013)

Significance levels: * p<0.10, ** p<0.05, ***p<0.01; Data Sources: Ghana Living Standards Surveys [wave 1-7] and Ghana Socioeconomic Panel Survey [wave 1-2]

Table S6. Stochastic Meta-Frontier Results for Plantain Production in Ghana (1987-2017)

-	Transitional Zone	Forest Zone	Coastal Savanah	National	Meta-frontier
**Production function**	-	-	-	-	-
Land(ln[ha]) {lnI1}	0.35*** (0.106)	0.31*** (0.044)	0.24* (0.138)	0.32*** (0.038)	0.33*** (0.009)
Seed (ln[Mt]) {lnI2}	0.04 (0.062)	0.03 (0.023)	-0.06 (0.057)	0.03 (0.020)	0.03*** (0.005)
Hired labor(ln[days]) {lnI3}	0.02 (0.206)	0.07 (0.100)	-0.17 (0.292)	0.07 (0.088)	0.03 (0.025)
Family labor(ln[days]) {lnI4}	0.13*** (0.048)	0.13*** (0.019)	0.11* (0.066)	0.13*** (0.017)	0.13*** (0.004)
Pesticide (ln[liter]) {lnI5}	0.06 (0.055)	0.05*** (0.017)	0.16*** (0.049)	0.06*** (0.016)	0.06*** (0.004)
Trend {I6}	0.40* (0.215)	0.05 (0.079)	-0.13 (0.255)	0.09 (0.070)	0.17*** (0.021)
P5·lnI1·lnI1	-0.04 (0.033)	-0.01 (0.013)	-0.09** (0.038)	-0.01 (0.012)	-0.01*** (0.002)
P5·lnI2·lnI2	0.02** (0.009)	0.02*** (0.004)	-0.02 (0.012)	0.02*** (0.003)	0.02*** (0.001)
P5·lnI3·lnI3	0.11 (0.166)	-0.11 (0.084)	-0.23 (0.230)	-0.07 (0.072)	-0.04** (0.019)
P5·lnI4·lnI4	0.03*** (0.008)	0.02*** (0.003)	0.04*** (0.009)	0.02*** (0.003)	0.03*** (0.001)
P5·lnI5·lnI5	0.02** (0.010)	0.01*** (0.003)	0.04*** (0.011)	0.02*** (0.003)	0.01*** (0.001)
P5·I6·I6	0.02** (0.010)	0.01*** (0.003)	0.04*** (0.011)	0.02*** (0.003)	0.01*** (0.001)
lnI1.lnI2	0.01 (0.008)	0.01* (0.004)	0.00 (0.010)	0.01** (0.003)	0.01*** (0.001)
lnI1.lnI3	0.11** (0.050)	-0.01 (0.021)	0.00 (0.057)	0.01 (0.018)	0.01*** (0.004)
lnI1.lnI4	0.00 (0.008)	0.00 (0.003)	-0.02** (0.009)	0.00 (0.003)	0.00*** (0.001)
lnI1.lnI5	0.00 (0.008)	-0.01*** (0.003)	0.02** (0.009)	-0.01** (0.003)	-0.01*** (0.001)
lnI1.I6	0.01** (0.003)	0.01*** (0.001)	0.01 (0.004)	0.01*** (0.001)	0.01*** (0.000)
lnI2.lnI3	0.01 (0.017)	-0.01 (0.008)	-0.01 (0.023)	-0.01 (0.007)	-0.01*** (0.002)
lnI2.lnI4	-0.01** (0.002)	0.00** (0.001)	0.00 (0.005)	0.00*** (0.001)	0.00*** (0.000)
lnI2.lnI5	0.00* (0.003)	0.00 (0.001)	0.00 (0.003)	0.00* (0.001)	0.00*** (0.000)
lnI2.I6	0.00** (0.002)	0.00*** (0.001)	0.00* (0.002)	0.00*** (0.000)	0.00*** (0.000)
lnI3.lnI4	0.02 (0.016)	0.00 (0.007)	0.02 (0.019)	0.01 (0.006)	0.01*** (0.001)
lnI3.lnI5	-0.01 (0.015)	0.01 (0.007)	-0.02 (0.018)	0.01 (0.006)	0.00** (0.002)
lnI3.I6	0.02** (0.006)	0.00 (0.003)	0.01 (0.007)	0.00 (0.003)	0.01*** (0.001)
lnI4.lnI5	0.00 (0.002)	0.00 (0.001)	0.00 (0.003)	0.00 (0.001)	0.00 (0.000)
lnI4.I6	0.00 (0.001)	0.00 (0.000)	0.00* (0.001)	0.00 (0.000)	0.00*** (0.000)
lnI5.I6	0.00 (0.001)	0.00** (0.000)	0.00 (0.001)	0.00** (0.000)	0.00*** (0.000)
Constant	-1.36 (2.633)	-0.02 (1.119)	0.85 (3.498)	-0.20 (0.943)	-1.04*** (0.257)
Source (base=GLSS7)					
GLSS1	2.31 (2.633)	0.44 (1.102)	-0.09 (3.486)	0.83 (0.935)	1.98*** (0.265)
GLSS2	2.05 (2.476)	0.36 (1.057)	-1.04 (3.263)	0.65 (0.892)	2.03*** (0.246)
GLSS3	0.59 (2.087)	-0.04 (0.944)	-0.16 (2.715)	0.10 (0.789)	0.84*** (0.225)
GLSS4	-1.38 (1.514)	-0.05 (0.752)	-0.20 (1.785)	-0.20 (0.615)	0.10 (0.167)
GLSS5	-1.86* (1.104)	0.08 (0.556)	0.30 (1.110)	-0.13 (0.447)	-0.17 (0.112)
GLSS6	-0.94* (0.527)	-0.06 (0.257)	0.79 (0.512)	-0.11 (0.209)	-0.32*** (0.059)
GSPS1	-1.37* (0.779)	-0.09 (0.401)	0.68 (0.771)	-0.14 (0.323)	-0.39*** (0.090)
GSPS2	-0.06 (0.362)	0.01 (0.176)	-0.12 (0.408)	-0.01 (0.144)	0.07 (0.057)
**Uncertainty function**	-	-	-	-	-
Mean	-1.15*** (0.148)	-0.62*** (0.112)	-0.90*** (0.145)	-0.69*** (0.083)	-3.76*** (0.078)
Variance	0.56 (0.042)	0.73 (0.041)	0.64 (0.046)	0.71 (0.029)	0.15 (0.006)

Significance levels: * p<0.10, ** p<0.05, ***p<0.01; Data Sources: Ghana Living Standards Surveys [wave 1-7] and Ghana Socioeconomic Panel Survey [wave 1-2]

Table S7. Determinants of Starchy Staple Technical Inefficiency/ Technology Gap in Ghana (1987-2017)

-	Female (dummy)	Age (years)		Education (years)		Land owned (dummy)	Extension (dummy)	Credit (dummy)	Mechanization (dummy)
		Level	Squared	Level	Squared				
Cassava									
Guinea Savanah	0.49* (0.263)	0.53* (0.315)	-0.78 (0.892)	0.13 (0.244)	0.01 (0.028)	-0.58*** (0.206)	0.26 (0.215)	0.79*** (0.236)	0.32 (0.451)
Transitional Zone	0.37*** (0.116)	0.12 (0.184)	1.05*** (0.371)	0.10 (0.129)	0.01 (0.015)	0.03 (0.114)	-0.26* (0.135)	0.41*** (0.151)	-
Forest Zone	0.16** (0.077)	0.24* (0.125)	0.39 (0.313)	0.05 (0.073)	0.00 (0.008)	-0.12* (0.069)	-0.10 (0.082)	-0.02 (0.106)	0.37 (0.329)
Coastal Savanah	0.35** (0.159)	0.08 (0.223)	0.05 (0.507)	-0.08 (0.196)	-0.01 (0.023)	0.21 (0.157)	-	0.41* (0.213)	0.01 (0.431)
National	0.28*** (0.054)	0.28*** (0.085)	0.39* (0.206)	0.09 (0.055)	0.01 (0.006)	-0.11** (0.050)	-0.06 (0.060)	0.19*** (0.069)	0.12 (0.164)
Meta-frontier	0.11 (0.070)	0.03 (0.088)	-0.12 (0.205)	0.01 (0.062)	0.00 (0.007)	-0.02 (0.058)	-0.14 (0.088)	0.15** (0.066)	0.47*** (0.135)
Yam									
Sudan Savanah	-1.96* (1.138)	-0.05 (0.559)	1.28 (1.284)	1.48* (0.852)	0.18* (0.103)	-2.20** (1.118)	-0.38 (1.012)	-0.19 (0.412)	-1.92** (0.899)
Guinea Savanah	0.33* (0.196)	-0.14 (0.161)	0.22 (0.305)	0.15 (0.136)	0.02 (0.016)	-0.16 (0.133)	0.03 (0.117)	0.19 (0.130)	-0.79** (0.401)
Transitional Zone	0.36** (0.152)	0.30 (0.195)	0.74* (0.440)	0.54*** (0.152)	0.06*** (0.018)	0.07 (0.132)	-0.20 (0.156)	0.40** (0.173)	-0.46 (0.550)
Forest Zone	0.01 (0.166)	0.14 (0.254)	-0.09 (0.690)	-0.11 (0.154)	-0.02 (0.018)	0.02 (0.163)	0.26 (0.200)	-0.15 (0.178)	-0.96* (0.495)
National	0.41*** (0.077)	0.38*** (0.098)	0.24 (0.223)	0.22*** (0.071)	0.02** (0.008)	-0.02 (0.069)	-0.15** (0.076)	0.17** (0.073)	-0.77*** (0.202)
Meta-frontier	0.47*** (0.086)	0.64*** (0.126)	-0.02 (0.281)	0.04 (0.077)	-0.01 (0.009)	-0.05 (0.087)	-0.29*** (0.100)	0.16* (0.088)	-0.81*** (0.215)
Cocoyam									
Transitional Zone	0.45* (0.246)	-0.69 (0.428)	-0.11 (1.029)	-0.17 (0.221)	-0.02 (0.025)	0.92*** (0.337)	0.37 (0.379)	0.57* (0.327)	-49.53 (0.000)
Forest Zone	0.09 (0.123)	0.33* (0.198)	0.36 (0.412)	0.13 (0.110)	0.01 (0.013)	-0.17 (0.109)	0.07 (0.119)	0.06 (0.133)	-0.39 (0.449)
National	0.27** (0.131)	0.25 (0.200)	0.52 (0.422)	0.09 (0.113)	0.01 (0.013)	0.00 (0.118)	0.06 (0.127)	0.12 (0.143)	-0.62 (0.660)
Meta-frontier	0.08 (0.141)	-0.14 (0.252)	0.63 (0.505)	0.25 (0.169)	0.03 (0.019)	-0.16 (0.124)	-0.19 (0.141)	0.02 (0.161)	-0.55 (0.835)
Plantain									
Transitional Zone	-0.02 (0.161)	0.29 (0.244)	0.48 (0.576)	0.05 (0.159)	0.01 (0.018)	-0.24* (0.137)	-0.36* (0.183)	-0.09 (0.195)	-3.61 (8.208)
Forest Zone	0.00 (0.077)	0.20* (0.118)	0.49* (0.294)	0.04 (0.079)	0.00 (0.009)	-0.23*** (0.080)	-0.13 (0.089)	-0.02 (0.094)	-
Coastal Savanah	0.04 (0.283)	0.06 (0.363)	1.18 (1.079)	0.37 (0.270)	0.04 (0.032)	0.01 (0.249)	-0.21 (0.363)	-0.47 (0.396)	0.54 (1.051)
National	-0.02 (0.066)	0.20** (0.099)	0.39 (0.239)	0.06 (0.065)	0.01 (0.007)	-0.22*** (0.063)	-0.14* (0.075)	-0.06 (0.078)	-0.39 (0.335)
Meta-frontier	-0.09 (0.085)	0.07 (0.126)	-0.32 (0.280)	0.02 (0.091)	0.00 (0.011)	-0.06 (0.081)	-0.37*** (0.140)	-0.20* (0.101)	1.02*** (0.394)

Significance levels: * p<0.10, ** p<0.05, ***p<0.01
Data Sources: Ghana Living Standards Surveys [wave 1-7] and Ghana Socioeconomic Panel Survey [wave 1-2

Table S8: Ghanaian Starchy Staple Production Technology Level and Technical Efficiency Parameters Across Seasons (1987-2017)

Season	-	TGR	TE	MTE	GAP	-	TGR	TE	MTE	GAP
		Cassava					Yam			
1987/88		0.717	0.455	0.324	28.30%		0.663	0.401	0.268	33.70%
1988/89		0.998	0.490	0.493	0.16%		0.678	0.572	0.364	32.16%
1990/91		1.000	0.833	0.836	0.03%		0.530	0.466	0.255	47.02%
1991/92		1.000	0.850	0.852	0.03%		0.642	0.450	0.295	35.81%
1997/98		0.969	0.700	0.682	3.13%		0.381	0.812	0.216	61.89%
1998/99		0.967	0.690	0.669	3.29%		0.622	0.661	0.365	37.84%
2004/05		0.981	0.744	0.728	1.94%		0.566	0.562	0.311	43.40%
2005/06		0.982	0.748	0.736	1.84%		0.588	0.568	0.324	41.19%
2009/10		0.702	0.751	0.518	29.84%		0.837	0.535	0.449	16.34%
2011/12		0.738	0.645	0.457	26.24%		0.594	0.512	0.298	40.60%
2012/13		0.757	0.645	0.476	24.30%		0.580	0.489	0.267	42.01%
2014/15		0.832	0.592	0.488	16.84%		0.406	0.427	0.187	59.37%
2015/16		0.876	0.443	0.389	12.38%		1.000	0.496	0.484	0.00%
2016/17		0.879	0.459	0.404	12.08%		1.000	0.550	0.526	0.00%
Season		Cocoyam					Plantain			
1987/88		0.981	0.567	0.561	1.90%		0.719	0.410	0.298	28.08%
1988/89		-	0.534	-	-		0.534	0.404	0.209	46.56%
1990/91		0.653	0.494	0.316	34.75%		0.859	0.704	0.604	14.07%
1991/92		0.576	0.643	0.301	42.44%		0.837	0.674	0.563	16.28%
1997/98		0.784	0.590	0.467	21.63%		0.854	0.662	0.558	14.63%
1998/99		0.817	0.603	0.502	18.30%		0.839	0.675	0.564	16.05%
2004/05		0.782	0.415	0.336	21.79%		0.890	0.659	0.584	11.03%
2005/06		0.794	0.410	0.318	20.57%		0.905	0.624	0.561	9.48%
2009/10		1.000	0.564	0.557	0.00%		0.995	0.724	0.721	0.50%
2011/12		0.665	0.437	0.294	33.55%		1.000	0.587	0.578	0.00%
2012/13		0.676	0.419	0.286	32.38%		1.000	0.576	0.571	0.00%
2014/15		0.822	0.556	0.448	17.81%		0.767	0.544	0.413	23.29%
2015/16		0.749	0.095	0.065	25.08%		0.831	0.439	0.356	16.89%
2016/17	-	0.828	0.271	0.221	17.23%	-	0.852	0.452	0.382	14.76%

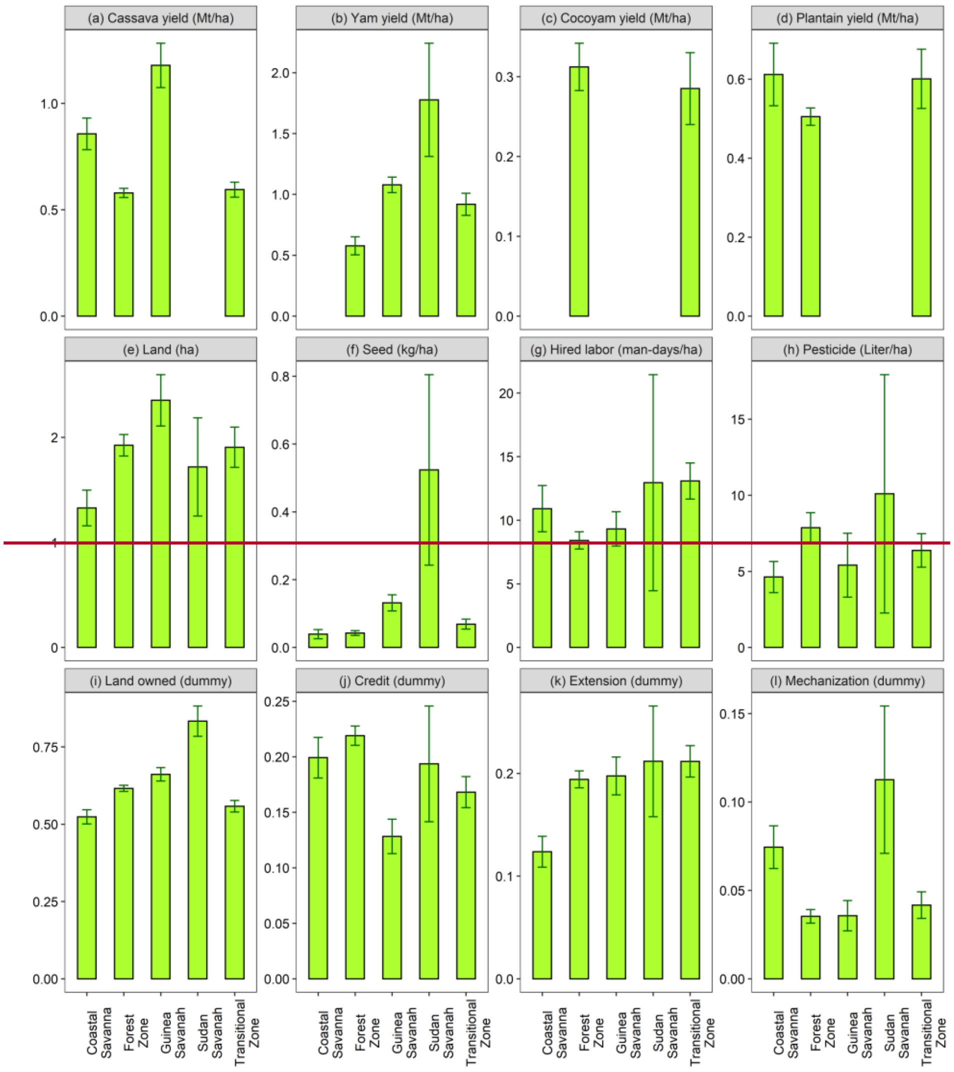

Figure S1a: Ecological variation in Starchy Staple production yield, input, and enabling environment factors in Ghana (1987-2017)

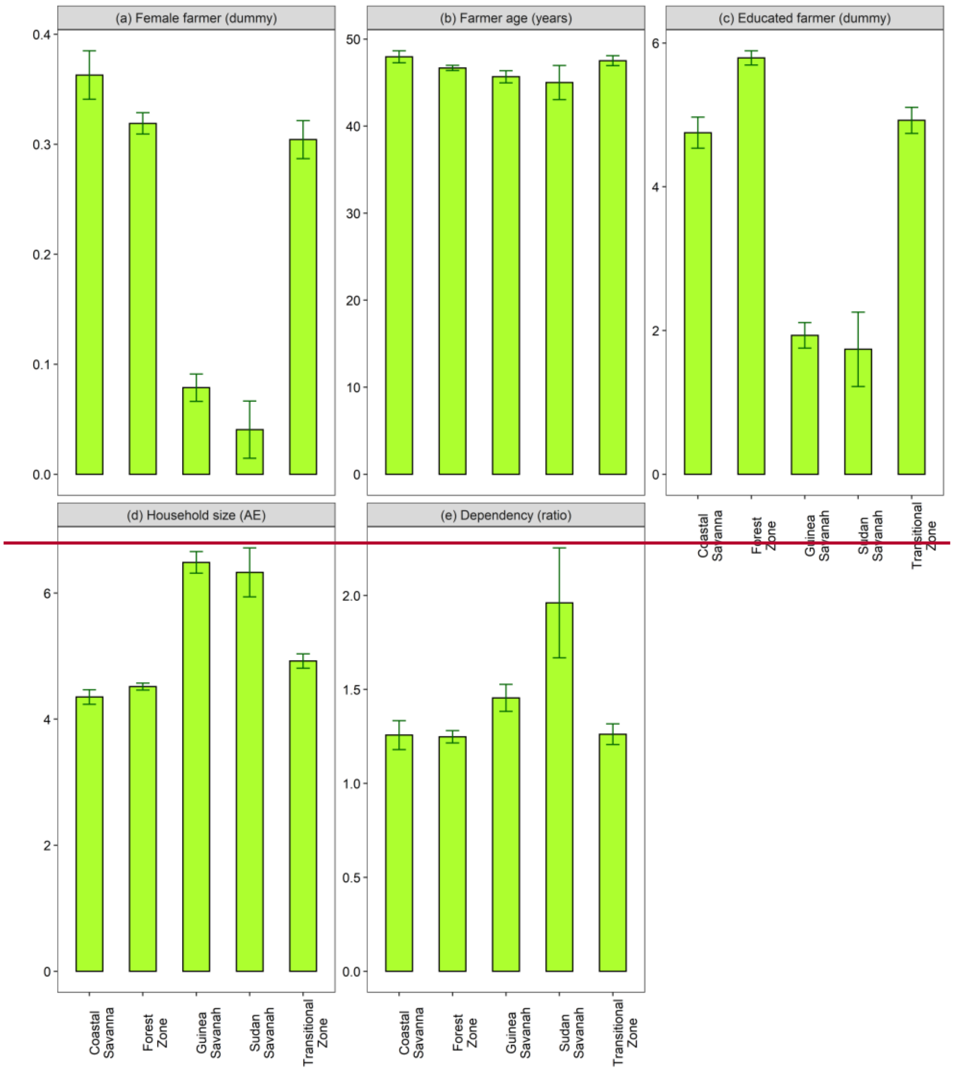

Figure S1b: Ecological variation in Starchy Staple Farmer and Household Demographics factors in Ghana (1987-2017)

Note S1. Construction of variables

Female (dummy): Across all surveys, this variable takes on a value of unity if the actual farmer is female, zero otherwise.

Age (years): Across all surveys, this variable was taken as the recorded age of the farmer whose production information was analyzed.

Education (years): Across all surveys, this variable was taken as the recorded years of education of the farmer whose production information was analyzed.

Land owned (dummy): Across all surveys, this variable takes on a value of unity if the actual farmer owned portions of the land under cultivation.

Land (ha): Across all surveys, this variable is taken as the farmer recalled land under production, converted to hectares.

Yield (kg/ha): Across all surveys, this value was taken as the quantity of harvest divided by the land under cultivation of a given starchy staple.

Family labor (AE): Across all surveys, family labor was calculated as the total adult equivalence (AE) attributable to members aged 15 years and older.

Hired labor (man-days): For GSPS, input usage was reported at the plot level, thus the quantity of hired labor was taken as the farmer recalled quantity of paid labor in man-days, multiplied by the ratio of production value to total production value for a given plot. These were then summed for a given farmer and starchy staple. Information on inputs in all seven GLSSs were collected at the household as expenditures. Consequently, for each farmer, the expenditure for hired labor was calculated as the respective share of that farmer's crop production value in total household crop harvest value; subsequently, these were divided by the annual minimum agricultural daily wage to approximate the man-days of hired labor used.

Pesticide (Liter): Same as hired labor, the only exception is that, where necessary, the annual price for fertilizer (GHC/kg) was used to convert pesticide expenditures to liters.

Household Size (AE): Across all surveys, household size in adult male equivalence (AE) was computed by dividing the total energy requirements of the household by 2,250 kcal.

Dependency (ratio): Given the AE of its members, the dependency ratio of the household was then calculated as the total AE attributable to members aged between 17 and 70 years divided by that for those aged below 18 years or above 69 years.

Credit (dummy): Across all surveys, this variable takes on a value of unity if at least one household member applied for a loan or received inputs on credit, zero if not, and two if no credit information was collected in the survey. A value of two was assigned to data drawn from the GLSS1, GLSS2, GLSS3, and GLSS4 surveys since they were never collected. In this case, the coefficient on category one is taken as the credit effect in any econometric estimation.

The extension (dummy): Across all surveys, this variable takes on a value of unity if the actual farmer is in an enumeration area (as defined by the survey) in which at least one farmer reported having received extension services from MoFA or NGOs. Also, where community information was collected, this variable takes on a value of unity if there was an extension agent or office in that community. This variable takes on a value of zero if the above conditions are not met.

Note S2. Test for Model Specification

Skewness test: The Coelli [1] and Schmidt and Lin [2] skewness tests for OLS residuals are negative for all the models, suggesting that the production variation of starchy staples across Ghana's ecologies is negatively skewed. The Gutierrez [3] test confirmed the conclusions of the Coelli [1] and Schmidt and Lin [2] tests. The outcome of these tests indicates that the negative skewed error needed for the justification of SFA is strong. Furthermore, the null hypotheses $H_o: \alpha = 0$ was also rejected, thus, providing further justification for the technical inefficiency functions.

The similarity of production frontiers across ecologies: To statistically check the similarities of the production frontiers for a given starchy staple across the different ecologies, the study applied a generalized likelihood ratio test. The log-likelihood values under the null hypothesis of a uniform technology were all smaller than that of the alternate hypothesis of heterogeneous technology. The differences in the log-likelihood values led to test statistics that soundly rejected the null hypothesis, and thus, support the fact that starchy staple farmers are operating under heterogeneous technologies along ecological lines.

Production variance: The proportion of production variance due to inefficiency (γ) ranged from 0.20 to 0.99 for the ecology frontiers; for a given crop, this was always higher than that of the Meta-frontier. The γ values suggest that a considerable amount of the observed variation in output for the ecology frontiers [Meta-frontier] could be attributed to inefficient use of inputs [technological gaps]. The range of γ for the Meta-frontier also suggests that the observed variation in output, given the ecology frontiers, could not be attributed to idiosyncrasies. Finally, the SFA model chi-squared test statistics indicate that the models are all significant. In Table C1, we report the summary of estimates for the ecology- and meta-frontier production elasticities for the starchy staples and discuss the details in the following subsections along crop-specific lines.

Note S3. Drivers of Technical Inefficiency and Technology gap

In assessing the factors that influence technical efficiency and technology gaps of starchy staples in Ghana, the key variables considered were farmer characteristics (age, education, and gender) and institutional variables (land ownership, extension service access, credit access, and mechanization service utilization). Table D1 reports the estimates across crops and ecological zones. Results for the case of technical inefficiency are captured by the ecology frontiers and that of the technology gap is captured by the meta-frontier. With TE, negative [positive] values indicate that the variables reduce [increase] technical inefficiency. For the case of TGR, negative [positive] values indicate that the variables reduce [increase] the technology gap.

Formatted: Line spacing: Double, Tab stops: 3.83", Left

The following caveats are to be noted in interpreting the effects of these variables on technical inefficiency and technological gaps. First, these variables were harmonized across different surveys and used mainly as controls in estimating the drivers of differences in starchy staples production outcomes in Ghana. Due to this level of aggregation, we are not confident that there is enough exogenous variation in the control variables to attach real causal interpretation to them. Notwithstanding, given this study's focus on establishing which technological gaps or technical inefficiency contributes the most to production shortfalls, we believe the outcomes of these farmer characteristics and institutional factors can help to direct policies. To this end, we interpret these variables in terms of general associations and not in terms of individual coefficients.

First, the results show that females are doubly disadvantaged in the production of starchy staples as they are both less technically efficient and have higher technology gaps when compared to their male counterparts. These phenomena are well established by similar findings in the literature for rice [1], yam [2], and vegetables [3].

Turning to the age of the farmer, the results show that age has generally positive levels and long-run associations with farmers' TE and technological gaps. Thus, older farmers tend to be more inefficient in starchy staples production and express greater gaps in adopting modern technologies. Owusu Asante et al. [2] found similar effects of age on yam farmers' technical inefficiency in Ghana's Ashanti and Brong-Ahafo regions. Similarly, farmers' years of education showed mostly negative short- and long-run associations with technical efficiency and adoption of superior technologies.

Where statistically significant, general results for land ownership shows that farm households that own lands are more technically efficient than non-owners of land. This highlights the positive effect of tenure security: we would expect landowners to be more motivated to undertake management practices that improve soil fertility and land structure, and that eventually lead to more efficient crop production than non-owners of land [4]. On the other hand, the effect of ownership on bridging technology gaps appears inconclusive. On its own, land ownership may not be enough incentive to adopt and maintain the application of modern technologies. Modern

production technologies may require significant extra labor and capital investment to apply. In such instances, resource-constrained farmers may rather opt for conservative land management techniques that improve technical efficiency. Access to agricultural extension services often plays a key role in helping farmers improve their crop production by promoting good agronomic practices and the adoption of better technology that improves efficiency. Our results confirm this *a priori* expectation by their generally negative associations with TE and TGR. As in this study, Owusu Asante et al. [2] also found that extension access reduced production inefficiency.

Access to credit is expected to enable farmers to acquire supporting inputs necessary to increase efficiency and productivity. The results in Table D1 show generally positive associations with both TE and TGR. This implies that farmers with access to credit are not only less efficient but also less likely to apply the best production technologies relative to their credit-rationed counterparts. The counterintuitive finding that credit decreases TE and increases TGR is difficult to explain from the available data, but one possible prediction could relate to the exact use of the credit accessed. Do farmers invest credit funds in their starchy staple production? Oftentimes, farmers use the credit for other purposes than farm investment [5]. For mechanization, the results show that access correlates directly to reduced technical inefficiency but inversely with TGR. This mixed result is not surprising and may be reflective of the heterogenous access to mechanized services across production ecologies and among farmers in Ghana [6]. That said, we are mindful of claiming too much inference to this finding given the conditions.

Formatted: Font: Italic

Formatted: Line spacing: Double, Tab stops: 3.83", Left

Appendix S References

1. Coelli T. Estimators and hypothesis tests for a stochastic frontier function: A Monte Carlo analysis. J Product Anal. 1995;6: 247–268. doi:10.1007/BF01076978

2. Schmidt P, Lin T-F. Simple tests of alternative specifications in stochastic frontier models. J Econom. 1984;24: 349–361. doi:10.1016/0304-4076(84)90058-7

3. Gutierrez RG, Carter S, Drukker DM. On boundary-value likelihood-ratio tests. Stata Tech Bull. 2001;10. Available: https://econpapers.repec.org/article/tsjstbull/y_3a2001_3av_3a10_3ai_3a60_3asg160.htm

4. Owusu V, Donkor E, Owusu-Sekyere E. Accounting for the Gender Technology Gap Amongst Smallholder Rice Farmers in Northern Ghana. J Agric Econ. 2018;69: 439–457. doi:10.1111/1477-9552.12236

5. Owusu Asante B, Villano RA, Battese GE. The effect of the adoption of yam minisett technology on the technical efficiency of yam farmers in the forest-savanna transition zone of Ghana. African J Agric Resour Econ. 2014.

6. Tsiboe F, Asravor J, Osei E. Vegetable Production Technical Efficiency and Technology Gaps in Ghana. African J Agric Resour Econ. 2019;14. Available: http://afjare.org/wp-content/uploads/2020/01/3.-Tsiboe-et-al.pdf

7. Besseah FHFA, Kim S. Technical Efficiency of Cocoa Farmers in Ghana. J Rural Dev. 2014;37: 159–182. Available: http://ageconsearch.umn.edu/record/196615?ln=en

8. Appiah-Twumasi M, Donkoh SA, Ansah IGK. Farmer innovations in financing smallholder maize production in Northern Ghana. Agric Financ Rev. 2019;80: 421–436. doi:10.1108/AFR-05-2019-0059

9. Ministry of Food and Agriculture [MOFA]. Agriculture in Ghana: Facts and figures (2018). 2019.

Formatted: Left, Indent: Left: 0", Hanging: 0.44", No widow/orphan control, Don't adjust space between Latin and Asian text, Don't adjust space between Asian text and numbers

Responses to reviewer comments on the manuscript PONE-D-22-23407_R1

Reviewer #2:

Authors appreciation: *Dear Reviewer, we appreciate your time and commitment to provide important comments on a previous of our paper. Your comments have been useful in helping us to improve upon the paper. We have carefully considered your comments and attempted to address them according to our understanding of your comments and to the best of our ability. We hope that the responses we provide below can be considered sufficient in addressing those comments. Nonetheless, we are open and ready to work on any further comment or suggestion that you deem a necessary addition to the manuscript. Thank you.*

Reviewer comment 1: The key aim of the stochastic Meta-frontier technique is technological comparison. How was this addressed in the work.

Author response*: Thank you for this comment. Throughout the manuscript, we compare the technologies across different ecological zones in Ghana. In fact, we adopted the meta-frontier solely for this process. To provide more insight into how we did this technological comparison, we have done the following two things:*

First, we have now combined the theoretical and empirical model sections into one section (Methodology) to streamline the writeup and directly show how we implement the stochastic meta-frontier technique. The specific paragraph that highlights how we used the Meta-frontier technique for technological comparison is as follows:

"Several studies have shown that farmers in Ghana operate under spatially differentiated technologies [8,47] which have been linked to variability in Ghana's agroecology. We present Figures in the online appendix and statistical tests in Table 2 that suggest that production characteristics do vary across ecologies and starchy staples. Thus, it is important to account for these spatial heterogeneities driven by agroecological zones when investigating the production performance of starchy staple farmers operating at different levels relative to the best practice frontier. According to Battesse et al. [23], not accounting for these technological differences could lead to false attribution of production shortfalls. In the estimation of our empirical model, we group farmers into agroecological technology groups and apply the MSF following Huang et al. [25] two-step method as outlined in the theoretical framework section above. The MSF is estimated separately for each crop, so we use the physical measure of total output and input (log-transformed) used by the farmer. Thus, all outputs and inputs are not standardized by farm size.

Second, throughout the results and discussions, we highlight the technological differences across the ecologies in terms of the technological gaps.

Reviewer comment 2: Improvements in crop productivity by closing production gaps in rural economies are generally touted as an important pathway to achieving three main developmental goals, namely food security, reduced poverty, and sustainable resource utilization ==> This statement should be deleted in the conclusion because authors did not address that

Author response*: Thanks for your critical reading and pointing out this. We agree with you that the statement is immaterial to our paper and have thus adhered to your suggestion by expunging it from the revised manuscript.*

Reviewer comment 3: Quote Onumah, Onumah, Al-Hassan, Bruemmer (2013) to support literature on Meta-frontier approach in the Cocoa industry

Thank you for this comment we have now added the recommended citation to the introduction

Reviewer #3:

Authors appreciation: *Dear Reviewer, despite your busy schedules, you still manage to make time to provide such a comprehensive comment on our paper. We are so grateful to you for raising those useful comments that have been important pointers in helping us to bring significant improvements to our paper. We have carefully reviewed your comments and attempted to address them according to our understanding of these comments. We have committed much effort in addressing your comments. We hope that the responses we have provided below can be considered sufficient, and that our paper can be considered for publication in the PLoS One journal. Nonetheless, we are open and ready to work on any further comment or suggestion that you deem fit to bring the manuscript to an acceptable level for publication in the PLoS One journal. Thank you.*

General Comment:

This study investigates yield gaps in starchy staples in Ghana based a meta-frontier efficiency analysis. Mainly, the authors use household survey data to investigates the yield gaps of the starchy staples across various agroecological zones in Ghana. Their analysis highlights differences in yield gaps across the agroecological zones. These differences are mainly associated with technical inefficiency than technological differences, which is an interesting finding. However, it is not clear what does technical inefficiency mean. Does it refer to technical deficiencies? It would have been great when the authors could have highlighted the key reasons behind the so-called inefficiency. In my view, this is the main shortcoming of the manuscript, other shortcomings are listed below. Thus, I suggest considering it for publication after addressing the shortcomings.

Author response: *We are grateful for your dedicated effort to helping us improve our paper by critically reading through our manuscript and identifying areas for improvement. Your major comment, which borders on the lack of clarity on technical efficiency and the reasons behind their existence has been given serious attention. We hope that our responses are sufficient to address this shortcoming. We have addressed your major concern in the following ways:*

- *In the methodology section, we have provided further explanation of technical efficiency. Specifically, on page 10 lines 217-221, we have inserted the following explanation of technical efficiency:*

  "Technical efficiency relates to how much output can be obtained from a given input, such as a worker or a machine, or a specific combination of inputs. In other words, it the effectiveness with which a given set of inputs is used to produce an output. If the farmer is ineffective in converting the inputs to the desired outputs, the farmer is said to be technically inefficient. The extent of inefficiency is measured by how much of the desired output is lost due to improper combination of the outputs"

- *In the discussion section, we have outlined possible reasons that influence technical efficiency. Specifically, we include the following statement in the discussion on page 33 lines 631-633:*

"On the other hand, technical efficiency is driven by the ability and willingness of the farmer to properly use and combine the available input sets. Thus, management practices and enabling environment as opposed to new technologies, are key determinants of technical efficiency."

Reviewer comment: First, the abstract could be longer. Currently, it does not highlight the research gaps, method applied, and implications of the study.

Author response: *Thank you for this recommendation. We have now extended the abstract to 200 words, which reads as follows:*

"Starchy staples are a major source of livelihood support for farmers, traders, and processors who participate in these crops' value chains, while also providing staple food to many people, especially the less affluent in society. Despite this position, the productivity figures of starchy staples are low. We use a unique data set and meta-frontier efficiency analysis to assess whether the production shortfalls of major starchy staple crops in Ghana could be attributed to technical inefficiency, technology gaps or both. Results show strong evidence of about 50% production shortfall for cassava, yam, cocoyam, and plantain. For cassava production, the Guinea Savannah zone has the most superior technology, with a technology gap ratio of 0.92, while yam production is more technically efficient in the Sudan Savannah zone, with a technical efficiency score of 0.67. Cocoyam production is more technically efficient (0.56) in the Transition zone, but yam is more technically efficiently produced in the Coastal Savannah zone of Ghana. These results show that production shortfall is more influenced by pure farmer technical inefficiencies (about 45%) rather than by technology gaps (about 20%) along ecological lines. Thus, the sector could benefit from improvements in farmer managerial skills and efficient use of existing technologies".

Reviewer comment: Second, the introduction section needs to be improved, mainly be updating the references based on the recent studies. See the specific comments for the details.

Author response*: Thank you for this comment. We have updated the introduction with several relevant and more current references including the one you suggested. Specifically, we have now added the following references:*

- Pradhan P, Fischer G, van Velthuizen H, Reusser DE, Kropp JP. Closing Yield Gaps: How Sustainable Can We Be? Gonzalez-Andujar JL, editor. PLoS One. 2015;10: e0129487. doi:10.1371/journal.pone.0129487
- Onumah JA, Onumah EE, Al-Hassan RM, Brümmer B. Meta-frontier analysis of organic and conventional cocoa production in Ghana. Agric Econ (Czech Republic). 2013.
- Ladha JK, Jat ML, Stirling CM, Chakraborty D, Pradhan P, Krupnik TJ, et al. Achieving the sustainable development goals in agriculture: The crucial role of nitrogen in cereal-based systems. Advances in Agronomy. 2020. pp. 39–116. doi:10.1016/bs.agron.2020.05.006

Reviewer comment: Third, it would be better to separate the result and discussion section. The authors could highlight the key findings in the result section, with their immediate discussion. In the discussion section, they could highlight the key novelties of the studies, comparing their results with other studies, discussing the limitation and policy implications. Moreover, the authors could have highlighted the reasons behind the differences in the yields based on the survey data. Currently, these reasons are diluted in the huge text. Even these reasons are vaguely mentioned as technical inefficiency in the conclusion. It would be better for readers and policy makers to pinpoint these technical inefficiencies.

Author response: *We are grateful for this comment. Separating the combined results and discussion sections was a difficult thing to do, but we adhered to your useful comment and did so. After doing so, we*

agree with you that it is better we did. The results section now mainly focus on reporting the outcomes from the analysis, while the discussion section is tilted towards bringing in arguments that address our main research objective. In the modified manuscript, we dedicate a section for discussion where we highlight the key trends in the starchy staple production shortfalls and discuss the possible reasons that lead to the observed trends. We look at it from both management perspectives and policy frameworks.

Specific comments:

Reviewer comment: L43-36: Please update these numbers for the recent FAO report in 2022, see https://www.fao.org/publications/sofi/2022/en/

Author response*: Thank you for this comment. On page 3 lines 51-53, the citation has been updated. The modified text now reads as follows:*

> *"In 2022, the Food and Agriculture Organization's document on the State of Food Security and Nutrition in the World projected that between 702 and 828 million people faced hunger in 2021, with Africa and Asia being the main hosts of these challenges [1]."*

Reviewer comment: L52-59: Please update these statements based on the recent FAO report in 2022, see https://www.fao.org/publications/sofa/2022/en/. Here, the authors may highlight the crop yield gaps in Africa, e.g., see Pradhan et al. 2015 (https://doi.org/10.1371/journal.pone.0129487)

Author response*: Thank you for this comment. On page 3 line 64 to page 5 line 73, the citation has been updated. The update also includes information on crop yield gaps in Africa that were collected from Pradhan et al. (2015). The modified text now reads as follows:*

> "*In many parts of the developing world, smallholder farmers mostly organize the production of staples. The 2022 FAO report on "The State of Food and Agriculture" point to low agricultural productivity as characterizing smallholder production systems [3]. Based on global projections, Pradhan et al. [4] conclude that Africa has the highest crops yield gaps, and that closing these gaps has the potential to improve food and nutrition security in Africa. Thus, productivity growth and resource use efficiency are important causal pathways to increased food production and access for the poor in these areas. Notably, Pradhan et al. [4] attribute the wide yield gaps in crops, especially those cultivated under rainfed, to biophysical factors (e.g., soil quality, agro-climatic, etc.) and socioeconomic factors (e.g., weather-induced yield variability that affect risk attitudes, market access, etc.).*"

Reviewer comment: L67: In stead of footnotes, these numbers could be included in the main text in a condensed form.

Author response*: Thank you for this comment. We have included some of the footnotes directly in the maintain text and only left those that are necessary to remain as such.*

Reviewer comment: L68-77: This paragraph argument that inefficient use of resources as a cause of yield gaps without any citation. Often these gaps are due to lack of resources, e.g., water, seed, and fertilizer , e.g., see Ladha et al. 2020 (https://doi.org/10.1016/bs.agron.2020.05.006).

Author response*: Thank you for this nice pointer. We found the recommended text useful and have incorporated some ideas from it and cited it accordingly. Also, following your advice to separate the Results and Discussion sections, we have highlighted how inefficient use of resources by farmers in our sample*

lead to yield gaps in these sections and buttressed this argument with relevant literature. A few other studies in Ghana documenting inefficient use of resources as a cause of yield gaps in the subsequent section have been added.

Reviewer comment: L81: May be "Owusu Asante and colleagues [19]" instead of [19] only. Better to mention the author names in such cases.

Author response*: Thanks for the comment and we are sorry for this error. We have now corrected this throughout the MS.*

Reviewer comment: L198: Here, the authors could also refer to SDG 2.3 on doubling crop productivity, contribution to rescue SDG from failing, e.g., see Pradhan 2023 (https://doi.org/10.1093/nsr/nwad015).

Author response*: Thanks for this comment. After reading the suggested paper, we were unsure on how the issues discussed in the paper directly fit or add to the section in question, as that part of the manuscript only summarizes the work by Owusu [10].*

Reviewer comment: L112-210: This is a very long section on literature. It is interesting to read. However, the added value of it is not clear. Instead, it may divert the reader's attention from this study. Thus, it might be better to have very condense justification of the selected method in the method section.

Author response*: Thank you for this suggestive comment. We agree with you that the literature section tended to overlap with the method section in the previous draft. We have therefore expunged the literature section, improved the introduction section and modified the method section to directly address the needs of the paper.*

Reviewer comment: L371: "in the interest of space" is not a valid argument here. The authors have used a lot of space to justify the methods which can be move to SI. The authors would present and exploit the results properly.

Author response*: Thank you for this comment. Based on your recommendation the discussion on the drivers are now part of the main text and we have also removed the text "in the interest of space."*
