# Supplementary material for: Starchy staples production shortfalls in Ghana: Technical inefficiency effects outweigh technological differences across ecologies
Source: PLoS One. 2023 Apr 24;18(4):e0284780. doi: 10.1371/journal.pone.0284780 (PMC10124864; doi:10.1371/journal.pone.0284780)
Supplement: Attachment — Submitted filename: October 2022.docx [file pone.0284780.s002.docx]

Comments for the paper

“Starchy staples production shortfalls in Ghana: Technical inefficiency effects outweigh technological differences across ecologies”

The paper highlights an important topic in agricultural technology literature. The authors assess the production shortfalls of a selected number of major starchy staple crops in Ghana. They utilize meta-frontier efficiency analysis using secondary data obtained from the national socio-economic panel data sets.

An interesting finding of the study is that the biggest proportion of production shortfall is influenced by farmer technical inefficiencies rather than technology gaps. This is an interesting finding as it questions the assumption of farmers’ rationality in the adoption and utilization of agricultural technologies to enhance production and productivity. The recommendation is that farmers' managerial skills and efficient use of existing technologies is very critical for enhanced adoption and impact of agricultural innovations. I enjoyed reading this paper.

The paper is well written and therefore has very few comments.

Comments:

1. Datasets: the authors mentioned in the introduction that the dataset was pooled, but they indicated having a panel dataset in the methodology section. Panel data enhances the efficiency of the results and hence confidence in the generalization of the results; they should make this clear
2. Comparison of production shortfalls across different agro-ecological zones: I probably missed how the authors controlled for differences of various agro-ecologies favoring different crops, i.e. production differences should be compared across similar agroecological zones and using similar technologies
3. Consider adding a paragraph or two in the conclusions section highlighting the key shortcomings of the study (for future research) and key policy recommendations generated from the study.
