# Supplementary material for: Starchy staples production shortfalls in Ghana: Technical inefficiency effects outweigh technological differences across ecologies
Source: PLoS One. 2023 Apr 24;18(4):e0284780. doi: 10.1371/journal.pone.0284780 (PMC10124864; doi:10.1371/journal.pone.0284780)
Supplement: Attachment — Submitted filename: Responses to Reviewers.docx [file pone.0284780.s004.docx]

5. Review Comments to the Author

Please use the space provided to explain your answers to the questions above. You may also include additional comments for the author, including concerns about dual publication, research ethics, or publication ethics. (Please upload your review as an attachment if it exceeds 20,000 characters)

# Reviewer #1: Comments for the paper.

“Starchy staples production shortfalls in Ghana: Technical inefficiency effects outweigh technological differences across ecologies”

The paper highlights an important topic in agricultural technology literature. The authors assess the production shortfalls of a selected number of major starchy staple crops in Ghana. They utilize meta-frontier efficiency analysis using secondary data obtained from the national socio-economic panel data sets.
An interesting finding of the study is that the biggest proportion of production shortfall is influenced by farmer technical inefficiencies rather than technology gaps. This is an interesting finding as it questions the assumption of farmers’ rationality in the adoption and utilization of agricultural technologies to enhance production and productivity. The recommendation is that farmers' managerial skills and efficient use of existing technologies is very critical for enhanced adoption and impact of agricultural innovations. I enjoyed reading this paper.

The paper is well written and therefore has very few comments.

*We are grateful to you for your compliments on our paper, as well as the insightful comments you raised that helped us to improve on the paper.*

**Comments:**
1. Datasets: the authors mentioned in the introduction that the dataset was pooled, but they indicated having a panel dataset in the methodology section. Panel data enhances the efficiency of the results and hence confidence in the generalization of the results; they should make this clear

*Thank you for this comment. Our data comes from two main sources; the seven rounds of the GLSS which are repeated cross section and the two waves of GSPS which constitute a panel. The GSPS constitute only 16% of the sample. Thus, most of the sample is a repeated cross-section. The time dimension of the GSPS is very short (only two seasons and they are four years apart). In the data section of the paper, we do indicate that while the GSPS constitutes a panel, households are resampled for each GLSS round, hence not a panel. Consequently, since a large proportion of the data is from the GLSS, the harmonized data by Tsiboe (2020) can be viewed as a cross-sectional sample of the population of Ghanaian starchy staple farmers at roughly five-year intervals.*

*The specific sentences in the paper we have updated on page 11 lines 235-239 to reflect these views are*

*It is worth noting that while the GSPS constitutes a panel, households are resampled for each GLSS round, hence repeated cross-sectional data. Consequently, since a large proportion of the data is from the GLSS (84%), the harmonized data by Tsiboe (2020) can be viewed as a cross-sectional sample of the population of Ghanaian starchy staple farmers at roughly five-year intervals.*

2. Comparison of production shortfalls across different agro-ecological zones: I probably missed how the authors controlled for differences of various agro-ecologies favoring different crops, i.e., production differences should be compared across similar agroecological zones and using similar technologies

*Thank you for this comment. Sorry for the lack of clarity in the previous version of the manuscript on how we controlled for differences in various agro-ecologies favoring different crops. For each crop, we do so by estimating separate production functions for each agroecological zone in the first stage. This is at the heart of the analysis. The specific paragraph in the paper we have added right after the empirical model description on page 17 lines 355 – 367 to make this clear is*

*Several studies have shown that farmers in Ghana operate under spatially differentiated technologies (Asante et al. 2019; Asravor et al. 2019) which has been linked to variability in Ghana’s agroecology (see Figure 1). We present Figures in the appendix and statistical tests in Table 1 that suggest that production characteristics do vary across ecologies and starchy staples. Thus, it is important to account for these spatial heterogeneities driven by agroecological zones when investigating the production performance of starchy staple farmers operating at different levels relative to the best practice frontier. According to (Battese, Rao, and O’Donnell, 2004), not accounting for these technological differences could lead to false attribution of production shortfalls. In the estimation of our empirical model, we group farmers into agroecological technology groups and apply the Meta-Stochastic-frontier (MSF) following Huang et al. (2014)’s two-step method as outlined in the theoretical framework section above. The MSF is estimated separately for each crop, so we use a physical measure of total output and input used by the farmer. Thus, all output and input are not standardized by farm size.*

3. Consider adding a paragraph or two in the conclusions section highlighting the key shortcomings of the study (for future research) and key policy recommendations generated from the study.

*Thank you for this comment. We do agree that our study has a few shortcomings that future research can take and evaluate how those change or reaffirm our core findings. The key ones are that the scores are partly driven by variability in each sample, and endogeneity of input use. While the first can be addressed by using new data sources, it will be very unlikely that any other data can match the spatiotemporal extent of the one used in the present study. On the issue of endogeneity, one can reduce this by using input/output price data instead to estimate a cost or profit function.*

*On the issue of policy recommendations, one of the authors has a disclosure that precludes us from making direct policy recommendations. Ideally, we are of the view that the ongoing Planting for Food and Jobs program of the Government of Ghana is an important entry point to reinforce extension services as well as enhance the supply of improved planting materials to starchy staple crop farmers. This means that the Ministry of Food and Agriculture in Ghana could investigate the documentation of lessons from the frontier enhancing technologies and practices in high-performing ecological zones, improve them further and disseminate such improved technologies to less-performing agro-ecologies, guided of course by contextual factors.*

*Because of the disclosure, we cannot name governmental agencies (here, Ministry of Food and Agriculture in Ghana) or reference specific policies needing modifications (here, Planting for Food and Jobs program of the Government of Ghana). Without doing any of these we have now modified our policy implications on page 27 line 597 to page 28 line 605 to read:*

*“**We generally find that across all the crops considered, the technology gap across ecological lines is minimal but pure farmer technical inefficiency is chronic. This means that the production shortfalls for starchy stables in Ghana could be reduced if pure farmer technical inefficiency is adequately addressed. Existing policies, aimed at improving the technology level (e.g., those supplying improved planting materials and subsidized inputs) should also emphasize the efficient use of these technologies. Stakeholders could investigate the documentation of lessons from the frontier enhancing technologies and practices in high-performing ecological zones, improve them further and disseminate such improved technologies to less performing agro-ecologies, guided of course by contextual factors”*

# Reviewer #2:

*We are grateful to this reviewer for taking the time to thoroughly read our paper and providing the great comments that helped us to improve upon the initial draft we submitted.*

**Comments**

1. "Staple crop production in developing Africa provides both food and income to participating households, hence closing the yield gaps is crucial to improving food security and nutrition" - This is not important in the abstract

*Thank you for your suggestion. We have deleted the sentence as you suggested. The substantive message of the abstract is not lost after deleting it.*

2. Be specific on the FAO reports in line 47+

*We have now inserted 2017 FAO Report on the State of Food and Agriculture that makes the claims in the statement. This can be found on page 3 lines 53-54.*

3. The reason underlining your study is weak. A better justification would do. This reason provided is different from the main objective of the paper.

*Thank you for this comment. On page 5 we have modified our justification to read*

*“In the last two decades, research on productivity and efficiency analyses in Ghana is highly skewed towards cereal crops. In principle, the starchy staples have received little attention, against the backdrop of the critical roles these crops play, and the significant yield gaps observed in practice. Except for a few studies (Adaku et al., n.d; Tsiboe, 2021; Tsiboe et al., 2021, 2019), all efficiency studies in Ghana used data based on a single season, and data size is limited to a few villages or at best a region. This data limitation and lack of locational heterogeneity masks important lessons that could emerge from* *analyzing a more comprehensive and heterogeneous sample. For example, knowledge on whether technology gaps across ecologies or resource use inefficiencies among farmers contribute most to production shortfalls and how these evolve is not yet clear. Such incomplete information might limit scientific progress, and serve as a barrier to societal development, in the sense that policymakers may be inadequately or wrongly informed. Focusing on starchy staples, this study addresses most of these limitations by accommodating multiple seasons and heterogeneity in ecological technology adoption.”*

4."The TE estimates showed that male farmers were marginally more efficient because of higher seeding and fertilizer application rates, while the MTE and TGR showed that female-managed farms were closer to the MSF than that of their male counterparts" How was this estimated? Were they considering two variables or same? Revisit the statement

*Thank you for this comment. Owusu et al (2018) considered one gender variable which was used in a two-stage estimation procedure. The TE estimates were generated in the first using their pooled sample whiles the TGR and MTE were estimated along gender lines using a linear programming procedure in the second stage.*

*We have revised the statement on page 9 lines 184-190 to read*

*"For instance, Owusu et al. [6] assessed the gender technology gap among smallholder rice farmers by estimating a two-stage Cobb-Douglas MSF function to generate TE, Meta-Technical Efficiency (MTE), and Technology Gap Ratio (TGR) for male and female rice farmers. TE estimates from their first stage showed that male farmers were marginally more efficient because of higher seeding and fertilizer application rates; however, the MTE and TGR scores from the second stage showed that female-managed farms were closer to the MSF than their male counterparts after accounting for group heterogeneities."*

5. "Furthermore, Tsiboe et al. (2019) showed that ecological gaps are very small for pepper, modest for tomato, and high for okra" - What is the main difference between this paper and your current study? Is it only about the crops?

*Thank you for this question. Apart from the different crops, this study includes additional data sources (i.e., the GSPS) and the pattern of our core findings is different from Tsiboe et al. (2019). Tsiboe et al. (2019) found no clear pattern in terms of which of the two, pure farmer technical inefficiency or ecological technology gap, dominates in Ghana’s vegetable sector. On the contrary, we find that the dominance of the two generalizes across all the starchy staples we consider. We generally find that across all the starchy staples considered, the technology gap across ecological lines is minimal but pure farmer technical inefficiency is chronic.*

6. "Given these scales of production, yields were estimated at 0.66, 0.96, 0.31, and 0.53 Mt/ha for cassava, yam, cocoyam, and plantain, respectively. Except for cocoyam, these yields also vary along ecological lines" - These figures should be compared with MoFA standards

*Thank you for this comment. At levels, we do not find it is particularly useful to compare the yields from this paper to MoFA standards. The reason is that the root/tuber yield data from the GLSS used in this study are collected over a recall period of 2 weeks (farmers are asked to provide the quantity harvested in the past two weeks), while MoFA yields published in their annual Facts and Figures reports (Ministry of Food and Agriculture [MoFA], 2019) are based on entire year crop yield. We find, however, that while the level of yield is incomparable, our data reflects the relative yields distribution across locations as shown by the MoFA Facts and Figures.*

7. Your Tables did not tell your readers how the dependent variable in model 6 was captured. Is it in physical or value terms? Since your work is on four starch foods, using physical measure could be misleading. On the other hand, using value terms should be done with care due to inflation differentials.

*Thank you for this comment. The models are estimated separately for each crop, so we use physical measures of output. In the empirical model specification, we indicate that we use log transformed values of physical quantities.*

*We have added the statement on page 17 lines 364-367 that reads*

*“The MSF is estimated separately for each crop, so we use the physical measure of total output and input (log-transformed) used by the farmer. Thus, all outputs and inputs are not standardized by farm size”.*

8. "land contributes the highest returns to yam production in all ecological zones" - This statement is very misleading in this paper. You have your output and input variables in the frontier model normalized by land (ha) and you went ahead to include land in the model? This cannot be correct

*Thank you for this comment. We have not normalized the input and output variables by land. We use total output, but log-transform it for the estimation. The specific change we have made to the paper is reflected in our response to comment 7.*

9. Some diagnostic findings of model fitness and data adequacy should be conducted and mentioned in the discussions and in the conclusion.

*Thank you for this comment. We have all the conventional model diagnostics for this type of analysis in table 2. Our discussion of table 2 presented on page 18 lines 378-389 is*

*“**The diagnostics on the negative skewed error needed for the justification of SFA shown in Table 2 confirmed the appropriateness of the empirical model. Further, test results on the similarity of production frontiers across ecologies support the fact that starchy staple farmers are operating under heterogeneous technologies in the various ecologies. This justifies further inquiry into whether technology gaps across ecologies or resource use inefficiencies among farmers contribute most to production shortfalls. The major learning points include the wide range of the estimated* $\gamma$ *values which suggest that considerable amounts of the observed crop output variation for the ecology-frontiers [meta-frontier] could be attributed to inefficient use of inputs [technological gaps]. The range of* $\gamma$ *for the meta-frontier also suggests that the observed variation in output, given the ecology frontiers, could not be attributed to idiosyncrasies. Also, the SFA model chi-squared test statistics indicate that the models are all significant. A specific discussion of relevant diagnostics is presented in Note S2.”*

10. "The key findings suggest that there are significant shortfalls in starchy staples production among agroecological zones, which could be more attributed to technical efficiencies and less to technology gaps" - Since the efficiencies determine the levels of individuals technologies and the technology gaps measures the distance to the potential technology, it becomes difficult to compare. Authors should really justify this comparison.

*We appreciate your comment on this statement, and we are sorry for not being clear on how we controlled for differences in various agro-ecologies favoring different crops. For each crop, we do so by estimating separate production function for each agroecological zone in the first stage. This is at the heart of the analysis.*

*The specific paragraph in the paper we have added right after the empirical model description on page 17 lines 355-367 for clarity is*

*Several studies have shown that farmers in Ghana operate under spatially differentiated technologies (Asante et al. 2019; Asravor et al. 2019) which has been linked to variability in Ghana’s agroecology (see Figure 1). We present Figures in the appendix and statistical test in Table 1 that suggests that production characteristics do vary across ecologies and starchy staples. Thus, it is important to account for these spatial heterogeneities driven by agroecological zones when investigating the production performance of starchy staple farmers operating at different levels relative to the best practice frontier. According to (Battese, Rao, and O’Donnell, 2004), not accounting for these technological differences could lead to false attribution of production shortfalls. In the estimation of our empirical model, we group farmers into agroecological technology groups and apply the Meta-Stochastic-frontier (MSF) following Huang et al. (2014)’s two-step method as outlined in the theoretical framework section above. The MSF is estimated separately for each crop, so we use the physical measure of total output and input used by the farmer. Thus, all output and input are not standardized by farm size.*

11. Authors should concentrate on their findings to conclude and not quote the works of others.

*Thanks for your comment. We have deleted the references we made to other works in the conclusion and incorporated more implications and policy recommendations based on our findings. For example, on page 27 line 597 to page 28 line 605, we have added the following:*

*“We generally find that across all the crops considered, the technology gap across ecological lines is minimal but pure farmer technical inefficiency is chronic. This means that the production shortfalls for starchy stables in Ghana could be reduced if pure farmer technical inefficiency is adequately addressed. Existing policies, aimed at improving the technology level (e.g., those supplying improved planting materials and subsidized inputs) should also emphasize the efficient use of these technologies. Stakeholders could investigate the documentation of lessons from the frontier enhancing technologies and practices in high-performing ecological zones, improve them further and disseminate such improved technologies to less performing agro-ecologies, guided of course by contextual factors”.*

12. The essence of using the meta frontier model is to compare efficiencies for different technologies. If authors went ahead to estimate ecological gaps, then comparison be clear on technological lines and ecological lines. Use the meta efficiency scores to do this rather than the individual efficiency scores and the gaps.

*Thanks for your comment. We agree with you that the essence of using the meta frontier model is to compare efficiencies for different technologies and the meta efficiency scores are suitable for this purpose. We also agree that clear comparisons along technological and ecological lines are essential for meeting this study’s core objective of understanding whether existing shortfalls in Ghanaian starchy staples production are driven by technological gaps, farmer inefficiencies, or both. Indeed, throughout the Results and Discussion section for the four crops, this study’s approach to meeting its objective has been to highlight and compare the performance in starchy staple production for each ecology engaged in the production of the specific starchy staple using the estimated technology gap ratios, ecology-specific technical efficiency, and the joint effects of these as captured by the meta efficiency scores. Thus, initial comparisons of the gaps and individual efficiency scores lay the foundation for systematically comparing the meta frontier performance along technological and ecological lines.*
