# Supplementary material for: Starchy staples production shortfalls in Ghana: Technical inefficiency effects outweigh technological differences across ecologies
Source: PLoS One. 2023 Apr 24;18(4):e0284780. doi: 10.1371/journal.pone.0284780 (PMC10124864; doi:10.1371/journal.pone.0284780)
Supplement: Attachment — Submitted filename: Responses to Reviewers - PlosOne - R2.docx [file pone.0284780.s006.docx]

# Responses to reviewer comments on the manuscript PONE-D-22-23407_R1

**Reviewer #2:**

**Authors appreciation:** *Dear Reviewer, we appreciate your time and commitment to provide important comments on a previous of our paper. Your comments have been useful in helping us to improve upon the paper. We have carefully considered your comments and attempted to address them according to our understanding of your comments and to the best of our ability. We hope that the responses we provide below can be considered sufficient in addressing those comments. Nonetheless, we are open and ready to work on any further comment or suggestion that you deem a necessary addition to the manuscript. Thank you.*

**Reviewer comment 1**: The key aim of the stochastic Meta-frontier technique is technological comparison. How was this addressed in the work.

**Author response***: Thank you for this comment. Throughout the manuscript, we compare the technologies across different ecological zones in Ghana. In fact, we adopted the meta-frontier solely for this process. To provide more insight into how we did this technological comparison, we have done the following two things:*

*First, we have now combined the theoretical and empirical model sections into one section (Methodology) to streamline the writeup and directly show how we implement the stochastic meta-frontier technique. The specific paragraph that highlights how we used the Meta-frontier technique for technological comparison is as follows:*

*“Several studies have shown that farmers in Ghana operate under spatially differentiated technologies [8,47] which have been linked to variability in Ghana’s agroecology. We present Figures in the online appendix and statistical tests in Table 2 that suggest that production characteristics do vary across ecologies and starchy staples. Thus, it is important to account for these spatial heterogeneities driven by agroecological zones when investigating the production performance of starchy staple farmers operating at different levels relative to the best practice frontier. According to Battesse et al. [23], not accounting for these technological differences could lead to false attribution of production shortfalls. In the estimation of our empirical model, we group farmers into agroecological technology groups and apply the MSF following Huang et al. [25] two-step method as outlined in the theoretical framework section above. The MSF is estimated separately for each crop, so we use the physical measure of total output and input (log-transformed) used by the farmer. Thus, all outputs and inputs are not standardized by farm size.*

*Second, throughout the results and discussions, we highlight the technological differences across the ecologies in terms of the technological gaps.*

**Reviewer comment 2**: Improvements in crop productivity by closing production gaps in rural economies are generally touted as an important pathway to achieving three main developmental goals, namely food security, reduced poverty, and sustainable resource utilization ==> This statement should be deleted in the conclusion because authors did not address that

**Author response***: Thanks for your critical reading and pointing out this. We agree with you that the statement is immaterial to our paper and have thus adhered to your suggestion by expunging it from the revised manuscript.*

**Reviewer comment 3**: Quote Onumah, Onumah, Al-Hassan, Bruemmer (2013) to support literature on Meta-frontier approach in the Cocoa industry

*Thank you for this comment we have now added the recommended citation to the introduction*

# Reviewer #3:

**Authors appreciation:** *Dear Reviewer, despite your busy schedules, you still manage to make time to provide such a comprehensive comment on our paper. We are so grateful to you for raising those useful comments that have been important pointers in helping us to bring significant improvements to our paper. We have carefully reviewed your comments and attempted to address them according to our understanding of these comments. We have committed much effort in addressing your comments. We hope that the responses we have provided below can be considered sufficient, and that our paper can be considered for publication in the PLoS One journal. Nonetheless, we are open and ready to work on any further comment or suggestion that you deem fit to bring the manuscript to an acceptable level for publication in the PLoS One journal. Thank you.*

General Comment:

This study investigates yield gaps in starchy staples in Ghana based a meta-frontier efficiency analysis. Mainly, the authors use household survey data to investigates the yield gaps of the starchy staples across various agroecological zones in Ghana. Their analysis highlights differences in yield gaps across the agroecological zones. These differences are mainly associated with technical inefficiency than technological differences, which is an interesting finding. However, it is not clear what does technical inefficiency mean. Does it refer to technical deficiencies? It would have been great when the authors could have highlighted the key reasons behind the so-called inefficiency. In my view, this is the main shortcoming of the manuscript, other shortcomings are listed below. Thus, I suggest considering it for publication after addressing the shortcomings.

**Author response**: *We are grateful for your dedicated effort to helping us improve our paper by critically reading through our manuscript and identifying areas for improvement. Your major comment, which borders on the lack of clarity on technical efficiency and the reasons behind their existence has been given serious attention. We hope that our responses are sufficient to address this shortcoming. We have addressed your major concern in the following ways:*

- *In the methodology section, we have provided further explanation of technical efficiency. Specifically, on page 10 lines 217-221, we have inserted the following explanation of technical efficiency:*

*“Technical efficiency relates to how much output can be obtained from a given input, such as a worker or a machine, or a specific combination of inputs.* *In other words, it* the effectiveness with which a given set of inputs is used to produce an output. If the farmer is ineffective in converting the inputs to the desired outputs, the farmer is said to be technically inefficient. The extent of inefficiency is measured by how much of the desired output is lost due to improper combination of the outputs*”*

- *In the discussion section, we have outlined possible reasons that influence technical efficiency. Specifically, we include the following statement in the discussion on page 33 lines 631-633:*

*“On the other hand, technical efficiency is driven by the ability and willingness of the farmer to properly use and combine the available input sets. Thus, management practices and enabling environment as opposed to new technologies, are key determinants of technical efficiency.*”

**Reviewer comment:** First, the abstract could be longer. Currently, it does not highlight the research gaps, method applied, and implications of the study.

**Author response:** *Thank you for this recommendation. We have now extended the abstract to 200 words, which reads as follows:*

*“Starchy staples are a major source of livelihood support for farmers, traders, and processors who participate in these crops' value chains, while also providing staple food to many people, especially the less affluent in society. Despite this position, the productivity figures of starchy staples are low. We use a unique data set and meta-frontier efficiency analysis to assess whether the production shortfalls of major starchy staple crops in Ghana could be attributed to technical inefficiency, technology gaps or both. Results show strong evidence of about 50% production shortfall for cassava, yam, cocoyam, and plantain. For cassava production, the Guinea Savannah zone has the most superior technology, with a technology gap ratio of 0.92, while yam production is more technically efficient in the Sudan Savannah zone, with a technical efficiency score of 0.67. Cocoyam production is more technically efficient (0.56) in the Transition zone, but yam is more technically efficiently produced in the Coastal Savannah zone of Ghana. These results show that production shortfall is more influenced by pure farmer technical inefficiencies (about 45%) rather than by technology gaps (about 20%) along ecological lines. Thus, the sector could benefit from improvements in farmer managerial skills and efficient use of existing technologies”.*

**Reviewer comment**: Second, the introduction section needs to be improved, mainly be updating the references based on the recent studies. See the specific comments for the details.

**Author response***: Thank you for this comment. We have updated the introduction with several relevant and more current references including the one you suggested. Specifically, we have now added the following references:*

- Pradhan P, Fischer G, van Velthuizen H, Reusser DE, Kropp JP. Closing Yield Gaps: How Sustainable Can We Be? Gonzalez-Andujar JL, editor. PLoS One. 2015;10: e0129487. doi:10.1371/journal.pone.0129487
- Onumah JA, Onumah EE, Al-Hassan RM, Brümmer B. Meta-frontier analysis of organic and conventional cocoa production in Ghana. Agric Econ (Czech Republic). 2013.
- Ladha JK, Jat ML, Stirling CM, Chakraborty D, Pradhan P, Krupnik TJ, et al. Achieving the sustainable development goals in agriculture: The crucial role of nitrogen in cereal-based systems. Advances in Agronomy. 2020. pp. 39–116. doi:10.1016/bs.agron.2020.05.006

**Reviewer comment**: Third, it would be better to separate the result and discussion section. The authors could highlight the key findings in the result section, with their immediate discussion. In the discussion section, they could highlight the key novelties of the studies, comparing their results with other studies, discussing the limitation and policy implications. Moreover, the authors could have highlighted the reasons behind the differences in the yields based on the survey data. Currently, these reasons are diluted in the huge text. Even these reasons are vaguely mentioned as technical inefficiency in the conclusion. It would be better for readers and policy makers to pinpoint these technical inefficiencies.

**Author response**: *We are grateful for this comment. Separating the combined results and discussion sections was a difficult thing to do, but we adhered to your useful comment and did so. After doing so, we agree with you that it is better we did. The results section now mainly focus on reporting the outcomes from the analysis, while the discussion section is tilted towards bringing in arguments that address our main research objective. In the modified manuscript, we dedicate a section for discussion where we highlight the key trends in the starchy staple production shortfalls and discuss the possible reasons that lead to the observed trends. We look at it from both management perspectives and policy frameworks.*

Specific comments:

**Reviewer comment**: L43-36: Please update these numbers for the recent FAO report in 2022, see [https://www.fao.org/publications/sofi/2022/en/](https://na01.safelinks.protection.outlook.com/?url=https%3A%2F%2Fwww.fao.org%2Fpublications%2Fsofi%2F2022%2Fen%2F&data=05%7C01%7C%7C3c85be82fca7486670b708db05f0c7a5%7C84df9e7fe9f640afb435aaaaaaaaaaaa%7C1%7C0%7C638110304306673495%7CUnknown%7CTWFpbGZsb3d8eyJWIjoiMC4wLjAwMDAiLCJQIjoiV2luMzIiLCJBTiI6Ik1haWwiLCJXVCI6Mn0%3D%7C3000%7C%7C%7C&sdata=7xUTBoZaX9LN1%2BU7fK27LA2AF0M6gsd67SnlngAN2Wc%3D&reserved=0)

**Author response***: Thank you for this comment. On page 3 lines 51-53, the citation has been updated. The modified text now reads as follows:*

*“**In 2022, the Food and Agriculture Organization’s document on the State of Food Security and Nutrition in the World projected that between 702 and 828 million people faced hunger in 2021, with Africa and Asia being the main hosts of these challenges [1]*.*”*

**Reviewer comment**: L52-59: Please update these statements based on the recent FAO report in 2022, see [https://www.fao.org/publications/sofa/2022/en/](https://na01.safelinks.protection.outlook.com/?url=https%3A%2F%2Fwww.fao.org%2Fpublications%2Fsofa%2F2022%2Fen%2F&data=05%7C01%7C%7C3c85be82fca7486670b708db05f0c7a5%7C84df9e7fe9f640afb435aaaaaaaaaaaa%7C1%7C0%7C638110304306673495%7CUnknown%7CTWFpbGZsb3d8eyJWIjoiMC4wLjAwMDAiLCJQIjoiV2luMzIiLCJBTiI6Ik1haWwiLCJXVCI6Mn0%3D%7C3000%7C%7C%7C&sdata=PE2fo%2FgfytSqSiJGy5Wn1ERUT95WbZp7zbw%2FAXioSbo%3D&reserved=0). Here, the authors may highlight the crop yield gaps in Africa, e.g., see Pradhan et al. 2015 ([https://doi.org/10.1371/journal.pone.0129487](https://na01.safelinks.protection.outlook.com/?url=https%3A%2F%2Fdoi.org%2F10.1371%2Fjournal.pone.0129487&data=05%7C01%7C%7C3c85be82fca7486670b708db05f0c7a5%7C84df9e7fe9f640afb435aaaaaaaaaaaa%7C1%7C0%7C638110304306673495%7CUnknown%7CTWFpbGZsb3d8eyJWIjoiMC4wLjAwMDAiLCJQIjoiV2luMzIiLCJBTiI6Ik1haWwiLCJXVCI6Mn0%3D%7C3000%7C%7C%7C&sdata=OXaj3eri%2Bq3b8mv0Z3ZOaesG5PAG9dPxordrZH6ODVQ%3D&reserved=0))

**Author response***: Thank you for this comment. On page 3 line 64 to page 5 line 73, the citation has been updated. The update also includes information on crop yield gaps in Africa that were collected from Pradhan et al. (2015). The modified text now reads as follows:*

“*In many parts of the developing world, smallholder farmers mostly organize the production of staples. The 2022 FAO report on “The State of Food and Agriculture” point to low agricultural productivity as characterizing smallholder production systems [3]. Based on global projections, Pradhan et al. [4] conclude that Africa has the highest crops yield gaps, and that closing these gaps has the potential to improve food and nutrition security in Africa. Thus, productivity growth and resource use efficiency are important causal pathways to increased food production and access for the poor in these areas. Notably, Pradhan et al. [4] attribute the wide yield gaps in crops, especially those cultivated under rainfed, to biophysical factors (e.g., soil quality, agro-climatic, etc.) and socioeconomic factors (e.g., weather-induced yield variability that affect risk attitudes, market access, etc.)*.”

**Reviewer comment**: L67: In stead of footnotes, these numbers could be included in the main text in a condensed form.

**Author response***: Thank you for this comment. We have included some of the footnotes directly in the maintain text and only left those that are necessary to remain as such.*

**Reviewer comment**: L68-77: This paragraph argument that inefficient use of resources as a cause of yield gaps without any citation. Often these gaps are due to lack of resources, e.g., water, seed, and fertilizer , e.g., see Ladha et al. 2020 ([https://doi.org/10.1016/bs.agron.2020.05.006](https://na01.safelinks.protection.outlook.com/?url=https%3A%2F%2Fdoi.org%2F10.1016%2Fbs.agron.2020.05.006&data=05%7C01%7C%7C3c85be82fca7486670b708db05f0c7a5%7C84df9e7fe9f640afb435aaaaaaaaaaaa%7C1%7C0%7C638110304306673495%7CUnknown%7CTWFpbGZsb3d8eyJWIjoiMC4wLjAwMDAiLCJQIjoiV2luMzIiLCJBTiI6Ik1haWwiLCJXVCI6Mn0%3D%7C3000%7C%7C%7C&sdata=Ts%2FkbyKP3ZxpcJwHQW63FsmAuCkdHh6XYFHQgsQARrI%3D&reserved=0)).

**Author response***: Thank you for this nice pointer. We found the recommended text useful and have incorporated some ideas from it and cited it accordingly. Also, following your advice to separate the Results and Discussion sections, we have highlighted how inefficient use of resources by farmers in our sample lead to yield gaps in these sections and buttressed this argument with relevant literature.*  *A few other studies in Ghana documenting inefficient use of resources as a cause of yield gaps in the subsequent section have been added.*

**Reviewer comment**: L81: May be “Owusu Asante and colleagues [19]” instead of [19] only. Better to mention the author names in such cases.

**Author response***: Thanks for the comment and we are sorry for this error. We have now corrected this throughout the MS.*

**Reviewer comment**: L198: Here, the authors could also refer to SDG 2.3 on doubling crop productivity, contribution to rescue SDG from failing, e.g., see Pradhan 2023 ([https://doi.org/10.1093/nsr/nwad015](https://na01.safelinks.protection.outlook.com/?url=https%3A%2F%2Fdoi.org%2F10.1093%2Fnsr%2Fnwad015&data=05%7C01%7C%7C3c85be82fca7486670b708db05f0c7a5%7C84df9e7fe9f640afb435aaaaaaaaaaaa%7C1%7C0%7C638110304306673495%7CUnknown%7CTWFpbGZsb3d8eyJWIjoiMC4wLjAwMDAiLCJQIjoiV2luMzIiLCJBTiI6Ik1haWwiLCJXVCI6Mn0%3D%7C3000%7C%7C%7C&sdata=79U%2BWyEJV3UxAazvglEFQ6Oj69YW0gqxvOcdLWQ8Qvo%3D&reserved=0)).

**Author response***: Thanks for this comment. After reading the suggested paper, we were unsure on how the issues discussed in the paper directly fit or add to the section in question, as that part of the manuscript only summarizes the work by Owusu [10].*

**Reviewer comment**: L112-210: This is a very long section on literature. It is interesting to read. However, the added value of it is not clear. Instead, it may divert the reader’s attention from this study. Thus, it might be better to have very condense justification of the selected method in the method section.

**Author response***: Thank you for this suggestive comment. We agree with you that the literature section tended to overlap with the method section in the previous draft. We have therefore expunged the literature section, improved the introduction section and modified the method section to directly address the needs of the paper.*

**Reviewer comment**: L371: “in the interest of space” is not a valid argument here. The authors have used a lot of space to justify the methods which can be move to SI. The authors would present and exploit the results properly.

**Author response***: Thank you for this comment. Based on your recommendation the discussion on the drivers are now part of the main text and we have also removed the text “in the interest of space.”*
